# Evolutionary history and recombination in the mitochondrial carrier SLC25 superfamily analyzed by similarities in the exon and transmembrane α‐helix sequences

**DOI:** 10.1002/pro.70727

**Published:** 2026-07-20

**Authors:** Magnus Monné, Daniela Valeria Miniero, Rosa Calvello, Antonia Cianciulli, Luigi Palmieri, Ferdinando Palmieri

**Affiliations:** ^1^ Department of Biosciences, Biotechnology and Environment University of Bari Aldo Moro Bari Italy; ^2^ Department of Health Sciences University of Basilicata Potenza Italy; ^3^ Department of Medicine and Surgery LUM University Giuseppe Degennaro Casamassima Italy; ^4^ Bioenergetics and Molecular Biotechnologies (IBIOM) CNR Institute of Biomembranes Bari Italy

**Keywords:** evolution, exon, exon shuffling, intron, intron position, mitochondrial carrier, mitochondrial transporter, recombination, SLC25, solute carrier family 25

## Abstract

Mitochondrial carriers (MCs), which constitute a superfamily also called the solute carrier family 25 (SLC25), are characterized by conserved signature motif sequences and a six‐transmembrane α‐helical transporter domain. They transport a wide variety of substrates ranging from protons, inorganic ions, citric acid cycle intermediates, and amino acids to nucleotides and cofactors. The superfamily members can be divided into subfamilies, each with a distinct substrate specificity. In an attempt to understand how different subfamilies have evolved, we analyzed the protein sequences of the exons (with conserved boundaries) and the six transmembrane α‐helices of MCs from highly diverged organisms. The results show that some MC subfamilies have all exons and transmembrane α‐helices most similar to a closely related subfamily, which is consistent with a scenario of gene duplication and mutational divergence from a last common ancestor. However, several MC subfamilies appear to be mosaics of exons and transmembrane α‐helices most similar to different and distant subfamilies, which in some cases could be explained by recombination between the superfamily genes during evolution. It seems that this latter mechanism could have played a role in the formation of new subfamilies with different substrate specificities by the combination of MC transporter domain segments that had been optimized previously for binding specific portions of the substrates. This study presents novel evolutionary relationships between MC subfamilies and may provide clues for how protein superfamilies have expanded and how to investigate their evolution.

## 1 INTRODUCTION

The mitochondrial carrier (MC) superfamily (in vertebrates named the solute carrier 25 family, SLC25) comprises transporters which are mainly localized in the inner membrane of mitochondria (Palmieri, 2004, 2013; Palmieri et al., 2011). All MC protein sequences exhibit a characteristic three‐fold repeat of about 100 residues, each containing two transmembrane α‐helices connected by a signature motif sequence (PX[DE]XX[KR]X[KR]X_20‐30_[DE]GXXXX[WYF][KR]G; PROSITE PS50920, PFAM PF00153 and IPR018108) (Palmieri, 1994; Saraste & Walker, 1982). They have a common membrane topology consisting of six transmembrane α‐helices (H1‐H6) in a transporter domain with the N‐ and C‐termini in the intermembrane space (Bisaccia et al., 1994; Capobianco et al., 1991; Capobianco et al., 1995; Palmieri et al., 1993). The transmembrane α‐helices are connected by loops, which in the mitochondrial matrix contain a short α‐helix. The 3D‐structures of the MC transporter domain display H1‐H6 in a three‐fold symmetric bundle around a substrate translocation pore with a centrally located substrate binding site, to which the access is regulated by the alternating opening/closing of an m‐gate and a c‐gate on the mitochondrial matrix and cytoplasmic sides of the membrane, respectively (Jones et al., 2023; Kang & Chen, 2023; Pebay‐Peyroula et al., 2003; Ruprecht et al., 2014; Ruprecht et al., 2019). A common transport mechanism for MCs has been proposed based on the original “single‐binding center gated pore” hypothesis (Klingenberg, 1979), the available 3D‐structures, their monomeric state (Bamber et al., 2007) and the transport properties, which suggest that they are antiporters operating with ping‐pong kinetics (Cimadamore‐Werthein et al., 2023; Cimadamore‐Werthein et al., 2024; Indiveri et al., 1994; Palmieri & Pierri, 2010; Ruprecht & Kunji, 2021).

MCs have similarities in sequence, structure, and transport mechanism, although they transport a wide range of different substrates, from protons, phosphate, sulfate, carboxylated metabolites (e.g., citrate, malate, and 2‐oxoglutarate), and amino acids to large nucleotides and cofactors, such as S‐adenosylmethionine, NAD^+^, and FAD (Palmieri, 2021). The importance of MCs in metabolism and physiology is underlined by the numerous human diseases caused by mutations in their genes (Palmieri et al., 2020). Most of the substrates have been identified by the EPRA approach: recombinant over‐expression, purification, and reconstitution of MCs into liposomes that were used in transport assays (Palmieri & Monné, 2016). Based on the biochemical characterization, MCs can be divided into subfamilies that transport the same or very similar substrates (Palmieri, 2021; Palmieri & Pierri, 2010). Furthermore, by analyzing MC subfamily sequences and structures, residues in three specific positions on H2, H4, and H6 (contact points I, II, and III, respectively) between the two gates in the substrate translocation pore have been proposed to participate in substrate binding (Robinson & Kunji, 2006). In fact, the presence of G[IVLM], R[QHNT] or R[DE] in contact point II on H4 correlates with MCs transporting three major classes of substrates: nucleotides, carboxylated metabolites and amino acids, respectively. Residues in the other contact points and in their vicinity are thought to provide the more precise substrate specificity (Marobbio et al., 2008; Monné et al., 2012; Palmieri et al., 2011; Robinson & Kunji, 2006).

The evolution of MCs is thought to have involved an initial triplication of a gene encoding a single 100‐residue repeat (Fiermonte et al., 1999; Kuan & Saier, 1993; Monné et al., 2023; Palmieri, 1994; Pierri et al., 2014; Saraste & Walker, 1982). Gene duplications and mutations of the resulting about 300‐residue ancestral MC into multiple variants with diversified substrate specificity initiated the formation of MC subfamilies. All genes of MCs are found on nuclear chromosomes in eukaryotes, with some exceptions in the genomes of a virus and a few pathogenic prokaryotes (Dolezal et al., 2012; Monné et al., 2007). Presumably, some subfamilies arose already during eukaryogenesis in response to transport requirements of primitive mitochondria and certainly many of them before the split between the animal, fungi and plant kingdoms, because clear orthologs exist among the 53 MCs of *Homo sapiens*, the 35 of *Saccharomyces cerevisiae* and the 58 of *Arabidopsis thaliana* (Palmieri et al., 2011; Palmieri & Pierri, 2010). Therefore, it appears that this superfamily has undergone an expansion to evolve new subfamilies for the transport of new substrates. Previously, single nucleotide evolution as well as conserved, structurally and functionally important residues in MC subfamilies have been analyzed (Palmieri et al., 2011; Pierri et al., 2014; Robinson et al., 2008; Robinson & Kunji, 2006). Recently, we have reported that many intron positions (IPs) found in human MCs are conserved in orthologs of the same subfamily or groups of subfamilies with similar substrates, but that some are also found in phylogenetically distant subfamilies, which could have originated from recombination or exon shuffling of gene segments containing these particular IPs (Monné et al., 2023). The present investigation has proceeded in this direction with the main aim to find evidence for gene duplication versus recombination events among MC subfamilies by analyzing similarities in exon and transmembrane α‐helix sequences, that is, the genetic and structural/functional units from which the genes and proteins are built. The results show that (i) many MC subfamilies consist only of segments most similar to the closest related subfamilies, whereas (ii) other subfamilies have segments most similar to sequences from distantly related subfamilies. The former findings are indicative of gene duplications, while the latter findings, which could not have been disclosed by normal phylogenetic analysis, suggest that recombination between MC subfamily ancestors has contributed to the formation of new MC subfamilies with different substrate specificities. The tentative approach explored here to understand the evolution of the MC superfamily may be developed further and applied to other protein superfamilies in the future.

## 2 RESULTS

**FIGURE 1:**
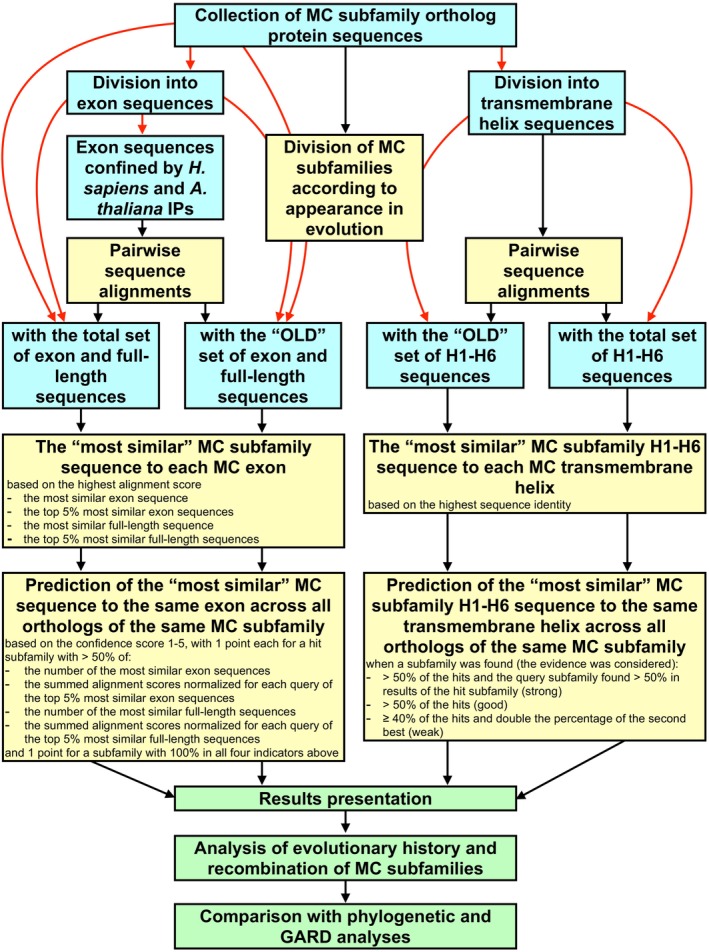
Flowchart of the procedure of the MC exon and transmembrane α‐helix similarity analysis. The directions of the analysis and assembly of sequence sets are indicated by black and red arrows, respectively. Sequence collections, data analysis, and results interpretation are shown in boxes colored in cyan, yellow, and green, respectively.

We investigated the evolutionary history of the MC subfamilies and new isoforms by analyzing protein sequence similarities in their exon and transmembrane α‐helix segments. How the sequence analysis was performed is described in Section 2.1, and how the results were interpreted and evaluated in Section 2.2. The whole procedure is illustrated in a flowchart (Figure 1).

### 2.1 Analysis of sequence similarities in the MC exon and transmembrane α‐helix segments

#### 2.1.1 MC subfamily division based on phylogenetic analysis and biochemically determined substrate specificities

MC protein sequences were collected from the genomes of *H. sapiens*, *S. cerevisiae*, and *A. thaliana*, representing the three kingdoms of animals, fungi, and plants, and divided into subfamilies based on their location in the phylogenetic tree and biochemically determined substrate specificities (Figure S1) (Palmieri, 2021). By using this approach, 51 MC subfamilies were identified in the three species and named as commonly found in the literature (Palmieri, 2021). The phylogenetic tree clearly grouped most subfamilies into five main clusters of MCs transporting nucleotides (MC‐NT1 and MC‐NT2, also including nucleotide‐analog cofactors), carboxylates (MC‐CA, many of them also transport aspartate, phosphate, and sulfate), positively (MC‐AAP, enclosing carnitine) and negatively (MC‐AAN) charged amino acids. Some MC subfamilies, however, were found outside the five main clusters.

#### 2.1.2 MC subfamily ortholog collection and appearance in the evolution

Orthologs of the MC subfamilies (as defined in Figure S1) were collected from a wide range of well‐annotated genomes in highly diverged species representing different groups of organisms from the animal, fungi, and plant kingdoms (see Section 4 for details). The final MC sequence collection contained 768 proteins (Table S1). The presence of the MC subfamilies and new isoforms in species of the three kingdoms and mammals was used to estimate their appearance during the evolution that was mapped onto a simplified tree (Figure S2). At the origin of the tree, the “original” MC subfamilies with clear orthologs in both the animal/fungi and plant branches are indicated. The subsequent appearance of new MC subfamilies and isoforms specifically found in plants, fungi, animals, or mammals was added to the tree, as well as the disappearance of some subfamilies in some lineages.

#### 2.1.3 Sequence similarity analysis of the MC subfamily exons

The transporter domains of the collection of MC sequences described in the previous section were divided into subfamilies, aligned with the IPs mapped (see Section 4 for details), which in part had already been accomplished previously (Monné et al., 2023), and the exon sequences (ESs) were extracted. As noted before, the IP pattern was different between subfamilies, but within each subfamily, the IPs of the human sequences were totally conserved in vertebrates, partially in invertebrates, and only exceptionally in fungi (Monné et al., 2023). The IPs of plant sequences were different from the human IPs but also conserved within each subfamily. Therefore, we chose to analyze sequence similarities of the MC subfamily ESs with conserved boundaries confined by IPs identical to *H. sapiens* from animals and fungi, and separately ESs with IPs identical to *A. thaliana* from plants.

The ESs confined by *H. sapiens* and *A. thaliana* IPs were used in pairwise sequence alignments against the total set of 768 MC full‐length and 4021 exon sequences. The “most similar” exon and full‐length sequences of a query ES were considered those of another MC subfamily than the query with the highest alignment score and a sequence identity (SI) of at least 30%. The same exon (with identical boundaries) across all orthologs of the same subfamily was analyzed: if the absolute majority of the top sequence hits matched a single MC subfamily, a “most similar” subfamily prediction was assigned to the exon and given a confidence score on the scale from one to five, with five being the highest (see Section 4 for details) (Table S2). In this report, the generic term “most similar” sequence to an exon is used, referring to the above definition. By using the majority approach, the sequence variations in exons among subfamily orthologs, paralogs, and extremely diverged isoforms were taken into account (both for queries and hits) because of the inclusion of sequences from highly diverged species. In fact, for many exons, the most similar sequence prediction could not be determined, that is, no absolute majority of a MC subfamily among the hits was found, which was probably due to high sequence divergence. Therefore, the predictions identify the closest homologous MC subfamily sequences to each MC subfamily exon.

In an attempt to investigate the direction of evolution, the animal/fungi and plant ESs were also used in pairwise sequence alignments only against sequences of subfamilies estimated to have been formed before the query subfamily based on their appearance in the simplified tree (Figure S2). These exon and full‐length sequences, depending on the appearance of the query MC subfamily, were selected in separate sequence sets called “OLD”. In the case of the analysis of the ESs of the “original” MC subfamilies, the “OLD” sequence set included all of the original ones. Therefore, the results of this analysis suggest the potential evolutionary origin of sequence segments of the more recently formed subfamilies and isoforms examined. The results obtained by using both the “OLD” and the total set of sequences are found in Table S2.

#### 2.1.4 Sequence similarity analysis of the MC subfamily transmembrane α‐helices

The collected full‐length MC sequences were aligned, and the transmembrane α‐helices could be identified easily due to the signature motifs. The central transmembrane α‐helix sequences, comprising the residues that protrude into the substrate binding site both when the m‐ and c‐gates are closed, were extracted from the alignment. The resulting H1‐H6 set included 4531 sequences, excluding transmembrane α‐helices that could not be clearly or only partially identified in the sequences.

Each central transmembrane α‐helix sequence was used against the H1‐H6 collection in pairwise sequence alignments without gaps. The hit sequence with the highest SI and belonging to another MC subfamily than the query was considered the most similar. By analyzing the same transmembrane α‐helix of all the members of the same subfamily, the percentage of the sequence hits from one and the same MC subfamily could be calculated. The evidence for the “most similar” MC subfamily H1‐H6 was considered: strong, when a subfamily had more than 50% of the hits and the query subfamily was reciprocally found in more than 50% in the results of the hit subfamily; good, when a subfamily had more than 50% of the hits; or weak, when a subfamily had at least 40% of the hits and double the percentage of the second best (Table S3). Whereas the parameters used in the analysis of exon similarities may be considered too permissive, especially for short exon segments and highly variable loop regions, those of the analysis of the transmembrane α‐helices were very stringent and most of considered hits exhibited very high SI. At variance with the exon similarity analysis, in the H1‐H6 similarity analysis subfamily members from both animal/fungi and plant were analyzed together. Therefore, the predictions identify the closest MC subfamily H1‐H6 sequence to each MC subfamily transmembrane α‐helix that is likely to reflect the conservation of the segments that are linked to substrate binding.

As in the analysis of the ESs, the H1‐H6 sequences were also used in pairwise sequence alignments against “OLD” sets (of H1‐H6) sequences from subfamilies that had appeared prior to the query subfamily in the simplified tree (Figure S2), and the “OLD” set of H1‐H6 sequences for the “original” MC subfamilies included all the ones at the origin. The results of the analysis of the H1‐H6 similarities in the “OLD” and total set of sequences are both reported in Table S3.

### 2.2 Evolutionary history of the MC subfamilies based on similarity analysis of the exon and transmembrane α‐helix segments

**FIGURE 2:**
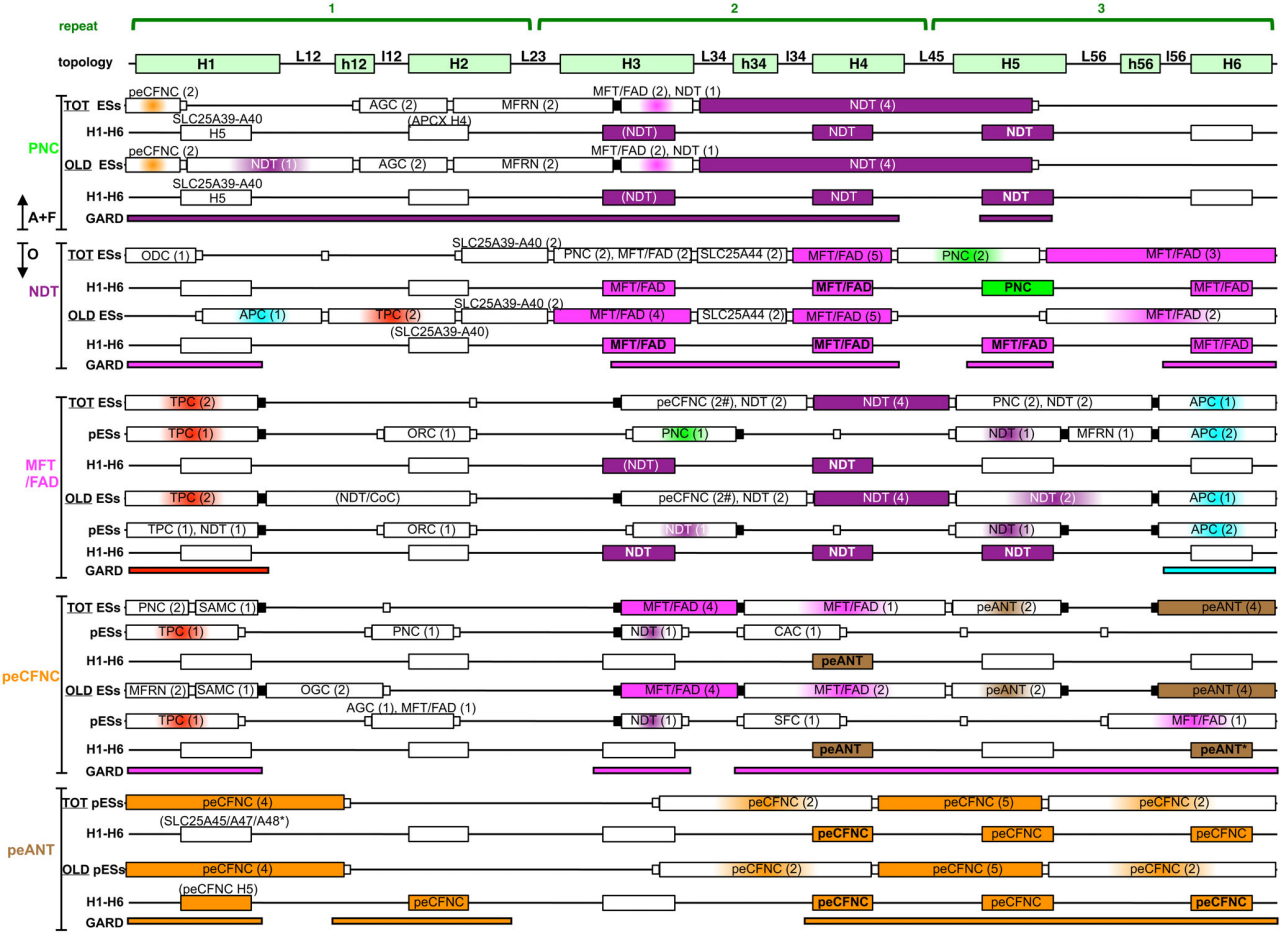
The most similar sequences to the MC‐NT2 cluster subfamily exons and transmembrane α‐helices. The MC topology is displayed at the top with the following segments: The transmembrane α‐helices H1‐H6; the matrix loops L12, l12, L34, l34, L56 and l56; the intermembrane space loops L23, L45; and the matrix α‐helices h12, h34 and h56. The MC‐NT2 cluster subfamilies are shown on the left with specific colors and divided into those that appeared in animals and fungi (A + F) and at the origin (O) (according to Figure S2). The horizontal black lines represent the MC subfamily sequences (aligned to the MC topology), upon which the results of the most similar sequences in the total (TOT) and OLD sequence sets to the exon and H1‐H6 sequences are displayed (extracted from Tables S2 and S3). The results for the plant exons are indicated as pESs. The ES results are shown with the conserved subfamily IPs as small vertical rectangles, of which those occurring in more than one subfamily of this cluster with the same codon frame position are filled with black. The ES results are displayed with the confidence score in parentheses in the horizontal rectangles (indicating the exons) with the most similar MC subfamily color: Gradient and full coloring for the weaker (maximum 2) and stronger (minimum 3) scores, respectively. Single‐hit sequences from only one species are indicated with #. The H1‐H6 results are shown in the H1‐H6 vertical rectangles with the most similar MC subfamily color and indicated in bold, regular, and parentheses for strong, good, and weak predictions, respectively. The H1‐H6 similarity results are always from the same corresponding helix or otherwise indicated. The H1‐H6 results, mainly depending on hit sequences, are indicated with *. The GARD results are displayed with the predicted recombined fragments (confined by breakpoints) of one MC subfamily as thin horizontal rectangles with the color of the other MC subfamily. Segments similar to the MC‐NT1 cluster TPC and APC subfamilies are indicated in red and cyan, respectively.

**FIGURE 3:**
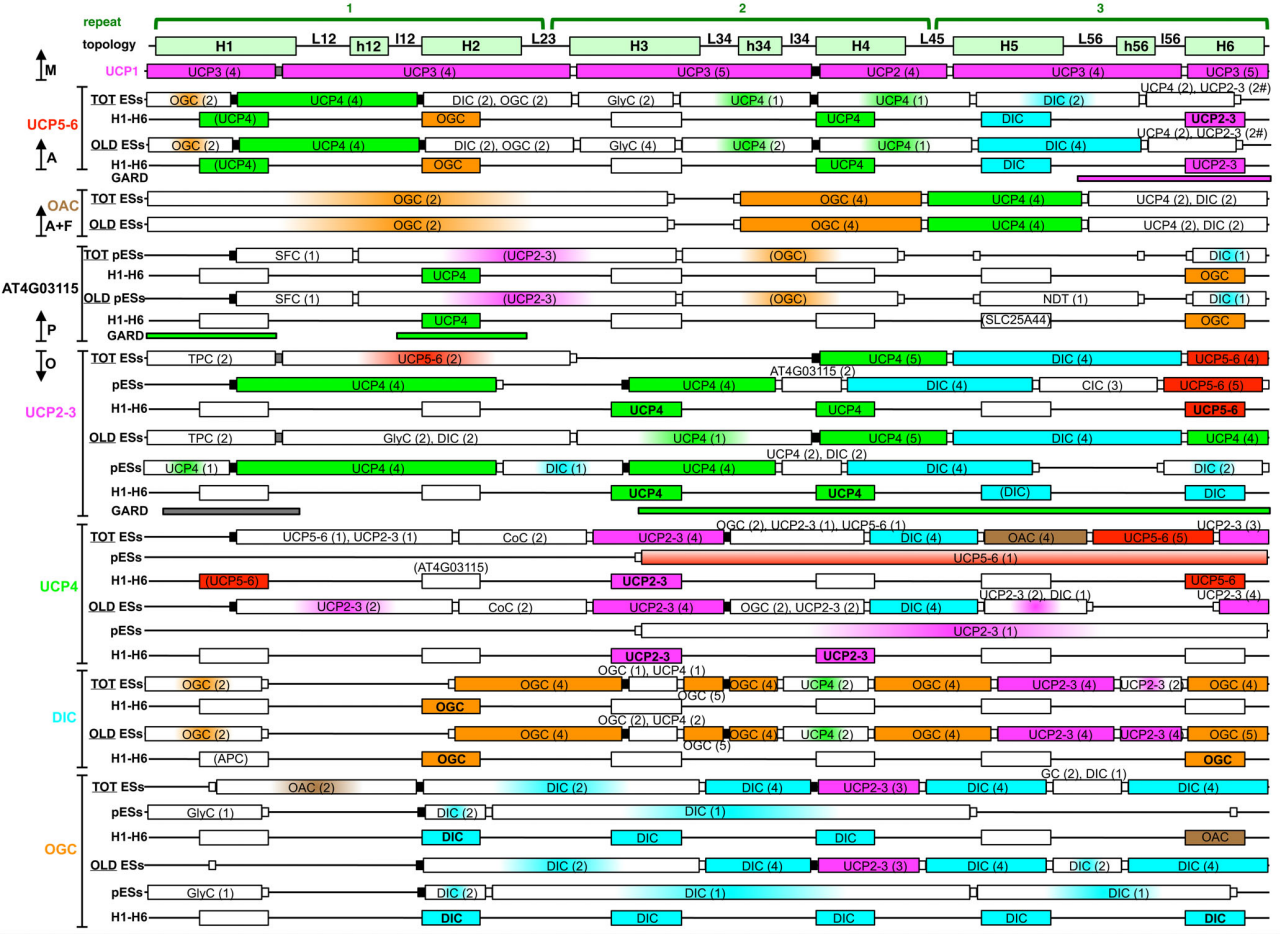
The most similar sequences to the MC‐CA cluster subfamily exons and transmembrane α‐helices. The MC topology, MC subfamily sequences and IPs, as well as the results of GARD analysis, the most similar sequences in the total (TOT) and OLD sequence sets of the ESs, plant ESs (pESs) and H1‐H6 (extracted from Tables S2 and S3) are all displayed as in Figure 2. The MC‐CA cluster subfamilies are shown on the left with specific colors and divided into those that appeared in mammals (M), animals (A), animals and fungi (A + F), plants (P), and at the origin (O) (according to Figure S2). The OAC lacked H1‐H6 results to display (Table S3). The fragment similar to the MC‐NT1 cluster TPC subfamily is indicated in gray.

**FIGURE 4:**
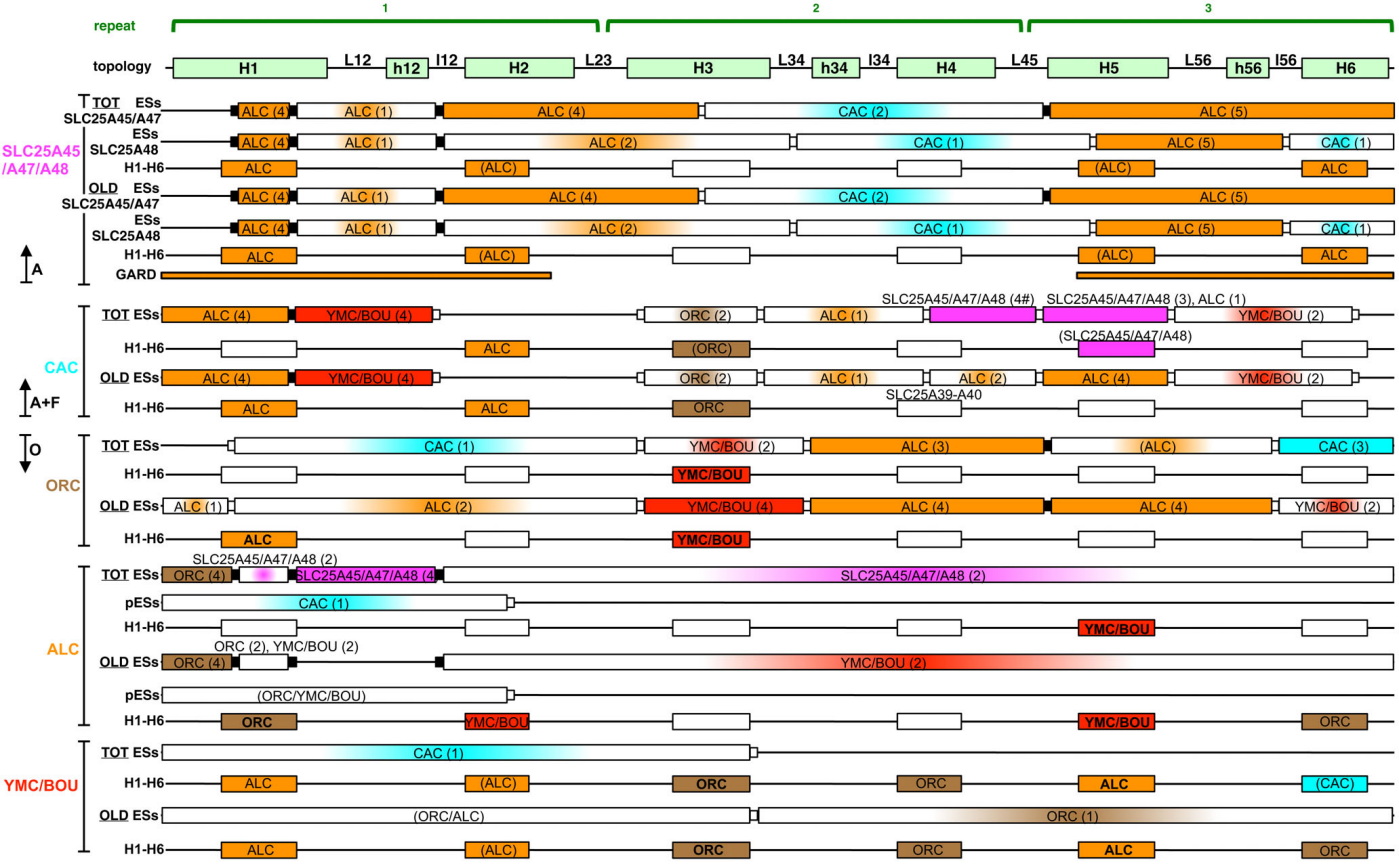
The most similar sequences to the MC‐AAP cluster subfamily exons and transmembrane α‐helices. The MC topology, MC subfamily sequences and IPs, as well as the results of GARD analysis, the most similar sequences in the total (TOT) and OLD sequence sets of the ESs, plant ESs (pESs) and H1‐H6 (extracted from Tables S2 and S3) are all displayed as in Figure 2. The MC‐AAP cluster subfamilies are shown on the left with specific colors and divided into those that appeared in animals (A), animals and fungi (A + F), and at the origin (O) (according to Figure S2).

**FIGURE 5:**
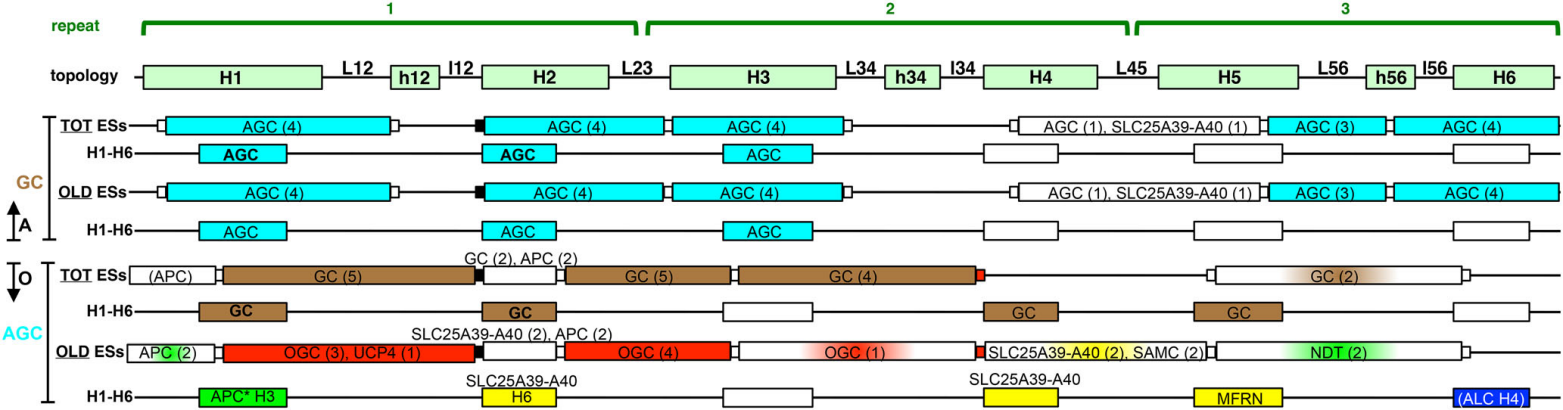
The most similar sequences to the MC‐AAN cluster subfamily exons and transmembrane α‐helices. The MC topology, MC subfamily sequences, and IPs, as well as the results of GARD analysis, the most similar sequences in the total (TOT) and OLD sequence sets of the ESs and H1‐H6 (extracted from Tables S2 and S3) are all displayed as in Figure 2. The MC‐AAN cluster subfamilies are shown on the left with specific colors and divided into those that appeared in animals (A) and at the origin (O) (according to Figure S2). Subfamily IPs commonly found in the MC‐CA cluster are shown in red. The segments similar to MCs of the MC‐NT1‐2, MC‐CA, MC‐AAP clusters and outside the main clusters are shown in green, red, blue, and yellow, respectively.

**FIGURE 6:**
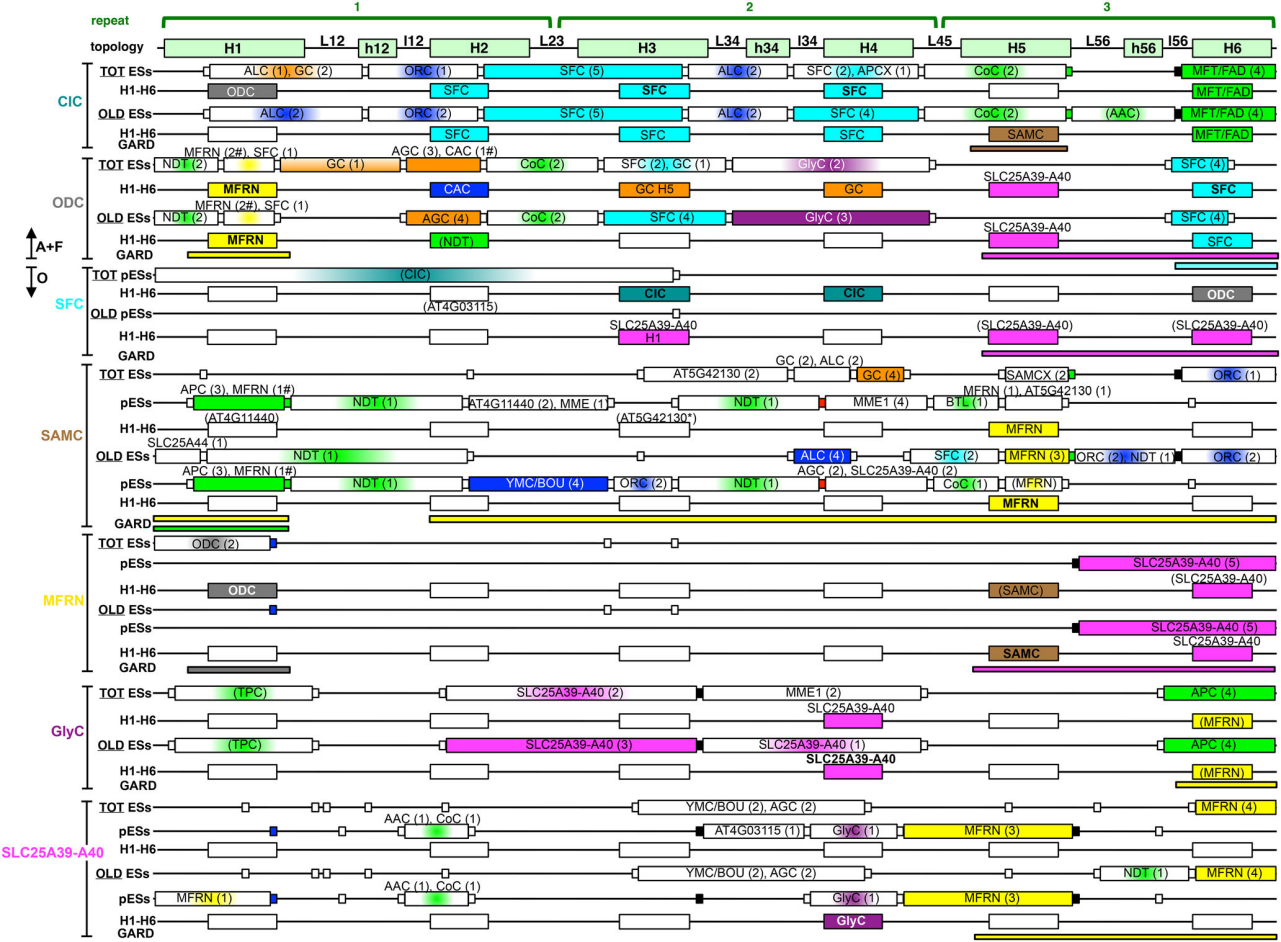
The most similar sequences to the exons and transmembrane α‐helices of MC subfamilies outside the main clusters. The MC topology and MC subfamily sequences, as well as the results of GARD analysis, the most similar sequences in the total (TOT) and OLD sequence sets of the ESs, plant ESs (pESs) and H1‐H6 (extracted from Tables S2 and S3) are all displayed as in Figure 2. The MC subfamilies are shown on the left with specific colors and divided into those that appeared in animals and fungi (A + F) and at the origin (O) (according to Figure S2). The other MC subfamilies outside the main cluster are shown in Figure S5. The ES results are shown with the conserved subfamily IPs as small vertical rectangles, of which those occurring in more than one subfamily with the same codon frame position are filled with black or blue, and those commonly found in the MC‐NT1‐2, MC‐CA and MC‐AAP clusters are colored green, red and blue, respectively. The segments similar to MCs of the MC‐NT1‐2, MC‐AAP, and MC‐AAN clusters are shown in green, blue, and orange, respectively.

To make a comprehensive evolutionary analysis, the similarity results of the MC segments (Tables S2 and S3) were transferred onto schematic figures (divided based on the phylogenetic clusters, i.e., MC‐NT2, MC‐NT1, MC‐CA, MC‐AAP, MC‐AAN and MC subfamilies outside the five clusters), which also include the IPs (Figures 2, 3, 4, 5, 6 and S3–S5). Most interestingly, the results show that MC subfamilies are mosaics of segments most similar to different subfamilies, which allow to “read” their evolutionary history. The interpretation of an MC subfamily exon or transmembrane α‐helix sequence being most similar to another MC subfamily is that the two subfamily segments have a last common ancestor. When an MC subfamily consisted of segments similar to subfamilies in the same lineage, which could reflect that some parts are closer to an ancestor (the OLD dataset results) and some closer to an offspring subfamily (sometimes in the total dataset results), the interpretation is a duplication of the whole common ancestral gene. However, when the similar subfamilies were distant or not on the same line of descent, this finding is taken as evidence for donor or recipient events from different ancestral genes through recombination or exon shuffling. In several cases, the evolutionary relationships between similar MC subfamily segments were further supported by the presence of identical IPs.

In the following sections, the results for the MC subfamilies of the different clusters are examined briefly and compared to the phylogenetic analysis. The major focus is on the results for segments with stronger evidence for the similarities, such as the stringent H1‐H6 results, ES predictions with higher confidence scores, identical IPs, and where several (and adjacent) MC segments are similar to the same subfamily. Moreover, the Genetic Algorithm for Recombination Detection (GARD) was applied to the MC subfamilies shown to share most similar segments with the exon and transmembrane α‐helix similarity analyses to give further support for potential recombination events (also included in Figures 2, 3, 4, 5, 6 and S3–S5) (Kosakovsky Pond et al., 2006; Weaver et al., 2018).

#### 2.2.1 Evolutionary history of the MC‐NT2 cluster subfamilies

The results of the segment similarity analysis for the MC‐NT2 cluster subfamilies give quite a clear overall picture of how the majority of the subfamily sequences of this cluster evolved through gene duplications (Figure 2). The mitochondrial folate or FAD transporter subfamily (MFT/FAD) contains segments most similar to MC‐NT1 cluster subfamilies (thiamine pyrophosphate carrier (TPC) and ATP‐Mg/phosphate carrier (APC)), suggesting that MFT/FAD could have a common ancestor with the MC‐NT1 cluster and then branched off into the whole MC‐NT2 cluster. The NAD^+^ transporter (NDT) and peroxisomal Coenzyme A, FAD, and NAD^+^ carrier (peCFNC) include segments most similar to MFT/FAD, and vice versa, which indicate that the two former subfamilies could have been derived from the latter. Furthermore, MFT/FAD and peCFNC contain five identical IPs, some of them confining exons that are similar between these two subfamilies, which are observations that further corroborate their close common origin. Subsequently, based on the segment similarities, the pyrimidine carrier (PNC) and peroxisomal adenine nucleotide translocator (peANT) could have been formed from NDT and peCFNC, respectively. Thus, these results for the MC‐NT2 cluster subfamilies (Figure 2) give specific details beyond the phylogenetic analysis (Figure S1). Moreover, it appears that the MFT/FAD, NDT, PNC, and peCFNC subfamilies have the H3‐H5 regions particularly similar to each other, whereas peANT has nearly all exons and transmembrane α‐helices (except H3) most similar to peCFNC (Figure 2). The non‐similar regions between the MC‐NT2 subfamilies could have evolved through mutations, or alternatively, as our analysis suggests, by recombination. Potential recombination of the regions around H3 of peANT, H6 of PNC, and H2 of MFT/FAD, NDT, and peCFNC is further supported by the GARD analysis. It is likely that the similar parts among the MC‐NT2 cluster subfamilies bind identical portions of the structurally related substrates, whereas the diverged parts are responsible for the differences in substrate specificities (see table in Figure S1).

#### 2.2.2 Evolutionary history of the MC‐NT1 cluster subfamilies

TPC contains several ESs most similar to MFT/FAD of the MC‐NT2 cluster (Figure S3), again suggesting that a common ancestor of these subfamilies links the two clusters (as proposed in the previous section). There are also many identical IPs found among both the MC‐NT2 and MC‐NT1 subfamilies (in black in Figure S3) that support this idea. The subsequent order of appearance of the original MC‐NT1 cluster subfamilies is difficult to predict, but from the similar segments, it seems that the Coenzyme A carrier (CoC) could have branched off from TPC, and then the other subfamilies from CoC. Especially CoC and YPR011C have many transmembrane α‐helices and ESs most similar to each other and share four IPs, illustrating that they are very closely related. Furthermore, the recently occurring MC‐NT1 isoforms of the ADP/ATP carrier (AAC) (mammalian AAC4 and the plant AACs located in the ER and plasma membrane ER‐ANT and PM‐ANT) and APC (mammalian APC4) have all ESs most similar to their subfamily and share several of their IPs (Figure S4). Therefore, the results of our analysis suggest that these new isoforms have arisen through gene duplications of another subfamily member, as expected, and may be considered as positive controls of the exon similarity analysis method presented here. The conclusions of the segment similarity analysis of the MC‐NT1 cluster subfamilies are largely in agreement with the phylogenetic tree (Figure S1) and have presumably evolved through gene duplications, as no strong indication of recombination was detected.

#### 2.2.3 Evolutionary history of the MC‐CA cluster subfamilies

The segment similarity analysis suggests additional and alternative information for the MC‐CA cluster subfamilies with respect to those deduced from the phylogenetic tree (Figure S1). The 2‐oxoglutarate carrier subfamily (OGC) contains sequence segments almost exclusively most similar to the dicarboxylate carrier (DIC), and both the uncoupling protein (UCP) subfamilies UCP2‐3 and UCP4 contain some ESs most similar to DIC, suggesting that a DIC member was the ancestor of these MC‐CA cluster subfamilies (Figure 3). Therefore, OGC as well as the common UCP2‐3 and UCP4 ancestor, which then deviated into these two subfamilies, appear to be derived from a gene duplication of DIC. An interesting finding is that the N‐terminal exon of animal UCP2‐3 is most similar to TPC of the MC‐NT1 cluster (and vice versa, Figures 3 and S3) and it ends with an IP (colored gray in Figure 3) identical to that found in TPC and in many MC‐NT1‐2 cluster subfamilies but in no other MC‐CA subfamily. At variance, the N‐terminal exon of the plant UPC2‐3 version (pUCP2‐3) is defined by a different IP that is conserved among MC‐CA subfamilies. One explanation, which is substantiated by the GARD analysis (gray fragment), is that the N‐terminal exon of TPC was transferred to animal UCP2‐3 by recombination after the deviation from plants. Moreover, the mammalian‐specific isoform UCP1 has identical IPs, and all the ESs are most similar to UCP2‐3, which clearly demonstrates that it belongs to this subfamily. In addition, UCP5‐6, AT4G03115, and the oxaloacetate carrier (OAC) contain a mix of similar segments and identical IPs to original MC‐CA cluster subfamilies (Figure 3), which might reflect that they deviated earlier through gene duplications (as suggested for AT4G03115 and OAC in Figure S1) or appeared later (as in Figure S2) through recombination of segments. The GARD analysis is in agreement with the latter hypothesis for some segments in AT4G03115 and UCP5‐6 (Figure 3).

#### 2.2.4 Evolutionary history of the MC‐AAP cluster subfamilies

In the MC‐AAP cluster, the patterns of similar segments indicate that: the subfamilies of the ornithine carrier (ORC), arginine‐lysine carrier (ALC) and YMC/BOU might have arisen from a common ancestor; the carnitine carrier (CAC) from ALC and ORC; and the SLC25A45/A47/A48 subfamily mainly from ALC (Figure 4). However, it should be noted that, although SLC25A45/A47/A48 has almost all ESs, H1‐H2, and H3‐H4 most similar to ALC, and both subfamilies also have the first three IPs in common, a region including H4 (and partly H3) appears different (Figure 4). In particular, the ES analysis shows that the H4 region of the SLC25A45/A47/A48 subfamily is more similar to CAC than ALC. We would like to hypothesize, therefore, that the ancestor of SLC25/A47/A48 evolved from a gene duplication of ALC with an insertion of the H3‐H4 region from CAC. In fact, the GARD analysis strongly supports the conclusion that the segments around H1‐H2 and H5‐H6 are likely to have come from ALC and that a recombination event in the H3‐H4 region has occurred.

#### 2.2.5 Evolutionary history of the MC‐AAN cluster subfamilies

Almost all ESs and transmembrane α‐helices of the glutamate carrier subfamily (GC) are most similar to the aspartate/glutamate carrier (AGC), and vice versa in the AGC results of total sequence sets (Figure 5), which suggests that GC could have been formed by a gene duplication of the previously appeared AGC. Furthermore, it is clear from the results of the “OLD” sequence sets that the N‐terminal half of AGC contains three ESs most similar to MC‐CA cluster subfamilies (mainly OGC) as well as two IPs found specifically in MC‐CA members. In contrast, H4 of AGC is most similar to the glutathione carriers SLC25A39‐A40. These results suggest that three ESs of the AGC N‐terminal half and the AGC H4 could have come by recombination from the MC‐CA cluster and SLC25A39‐A40, respectively, both transporting substrates containing structural similarities to those of AGC. Notably, there is no evidence, either in the results presented here or in the phylogenetic analysis, for an evolutionary link between the MC‐AAN cluster and the amino acid‐transporting MCs of the MC‐AAP cluster.

#### 2.2.6 Evolutionary history of the MC subfamilies outside the main clusters

For the subfamilies of the oxodicarboxylate carrier (ODC), citrate carrier (CIC), and succinate/fumarate carrier (SFC), the segment similarity approach suggests evolutionary relationships with a series of potential recombination events. By comparing the results of the OLD sequence sets for CIC and ODC with those of the total sequence set of SFC, it seems that the regions around H2‐H4 of CIC as well as those around H3 and H6 of ODC could have come from SFC (Figure 6). On the other hand, H5 of CIC and H1 of ODC may have been derived by recombination from the S‐adenosylmethionine carrier (SAMC) and mitoferrin (MFRN) subfamilies, respectively. Furthermore, for SFC, the results show that the H5‐H6 region may have been derived from SLC25A39‐A40. Nearly all these results are supported by the GARD analysis. It is noteworthy that none of the three subfamilies ODC, CIC, and SFC have any segments similar to the MC‐CA cluster subfamilies that have the same contact point II residues and some identical substrates, which means that they have evolved independently, as also suggested by the phylogenetic tree (Figure S1).

There are also similarities between the glutathione carrier SLC25A39‐A40, glycine carrier (GlyC), MFRN and SAMC subfamilies (Figure 6). The segment analysis indicates that (i) the regions of SLC25A39‐A40 and GlyC around H3‐H4, including an identical IP, are most similar to each other; (ii) segments of SLC25A39‐A40 in the H5‐H6 area, including an identical IP, are most similar to MFRN; (iii) H5 of MFRN is reciprocally most similar to SAMC; and (iv) SAMC has many exons most similar to MC‐NT1‐2 and MC‐AAP cluster subfamilies (Figure 6). On the basis of these results, it might be hypothesized evolutionary paths which include gene duplications (SAMC arose from an ancestor in common with the MC‐NT1‐2 and MC‐AAP clusters; MFRN deviated from SAMC; SLC25A39‐A40 from MFRN (a relationship between them is corroborated by two identical IPs); and GlyC from SLC25A39‐A40) and recombinations (SAMC could have been assembled through recombination of MC‐NT1‐2 and MC‐AAP cluster members; H5‐H6 of MFRN could have been recombined into SLC25A39‐A40, which is also supported by GARD; and H3‐H4 of SLC25A39‐A40 into GlyC).

For the remaining MC subfamilies outside the main clusters, the segment similarity analysis gives some weak evidence for evolutionary relationships (Figure S5) that are not detected or not well supported by the phylogenetic analysis (Figure S1). In fact, there are some most similar segments indicating that MTCH and SLC25A46 could have originated from the MC‐NT1‐2 clusters; AT4G11440 and the S‐adenosylmethionine‐like carrier (SAMCX) from SAMC and SLC25A39‐A40; and the mitochondrial magnesium exporter (MME) from SAMC and MFRN (Figure S5). In some of these cases, the GARD analysis supports recombination (Figure S5).

## 3 DISCUSSION

**FIGURE 7:**
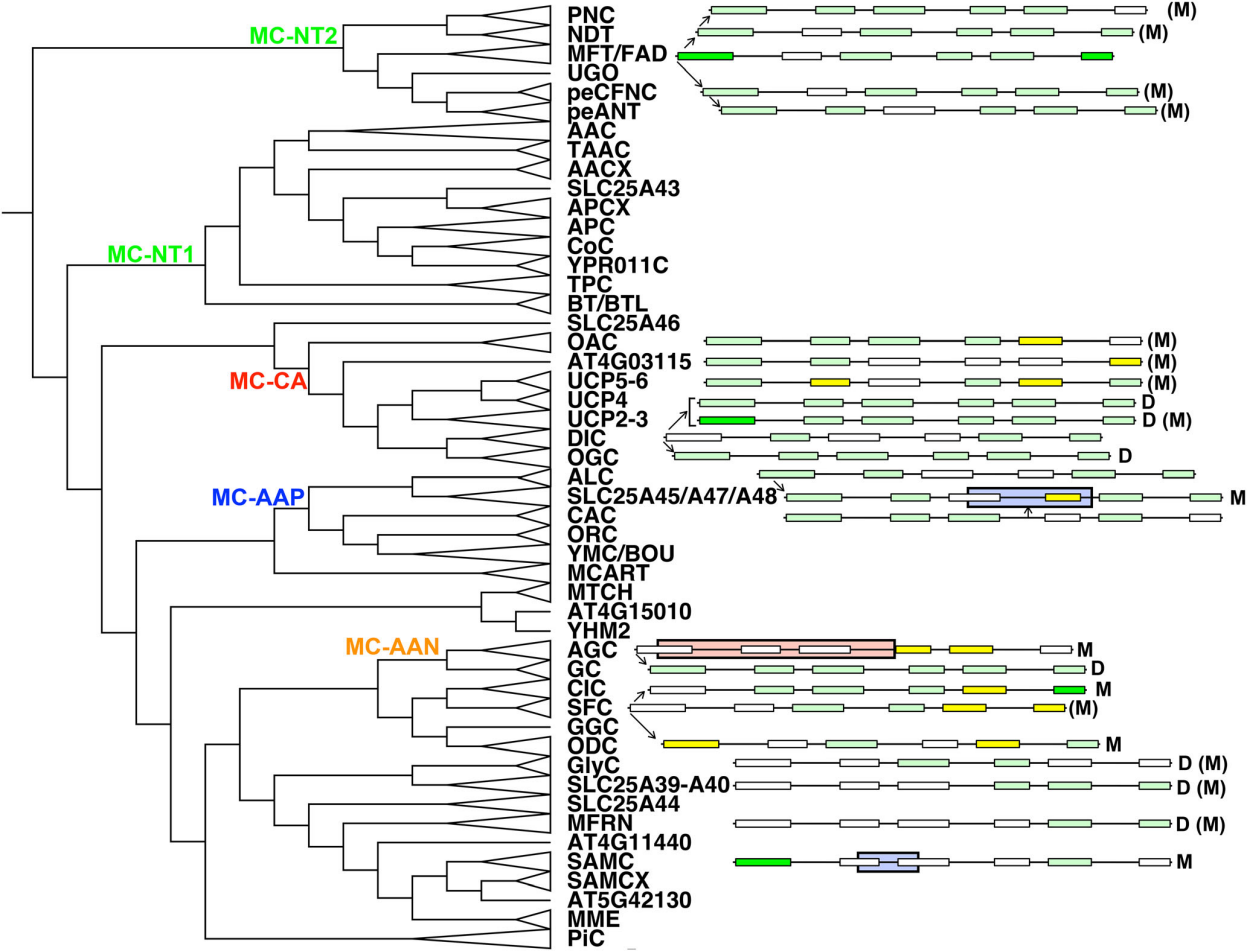
Main conclusions about the evolutionary history of MC subfamilies. Cladogram of the MC subfamilies made from the phylogenetic analysis in Figure S1 with the main clusters indicated (on the left). The topology of the MCs is displayed with the transmembrane α‐helices as rectangles on the full‐length sequences (line) (on the right). Arrows indicate the proposed lineages suggested by the segment similarity analysis. The segments are colored according to their suggested origin: When segment is from the same lineage (light green); a different lineage (yellow); a potentially different but unidentified origin (white); the MC‐NT1 cluster (dark green); the MC‐CA cluster (light red); and MC‐AAP cluster (light blue). The suggested evolutionary history as a gene duplication (D) or mosaic composition (M) is indicated and in parentheses if they are more uncertain or alternative.

In this study, we have applied an innovative approach of analyzing the evolution of exon and transmembrane α‐helix sequences to the MC superfamily. Perhaps the most striking result is that many MC subfamilies of the currently living organisms are mosaics of ESs and transmembrane α‐helices most similar to different MC subfamilies. The findings propose plausible novel evolutionary links between MC subfamilies and identify potential recombined fragments, with the main conclusions illustrated in Figure 7. Interestingly, the study also suggests that (i) many IPs in the MC gene structure, partially investigated in a previous study (Monné et al., 2023), have been transferred together with the surrounding segments and (ii) the specific combinations of MC segments most similar to various other subfamilies are likely to be correlated with similarities and differences in substrate specificity. Although an automated tool for this approach is still lacking, the general guidelines deduced from this study (Figure S6) can be applied to other superfamilies and could be the first step towards new machine learning methods to identify a wider spectrum of inherited sequence segments, explore substrate specificity in the sequence‐structure space, and predict function.

Many of the results of the MC segment similarity analysis are in line with the phylogenetic analysis. Not surprisingly, many new plant‐, animal‐, and mammalian‐specific isoforms and some of the subfamilies have all or almost all of their exons and transmembrane α‐helices most similar to those of a single subfamily (isoforms: UCP1 to UCP2‐3; APC4 to APC; ER‐ANT, PM‐ANT, and AAC4 to AAC; subfamilies: OGC to DIC; and GC to AGC; Figure 7). Therefore, these MCs are likely to have originated by gene duplications and diverged by mutations for the transport of identical or very similar substrates. Furthermore, the similarity analysis can suggest the position of the MC subfamilies in the evolutionary context by the segments most similar to different subfamilies that appeared immediately before or after the subfamily in question in the same lineage (see arrows in Figure 7). Thus, some of the results give additional information with respect to the phylogenetic analysis.

The results, presented here, also show differential conservation among MC fragments and detect potential recombination events, which cannot be revealed by the phylogenetic analysis. Some of the main cluster subfamilies contain exons and transmembrane α‐helices most similar to different subfamilies within the same cluster that may be linked to the substrate specificity. For example, the results of the MC‐NT2 cluster (Figure 7) indicate that the similar parts between the subfamilies, that is, 2–5 transmembrane α‐helices could play a role in binding identical structural entities of the related substrates, such as the ribose‐5′‐diphosphate portion found in all MC‐NT2 cluster substrates. In contrast, the regions with less similarity between the subfamilies of the same cluster and predicted to have been recombined could be partly responsible for the differences in substrate specificities between the MC‐NT2 subfamilies. Another example is the SLC25A45/A47/A48 ancestor that is proposed to have been formed from ALC by a gene duplication and from CAC by recombination of a region including H3‐H4 (Figure 7). Contact point II in H4 is thought to bind the α‐amino and carboxylic groups of the substrate amino acids transported by ALC and other MCs for amino acids as well as the quaternary ammonium group of the CAC substrates (Giangregorio et al., 2010; Indiveri et al., 1998; Monné et al., 2012; Palmieri et al., 1999; Porcelli et al., 2014; Robinson et al., 2008; Robinson & Kunji, 2006). Therefore, the H3‐H4 recombination could have contributed to the substrate specificity of SLC25A45/A47/A48 carriers, which have been shown to transport the quaternary ammonium compounds choline and trimethyl‐lysine (Auger et al., 2026; Dias et al., 2025; Khan et al., 2024; Khan et al., 2025; Patil et al., 2024; Verkerke et al., 2024). In addition, other subfamilies contain exons and transmembrane α‐helices most similar to phylogenetically unrelated subfamilies, and some of these may also be correlated with the substrate specificity. For example, our results suggest that AGC (with the substrates aspartate and glutamate containing the typical amino acid α‐amino and α‐carboxylic groups as well as a carboxylated side chain) could have been formed by H4 from an amino acid‐transporting MCs from outside the main clusters and other fragments from a carboxylate‐transporting MC‐CA cluster member (Figure 7). Another example is SAMC (with the substrates S‐adenosylmethionine and S‐adenosylhomocysteine, that is, chimeras of a nucleotide and amino acids), which is a fusion of fragments similar to nucleotide‐ and amino acid‐transporting MCs of the MC‐NT1‐2 and MC‐AAP clusters, respectively (Figure 7). Thus, our results pointing towards recombination may explain why segments including H4 with a contact point II typical for amino acid transport are found in groups of MCs apparently unrelated to each other in the MC‐AAP, MC‐AAN clusters and outside the main clusters. Therefore, the recombination of segments with residues binding specific parts of the substrates from different MC subfamilies may have contributed to the formation of new subfamilies with new “combined” substrate specificities during evolution.

The findings of this study on the exon and transmembrane α‐helix evolution in the MC superfamily have some general implications. It has already been reported that recombination and exon shuffling can unify different structural domains into single‐polypeptide multi‐domain proteins combining various functions, in the expansion of superfamilies in animals and plants (Bork, 1991; França et al., 2012; Gilbert, 1978; Hynes, 2012; Liu & Grigoriev, 2004; Patthy, 1999; Roy & Gilbert, 2006; Vogel & Chothia, 2006). In contrast, the results presented here indicate that recombinations of “intra‐domain” segments of different MCs within the same transporter domain could have provided new substrate specificities. Some analogous processes to recombination and exon shuffling have been found to mix segments in the same superfamily domain that give rise to proteins with functional differences. For example: somatic recombination for developing ligand‐binding specificities in immunoglobulins and T‐cell receptors (Jones & Gellert, 2004); in vitro exon shuffling and recombination for providing enhanced activity and altered substrate specificity in enzymes (Carbone & Arnold, 2007; Crameri et al., 1998; Trudeau et al., 2013); exon shuffling and alternative splicing for variation in algal immune system proteins (Teng et al., 2023); and alternative splicing in many proteins, for example, the mitochondrial phosphate carrier and among voltage‐gated calcium channels (Dolce et al., 1994; Fiermonte et al., 1998; Lipscombe et al., 2013; Nilsen & Graveley, 2010). Therefore, it is tempting to propose that the expansion and functional diversity of other protein superfamilies might, at least in part, have evolved as envisaged for the MC subfamilies here: by (1) fusion of duplicated DNA fragments encoding segments of the same fold (with/without mutations) from different subfamilies; (2) selection of new combinations resulting in proteins with new functions of adaptive advantage; (3) transfer of these new subfamily founders to subsequent generations; and (4) further optimization by mutagenesis.

## 4 METHODS

### 4.1 MC sequence collection and subfamily division

NCBI Gene was used to collect the protein sequences of the MCs from *H. sapiens*, *A. thaliana*, and *S. cerevisiae* based on their annotated nomenclature. The subfamily division was achieved based on the phylogenetic tree (considering high bootstrap values) and the reported substrate specificity for many MCs (Figure S1). Ortholog protein sequences of the *H. sapiens*, *A. thaliana*, and *S. cerevisiae* MC subfamilies were searched in the NCBI non‐redundant protein sequence database of NCBI annotated genomes with BLASTP (https://blast.ncbi.nlm.nih.gov/ with default parameters: BLOSUM62 matrix; existence and extension gap penalties of 11 and 1, respectively). The ortholog protein sequences were selected based on unambiguous reciprocal best hits with the highest SI and at least 70% sequence coverage of the query sequence, database subfamily annotation, and considering their orthology group. The searched genomes included: (i) animals: vertebrates (mammal (*H. sapiens*), bird (*Gallus gallus*), amphibian (*Xenopus tropicalis*) and fish (*Danio rerio*)), non‐insect invertebrates (nematode (*Caenorhabditis elegans*), mollusk (*Aplysia californica*), tunicata (*Ciona intestinalis*) and echinodermata (*Acanthaster planci*)) and insects (diptera (*Drosophila melanogaster*), hemiptera (*Bemisia tabaci*), lepidoptera (*Spodoptera frugiperda*), hymenoptera (*Acromyrmex echinatior*), polyneoptera (*Cryptotermes secundus*) and coleoptera (*Dendroctonus ponderosae*)); (ii) fungi (*Saccharomyces cerevisiae*, *Schizosaccharomyces pombe*, *Neurospora crassa* and *Aspergillus fumigatus*); and (iii) plants (eudicot (*Arabidopsis thaliana*), monocot (*Oryza sativa*), bryophyta (*Physcomitrium patens*) and green algae (*Chlamydomonas reinhardtii*)). In the case of an MC isoform only present in *H. sapiens* among the above‐mentioned genomes, the sequences of three other mammals were included: cow (*Bos taurus*), dog (*Canis familiaris*), and mouse (*Mus musculus*). The belonging of all protein sequences to the subfamilies was confirmed by phylogenetic analysis (Figure S7) and mainly by using OrthoDB v11 and OMA 2024, and sometimes also considering Treefam v9, EggNOG 6.0 and HOGENOM (Table S1) (Altenhoff et al., 2024; Hernández‐Plaza et al., 2023; Kuznetsov et al., 2023; Penel et al., 2009; Schreiber et al., 2014).

### 4.2 Sequence alignments and phylogenetic analysis

Multiple protein sequence alignments were done with Clustal Omega in Seaview4 with default parameters (Gouy et al., 2010; Sievers et al., 2011). Phylogenetic trees were constructed by using PhyML v3.1 (Guindon et al., 2010) in Seaview4 with default parameters: model: LG; amino acid equilibrium frequencies: model‐given; invariable sites: none; across‐site variation: optimized; tree searching operations: NNI; starting tree: BioNJ with optimized tree topology. The resulting phylogenetic trees were drawn in FigTree v1.4.2 (http://tree.bio.ed.ac.uk/software/figtree/).

### 4.3 Division of MC protein sequences into exons, transmembrane α‐helices, and “OLD” sequence sets

Genome Data Viewer (in NCBI Gene) was used to visualize the exons and introns in “transcription‐translation aligned” sequences of the MC genes, cDNA, and proteins to detect the IPs, which were mapped onto the protein sequence alignments manually (Figure S8). The IPs in the protein sequence alignments were carefully transferred onto the schematic figures, maintaining their proportional relative positions to allow comparison of identical positions in different MC subfamilies, in a similar way as previously done (Monné et al., 2023). The codon frame of the identical IPs was checked in the “transcription‐translation aligned” sequences.

All the MC protein sequences were divided into different sequence sets. The total set of exon and full‐length protein sequences was created, including only the sequences of the MC transporter domain (from 25 residues before the proline in the first signature motif sequence and until 22 residues after the last glycine in the third signature motif) and excluding N‐ and C‐terminal extensions. The total set of the H1‐H6 sequence collection contained the central portions of the MC transmembrane α‐helices, including the binding site residues in conformations of the carrier with the m‐ and c‐gates closed. These sequences included: the odd‐numbered α‐helices from 13 residues before the signature motif proline to 6 residues after; and the even‐numbered α‐helices from the last signature motif glycine and the following 16 residues. The “OLD” sequence sets contained separately the exon/full‐length and H1‐H6 sequences of MC subfamilies that had appeared before the query subfamily (as deduced from Figure S2). The “OLD” sequence sets consisted of MC subfamilies found in different combinations of kingdoms: those present in plant+fungi+animal, plant+fungi, and plant+animal for the original and plant sequence queries; the same sequences with the addition of those that appeared in fungi+animals for the animal sequence queries; all the sequences indicated before with the addition of those that appeared in animals for mammalian sequence queries.

### 4.4 Exon protein sequence similarity analysis

For the exon similarity analysis, the total set of full‐length and exon protein sequences was used in pairwise sequence alignments in STRAP using ClustalW (Gille & Frömmel, 2001), which assigns an alignment score for each sequence pair. The SI of each pairwise alignment was calculated by ClustalO (Sievers et al., 2011). For each exon (confined by human or *A. thaliana* IPs), the “most similar” sequence was identified separately for exon and full‐length sequences, based on the highest alignment score, provided it also had an SI of at least 30% within the three highest‐ranking hits. For the same exon across all orthologs of the same subfamily as queries, the number of the top sequence hits of different MC subfamilies was counted, and their average alignment score and average SI were calculated. Besides the top hit, also highly similar sequences that ranked just below were considered in a measurement to give an idea of how frequent and how strong a subfamily is represented among the “top 5% hits”. The exon and full‐length sequence hits with alignment score and SI no <5% below the top hit were used in this calculation: (1) for each exon query the hit alignment scores of each MC subfamily were summed and divided by the total number of hits; (2) these fractions, from all results of the same subfamily exon query of all orthologs, were then summed and normalized by the total alignment score fractions of all subfamily hits.

To predict the “most similar” sequence for each exon in relation to MC subfamilies, a confidence score (ranging from 0 to 5) was calculated. One point was assigned for each of the following four criteria, provided a single subfamily had an absolute majority (>50%) based on at least four sequence hits in each of them: (1) the number of most similar exon sequence hits, (2) the top 5% exon hits, (3) the number of the most similar full‐length sequence hits and (4) the top 5% full‐length sequence hits. An additional point was awarded if only one subfamily was present across all four parameters, bringing the maximum confidence score to 5, indicating the highest confidence in subfamily similarity prediction. If the analysis of an exon did not result in a confidence score by reaching the thresholds of the parameters (above), but a clear trend for a single subfamily hit was observed, a final prediction was indicated in parentheses. The procedure of identifying the most similar sequences to the MC exons was performed by searching both the total and “OLD” sequence sets with MC exon and full‐length sequences.

### 4.5 Transmembrane α‐helix sequence similarity analysis

For the MC transmembrane α‐helix sequence analysis, the H1‐H6 sequence dataset was used in pairwise sequence alignments without gaps, and the SI was calculated by ClustalO (Sievers et al., 2011). The same transmembrane α‐helix of all orthologs of the same subfamily was used as the query, and the percentage of each MC subfamily hit (with the highest SI and other than the query) was calculated. The evidence for being the “most similar” MC subfamily transmembrane α‐helix was considered; strong, if the hit subfamily was found in more than 50% of hits and was mutually found in more than 50% of the hit subfamily; good, if the hit subfamily was found in more than 50% of hits; and weak, if the percentage was at least 40% and double that of the second best. The procedure of identifying the most similar MC transmembrane α‐helix sequences was performed by searching both the total and “OLD” H1‐H6 sequence sets.

### 4.6 GARD analysis

GARD on the Datamonkey Adaptive Evolution server was used with default parameters (Data type: protein; Run mode: faster; Genetic code: universal code; Site‐to‐site rate variation: none; Rate classes: 2) for detecting recombination between two MC subfamilies, for which the exon and transmembrane α‐helix sequence similarity analysis had suggested it (Kosakovsky Pond et al., 2006; Weaver et al., 2018). The ortholog protein sequences of the two MC subfamilies from vertebrates, fungi, and plants were aligned separately and analyzed through GARD. The GARD results displayed evidence for recombination breakpoints confining recombined fragments that were used in alternative phylogenetic trees. The closest GARD‐predicted recombined fragments between two MC subfamilies from any organism group are displayed in the figures.

## AUTHOR CONTRIBUTIONS

**Magnus Monné:** Conceptualization; methodology; investigation; formal analysis; writing – original draft; writing – review and editing. **Daniela Valeria Miniero:** Investigation. **Luigi Palmieri:** Conceptualization; investigation; writing – review and editing. **Rosa Calvello:** Investigation. **Antonia Cianciulli:** Investigation. **Ferdinando Palmieri:** Conceptualization; investigation; writing – review and editing.

## CONFLICT OF INTEREST STATEMENT

The authors declare no conflicts of interest.

## Supporting information


**TABLE S1.** The total collection of MC proteins divided into subfamilies. The MC protein sequences from *H. sapiens*, *A. thaliana* and *S. cerevisiae* were used to collect orthologs in well‐annotated genomes based on unambiguous reciprocal best hits. This collection contained a total of 768 MC proteins, which are listed according to their main phylogenetic cluster and subfamily. The belonging of all protein sequences to the MC subfamilies was confirmed by using mainly OrthoDB v11 and OMA 2024, and sometimes also considering Treefam v9, EggNOG 6.0 and HOGENOM. The ortholog groups in the hierarchy indicating the specific MC subfamilies are in color.
**TABLE S2.** Predictions of the most similar sequence to the MC subfamily exons. The table is divided into sections of MC subfamilies based on the main phylogenetic clusters: (a) MC‐NT2; (b) MC‐NT1; (c) MC‐CA; (d) MC‐AAP; (e) MC‐AAN; (f) MCs outside the main clusters; and more or less in the order of the phylogenetic tree (Figure S1). The plant subfamily members are indicated with “p” in front of the subfamily name. The exons are numbered as in the alignments for each subfamily (Figure S8). In the subfamily exons column, the OLD exon and full‐length sequence set is indicated when the total set is not used in the analysis. The results for each subfamily exon are divided into the columns for the most similar ES, the most similar full‐length sequence (FLS) and the final prediction. For details of the calculations of the parameters (subcolumns with a name initiating with “top”) and confidence scores, see the Section 4. The number of top ES and FLS hits out of the total for the same exon of all analyzed subfamily members is given in parentheses. The average alignment score (AAS) and average sequence identity (ASI) are given with standard errors of the mean. For the confidence score of the final prediction of the most similar sequence, one point each was given to the hit subfamily with an absolute majority (>50% based on at least four sequence hits) in the four subcolumns with a name initiating with “top”. An additional point was given to subfamilies found exclusively in all four subcolumns, and clear observed trends for final prediction subfamilies not reaching the thresholds of confidence scores are indicated in parentheses. Source: * only the most similar ES or FLS prediction was considered when the comparison of their average alignment scores had Chi‐square test *p*‐value <0.05; # subfamily hit based on one and the same single sequence hit.
**TABLE S3.** Predictions of the most similar MC subfamily transmembrane α‐helices. The table is divided into sections of MC subfamilies based on the main phylogenetic clusters: (a) MC‐NT2; (b) MC‐NT1; (c) MC‐CA; (d) MC‐AAP; (e) MC‐AAN; (f) MCs outside the main clusters; and more or less in the order of the phylogenetic tree (Figure S1). In the MC subfamily column of queries the OLD H1‐H6 sequence set is indicated when the total set is not used in the analysis. The results for the most similar transmembrane α‐helices are indicated with the percentage of the number of subfamily H1‐H6 hits with the highest SI to the same transmembrane α‐helix of all orthologs of the same subfamily. The “most similar” MC subfamily transmembrane α‐helix prediction was considered; strong (in bold), if the hit subfamily was found in more than 50% hits and was mutually found in more than 50% of the hit subfamily; good, if the hit subfamily was found in more than 50% hits; and weak (light green), if the percentage was at least 40% and double than the second best. For the strong and good predictions, the average SI of the hits is given in parentheses with the standard errors of the mean. The remaining results are shown in gray. Source: *mainly depending on one sequence hit.
**FIGURE S1.** Phylogenetic tree of the human, Arabidopsis, and yeast MCs. The phylogenetic tree with the 53 human, 58 *A. thaliana*, and 35 *S. cerevisiae* MCs was constructed by using PhyML v3.1 from a multiple‐sequence alignment with ClustalO in Seaview4 and drawn in FigTree v1.4.2 (rooted to the left). Bootstrap values for 1000 replicates are reported on the nodes. The names of the MCs initiate with the subfamily name followed by the organism abbreviation (Hs for *H. sapiens*, At for *A. thaliana*, and Sc for *S. cerevisiae*) and the protein name. The main clusters of MCs for nucleotides (MC‐NT1 and ‐NT2), carboxylates (MC‐CA), positively (MC‐AAP) and negatively (MC‐AAN) charged amino acids are indicated in green, red, blue and orange, respectively. The MC subfamily names and their transported substrates are shown in the table connected to the phylogenetic tree.
**FIGURE S2.** Simplified tree of the appearance of MC subfamilies or new isoforms in the plant, fungi, and animal kingdoms as well as in mammals. The original MC subfamilies present in plant and animal/fungi branches are indicated at the origin of the tree. AGC and GlyC are in italics because they are only found in *P. patens* of the plant species investigated in this study. In‐ and out‐going arrows indicate the appearance and disappearance, respectively, of subfamilies in the different branches. New isoforms are followed by the subfamily name in parentheses. The subfamilies are color‐coded according to their positions among the main phylogentic clusters (Figure S1): MC‐NT1‐2 (green), MC‐CA (red), MC‐AAP (blue) and MC‐AAN (orange).
**FIGURE S3.** The most similar sequences to the original MC‐NT1 cluster subfamily exons and transmembrane α‐helices. The MC topology and MC subfamily sequences, as well as the results of GARD analysis, the most similar sequences in the total (TOT) and OLD sequence sets of the ESs, plant ESs (pESs) and H1‐H6 (extracted from Tables S2 and S3) are all displayed as in Figure 2. The MC‐NT1 cluster subfamilies are shown on the left with specific colors and they appeared at the origin (according to Figure S2). Other MC‐NT1 cluster subfamilies are shown in Figure S4. The ES results of the CoC subfamily are divided into SLC25A42 and SLC25A16 orthologs because they have conserved IPs in different positions. The ES results are shown with the conserved subfamily IPs as small vertical rectangles, of which those occurring with the same codon frame position only among MC‐NT1 cluster subfamilies are filled with gray and in more than one MC‐NT1 and MC‐NT2 cluster subfamily are filled with black. Segments similar to the MC‐NT2 cluster NDT and MFT/FAD subfamilies are indicated in purple and magenta, respectively.
**FIGURE S4.** The most similar sequences to the mammalian‐, animal‐, and plant‐specific MC‐NT1 cluster subfamily/isoform exons and transmembrane α‐helices. The MC topology and MC subfamily sequences, as well as the results of GARD analysis, the most similar sequences in the total (TOT) and OLD sequence sets of the ESs, plant ESs (pESs), and H1‐H6 (extracted from Tables S2 and S3) are all displayed as in Figure 2. The MC‐NT1 cluster subfamilies are shown on the left, and they are divided into those that appeared in mammals (M), animals (A), and plants (P) (according to Figure S2). The ES results are shown with the conserved subfamily IPs as small vertical rectangles, of which those occurring with the same codon frame position only among MC‐NT1 cluster subfamilies are filled with black. Segments similar to the original MC‐NT1 cluster subfamilies are indicated with the colors as in Figure S3.
**FIGURE S5.** The most similar sequences to the exons and transmembrane α‐helices of the other MC subfamilies outside the main clusters. The MC topology and MC subfamily sequences as well as the results of GARD analysis, the most similar sequences in the total (TOT) and OLD sequence sets of the ESs, plant ESs (pESs) and H1‐H6 (extracted from Tables S2 and S3) are all displayed as in Figure 2. The MC subfamilies outside the main cluster (not included in Figure 6) are shown on the left and divided into those that appeared in animals (A), fungi (F), plants (P), and at the origin (O) (according to Figure S2). For some MC subfamilies outside the main cluster, no result is shown because they lacked conserved IPs and/or H1‐H6 results. IPs commonly found in the MC‐NT1‐2 and MC‐CA cluster subfamilies with the same codon frame position are filled with green and red, respectively. The segments similar MC‐NT1‐2, MC‐CA, MC‐AAP, and MC‐AAN cluster subfamilies are colored green, red, blue, and orange, respectively, whereas the other colors belong to the MC subfamilies in Figure 6.
**FIGURE S6.** Guidelines for how the segment analysis approach may be applied to other protein superfamilies. The flow chart in Figure 1 has been generalized in order to apply to the analysis of all protein superfamilies. The directions of the analysis and assembly of sequence sets are indicated by black and red arrows, respectively. Sequence collections, data analysis, and results interpretation are shown in boxes colored in cyan, yellow, and green, respectively.
**FIGURE S7.** Phylogenetic tree with the total collection of MC proteins. The phylogenetic tree with all the MC carriers listed in Table S1 was constructed by using PhyML v3.1 from a multiple‐sequence alignment with ClustalO in Seaview4 and drawn in FigTree v1.4.2 (rooted to the left). The sequence names have the same color as the subfamilies name. The main clusters of MCs for nucleotides (MC‐NT1 and ‐NT2), carboxylates (MC‐CA), positively (MC‐AAP) and negatively (MC‐AAN) charged amino acids are indicated with different colors.
**FIGURE S8.** Multiple protein sequence alignments of MC subfamily members with the exons indicated. The protein sequences of each MC subfamily (listed in Table S1) were divided based on their conserved IPs into organism kingdoms: (i) animals (including the sequences from *Homo sapiens*, *Gallus gallus*, *Xenopus tropicalis*, *Danio rerio*, *Caenorhabditis elegans*, *Aplysia californica*, *Ciona intestinalis, Acanthaster planci, Drosophila melanogaster*, *Bemisia tabaci*, *Spodoptera frugiperda*, *Acromyrmex echinatior*, *Cryptotermes secundus and Dendroctonus ponderosae*), (ii) plants (*Arabidopsis thaliana*, *Oryza sativa*, *Physcomitrium patens* and *Chlamydomonas reinhardtii*) and (iii) fungi (*Saccharomyces cerevisiae* [no introns in MC genes], *Schizosaccharomyces pombe*, *Neurospora crassa* and *Aspergillus fumigatus*). In the case of an MC isoform only present in *H. sapiens* of the above‐mentioned genomes, the sequences of three other mammals were included: *Bos taurus*, *Canis familiaris*, and *Mus musculus*. The divided MC subfamily sequences were aligned, and the order of the subfamily alignments presented follows the division of the main MC clusters (a, MC‐NT2; b, MC‐NT1; c, MC‐CA; d, MC‐AAP; e, MC‐AAN; f, MCs outside the main clusters) and more or less in the order of the phylogenetic tree (Figure S1). The IPs are indicated in red: single residues, when the IP is found in that codon, and double residues, when it is found in between the codons. The exons within the MC transporter domains of *H. sapiens* and *A. thaliana* are numbered. Some of the sequences have been truncated in the N‐ and/or C‐terminal outside the MC transporter domain.

## Data Availability

The data that supports the findings of this study are available in the supplementary material of this article.
