# Supplementary material for: Evolutionary history and recombination in the mitochondrial carrier SLC25 superfamily analyzed by similarities in the exon and transmembrane α‐helix sequences
Source: Protein Sci. 2026 Jul 20;35(8):e70727. doi: 10.1002/pro.70727 (PMC13385207; doi:10.1002/pro.70727)
Supplement: Supplementary file 1 — TABLE S1. The total collection of MC proteins divided into subfamilies. The MC protein sequences from H. sapiens, A. thaliana and S. cerevisiae were used to collect orthologs in well‐annotated genomes based on unambiguous reciprocal best hits. This collection contained a total of 768 MC proteins, which are listed according to their main phylogenetic cluster and subfamily. The belonging of all protein sequences to the MC subfamilies was confirmed by using mainly OrthoDB v11 and OMA 2024, and sometimes also considering Treefam v9, EggNOG 6.0 and HOGENOM. The ortholog groups in the hierarchy indicating the specific MC subfamilies are in color. TABLE S2. Predictions of the most similar sequence to the MC subfamily exons. The table is divided into sections of MC subfamilies based on the main phylogenetic clusters: (a) MC‐NT2; (b) MC‐NT1; (c) MC‐CA; (d) MC‐AAP; (e) MC‐AAN; (f) MCs outside the main clusters; and more or less in the order of the phylogenetic tree (Figure S1). The plant subfamily members are indicated with “p” in front of the subfamily name. The exons are numbered as in the alignments for each subfamily (Figure S8). In the subfamily exons column, the OLD exon and full‐length sequence set is indicated when the total set is not used in the analysis. The results for each subfamily exon are divided into the columns for the most similar ES, the most similar full‐length sequence (FLS) and the final prediction. For details of the calculations of the parameters (subcolumns with a name initiating with “top”) and confidence scores, see the Section 4. The number of top ES and FLS hits out of the total for the same exon of all analyzed subfamily members is given in parentheses. The average alignment score (AAS) and average sequence identity (ASI) are given with standard errors of the mean. For the confidence score of the final prediction of the most similar sequence, one point each was given to the hit subfamily with an absolute majority (>50% based on at least four seq [file PRO-35-e70727-s001.pdf]

Supplementary material for:

Evolutionary history and recombination in the mitochondrial carrier SLC25 superfamily analyzed by similarities in the exon and transmembrane α-helix sequences

Authors

Magnus Monné[1,2,*], Daniela Valeria Miniero[1,3], Rosa Calvello[1], Antonia Cianciulli[1], Luigi Palmieri[1,4] and Ferdinando Palmieri[1,4]

Affiliations

[1]Department of Biosciences, Biotechnology and Environment, University of Bari Aldo Moro, Bari, Italy

[2]Department of Health Sciences, University of Basilicata, Potenza, Italy

[3]Department of Medicine and Surgery, LUM University Giuseppe Degennaro, Casamassima, Italy

[4]CNR Institute of Biomembranes, Bioenergetics and Molecular Biotechnologies (IBIOM), Bari, Italy

* Corresponding authors: Magnus Monné (magnus.monne@unibas.it)

Supplementary Figures S1-S8 and Supplementary Tables S1-S3

<u>Index</u>

FIGURE S1……………………………………………………………2
FIGURE S2……………………………………………………………4
FIGURE S3……………………………………………………………6
FIGURE S4……………………………………………………………8
FIGURE S5…………………………………………………………...10
FIGURE S6…………………………………………………………...12
FIGURE S7…………………………………………………………...14
FIGURE S8…………………………………………………………...16
TABLE S1……………………………………………………..……33
TABLE S2……………………………………………………..……51
TABLE S3……………………………….…………………………..154

FIGURE S1. Phylogenetic tree of the human, Arabidopsis, and yeast MCs. The phylogenetic tree with the 53 human, 58 *A. thaliana*, and 35 *S. cerevisiae* MCs was constructed by using PhyML v3.1 from a multiple-sequence alignment with ClustalO in Seaview4 and drawn in FigTree v1.4.2 (rooted to the left). Bootstrap values for 1000 replicates are reported on the nodes. The names of the MCs initiate with the subfamily name followed by the organism abbreviation (Hs for *H. sapiens*, At for *A. thaliana*, and Sc for *S. cerevisiae*) and the protein name. The main clusters of MCs for nucleotides (MC-NT1 and -NT2), carboxylates (MC-CA), positively (MC-AAP) and negatively (MC-AAN) charged amino acids are indicated in green, red, blue and orange, respectively. The MC subfamily names and their transported substrates are shown in the table connected to the phylogenetic tree.

Fig. S1.

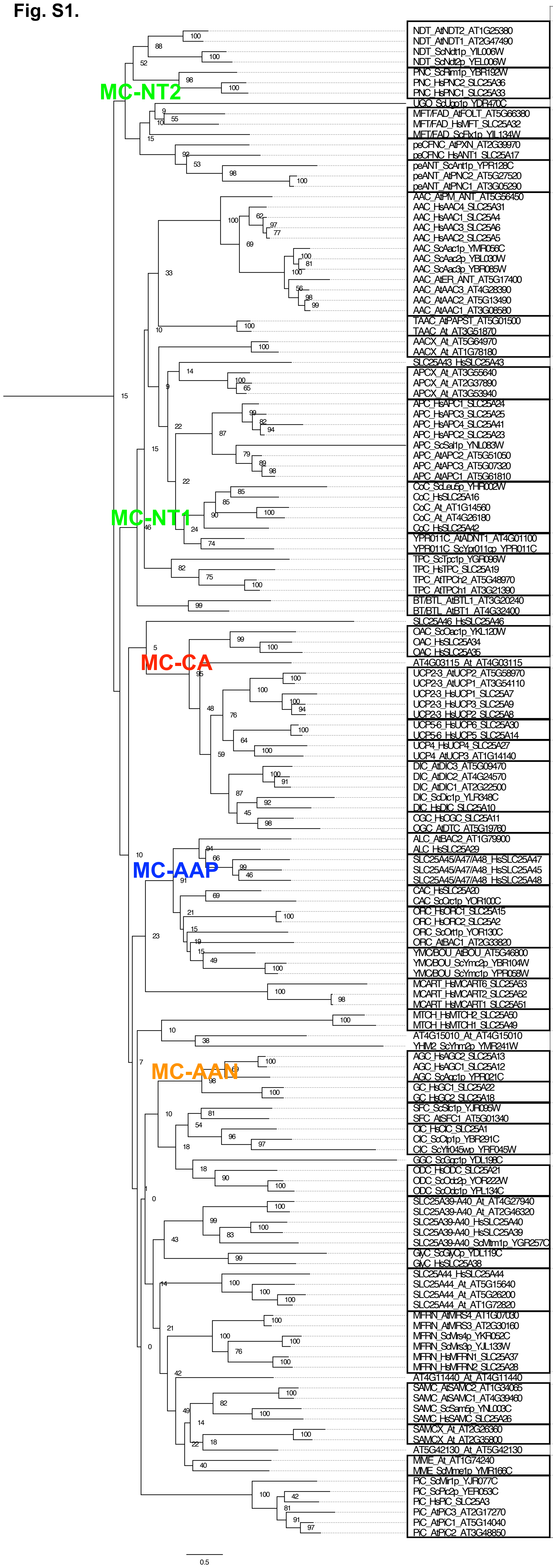

MC subfamily	name	substrates
NDT	NAD+ transporter	NAD+, (d)AMP, (d)GMP
PNC	pyrimidine carrier	pyrimidine (deoxy)nucleotides
UGO		
MFT/FAD	FAD carrier	FAD, folate
peCFNC	peroxisomal CoA, NAD+, FAD carrier	CoA, NAD+, FAD
peANT	peroxisomal adenine nucleotide carrier	(d)ATP, (d)ADP, (d)AMP
AAC	ADP/ATP carrier	ADP, ATP
TAAC	thylakoid ADP/ATP carrier	ATP, ADP, PAPS
AACX	AAC-like carrier	
SLC25A43		
APCX	APC-like carrier	
APC	ATP-Mg/phosphate carrier	ATP-Mg, phosphate, ATP, ADP, AMP
CoC	CoA carrier	CoA, dephospho-CoA, adenosine 3,5-diphosphate
YPR011C		PAPS, APS, phosphate, sulfate
TPC	thiamine pyrophosphate carrier	thiamine pyrophosphate, thiamine monophosphate, dNDP, dNTP, ADP, ATP
BT/BTL	Brittle	ATP, ADP, AMP
SLC25A46		outer membrane fusion/fission
OAC	oxaloacetate/sulfate	oxaloacetate, malonate, sulfate, thiosulfate, phosphoenolpyruvate
AT4G03115		
UCP2-3	uncoupling proteins UCP1-3	H+, 4-carbon dicarboxylates, aspartate, phosphate, sulfate
UCP5-6	uncoupling proteins UCP5-6	aspartate, malate, malonate, succinate, phosphate, sulfate, sulfite, thiosulfate
UCP4	uncoupling protein UCP4	aspartate
DIC	dicarboxylate carrier	malate, malonate, succinate, phosphate, sulfate, sulfite, thiosulfate (oxaloacetate)
OGC	oxoglutarate carrier	2-oxoglutarate, malate, malonate, succinate and oxaloacetate (sulfate, tricarboxylates)
ALC	arginine/lysine carrier	arginine, lysine
SLC25A45/A47/A48		choline, trimethyl-lysine
CAC	carnitine carrier	carnitine, acylcarnitine
ORC	ornithine carrier	ornithine, citrulline, arginine, lysine
YMC/BOU		glutamate
MCART		NAD+
MTCH		outer membrane insertase
AT4G15010		
YHM2		citrate, 2-oxoglutarate
AGC	aspartate/glutamate carrier	aspartate, glutamate
GC	glutamate carrier	glutamate
SFC	succinate-fumarate carrier	succinate, fumarate (citrate, isocitrate)
CIC	citrate carrier	citrate, isocitrate, malate
GGC	GTP/GDP carrier	(d)GNP
ODC	oxodicarboxylate carrier	2-oxoadipate, 2-oxoglutarate (2-aminoadipate, citrate)
SLC25A39-A40		glutathione
GlyC	glycine carrier	glycine
SLC25A44		valine, isoleucine, leucine
MFRN	mitoferrin	iron
AT4G11440		
SAMC	SAM carrier	S-adenosylmethionine, S-adenosylhomocysteine
SAMCX	SAM-like carriers	
AT5G42130		
MME	magnesium exporter	magnesium
PiC	phosphate carrier	phosphate

FIGURE S2. Simplified tree of the appearance of MC subfamilies or new isoforms in the plant, fungi, and animal kingdoms as well as in mammals. The original MC subfamilies present in plant and animal/fungi branches are indicated at the origin of the tree. AGC and GlyC are in italics because they are only found in *P. patens* of the plant species investigated in this study. In- and out-going arrows indicate the appearance and disappearance, respectively, of subfamilies in the different branches. New isoforms are followed by the subfamily name in parentheses. The subfamilies are color-coded according to their positions among the main phylogentic clusters (Figure S1): MC-NT1-2 (green), MC-CA (red), MC-AAP (blue) and MC-AAN (orange).

Fig. S2.

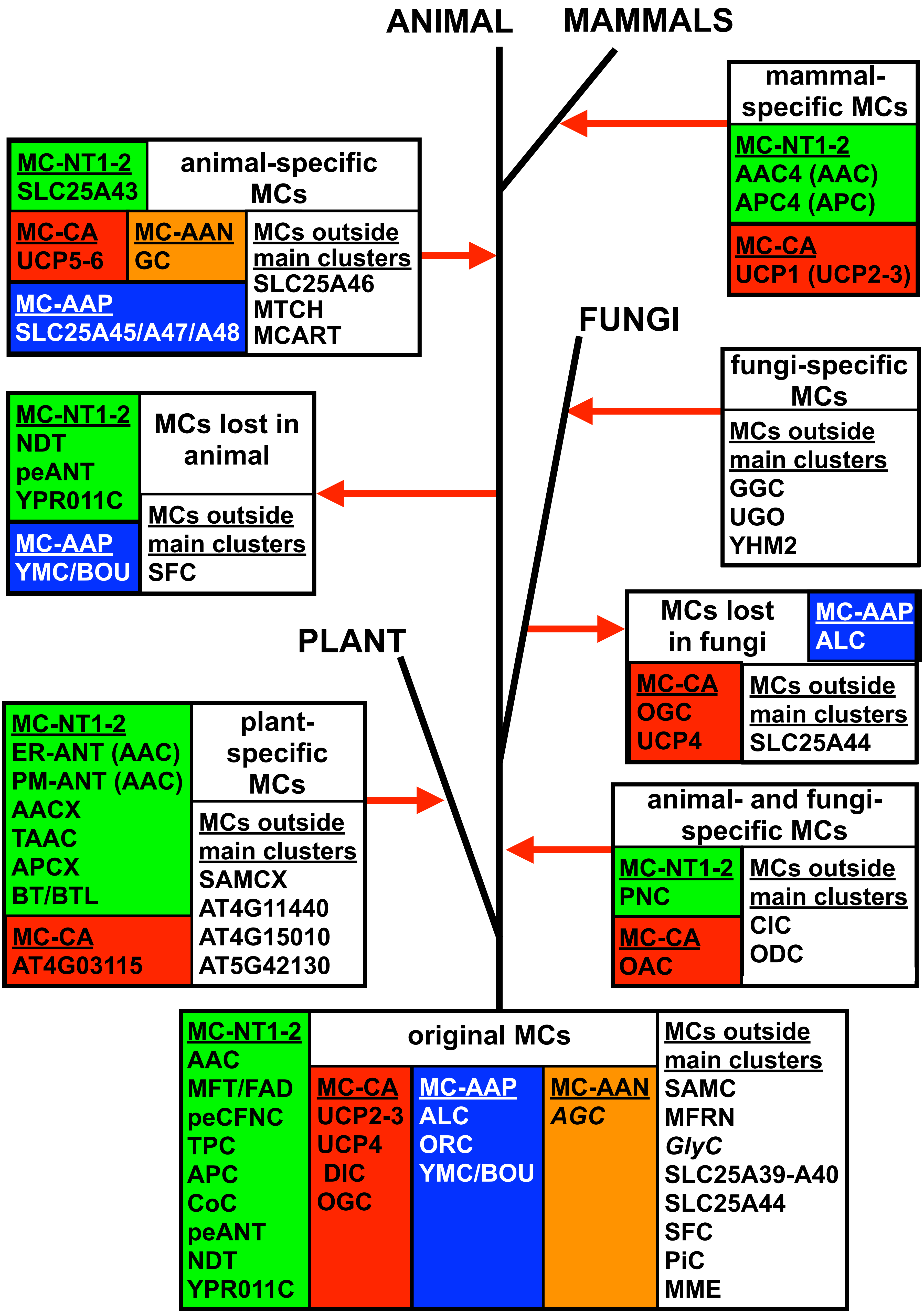

ANIMAL
MAMMALS
mammal-specific MCs
MC-NT1-2
AAC4 (AAC)
APC4 (APC)
MC-CA
UCP1 (UCP2-3)
MC-NT1-2
SLC25A43
animal-specific MCs
MC-CA
UCP5-6
MC-AAN
GC
MCs outside main clusters
SLC25A46
MTCH
MCART
MC-AAP
SLC25A45/A47/A48
FUNGI
fungi-specific MCs
MCs outside main clusters
GGC
UGO
YHM2
MC-NT1-2
NDT
peANT
YPR011C
MCs lost in animal
MCs outside main clusters
SFC
MC-AAP
YMC/BOU
MCs lost in fungi
MC-AAP
ALC
MC-CA
OGC
UCP4
MCs outside main clusters
SLC25A44
PLANT
MC-NT1-2
ER-ANT (AAC)
PM-ANT (AAC)
AACX
TAAC
APCX
BT/BTL
plant-specific MCs
MCs outside main clusters
SAMCX
AT4G11440
AT4G15010
AT5G42130
MC-CA
AT4G03115
animal- and fungi-specific MCs
MC-NT1-2
PNC
MCs outside main clusters
CIC
ODC
MC-CA
OAC
MC-NT1-2
AAC
MFT/FAD
peCFNC
TPC
APC
CoC
peANT
NDT
YPR011C
original MCs
MC-CA
UCP2-3
UCP4
DIC
OGC
MC-AAP
ALC
ORC
YMC/BOU
MC-AAN
AGC
MCs outside main clusters
SAMC
MFRN
GlyC
SLC25A39-A40
SLC25A44
SFC
PiC
MME

FIGURE S3. The most similar sequences to the original MC-NT1 cluster subfamily exons and transmembrane α-helices. The MC topology and MC subfamily sequences as well as the results of GARD analysis, the most similar sequences in the total (TOT) and OLD sequence sets of the ESs, plant ESs (pESs) and H1-H6 (extracted from Tables S2 and S3) are all displayed as in Figure 2. The MC-NT1 cluster subfamilies are shown on the left with specific colors and they appeared at the origin (according to Figure S2). Other MC-NT1 cluster subfamilies are shown in Figure S4. The ES results of the CoC subfamily are divided into SLC25A42 and SLC25A16 orthologs because they have conserved IPs in different positions. The ES results are shown with the conserved subfamily IPs as small vertical rectangles, of which those occurring with the same codon frame position only among MC-NT1 cluster subfamilies are filled with gray and in more than one MC-NT1 and MC-NT2 cluster subfamily are filled with black. Segments similar to the MC-NT2 cluster NDT and MFT/FAD subfamilies are indicated in purple and magenta, respectively.

Fig. S3.

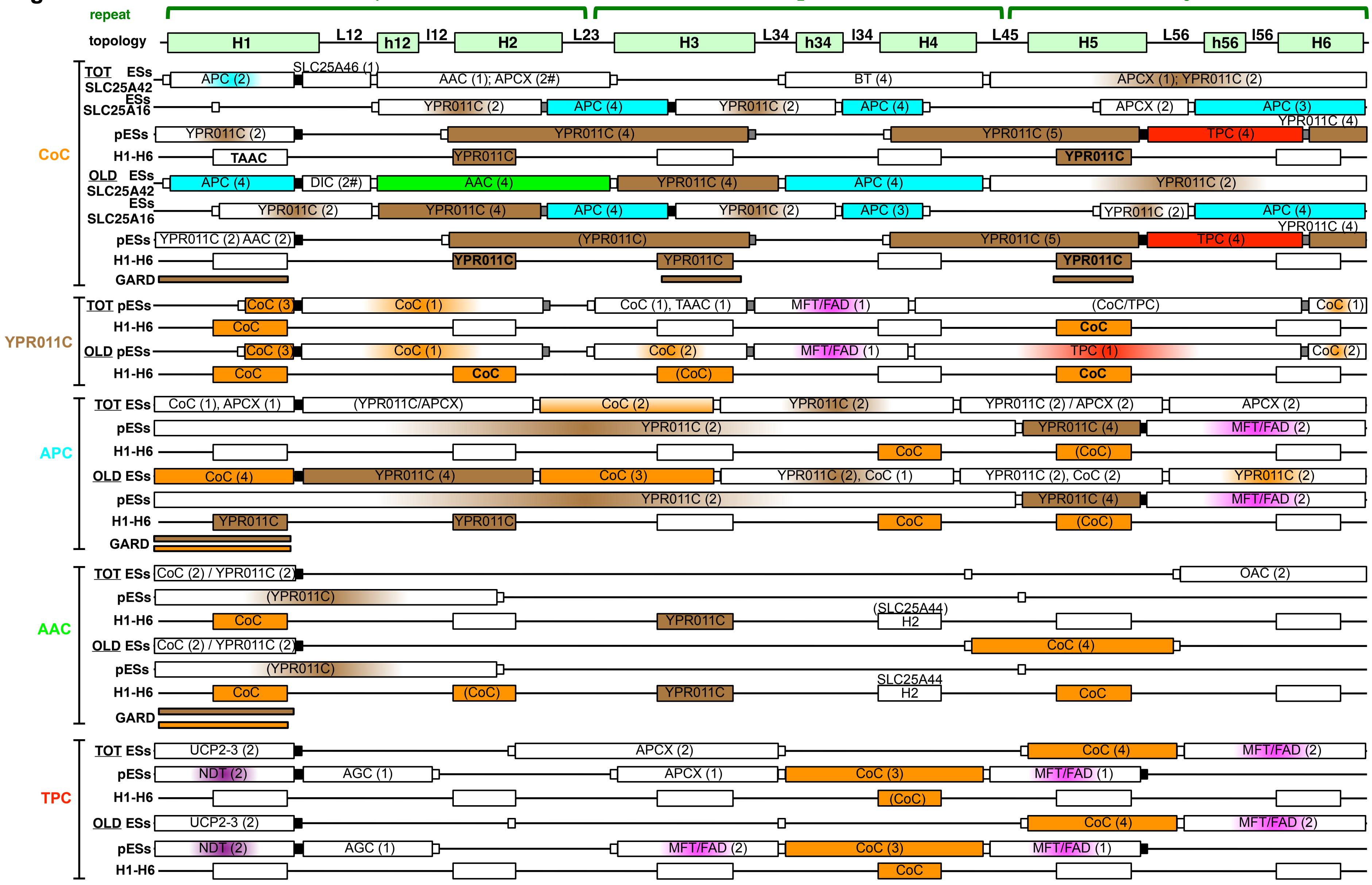

repeat
1
2
3
topology
H1
L12
h12
I12
H2
L23
H3
L34
h34
I34
H4
L45
H5
L56
h56
I56
H6
CoC
TOT ESs SLC25A42
ESs SLC25A16
pESs
H1-H6
OLD ESs SLC25A42
ESs SLC25A16
pESs
H1-H6
GARD
APC (2)
SLC25A46 (1)
AAC (1); APCX (2#)
BT (4)
APCX (1); YPR011C (2)
YPR011C (2)
APC (4)
APC (4)
APCX (2)
APC (3)
YPR011C (4)
YPR011C (4)
YPR011C (5)
TPC (4)
TAAC
YPR011C
DIC (2#)
AAC (4)
APC (3)
YPR011C (2) AAC (2)
(YPR011C)
YPR011C
TOT pESs
OLD pESs
CoC (3)
CoC (1)
CoC (1), TAAC (1)
MFT/FAD (1)
(CoC/TPC)
CoC
CoC (2)
TPC (1)
(CoC)
APC
TOT ESs
OLD ESs
CoC (1), APCX (1)
(YPR011C/APCX)
YPR011C (2) / APCX (2)
APCX (2)
MFT/FAD (2)
CoC (4)
CoC (3)
YPR011C (2), CoC (1)
YPR011C (2), CoC (2)
AAC
CoC (2) / YPR011C (2)
OAC (2)
(SLC25A44)
H2
SLC25A44
TPC
UCP2-3 (2)
NDT (2)
AGC (1)
APCX (1)

FIGURE S4. The most similar sequences to the mammalian-, animal-, and plant-specific MC-NT1 cluster subfamily/isoform exons and transmembrane α-helices. The MC topology and MC subfamily sequences, as well as the results of GARD analysis, the most similar sequences in the total (TOT) and OLD sequence sets of the ESs, plant ESs (pESs), and H1-H6 (extracted from Tables S2 and S3) are all displayed as in Figure 2. The MC-NT1 cluster subfamilies are shown on the left, and they are divided into those that appeared in mammals (M), animals (A) and plants (P) (according to Figure S2). The ES results are shown with the conserved subfamily IPs as small vertical rectangles, of which those occurring with the same codon frame position only among MC-NT1 cluster subfamilies are filled with black. Segments similar to the original MC-NT1 cluster subfamilies are indicated with the colors as in Figure S3.

Fig. S4.

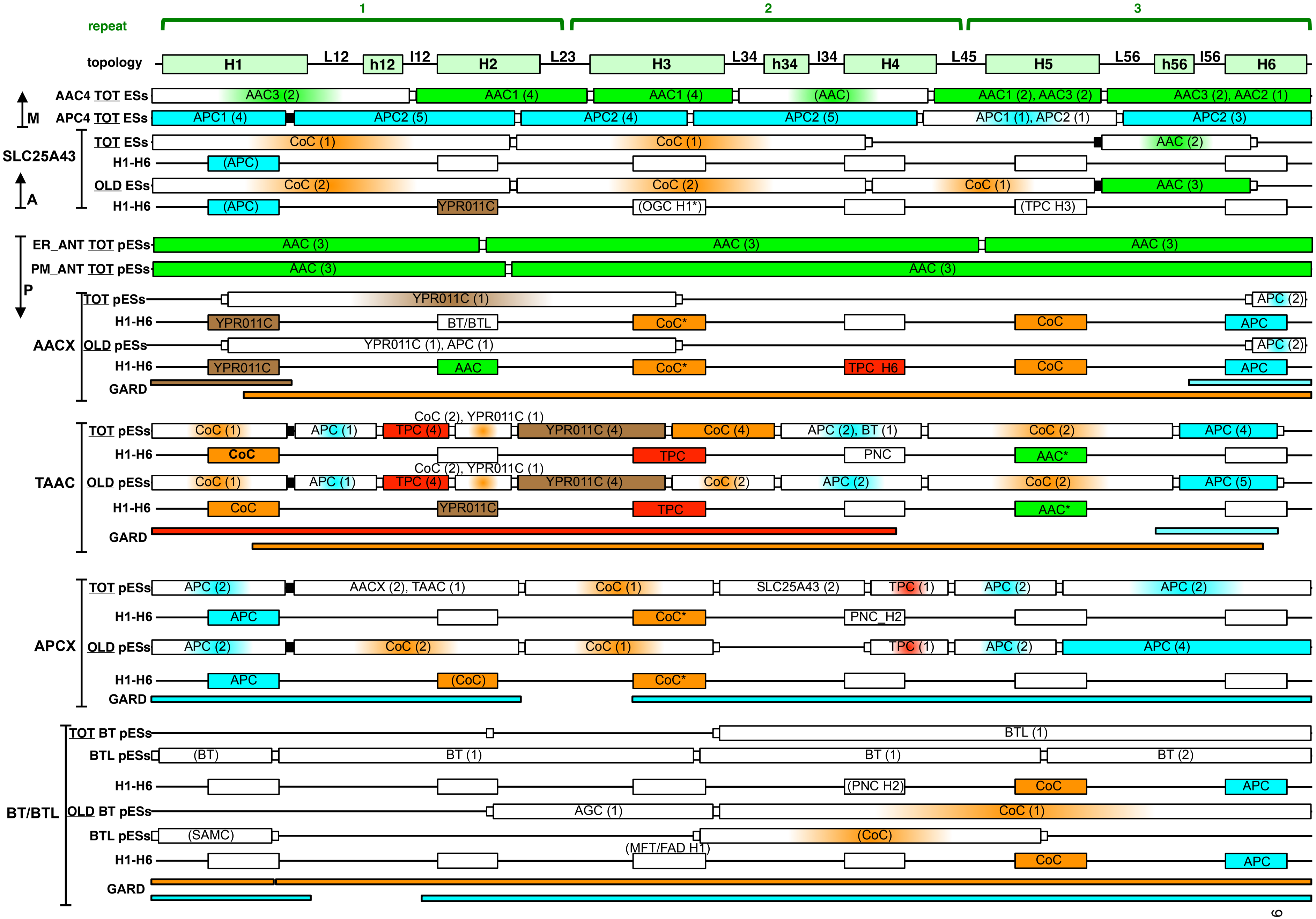
repeat
1
2
3
topology
H1
L12
h12
I12
H2
L23
H3
L34
h34
I34
H4
L45
H5
L56
h56
I56
H6
M
A
P
AAC4 TOT ESs
AAC3 (2)
AAC1 (4)
AAC1 (4)
(AAC)
AAC1 (2), AAC3 (2)
AAC3 (2), AAC2 (1)
APC4 TOT ESs
APC1 (4)
APC2 (5)
APC2 (4)
APC2 (5)
APC1 (1), APC2 (1)
APC2 (3)
SLC25A43
TOT ESs
CoC (1)
CoC (1)
AAC (2)
H1-H6
(APC)
OLD ESs
CoC (2)
CoC (2)
CoC (1)
AAC (3)
H1-H6
(APC)
YPR011C
(OGC H1*)
(TPC H3)
ER_ANT TOT pESs
AAC (3)
AAC (3)
AAC (3)
PM_ANT TOT pESs
AAC (3)
AAC (3)
AACX
TOT pESs
YPR011C (1)
APC (2)
H1-H6
YPR011C
BT/BTL
CoC*
CoC
APC
OLD pESs
YPR011C (1), APC (1)
APC (2)
H1-H6
YPR011C
AAC
CoC*
TPC H6
CoC
APC
GARD
TAAC
TOT pESs
CoC (1)
APC (1)
TPC (4)
CoC (2), YPR011C (1)
YPR011C (4)
CoC (4)
APC (2), BT (1)
CoC (2)
APC (4)
H1-H6
CoC
TPC
PNC
AAC*
OLD pESs
CoC (1)
APC (1)
TPC (4)
CoC (2), YPR011C (1)
YPR011C (4)
CoC (2)
APC (2)
CoC (2)
APC (5)
H1-H6
CoC
YPR011C
TPC
AAC*
GARD
APCX
TOT pESs
APC (2)
AACX (2), TAAC (1)
CoC (1)
SLC25A43 (2)
TPC (1)
APC (2)
APC (2)
H1-H6
APC
CoC*
PNC_H2
OLD pESs
APC (2)
CoC (2)
CoC (1)
TPC (1)
APC (2)
APC (4)
H1-H6
APC
(CoC)
CoC*
GARD
BT/BTL
TOT BT pESs
BTL (1)
BTL pESs
(BT)
BT (1)
BT (1)
BT (2)
H1-H6
(PNC H2)
CoC
APC
OLD BT pESs
AGC (1)
CoC (1)
BTL pESs
(SAMC)
(CoC)
(MFT/FAD H1)
H1-H6
CoC
APC
GARD

FIGURE S5. The most similar sequences to the exons and transmembrane α-helices of the other MC subfamilies outside the main clusters. The MC topology and MC subfamily sequences as well as the results of GARD analysis, the most similar sequences in the total (TOT) and OLD sequence sets of the ESs, plant ESs (pESs) and H1-H6 (extracted from Tables S2 and S3) are all displayed as in Figure 2. The MC subfamilies outside the main cluster (not included in Figure 6) are shown on the left and divided into those that appeared in animals (A), fungi (F), plants (P), and at the origin (O) (according to Figure S2). For some MC subfamilies outside the main cluster, no result is shown because they lacked conserved IPs and/or H1-H6 results. IPs commonly found in the MC-NT1-2 and MC-CA cluster subfamilies with the same codon frame position are filled with green and red, respectively. The segments similar MC-NT1-2, MC-CA, MC-AAP and MC-AAN cluster subfamilies are colored green, red, blue and orange, respectively, whereas the other colors belong to the MC subfamilies in Figure 6.

Fig. S5.

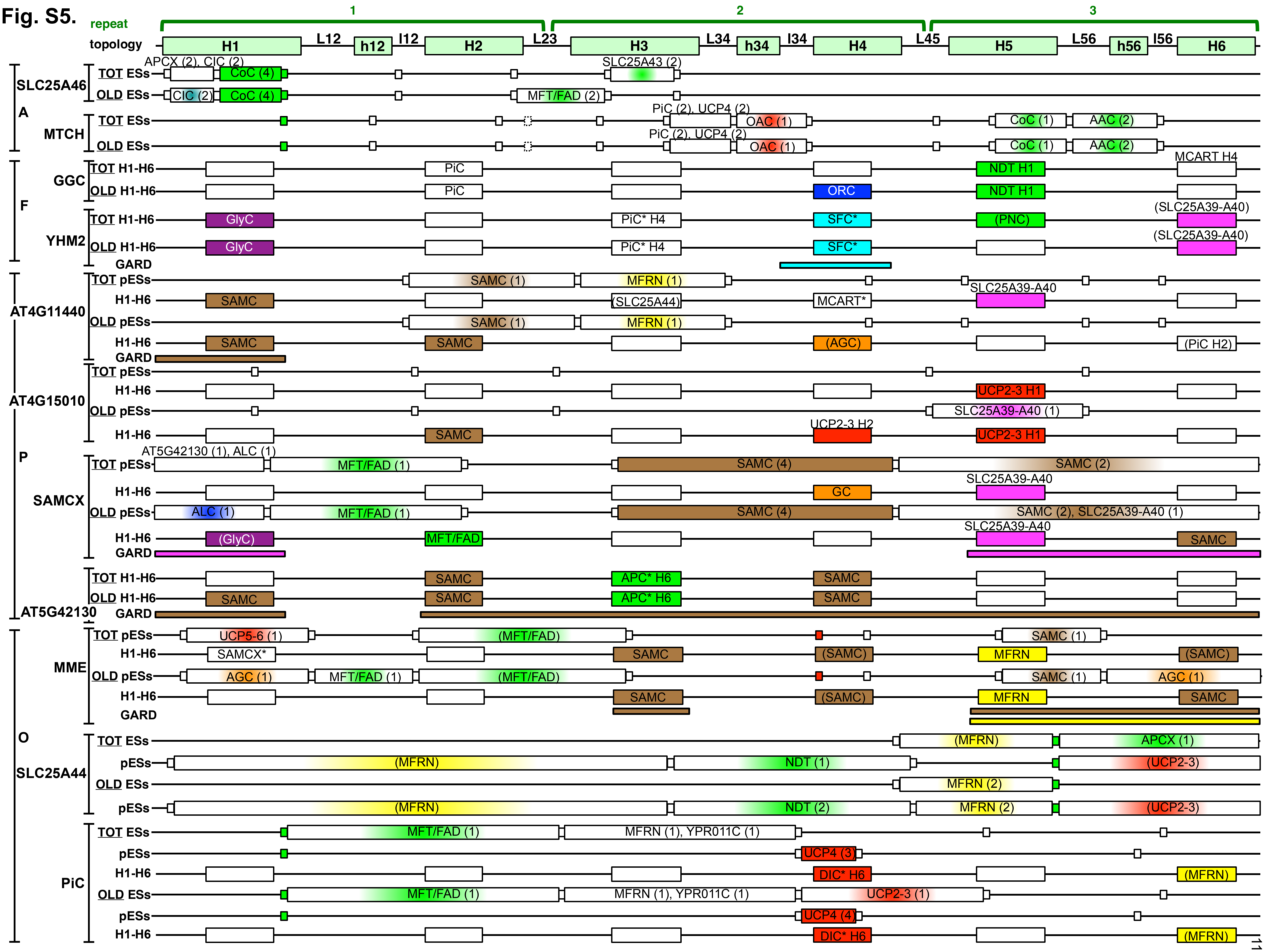
repeat
1
2
3
topology
H1
L12
h12
I12
H2
L23
H3
L34
h34
I34
H4
L45
H5
L56
h56
I56
H6
SLC25A46
A
MTCH
GGC
F
YHM2
AT4G11440
AT4G15010
P
SAMCX
AT5G42130
MME
O
SLC25A44
PiC
TOT ESs
OLD ESs
TOT H1-H6
OLD H1-H6
GARD
TOT pESs
OLD pESs
H1-H6
pESs
APCX (2), ClC (2)
CoC (4)
ClC (2)
SLC25A43 (2)
MFT/FAD (2)
PiC (2), UCP4 (2)
OAC (1)
CoC (1)
AAC (2)
PiC
NDT H1
MCART H4
ORC
GlyC
PiC* H4
SFC*
(PNC)
(SLC25A39-A40)
SAMC (1)
MFRN (1)
SLC25A39-A40
SAMC
(SLC25A44)
MCART*
(AGC)
(PiC H2)
UCP2-3 H1
SLC25A39-A40 (1)
UCP2-3 H2
AT5G42130 (1), ALC (1)
MFT/FAD (1)
SAMC (4)
SAMC (2)
GC
ALC (1)
SAMC (2), SLC25A39-A40 (1)
(GlyC)
MFT/FAD
APC* H6
UCP5-6 (1)
(MFT/FAD)
SAMCX*
(SAMC)
MFRN
AGC (1)
(MFRN)
APCX (1)
NDT (1)
(UCP2-3)
MFRN (2)
NDT (2)
MFRN (1), YPR011C (1)
UCP4 (3)
DIC* H6
UCP2-3 (1)
UCP4 (4)

FIGURE S6. Guidelines for how the segment analysis approach may be applied to other protein superfamilies. The flow chart in Figure 1 has been generalized in order to apply to the analysis of all protein superfamilies. The directions of the analysis and assembly of sequence sets are indicated by black and red arrows, respectively. Sequence collections, data analysis, and results interpretation are shown in boxes colored in cyan, yellow, and green, respectively.

Fig. S6.

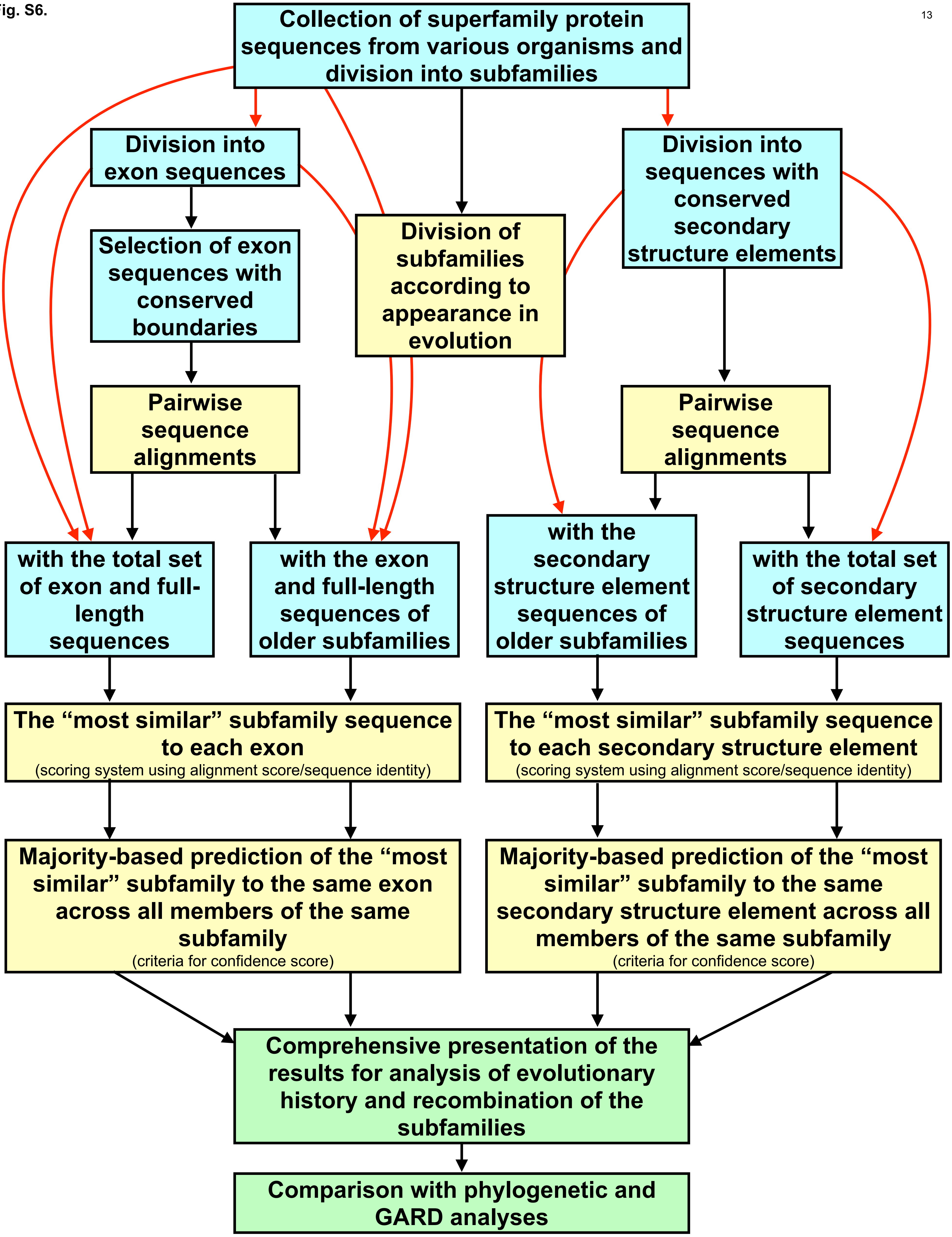
Collection of superfamily protein sequences from various organisms and division into subfamilies
Division into exon sequences
Selection of exon sequences with conserved boundaries
Pairwise sequence alignments
Division of subfamilies according to appearance in evolution
Division into sequences with conserved secondary structure elements
Pairwise sequence alignments
with the total set of exon and full-length sequences
with the exon and full-length sequences of older subfamilies
with the secondary structure element sequences of older subfamilies
with the total set of secondary structure element sequences
The "most similar" subfamily sequence to each exon
(scoring system using alignment score/sequence identity)
The "most similar" subfamily sequence to each secondary structure element
(scoring system using alignment score/sequence identity)
Majority-based prediction of the "most similar" subfamily to the same exon across all members of the same subfamily
(criteria for confidence score)
Majority-based prediction of the "most similar" subfamily to the same secondary structure element across all members of the same subfamily
(criteria for confidence score)
Comprehensive presentation of the results for analysis of evolutionary history and recombination of the subfamilies
Comparison with phylogenetic and GARD analyses

FIGURE S7. Phylogenetic tree with the total collection of MC proteins. The phylogenetic tree with all the MC carriers listed in Table S1 was constructed by using PhyML v3.1 from a multiple-sequence alignment with ClustalO in Seaview4 and drawn in FigTree v1.4.2 (rooted to the left). The sequence names have the same color as the subfamilies name. The main clusters of MCs for nucleotides (MC-NT1 and -NT2), carboxylates (MC-CA), positively (MC-AAP) and negatively (MC-AAN) charged amino acids are indicated with different colors.

Fig. S7.

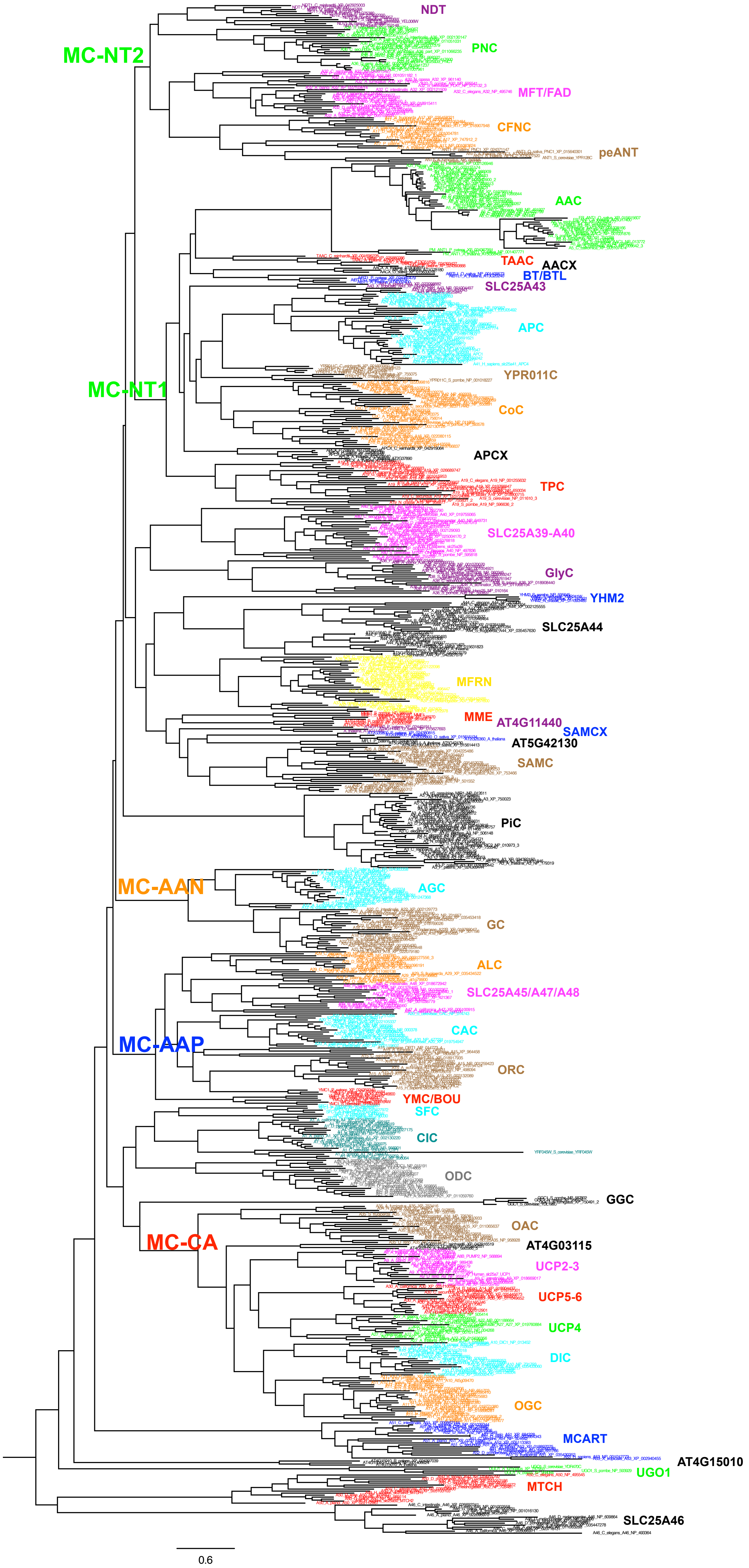
MC-NT2
NDT
PNC
MFT/FAD
CFNC
peANT
AAC
TAAC
AACX
BT/BTL
SLC25A43
APC
MC-NT1
YPR011C
CoC
APCX
TPC
SLC25A39-A40
GlyC
YHM2
SLC25A44
MFRN
MME
AT4G11440
SAMCX
AT5G42130
SAMC
PiC
MC-AAN
AGC
GC
ALC
SLC25A45/A47/A48
CAC
MC-AAP
ORC
YMC/BOU
SFC
CIC
ODC
GGC
OAC
MC-CA
AT4G03115
UCP2-3
UCP5-6
UCP4
DIC
OGC
MCART
AT4G15010
UGO1
MTCH
SLC25A46
0.6

FIGURE S8. Multiple protein sequence alignments of MC subfamily members with the exons indicated. The protein sequences of each MC subfamily (listed in Table S1) were divided based on their conserved IPs into organism kingdoms: (i) animals (including the sequences from *Homo sapiens*, *Gallus gallus*, *Xenopus tropicalis, Danio rerio*, *Caenorhabditis elegans*, *Aplysia californica*, *Ciona intestinalis, Acanthaster planci, Drosophila melanogaster*, *Bemisia tabaci*, *Spodoptera frugiperda*, *Acromyrmex echinatior*, *Cryptotermes secundus and Dendroctonus ponderosae*), (ii) plants (*Arabidopsis thaliana*, *Oryza sativa*, *Physcomitrium patens* and *Chlamydomonas reinhardtii*) and (iii) fungi (*Saccharomyces cerevisiae* [no introns in MC genes], *Schizosaccharomyces pombe*, *Neurospora crassa* and *Aspergillus fumigatus*). In the case of an MC isoform only present in *H. sapiens* of the above-mentioned genomes, the sequences of three other mammals were included: (*Bos taurus*, *Canis familiaris*, and *Mus musculus*). The divided MC subfamily sequences were aligned, and the order of the subfamily alignments presented follows the division of the main MC clusters (a, MC-NT2; b, MC-NT1; c, MC-CA; d, MC-AAP; e, MC-AAN; f, MCs outside the main clusters) and more or less in the order of the phylogenetic tree (Figure S1). The IPs are indicated in red: single residues, when the IP is found in that codon, and double residues, when it is found in between the codons. The exons within the MC transporter domains of *H. sapiens* and *A. thaliana* are numbered. Some of the sequences have been truncated in the N- and/or C-terminal outside the MC transporter domain.

a. MC-NT2 cluster subfamilies

NDT subfamily

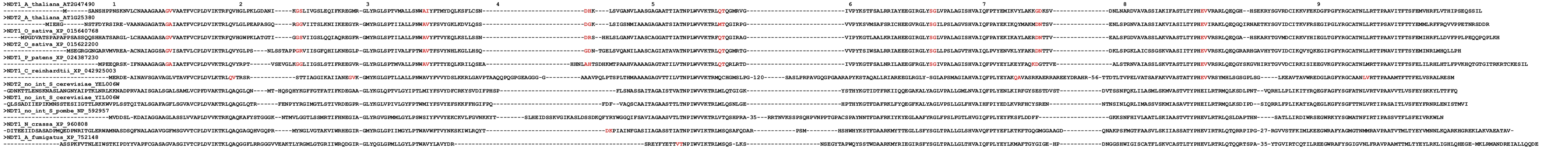

PNC subfamily

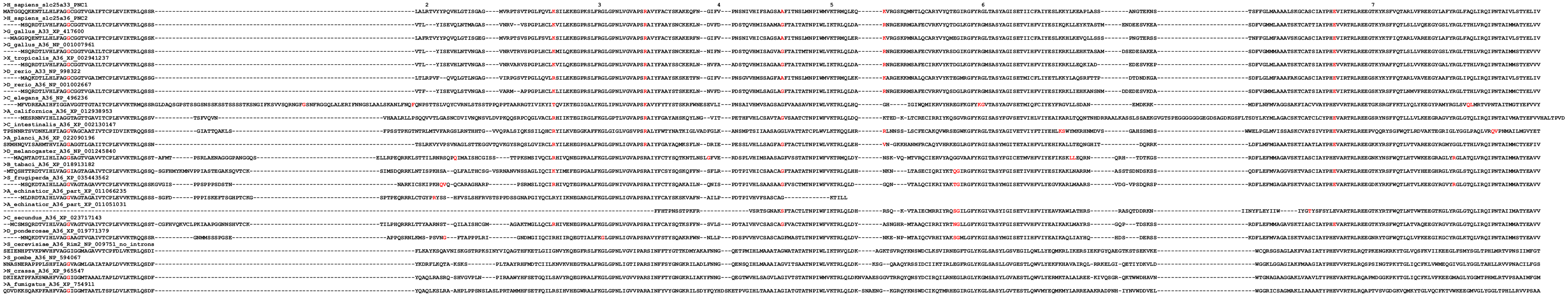

MFT/FAD subfamily

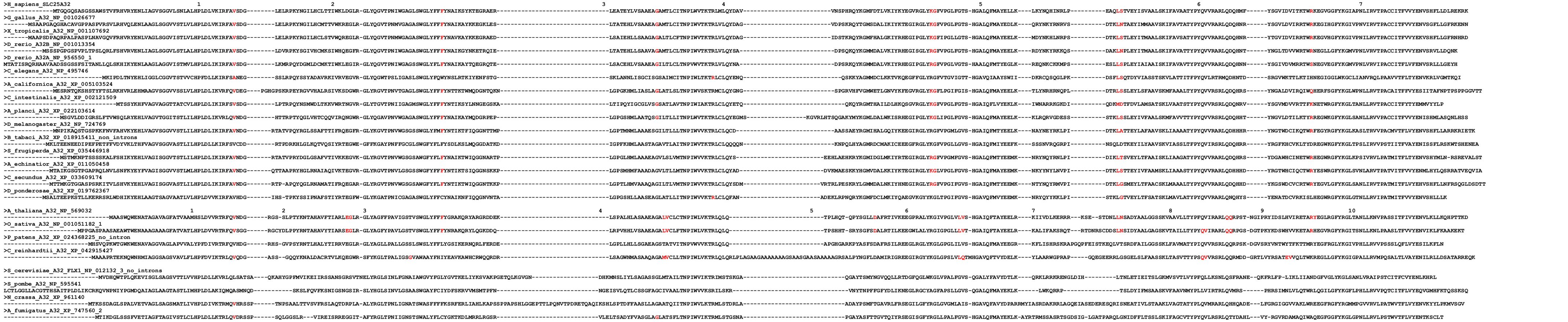

peCFCN subfamily

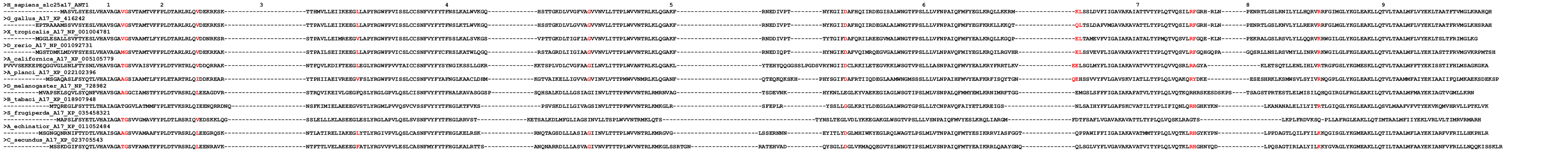

peANT subfamily

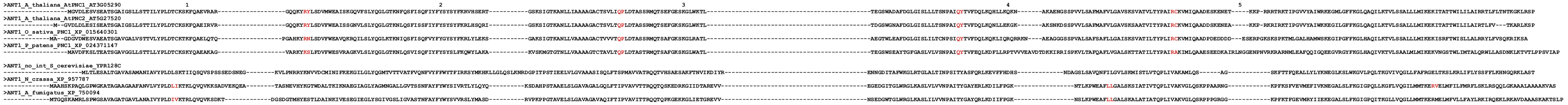

b. MC-NT1 cluster subfamilies

AAC subfamily

>H_sapiens_slc25a4_AAC1
[illegible]
>H_sapiens_slc25a5_AAC2
[illegible]
>H_sapiens_slc25a6_AAC3
[illegible]
>G_gallus_AAC4_NP_001006443
[illegible]
>G_gallus_AAC5_XP_004940900_2
[illegible]
>G_gallus_AAC6_NP_989562
[illegible]
>X_tropicalis_AAC4_NP_988909
[illegible]
>X_tropicalis_AAC5_NP_988913
[illegible]
>D_rerio_AAC4_NP_999867
[illegible]
>D_rerio_AAC5_NP_775354
[illegible]
>D_rerio_AAC6_NP_001122204
[illegible]

>C_elegans_AAC6A_NP_001022799
[illegible]
>C_elegans_AAC6B_NP_491927
[illegible]
>C_elegans_AAC6C_NP_501440
[illegible]
>C_elegans_AAC6D_NP_501727
[illegible]
>C_californica_AAC5_XP_005107474_no_introns
[illegible]
>C_intestinalis_AAC6A_XP_002131174_no_introns
[illegible]
>C_intestinalis_AAC6B_XP_002126946_no_introns
[illegible]
>A_planci_AAC5_XP_022108762
[illegible]

>D_melanogaster_AAC4_NP_727448
[illegible]
>B_tabaci_AAC6_XP_018907997
[illegible]
>S_frugiperda_AAC6_XP_035454457_no_introns
[illegible]
>A_echinatior_AAC4_XP_011066844
[illegible]
>A_echinatior_AAC6_XP_011059688
[illegible]
>C_secundus_AAC4_XP_023727041
[illegible]
>D_ponderosae_AAC5_XP_019759267
[illegible]
>D_ponderosae_AAC4_XP_019766514
[illegible]

>A_thaliana_AAC2_NP_001031876
[illegible]
>A_thaliana_AAC3_NP_001328166
[illegible]
>A_thaliana_AAC1_NP_187470
[illegible]
>O_sativa_AAC4_XP_015640040
[illegible]
>O_sativa_AAC5_XP_015625645
[illegible]
>P_patens_AAC4_XP_024394210
[illegible]
>C_reinhardtii_AAC4_XP_001695177
[illegible]

>S_cerevisiae_AAC3_NP_009642_3_no_introns
[illegible]
>S_cerevisiae_AAC2_NP_009523_no_introns
[illegible]
>S_cerevisiae_AAC1_NP_013772_no_introns
[illegible]
>S_pombe_AAC4_NP_595323_no_introns
[illegible]
>N_crassa_AAC4_XP_011393638
[illegible]
>A_fumigatus_AAC4_XP_750288
[illegible]

NEW MAMMALIAN AAC ISOFORM SLC25A31

>SLC25A31_Human_slc25a31
[illegible]
>SLC25A31_C_familiaris_XP_540952_3
[illegible]
>SLC25A31_B_taurus_NP_001039965
[illegible]
>SLC25A31_M_musculus_NP_848473_2
[illegible]

NEW PLANT AAC ISOFORM ER_ANT

>ER_ANT1_A_thaliana_AT5G17400
[illegible]
>ER_ANT1_O_sativa_XP_015615607
[illegible]

NEW PLANT AAC ISOFORM PM_ANT

>PM_ANT1_O_sativa_NP_001407771
[illegible]
>PM_ANT1_P_patens_XP_024367991
[illegible]

TAAC subfamily

>TAAC_A_thaliana_AT5G01500
[illegible]
>TAAC_A_thaliana_AT3G51870
[illegible]
>TAAC_O_sativa_XP_025880286
[illegible]
>TAAC_P_patens_XP_024390888
[illegible]
>TAAC_P_patens_XP_024393427
[illegible]
>TAAC_C_reinhardtii_XP_001696228
[illegible]

AACX subfamily

>AACX_A_thaliana_AT1G78180 1
--AQNDEIPGKDNRKRSVIGGVRRRGTMNTRKHLWAGAVAAMVSKTFLAPLERLKLEYTVRGEQ----------RNLL[illegible]
>AACX_A_thaliana_AT5G64970
--KGSVLIGGGQESKEKRRVKENGAGALNTTKHLWAGAFAAMVSRTCIAPLERMKLEYIVRGEQ----------GNLL[illegible]
>AACX_O_sativa_XP_015633018
--EEEVAEASEGKVVEEVKEARAGAGAMNMTKHLWSGAVAAMVSRTVVAPLERLKLEYIVRAEQ----------RNLF[illegible]

SLC25A43 subfamily

>H_sapiens_slc25a43 1
MATWRRDGRLTGGQRLLCAGLAGTLSLSLTAPLELATVLAQVGVVRGHARGPWATGHRVWRAEGLRALWKGNAVACLRL[illegible]
>G_gallus_A43_XP_420343
MATWRRDGRLSGPQRLCCAGLAGALSLSLTAPLELLTVLAQVGTWH-CGRGLLGAGRSLCRTEGVRALWKGNLTACLRL[illegible]
>X_tropicalis_A43_XP_002934585
MASLKRDTRLTPWQSTLCGGIAGVASRTLTAPLDVVKILSQVGTFH-TKQGFAGTFKLLCKAEGVRALWKGNLTACVRL[illegible]
>D_rerio_A43_NP_001004497
MATVKKDARLTSSQSLMCVGFAGIFSKTVTSPLEVVKILSQVGTFH-CKRGFLHSFVLICQNEGLRAFWKGNMVSCLRL[illegible]

>A_planci_A43_XP_022098882
MGGAARDHRLTYLQTFTCTTVAGVTSRSLTAPLDVIKVLSQVGTKE-TKQGFFKAFVNLYQSQGLRAFWKGNLIGCLRL[illegible]

APCX subfamily

>APCX_A_thaliana_AT3G53940
----M---EARVGVVVEGGQRALNTATTTAVHNTVVDAGNRKLLQQQPQTQQTQS--CHQHHQSNKQSLNQQQGHFGTV[illegible]
>APCX_A_thaliana_AT3G55640
MVMQT---EARVGVSSGHG---VASS------------SHR-----------------------LTQDQRSHIESA[illegible]
>APCX_A_thaliana_AT2G37890
--MNV---DARVGVAVDGAQSALNTATTVHS--SVV----------------------------MTQIKPQAKLGTF[illegible]
>APCX_O_sativa_XP_015632416
--MQT---EARVGMAAATMDSGAAAAA-----------ARRYSTQQQ-----------QQQPPPPQLHHHQPQLGTV[illegible]
>APCX_P_patens_XP_024361098
--MQPHAGEAQIAGVVGGAHIALPRAIDD----------------RPSSSNEASTPEAIEEGQVRPVVVRAPSQISTT[illegible]
>APCX_P_patens_XP_024383346
--MQPHAGDAQIAGVVGGAHRALASAIDD----------------RPNSNNEASSPGAMQEGQVRPVVVRAPSQISTA[illegible]
>APCX_C_reinhardtii_XP_042919064
[illegible]

APC subfamily

>H_sapiens_slc25a23_APC1 1 2
ENVLTGMWWKQLVAGAVAGAVSRTGTAPLDRLKVFMQVHASKTNR--------------------LNILGGLRSMVLE[illegible]
>H_sapiens_slc25a24_APC1
DEKKSGQWWRQLLAGGIAGAVSRTSTAPLDRLKIMMQVHGSKSDK--------------------MNIFGGFRQMVKE[illegible]
>H_sapiens_slc25a25_APC3
EERQTGMWWRHLVAGGGAGAVSRTCTAPLDRLKVLMQVHASRSNN--------------------MGIVGGFTQMIRE[illegible]
>H_sapiens_slc25a41_APC4
EVDNKEALWKFLLSGAMAGAVSRTGTAPLDRAKVYMQVYSSKTNF--------------------TNLLGGLQSMVQE[illegible]
>G_gallus_A24_XP_422180
EEKKSGQWWKQLLAGGVAGAVSRTGTAPLDRLKVMMQVHGSKSNK--------------------MNIASGFKQMLKE[illegible]
>G_gallus_A25_XP_004945868_2
EERQTGMWWRHLVAGGGAGAVSRTCTAPLDRLKVLMQVHASRSNN--------------------MCIIGGFTQMIRE[illegible]
>X_tropicalis_A23_NP_001135638
KEIRSGMWWKQLLAGGVAGAVSRTGTAPLDRLKVLMQVHGSQG----------------------LSILRGLRVMIEE[illegible]
>X_tropicalis_A24_NP_001011047
EEKKTGQWWKQLLAGGMAGAVSRTGTAPLDRLKVMMQVHGSKGN---------------------ANIITGLKQMVKE[illegible]
>X_tropicalis_A25_NP_001011052
EEKQTGMWWRHLVAGGGAGAVSRTCTAPLDRLKVLMQVHASRSNN--------------------MSMLGGFTQMIRE[illegible]
>D_rerio_A23B_NP_001038417
KERRSGVVWRQLVAGAMAGAVSRTGTAPLDRLKVFLQVHGTSG----------------------VTLFSGLQGMVRE[illegible]
>D_rerio_A23A_XP_005160242
KEKRSGFVWRQLMAGAVAGSVSRTGTAPLDRLKVFLQVHGQSSDK--------------------GNVWSGLRAMVKE[illegible]
>D_rerio_A24_NP_001004606
EEKTTGMWWKQLAAGGVAGAVSRTGTAPLDRMKVFMQVHSSKTNK--------------------ISLVNGFKQMIKE[illegible]
>D_rerio_A25_NP_001153492
EEKNTGMWWRHLVAGGGAGAVSRTCTAPLDRLKVLMQVHATRSNS--------------------MGIAGGFTQMIRE[illegible]
>C_elegans_A25_NP_001256365
QEMQEGIWWRHLVAGGAAGAVSRTCTAPFDRIKVYLQVNSSKTNR--------------------LGVMSCLKLLHAE[illegible]
>C_elegans_A25_NP_510081
QELLSGVWWRHLVAGGVAGAMSRTCTAPFDRIKVYLQVNSTKTNK--------------------LGVVSCVHLLHAE[illegible]
>A_californica_A25_XP_005111405
TELKTGMWWRHLVAGGAAGAVSRTCTAPLDRLKVLLQVHGSSKNN--------------------LTIKSGFKQMLEE[illegible]
>C_intestinalis_A24_XP_026691621
AEKVSGQWWRQLVAGGAAGVVSRTCTAPLDRLKVLMQVHATKSNQ--------------------LGISSGFNSMLKE[illegible]
>A_planci_A24_XP_022085201
DEKETGRWWRILLAGGAAGAVSRTVTAPLDRLKVIFQVIGSKKQN--------------------LSITSGFQHMYRE[illegible]
>D_melanogaster_A25_NP_996067
KEMQTGLWWRHLVAGGIAGAVSRTCTAPLDRIKVYLQVQTQRM----------------------GISECMHIMLNE[illegible]
>B_tabaci_A23_XP_018917803
NEMKTGMWWRHLVAGGFAGAVSRTCTAPLDRLKVYLQVHGHKSS---------------------KIRHTLQTLLQE[illegible]
>S_frugiperda_A25_XP_035437774
SELQTGKWWRHLLAGGIAGAVSRTCTAPLDRLKVFLQVNPTRE----------------------NMSGCLIRMINE[illegible]
>A_echinatior_A25_XP_011056141
GEMVSGMWWRHLVSGGIAGAVSRTCTAPLDRIKVYLQVHGTRHC---------------------NIMSCFRYMLRE[illegible]
>C_secundus_A25_XP_023727103
SEIASGQWWRHLLAGGIAGAVSRTCTAPLDRLKVYLQVHGTKQL---------------------GVKDCLKHLLQE[illegible]
>D_ponderosae_A25_XP_019772927_no_introns
IEMQTGRWWRHLAAGGVAGAVSRTCTAPLDRLKVFLQVQPSKQ----------------------RIFDCLQNMLRE[illegible]

>A_thaliana_A24_APC1_NP_568940
GISAHAQRSKLLLAGGIAGAVSRTATAPLDRLKVALQVQRTNLG---------------------VVPTIKKIWRE[illegible]
>A_thaliana_A24_APC3_NP_196349
GISKHVKRSRLLLAGGLAGAVSRTATAPLDRLKVVLQVQRAHAG---------------------VLPTIKKIWRE[illegible]
>A_thaliana_A24_APC2_NP_199918
GISKHIKRSNYFIAGGIAGAASRTATAPLDRLKVLLQIQKTDAR---------------------IREAIKLIWKQ[illegible]
>O_sativa_A23_NP_001058018
GISKSVNASKYLIAGGIAGAASRTATAPLDRLKVIMQVQTTRTT---------------------VMHSIKDIWSQ[illegible]
>O_sativa_A25_XP_015623396
RISKHASASKYLIAGGVSGATSRTATAPLDRLKVIMQVQTNRIT---------------------VLQAVKDIWRE[illegible]
>P_patens_A25_XP_024376863
GIGEHH-RLRYLAAGAMAGAVSRTATAPLDRLKVLLAIQTHSSTS--------------------SIMNGLVQIHKH[illegible]
>P_patens_A24_XP_024377247
GIDEHN-RMRFLLAGAVAGAMSRTATAPLDRLKVMLAVQTHSTTS--------------------SIMHGLTHIYQK[illegible]
>C_reinhardtii_A25_XP_042920166
VTNDPLRTYKVFLSGALSGAISRTATAPVDRLKMLLQTHDGAKG---------------------LSLRQGWQKMMAE[illegible]
>S_cerevisiae_A25_SAL1_EEU05492_no_introns
LINDFIRGFGFFIAGGISGVVSRTCTAPFDRLKVFLIARTDLSSILLNSKTDLLAKNPNADINKISSPLAKAVKSLYRQ[illegible]
>S_pombe_A24_NP_595952_no_introns
LENDPKNNVGYFISGGIAGIVSRTCTAPLDRLKVMLIS---------DTGSKPSPKYP-------FATLLHTTKVLWNR[illegible]
>N_crassa_A24_XP_956201
GLTGLLPDAGYFLAGAVSGGVSRTATAPLDRLKVFLLVNTKPKS--TTTVEAVKSGQPLSALRNAGGPIYDAIRTLWRA[illegible]
>A_fumigatus_A25_XP_755913
KLTENTPQLGYFLAGGIAGAVSRTATAPLDRLKVYLIAQTGAK---KSAAQVAKDGAPLKAAGFASRTLVGAVKELWRA[illegible]

APC NEW MAMMALIAN ISOFORM SLC25A41

>A41_H_sapiens_slc25a41_APC4 1 2
EVDNKEALWKFLLSGAMAGAVSRTGTAPLDRAKVYMQVYSSKTNFTNLLGGLQSMVQEGGFRSLWRGNGINVLKIAPEY[illegible]
>A41_B_taurus_NP_001069309
EVDNEGALWKFLLSGAMAGAVSRTGTAPLDRAKVYMQVYSSKKNFMNLLGGLRSLIQEGGIRSLWRGNGINVLKIAPEY[illegible]
>A41_C_familiaris_XP_003639787_2
EVDNEGALWKFLLSGAMAGAVSRTGTAPLDRAKVYMQVYSSKTNFMNLLGGLRSMVQEGGFHSLWRGNGINVLKIAPEY[illegible]
>A41_M_musculus_NP_780542
EEENKGTLWKFLLSGAMAGAVSRTGTAPLDRARVYMQVYSSKSNFRNLLSGLRSLVQEGGVRSLWRGNGINVLKIAPEY[illegible]

CoC subfamily

>A_thaliana_A16_NP_172908
[illegible]
>A_thaliana_A42_NP_194348
[illegible]
>O_sativa_A16_NP_001043375
[illegible]
>O_sativa_A42_XP_015623122
[illegible]
>P_patens_A16_XP_024367005
[illegible]

>S_cerevisiae_A42_Leu5p_NP_011865_no_introns
[illegible]
>S_pombe_A16_NP_593578
[illegible]
>N_crassa_A16_XP_962539
[illegible]
>A_fumigatus_A42_XP_756014
[illegible]

CoC subfamily SLC25A42

>H_sapiens_slc25a42
[illegible]
>G_gallus_A42_XP_424684
[illegible]
>X_tropicalis_A42_NP_001072712
[illegible]
>D_rerio_A42_NP_001038918
[illegible]

>C_elegans_A42_NP_492333
[illegible]
>A_californica_A42_XP_005109276
[illegible]
>A_planci_A42_XP_022099816
[illegible]

>D_melanogaster_A42_NP_650891
[illegible]
>B_tabaci_A42_XP_018910155
[illegible]
>S_frugiperda_A42_XP_035457558
[illegible]
>A_echinatior_A42_XP_011050869
[illegible]
>C_secundus_A42_XP_023717440
[illegible]
>D_ponderosae_A42_XP_019768973
[illegible]

CoC subfamily SLC25A16

>H_sapiens_slc25a16
[illegible]
>G_gallus_A16_XP_421570
[illegible]
>X_tropicalis_A16_NP_001135682
[illegible]
>D_rerio_A16_NP_991112
[illegible]

>A_californica_A16_XP_005094735
[illegible]
>C_intestinalis_A16_XP_002130726_no_introns
[illegible]
>A_planci_A16_XP_022080115
[illegible]

>B_tabaci_A16_XP_018916786
[illegible]
>S_frugiperda_A16_XP_035443559
[illegible]
>C_secundus_A16_XP_023713459
[illegible]
>D_ponderosae_A16_XP_019766837
[illegible]

YPR011 subfamily

>YPR011C_A_thaliana_ADNT1_AT4G01100
[illegible]
>YPR011C_O_sativa_ADNT1_XP_015639781
[illegible]
>YPR011C_P_patens_XP_ADNT1_024369123
[illegible]
>YPR011C_C_reinhardtii_XP_001693350_2
[illegible]

>YPR011C_no_int_S_cerevisiae
[illegible]
>YPR011C_no_int_S_pombe_NP_001018227
[illegible]
>YPR011C_N_crassa_XP_956574
[illegible]
>YPR011C_A_fumigatus_XP_755075
[illegible]

TPC subfamily

>H_sapiens_SLC25A19_NP_001119593
[illegible]
>G_gallus_A19_XP_004946425
[illegible]
>X_tropicalis_A19_NP_001015953
[illegible]
>D_rerio_A19_NP_991278
[illegible]
>C_elegans_A19_NP_001255632
[illegible]
>A_californica_A19_XP_012945487
[illegible]
>C_intestinalis_A19_XP_026687747
[illegible]
>A_planci_A19_XP_022109907
[illegible]
>D_melanogaster_A19_NP_650034_no_introns
[illegible]
>B_tabaci_A19_XP_018900715
[illegible]
>S_frugiperda_A19_XP_035444948_no_introns
[illegible]
>A_echinatior_A19_XP_011064587
[illegible]
>C_secundus_A19_XP_023724089
[illegible]
>D_ponderosae_A19_XP_019766947
[illegible]

>A_thaliana_A19_NP_566683
[illegible]
>A_thaliana_A19_NP_199708
[illegible]
>O_sativa_A19_NP_001045309
[illegible]
>P_patens_A19_XP_024398037
[illegible]

>S_cerevisiae_A19_NP_011610_3_no_introns
[illegible]
>S_pombe_A19_NP_596636_2
[illegible]
>N_crassa_A19_XP_958877
[illegible]
>A_fumigatus_A19_XP_755831_2
[illegible]

BT/BTL subfamily

>AtBT1_A_thaliana_AT4G32400
[illegible]
>AtBT1_O_sativa_XP_015643174
[illegible]
>AtBT1_P_patens_XP_024380479
[illegible]

>AtBTL1_A_thaliana_AT3G20240
[illegible]
>AtBTL1_O_sativa_NP_001409573
[illegible]

c. MC-CA cluster subfamilies

OAC subfamily

>H_sapiens_SLC25A34_NP_997231
[illegible]
>H_sapiens_SLC25A35_NP_958928
[illegible]
>G_gallus_A34_XP_015152658
[illegible]
>X_tropicalis_A34_NP_001135591
[illegible]
>D_rerio_A35_NP_001092917
[illegible]

>A_californica_A35_XP_005096117
[illegible]
>A_planci_A35_XP_022104222
[illegible]

>D_melanogaster_A34_NP_725361
[illegible]
>D_melanogaster_A34_NP_001260966
[illegible]
>D_melanogaster_A34_NP_610933
[illegible]
>B_tabaci_A35_XP_018898797
[illegible]
>S_frugiperda_A35_XP_035436167
[illegible]
>A_echinatior_A35_XP_011065637
[illegible]
>C_secundus_A35_XP_023704125
[illegible]
>D_ponderosae_A35_XP_019771649
[illegible]

>S_cerevisiae_A35_OAC1_NP_012802_no_introns
[illegible]
>S_pombe_A34_NP_593169_no_introns
[illegible]
>N_crassa_A34_XP_962817
[illegible]
>A_fumigatus_A35_XP_753416
[illegible]

AT4G03115 subfamily

>AT4G03115_A_thaliana_NP_680566_5
[illegible]
>AT4G03115_O_sativa_XP_015620551
[illegible]
>AT4G03115_C_reinhardtii_XP_042916519
[illegible]

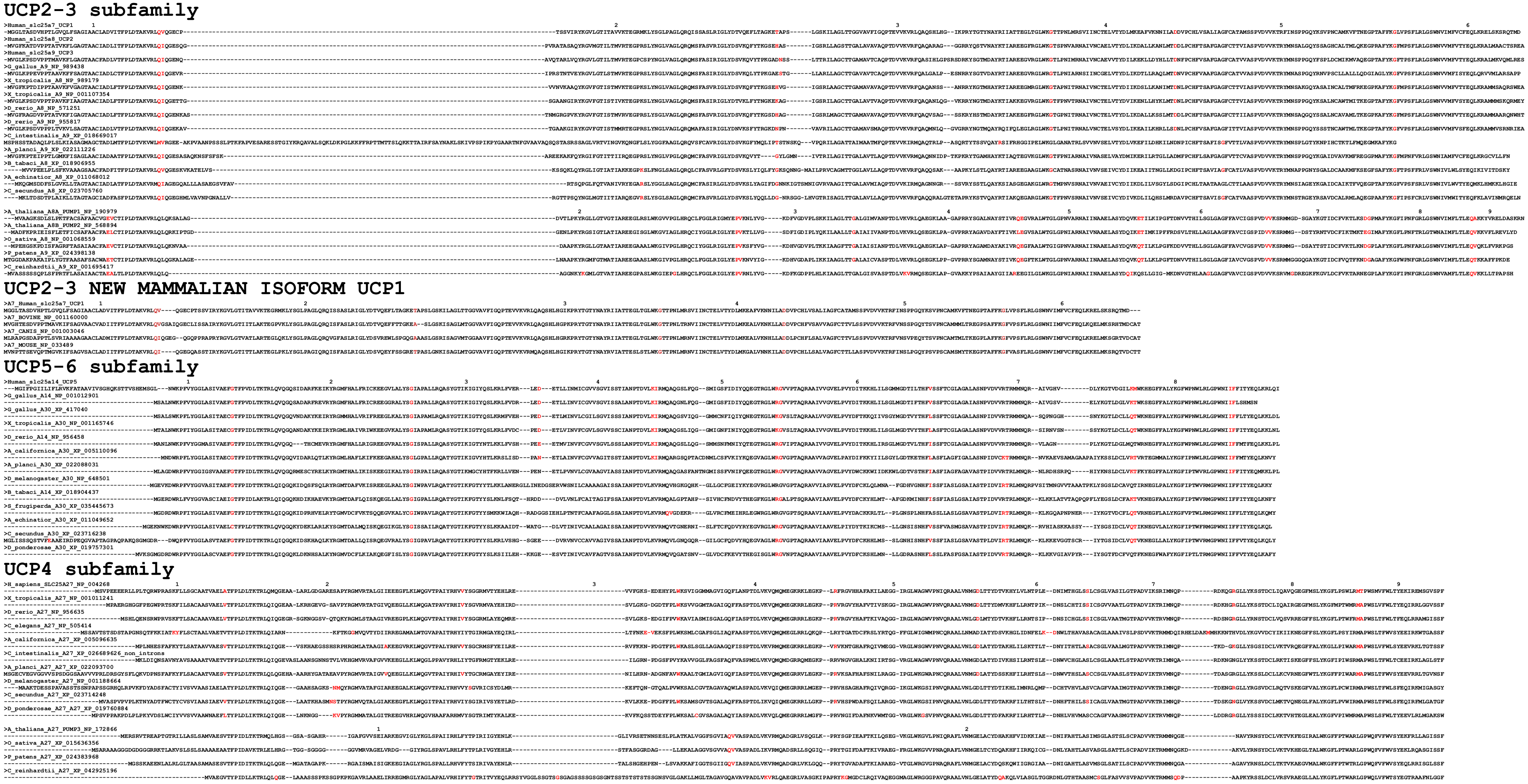
UCP2-3 subfamily
UCP2-3 NEW MAMMALIAN ISOFORM UCP1
UCP5-6 subfamily
UCP4 subfamily

DIC subfamily

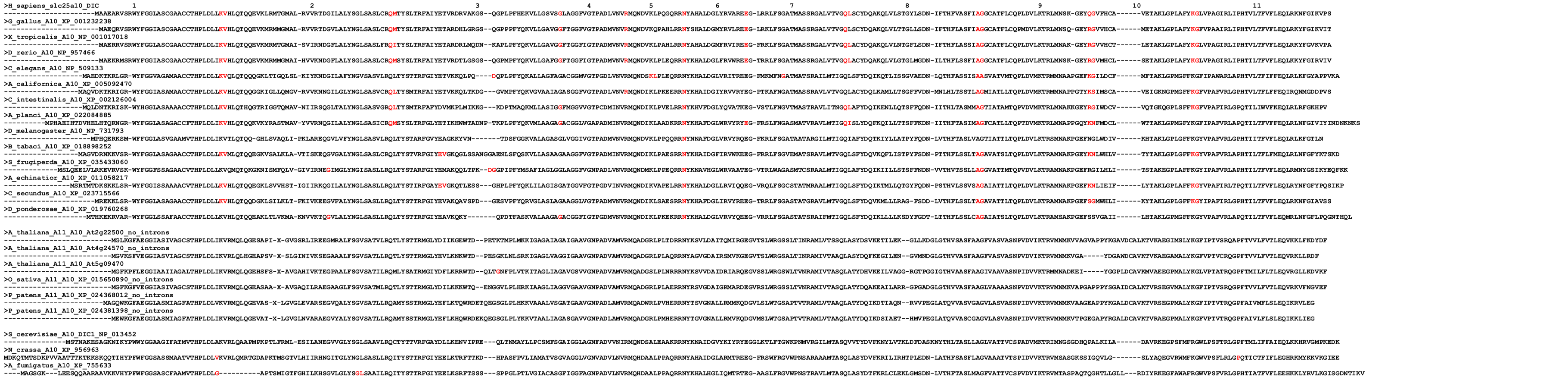

OGC subfamily

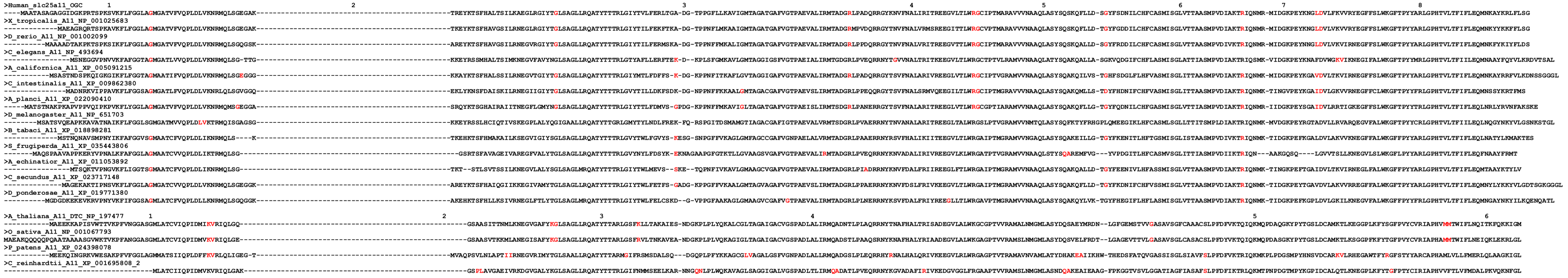

d. MC-AAP cluster subfamilies

ALC subfamily

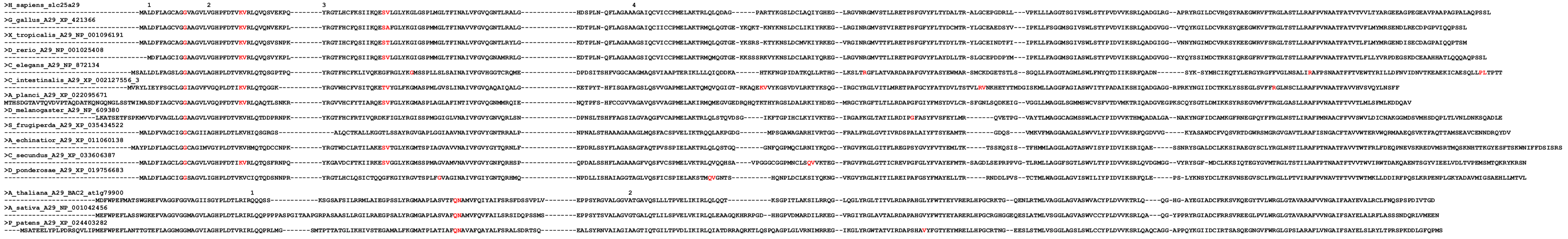

SLCA45/A47/A48 subfamily

```
>C_elegans_A45_NP_506030
---------------------------------------------MYADFIAGWAAGGAGLLVGHPLDTVKARLQTMNI---YKGIVDCMVKTMKQESVYGLYKGMFVPFISTGALHSLLFAGYGAGLKFLNP-GDS[illegible]
>A_californica_A47_XP_005100915
----------------------------------------MSETNIINDYLAGAIGGCAGVVVGHPLDTIKVQIQTQEYGGKYASMMDCIRTVQAQNLSKGYFRGLSWPLFSYGVINSVFFGTYGVALKAQGCEEIA[illegible]
>C_intestinalis_A48_XP_018672942
------------------------------------------MKLHGDAFVAGWVGGAASVLVSHPLDTVKVRLQTNSA---YRGAVHCIIKTFTREGVKGFYRGMSFPLASAAAYNALVFGVYSNTVNFLCHVRYG[illegible]
```

SLCA45/A47/A48 subfamily SLCA45/A47

```
>H_sapiens_A45_NP_872362
---------------------------------------------MPVEEFVAGWISGALGLVLGHPFDTVKVRLQTQTT---YRGIVDCMVKIYRHESLLGFFKGMSFPIASIAVVNSVLFGVYSNTLLVLTATSHQ[illegible]
>H_sapiens_A47_NP_997000_2
-----------------------------------------------MDFVAGAIGGVCGVAVGYPLDTVKVRIQTEPK---YTGIWHCVRDTYHRERVWGFYRGLSLPVCTVSLVSSVSFGTYRHCLAHICRLRYG[illegible]
>G_gallus_A47_XP_421367
-----------------------------------------------MDFIAGAIGGGLSTAVGYPLDTVKVRIQTEGH---YNGIWHCIQETYRTERVLGFYKGVSASVFTVSLISSVSFGTYRNFLCNICKLRYG[illegible]
>X_tropicalis_A45_NP_001106560
---------------------------------------------MPAAEFIAGWISGALGLVVGHPVDTVKVRLQTQSR---YRGILDCVIQTYRNETIFGFFKGMSFPVGSVAISNSLAFGSYSNALLYLSDQEIK[illegible]
>X_tropicalis_A47_XP_004917228
----------------------------------------------MADFIAGALGGACGVMVGYPLDTVKVRIQTQKN---YNGIWHCVRSTYKMERVSGFFKGVSMPMSMVSVSSSIVFGVYRNVLRNLCKLKYG[illegible]
>D_rerio_A45_XP_002662242
---------------------------------------------MPFVEFIAGWISGAVGLVVGHPLDTVKVRLQTQSV---YGGILDCVIKTYTREGLHGFFKGMSFPVLSVAVSNAVAFGSYSNALDYLTQSHHN[illegible]
>D_rerio_A47_NP_001038779
---------------------------------------------MHFADFLAGSFGGACGVAVGYPLDTVKVRIQTQKQ---FTGIWQCIVLTIRKEGVHGFFKGMFLPITTISMTSSVVFGTYRNCLQALSYIRKA[illegible]
>D_rerio_A47_NP_001083050
---------------------------------------------MHLADFLAGSVGGAFGVAVGYPLDTVKVRLQTQTG---YSGFWQCVRKTCRNEGLQGFYRGMSMPISTVSISSSLVFGTYRNILQFLHQLQHR[illegible]
```

SLCA45/A47/A48 subfamily SLC25A48

```
>H_sapiens_slc25a48
------------------------------------------MGSFQLEDFAAGWIGGAASVIVGHPLDTVKTRLQAGVG---YGNTLSCIRVVYRRESMFGFFKGMSFPLASIAVYNSVVFGVFSNTQRFLSQHRCG[illegible]
>G_gallus_A48_NP_001376493_1
------------------------------------------MGSLQLQDFAAGWVGGAASVVVGHPLDTVKTRLQAGQG---YGNTLNCILTVYRNESVAGFFKGMSFPLASIAVYSSVVFGVFSNTQRLLGQLRHG[illegible]
>X_tropicalis_A48_XP_031753840_1
MPDKAHTTRKVLTSLAEVAGVSMENIYRMETWSNPNTSVKHYSVDILASQEASFGSSNFGAASVIVGHPLDTVKARLQVGKG---YGNTFNCIFTIYKNENVAGFFKGMSFPLASIAVYNSVVFGVFSNTLSLLSQYRDK[illegible]
>D_rerio_A48_NP_001002367
------------------------------------------MTVFQLDDFLAGWIGGASSVIVGHPLDTVKTRLQAGKG---YKNTFHCVVTIYKKENVIGFFKGLSFPLASITLYNSMVFGFFSNTQRLISKYRYG[illegible]
```

CAC subfamily

```
>H_sapiens_SLC25A20_NP_000378
--------------------------------------MADQPKPISPLKNLLAGGFGGVCLVFVGHPLDTVKVRLQTQPP--SLPGQPPMYSGTFDCFRKTLFREGI-TGLYRGMAAPIIGVTPMFAVCFFGFGLGKKL[illegible]
>G_gallus_A20_XP_414400
--------------------------------------MAEQPQPISPVKNFFAGGFGGVCLVFVGHPLDTIKVRLQTQPR--PQPGQPPLYSGTFDCFRKTLTGEGV-RGLYRGMAAPIIGVTPMFAVCFFGFGLGKRL[illegible]
>X_tropicalis_A20_NP_989099
--------------------------------------MAKQPQPISPMKNFFAGGFGGICLVFAGHPLDTIKVRIQTQPK--PVPGIPPLYSGTFDCFKKTLVNEGL-RGLYKGMAAPIIGVTPMFAVCFFGFGLGKKL[illegible]
>D_rerio_A20_NP_957153
---------------------------------------MSKQQPISPMKNFFAGGFGGVCLVFAGHPLDTIKVRLQTQPK--PRPGEVAQYAGTFDCFKKTLAKEGV-RGLYKGMAAPIIGVTPMFAVCFFGFGLGKKL[illegible]
>C_elegans_A20_NP_501223
--------------------------------------------MSDVLLNFIAGGVGGSCTVIVGHPFDTVKVRIQTMPM--PKPGEKPQFTGALDCVKRTVSKEGF-FALYKGMAAPLVGVSPLFAVFFGGCAVGKWL[illegible]
>A_californica_A20_XP_005100578
----------------------------------------MPKTKPSAAKDFISGGVGGVCTVVAGHPLDTIKVRLQTMPV--PKPGEAPLFKGTFDCAMKTVRKEGF-FGLYKGMAAPITGVAPIFAICFLGFGIGKRL[illegible]
>C_intestinalis_A20_XP_002119607
----------------------------------------MGEAKGSVWKDFIAGGFGGICAVVSGHPLDTIKVRLQTMPA--PAPGQAPMFKGTWDCAVKTVRLEGF-RGLYKGMAAPIVGVSPIFALSFFGFGVGKEV[illegible]
>A_planci_A20_XP_022105337
------------------------------------------MSAEISPVKNFIAGGFGGVCLVAAGHPLDTIKVRLQTQPT--PAPGAAPIYAGTWDCAKKTVTKEGF-RGLYKGMFAPILGVTPMYAVCFLGFSIGKKL[illegible]
>D_melanogaster_A20_NP_477221
-----------------------------------MTTTENVSTERKANPVKSFLTGGFGGICNVLSGHPLDTIKVRLQTMPR--PAPGEQPLYRGTFDCAAKTIKNEGV-RGLYKGMSAPLTGVAPIFAMCFAGYALGKRL[illegible]
>B_tabaci_A20_XP_018908070
---------------------------------MSEEKKITSSSDQKVNPIKDFISGGAGGVCAVLIGHPLDTIKVRLQTMPQ--VTAGQAPLYAGTLDCATKIIKKEGP-LGLYKGMAAPLTGVAPIFALSFFGFGVGKKI[illegible]
>S_frugiperda_A20_XP_035443836_no_introns
-----------------------------------------MSENSSPIKYFLSGGFGGVCTVLAGHPMDTVKVRLQTMPL--PQSGEIAMYSGTYDCFKKTIQKEGF-RGLYKGMSAPLTGVAPIFAISFFGFGLGKKL[illegible]
>A_echinatior_A20_XP_011057778
------------------------------------------MSDTVSPIKYFLSGGFGGVCTVIAGHPLDTIKVRLQTMPV--PGPNELPLYTGTWDCAKKTITKEGI-RGLYKGMGAPLSGVAPIFAISFFGYSVGKKL[illegible]
>C_secundus_A20_XP_023722836
----------------------------------------MSEKKSVNPVKYFLSGGFGGVCTVVAGHPLDTIKVRLQTMPK--PAPGETPAYTGTWDCAKKTIKREGF-LGLYKGMGAPIAGVAPIFAISFMGFGVGKRM[illegible]
>D_ponderosae_A20_XP_019754947
------------------------------------------MSELSPLEYFICGGFGGISTILVGHPLDTIKVRLQTMPV--PKKGELPHYTSTLDCLKKTIKYEGP-KGLYKGMGVPLLGVSPIFALSFMGFGVGKTI[illegible]

>S_cerevisiae_A20_CAC_NP_014743_no_introns
-------------MSSDTSLSESSLLKEESGSLTKSRPPIKSNPVRENIKSFVAGGVGGVCAVFTGHPFDLIKVRCQNGQANSTVHAITNIIKEAKTQVKGTLFTNSV-KGFYKGVIPPLLGVTPIFAVSFWGYDVGKKL[illegible]
>N_crassa_A20_XP_962117
MSSTTSSPGAKIQQAVTDKLPHLPETKEELKAEIKAEAKAAASAGASQLRSFVAGGFGGVCAVVVGHPFDLAKVRLQT--------AEKGVYTGAIDVVKKSIAKDGLRRGLYAGVSAPLVGVTPMFAVSFWGYDLGKQI[illegible]
>A_fumigatus_A20_XP_751284
------------------MSTPGSSTPENLSDELKAIETKAVNQTIAQIRSLAAGAAGGICAVVVGHPFDLVKVRLQT--------AEKGVYSGAIDVVKKTIAREGLARGLYAGVSAPLVGVTPMFAVSFWGYDVGKTL[illegible]
```

ORC subfamily

```
>H_sapiens_slc25a15_ORC1
---------------------------------MKSNPAIQAAIDLTAGAAGGTACVLTGQPFDTMKVKMQT-------FPDLYRGLTDCCLKTYSQVG-FRGFYKGTSPALIANIAENSVLFMCYGFCQQVVRKVAG-[illegible]
>G_gallus_A15_NP_001008442
---------------------------------MRINSALQAAIDLTAGAAGGTACVVTGQPFDTAKVKMQT-------FPDMYKGIVDCFVKTYKQVG-FRGFYKGTTPALVANIAENSVLFMCYGFCQQIVRRIVG-[illegible]
>X_tropicalis_A15_NP_001090866
---------------------------------MPTNPVIQAAIDLTAGAAGGTACVLTGQPFDTAKVKMQT-------FPTMYRGLMDCAVKTYRQMG-LRGFYRGTSPALLANIAENSVLFMSYGFCQKVVRQIVG-[illegible]
>D_rerio_A15_NP_001074107
---------------------------------MAPHPVTQAVIDLSAGACGGTACVISGQPFDTAKVKMQT-------FPGLYRGFVHCFVSVYKQVG-LRGLYQGTTPALIANIAENAVLFMSYGFCQNVVRFLSG-[illegible]
>D_rerio_A15_NP_005165234
---------------------------------MAPHPVVQAVIDLSAGAIGGAACVFSGQPLDTAKVKMQT-------FPTLYRGFVDCFVSTYRQVG-LRGLYQGTTPALMANIAENSVLFMCYGFCQEVVRFVSG-[illegible]
>C_elegans_A15_NP_498094
--------------------MHESGEPTTATGDASANHFKDGIIDLLAGTAGGIANVYAGQPLDTVKVKVQT-------FPNLYSNWIVCLKDTYKLDG-IRGLYAGTLPALAANVAENAVLFTAYGYCQKTIATLNG-[illegible]
>A_californica_A15_XP_005105018
-----------------MEPPIELIEAEAGLTAKSRSTAADAVIDFAGGTMGGITSVYVGQPLDTVKVKMQT-------FPTMYRNGLDCFLKTFRQDGIARGLYAGTVPSLAANISENAVLFLFYGVCQKLVAGVMG-[illegible]
>C_intestinalis_A15_XP_002132089
-----------------------------------MNAAKELITNFVAGCAGGTACVYCGQPLDTVKVKLQT-------FPSLYKSALDCFRSTLKNEG-IQGLYKGSLPALLCNVSENAVLFVALGYMKSVIGAVVH-[illegible]
>A_planci_A15_XP_022103059
-----METSVQSKQGLQDDLKPMPGCDLRPATSSSNLAASYQAAVDFTAGALGGVACVLVGQPFDTVKVKLQT-------YPNLYPSAMRCCGQIFKKEG-IPGLFRGTLPAMAANVGETSVLFLCYGLCQKFVCRLSG-[illegible]
>D_melanogaster_A15_NP_001259423
---------------------------MHGGGTGNNINFVEGLIDFLAGSLGGAAQVYVSQPLDTVKVKLQT-------FPEAYRGMLDCFLSTYRKDGVLRGLYAGSVPAVFANVAENSVLFAAYGGCQKFVAFCVG-[illegible]
>B_tabaci_A15_XP_018917935
------MHDSGLSTRHGAGSSDSSGNDVSNSSEARKHSETRIHAVNFLAGTIGGAATVLVGQPLDTVKVKMQT-------SPTEFKGMTDCLKKIYRFEG-LRGLYAGSMPAVTANVAENSVLFLCYGAVQRSVAYCCG-[illegible]
>S_frugiperda_A15_XP_035431892
----------------------------MAHSSGSAGNLKNGIIDFTGGSLGGVAVVYVGQPLDTVKVKMQT-------FPHLYKGMYDCLKQTLRNDGVFRGLYAGTTPAIMANVAENSVLFAAYGYCQKFVCRLSG-[illegible]
>A_echinatior_A15_XP_011051191
MVTTKEFFTRTMTTMTTDSVAGLIDTEVSHVPMENCRGSIKEGAIDFVAGSLGGIALVYVGQPLDTVKVKMQT-------FPSMYKNMVNCFLRTLRTDGIARGLYAGTIPAIVANVAENSVLFAAYGGCQKVITHLSG-[illegible]
>C_secundus_A15_XP_023718668
-----------------------------MGPEGSSGSIKDAAIDFSAGSLGGVGLVYVGQPLDTIKVKMQT-------FPNLYRGMVDCFRQTLMKEGITKGLYAGTLPAVVANVAENSVLFAAYGTCQKAMMMMTR-[illegible]
>D_ponderosae_A15_XP_019754124
----------------MALNNNTNIENIKSNNHPGRSHVKDGVIDFSAGCLGGIALVYVGQPLDTVKVKMQT-------SPLLYTNMVDCCRNTYLQDGIIKGLYAGTTPALVTNIMENSVLFLCYGFCQKVIVNITG-[illegible]

>A_thaliana_A15_BAC1_NP_180938_2
----------------------------MGESKTTTGEGFGFYKEYVAGMMAGLATVAVGHPFDTVKVKLQKHNTDV--QGLRYKNGLHCASRILQTEGVK-GLYRGATSSFMGMAFESSLMFGIYSQAKLFLRGTLP-[illegible]
>C_reinhardtii_A15_XP_042915245
-------------------------MHGSSQEALSPAASAATRELFAGFAAGAANVTSGYPFDTVKVRLQSAPPG------AYRGALDCAVKVVRYEGLRRGLFRGLSSPLVGGTLETGVNYLVYSRVLAMLASSAPS[illegible]

>S_cerevisiae_A15_ORT1_NP_014773_4_no_introns
----------------------------MEDSKKKGLIEGAILDIINGSIAGACGKVIEFPFDTVKVRLQTQASN------VFPTTWSCIKFTYQNEGIARGFFQGIASPLVGACLENATLFVSYNQCSKFLEK----[illegible]
>N_crassa_A15_XP_964458
-MDSVPAQTHQGFKEAGAASASHLTTTTSLPTKVESRTAVMEALEDIVYGSAAGIVGKYIEYPFDTVKVRLQSQPDH---LPLRYTGPLDCFRQSIRADGFL-GLYRGISAPLVGAALENSSLFFFERIGRSLLYSSGF-[illegible]
>A_fumigatus_A15_XP_746931
-------------MAAAEHVLAIDNEKSMELPVLPPNQGFEAFKDIIFGSAAGMAGKVIEYPFDTVKVRLQSQPSH---IPLRYQGPLDCFRQSIQADGLR-GLYRGISAPLAGAAIENSCLFFSYRIIQDILRATCY-[illegible]
```

YMC/BOU subfamily

```
>YMC1_A_thaliana_BOU_AT5G46800
------------------------------MADAWKDLASGTVGGAAQLVVGHPFDTIKVKLQSQPTPAPGQLPRYTGAIDAVKQTVAS[illegible]
>YMC1_O_sativa_XP_015614184
------------------------------MGDVVKDLVAGTVGGAANLIVGHPFDTIKVKLQSQPTPAPGQFPKYAGAVDAVKQTIAT[illegible]
>YMC1_P_patens_XP_024359340
-----------------------------MSDAVAKDLTAGTFAGVAQLLVGHPFDTIKVKLQSQPVPLPGQPPKYSGAIDAVKQTLAS[illegible]

>YMC1_no_int_S_cerevisiae_YPR058W
MSEEFPSPQLIDDLEEHPQ---------HDNARVVKDLLAGTAGGIAQVLVGQPFDTTKVRLQTSS--------TPTTAMEVVRKLLAN[illegible]
>YMC2_no_int_S_cerevisiae_YBR104W
MSEEFPTPQLLDELEDQQKVTTPNEKRELSSNRVLKDIFAGTIGGIAQVLVGQPFDTTKVRLQTAT--------TRTTTLEVLRNLVKN[illegible]
>YMC1_S_pombe_NP_593701_2
---------------------MEPVIPEGALSQSTKDFLAGVSGGVAQVLVGQPFDCVKVRLQSQSNV----SPIYNNALDCVKKISKN[illegible]
>YMC1_N_crassa_XP_959913
-----MA-ASAADV--------DSHDGQNAGLQTAKDLFSGAVGGIAQVLIGQPFDIVKVRLQTST--------QYSSALTAAASIYKH[illegible]
>YMC1_A_fumigatus_XP_750402
-----MESEAFEDM--QKG---------GGALRTIKDLAAGAAGGIAQVLLDI----VKVRLQTTS--------EYKSALDCATRIFKN[illegible]
```

e. MC-AAN cluster subfamilies

AGC subfamily

```
>H_sapiens_slc25a12_AGC1
WLQIAESAYRFTLGSVAGAVGATAVYPIDLVKTRMQNQRGSGSVVGELMYKNSFDCFKKVLRYEGFFGLYRGLIPQLIGVAPEKAIKLT[illegible]
>H_sapiens_slc25a13_AGC2
LLQVAESAYRFGLGSVAGAVGATAVYPIDLVKTRMQNQRSTGSFVGELMYKNSFDCFKKVLRYEGFFGLYRGLLPQLLGVAPEKAIKLT[illegible]
>G_gallus_A12_XP_428938
WLQVAESAYRFTLGSIAGAVGATAVYPIDLVKTRMQNQRSTGSVVGELMYKNSFDCFKKVLRFEGFFGLYRGLLPQLIGVAPEKAIKLT[illegible]
>G_gallus_A13_NP_001012967
LIQIAESAYRFALGSVAGAVGATAVYPIDLVKTRMQNQRSTGSFVGELMYKNSFDCFKKVLRYEGFFGLYRGLLPQLLGVAPEKAIKLT[illegible]
>X_tropicalis_A13_NP_001107969
LVQVAESAYRFALGSIAGAVGATAVYPIDLVKTRMQNQRSTGSFVGELMYKNSFDCFKKVLRYEGFFGLYRGLLPQLLGVAPEKAIKLT[illegible]
>D_rerio_A12_NP_997947
WLQAAESAYRFTLGSIAGATGATAVYPIDLVKTRMQNQRSTGSFVGELMYKNSFDCAKKVLRYEGFFGFYRGLLPQLIGVAPEKAIKLT[illegible]
>C_elegans_A12_NP_497274
GTAFLESAYRFLLGSVAGACGATAVYPIDLVKTRMQNQRTSGSFVGEVMYKNSLDCFKKVVKFEGLLGLYRGLLPQIVGVAPEKAIKLT[illegible]
>A_californica_A13_XP_005095774_2
VMQIAESAYRFSLGALAGATGATAVYPIDLVKTRLQNQR-TGSYVGELMYKNSFDCAKKVIRHEGVAGLYRGLAPQLVGVAPEKAIKLT[illegible]
>C_intestinalis_A12_XP_009862417
YMAVLENAYRFSLGVIAGGVGATAVYPIDLVKTRLQNQRSTGSYVGELMYRNSFDCFFKVLRHEGFQGLYRGLIPQLVGVGPEKAIKLT[illegible]
>A_planci_A12_XP_022087095
LIQVAESIYRFCLGAIAGAVGATAVYPIDLVKTRMQNQR--GGLVGELMYENSLDCFTKVVRHEGFFGLYRGLPPQLLGVAPEKAIKLT[illegible]
>D_melanogaster_A12_NP_001247368
FIQVLESSYRFTLGSFAGAVGATVVYPIDLVKTRMQNQR-AGSYIGEVAYRNSWDCFKKVVRHEGFMGLYRGLLPQLMGVAPEKAIKLT[illegible]
>B_tabaci_A13_XP_018913363
GIQMLESLYRFSLGSVAGAIGATAVYPIDLVKTRMQNQR-TGSFIGELMYRSSWDCFKKVIRHEGVFGLYRGLLPQIMGVAPEKATKLT[illegible]
>S_frugiperda_A13_XP_035438281
LIQILESTYRFTLGSVAGAVGASAVYPIDLVKTRMQNQR-TGSFIGEVAYRNSWDCFKKVIRHEGIFGLYRGLVPQLIGVAPEKAIKLT[illegible]
>A_echinatior_A12_XP_011053164
IVQLLESGYRFVLGSIGGAVGATAVYPIDLVKTRMQNQR-TGSFIGELMYRNSFDCCKKVIRHEGFFGLYRGLMPQLMGVAPEKAIKLT[illegible]
>C_secundus_A12_XP_023712752
LIQILESAYRFTLGSIGGAVGATAVYPIDLVKTRMQNQR-TGSFIGELMYRNSFDCCKKVIRHEGFFGLYRGLVPQLMGVAPEKAIKLT[illegible]
>D_ponderosae_A12_XP_019768579
LIQILESAYRFTLGSIAGAVGATAVYPIDLVKTRMQNQR-TGSFIGELMYKNSFDCFKKVIRHEGIFGLYRGLVPQLLGVAPEKAIKLT[illegible]

>P_patens_A12_XP_024383358
SRFLARVAQHLGGGSVAGAVGATVVYPLDTIKTRMQAQNLDG---DEPQYKDEIDCFKQLVTKEGPASLYSGLVPQLLGIAPEKAMKLT[illegible]

>S_cerevisiae_A13_NP_015346_no_introns
FYPIFDSLYNFSLGSIAGCIGATVVYPIDFIKTRMQAQR------SLAQYKNSIDCLLKIISREGIKGLYSGLGPQLIGVAPEKAIKLT[illegible]
>N_crassa_A12_XP_961607
AHRAGESAYNFILGSLSGAFGAFMVYPIDLVKTRMQNQR--GASPGSRLYDNSIDCFRKVIRNEGFRGLYSGVLPQLVGVAPEKAIKLT[illegible]
>A_fumigatus_A12_KAH1350362
LHSVLESVHHFALGSLAGAFGAFMVYPIDLVKTRMQNQR-STRVGERLYNNSLDCFRKVIRNEGFLGLYSGVLPQLIGVAPEKAIKLT[illegible]
```

GC subfamily

```
>H_sapiens_slc25a18_GC2
----------------MTHQDLSITAKLINGGVAGLVGVTCVFPIDLAKTRLQNQH---GKAMYKGMIDCLMKTARAEG------FF[illegible]
>H_sapiens_slc25a22_GC1
----------------MADKQISLPAKLINGGIAGLIGVTCVFPIDLAKTRLQNQQN---GQRVYTSMSDCLIKTVRSEG------YF[illegible]
>G_gallus_A22_NP_001238997
----------------MADKQISLPAKLINGGIAGLIGVTCVFPIDLAKTRLQNQQN---GQRMYTSMSDCLIKTIRSEG------YF[illegible]
>X_tropicalis_A18_NP_001096428
-----------------MAEKISLPAKLINGGIAGLVGVTCVFPIDLAKTRLQNQQ----GSGIYRGMFDCLIKTVRSEG------FF[illegible]
>X_tropicalis_A22_NP_001005430
----------------MAEKQISLPAKLINGGVAGIIGVTCVFPIDLAKTRLQNQRN---GQQIYKSMWDCLRKTLRSDG------YF[illegible]
>D_rerio_A18_XP_005174420
----------------MAEKKLSLPAKLINGGVAGLVGVTCVFPIDLAKTRLQNQQ----GARVYSGMLDCLAKTIKMEG------YF[illegible]
>D_rerio_A22b_NP_001003448
----------------MTQQPISLPAKLINGGVAGLVGVTCVFPIDLAKTRLQNQRG---SQRVYKNMMDCLIKTVRSEG------YF[illegible]
>D_rerio_A22a_NP_998573
----------------MSQQ-ISLPAKLINGGIAGIVGVTCVFPIDLAKTRLQNQRQ---GQQVYKSMIDCLIKTVRTEG------YF[illegible]
>C_elegans_A18_NP_510493
---------------MGGDNNFSYGPKIMNGGLAGIIGVSCVFPIDLVKTRLQNQTVGADGKLQYSGIADCAKQTWRSGGASTLAKFR[illegible]
>C_elegans_A22_NP_501198
-----MDIDELELLQEEREEQPIRYLPKVLNGGISGIVGVSCVFPMDLVKTRLQNQ----KGTSKYTGIADCFKKSWQAGAPGRLNQIK[illegible]
>A_californica_A22_XP_012935169
-----------------MDNKFSLLPRVINGSIAGIVGVTCVFPIDLVKTRLQNQQPGPQGQLLYKSLPDCALKTFRNEG------FF[illegible]
>C_intestinalis_A22_XP_002129773
-----MCDMEMEFQARDNLMKYQTLIATTGNGCIAGFAGIVATFPLDLCKTRLQNQPVQN-GVRMYNGLWHCIIEVQRKEG------LR[illegible]
>A_planci_A18_XP_022079180
------------------MGDISLPAKCINGALAGVTGVTCIFPIDLVKTRLQNQQVI-DGRRMYKNLLDCFIKTAKADG------IK[illegible]
>D_melanogaster_A22_NP_731657
-----MLEQVEQKNQEQKKPQKFNVFPKIINGGVAGIIGVACVYPLDMVKTRLQNQTIGPNGERMYTSIADCFRKTIASEG------YF[illegible]
>D_melanogaster_A18_NP_001262490
----MSSSATIATPLPQPQHQQFALLPKIINGGIAGIIGVTCVFPLDLVKTRLQNQQIGPNGERMYNSMFDCFRKTYKAEG------YF[illegible]
>B_tabaci_A22_XP_018900680
---MKAAELNSSVDPPKQPPAKFSLVPKIVNGGIAGIIGVTVVFPLDLVKTRLQNQQTGPNGEKMYSSMLDCFRKTYKAEG------YF[illegible]
>S_frugiperda_A22A_XP_035453423
-----------MAAPKPPPAQQFNLIPKIVNGGIAGIVGVSIVFPLDLVKTRLQNQTIGPNGERQYKNMLDCFKQTYKAEG------YF[illegible]
>S_frugiperda_A22B_XP_035453418
-------MAAKKGPEQKSLLPQLNILPKVVNGGIASIVGICLVFPLDLVKTRLQNQTIGPNGEKQYKNMLDCFKQTYRAEG------YF[illegible]
>A_echinatior_A22_XP_011067792
---------MAQDSSIPKPAEQFKLIPKIINGGIAGIIGVSVVFPLDLVKTRLQNQVIGPNGEQMYKSMFDCFKKTYKAEG------YF[illegible]
>C_secundus_A18_XP_023706553
--------------MGEKGDRQFKLLPKIINGGIAGIIGVSCVFPLDLVKTRLQNQQIGPNGERQYRSMLDCFRKTYRAEG------YF[illegible]
>D_ponderosae_A22A_XP_019769026
---------------MAEKQSSFSLVPKIVNGGIAGIIGVTCVFPLDLVKTRLQNQQIGPNGEKMYNNMIDAFKKVYKQEG------FL[illegible]
>D_ponderosae_A22B_XP_019769047
---------------MAENQTKFTLLPKILNGGIAGIVGVACTFPLDLVKTRLQKQTVGPGGRKLYNSIFDAFSKIYVSEG------LR[illegible]
```

f. MC subfamilies outside the main clusters

SFC subfamily

>SFC1_A_thaliana_AT5G01340
[illegible]
>SFC1_O_sativa_XP_015627972
[illegible]
>SFC1_P_patens_XP_024387979
[illegible]

>SFC1_no_int_S_cerevisiae_YJR095W
[illegible]
>SFC1_no_int_C_reinhardtii_XP_042923487
[illegible]
>SFC1_N_crassa_XP_962756_2
[illegible]
>SFC1_A_fumigatus_XP_756030
[illegible]

CIC subfamily

>H_sapiens_CIC_SLC25A1_NP_005975
[illegible]
>G_gallus_CIC_XP_415059
[illegible]
>X_tropicalis_CIC_NP_989391
[illegible]
>D_rerio_CIC_NP_956901
[illegible]
>D_rerio_CIC_NP_001212457_1
[illegible]
>C_elegans_CIC_NP_499187
[illegible]
>A_californica_CIC_XP_012940275
[illegible]
>C_intestinalis_CIC_XP_002130220
[illegible]
>A_planci_CIC_XP_022105121
[illegible]
>D_melanogaster_CIC_NP_001027175
[illegible]
>B_tabaci_CIC_XP_018916557
[illegible]
>S_frugiperda_CIC_XP_035448019
[illegible]
>A_echinatior_CIC_XP_011067395
[illegible]
>C_secundus_CIC_XP_023713316
[illegible]
>D_ponderosae_CIC_XP_019768401
[illegible]

>S_cerevisiae_CIC_NP_009850_no_introns
[illegible]
>S_pombe_CIC_NP_594420
[illegible]
>N_crassa_CIC_XP_956064
[illegible]
>A_fumigatus_CIC_XP_755059_2
[illegible]

ODC subfamily

>H_sapiens_SLC25A21_NP_085134
[illegible]
>G_gallus_A21_XP_015143145_1
[illegible]
>X_tropicalis_A21_NP_001017069
[illegible]
>D_rerio_A21_NP_001070100
[illegible]
>C_elegans_A21_NP_510638
[illegible]
>A_californica_A21_XP_005108207
[illegible]
>C_intestinalis_A21_XP_002130378
[illegible]
>A_planci_A21_XP_022087672
[illegible]
>D_melanogaster_A21_NP_569856
[illegible]
>B_tabaci_A21_XP_018901660
[illegible]
>S_frugiperda_A21_XP_035437831
[illegible]
>A_echinatior_A21_XP_011059760
[illegible]
>C_secundus_A21_XP_023706270
[illegible]
>D_ponderosae_A21_XP_019757086
[illegible]

>S_cerevisiae_A21_ODC2_NP_014865_no_introns
[illegible]
>S_cerevisiae_A21_ODC1_NP_015191_no_introns
[illegible]
>S_pombe_A21_NP_594211
[illegible]
>N_crassa_A21_XP_960396_3
[illegible]
>A_fumigatus_A21_XP_752333
[illegible]

SLC25A39-A40 subfamily

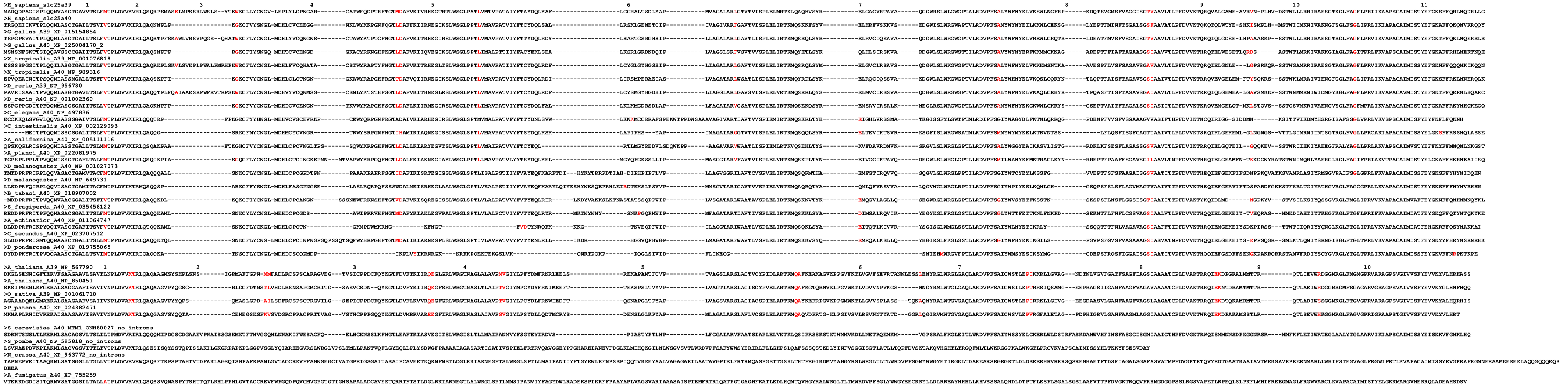

GlyC subfamily

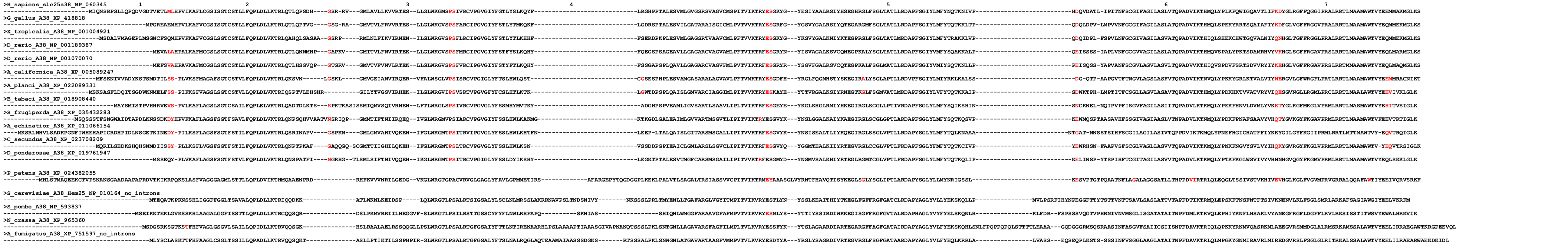

SLC25A44 subfamily

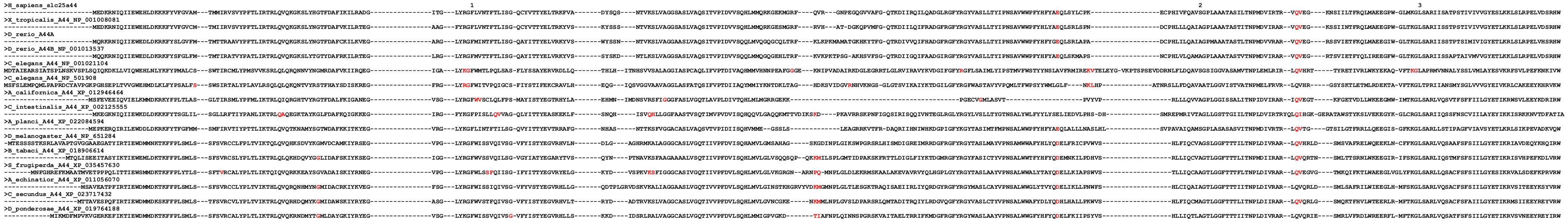

MFRN subfamily

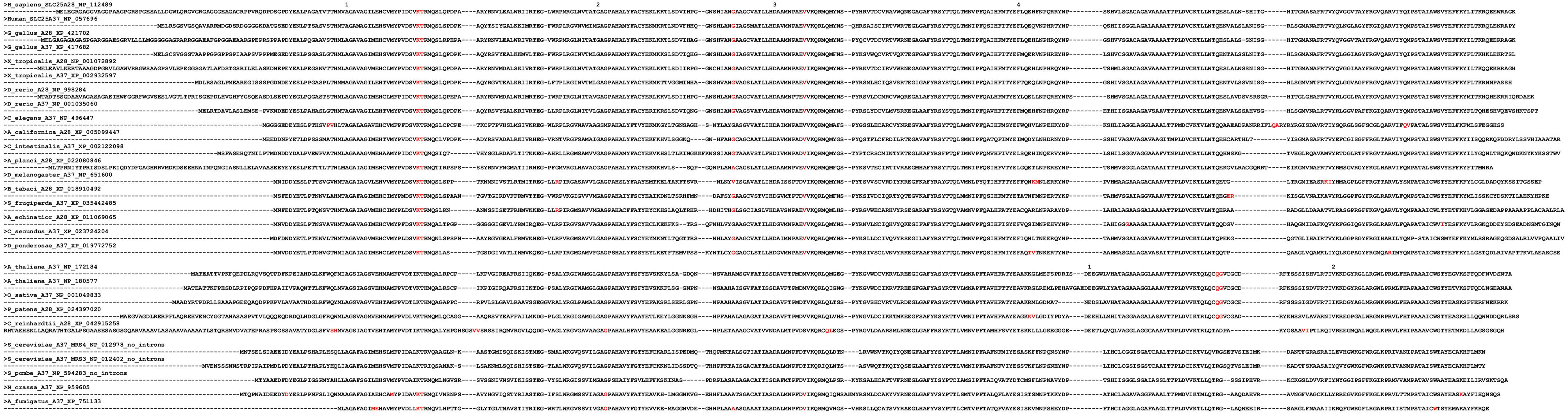

SAMC subfamily

```
>H_sapiens_slc25a26_SAMC                    1                    2                    3                    4                    5                    6                    7                    8                    9                    10
------------------------------------MDRPGFVAALVAGGVAGVSVDLILFPLDTIKTRLQSPQGFSKAGG---------FHGIYAGVPSAAIGSFPNAAAFFITYEYVKW--------FLH-----------------------ADSSSYLTPMKHMLAASAGEVVACLIRVPSEVVKQRAQ---VSASTRTFQIFSNILYEEG-------------IQGLYRGYKSTVLREIPFSLVQFPLWESLKALWSWR-QDH--------------VVDSWQSAVCGAFAGGFAAAVTTPLDVAKTRITLAKAGSSTADG----NVLSVLHGVWRS------QGLAGLFAGVFPRMAAISLGGFIFLGAYDRTHSLLLEVGRKSP
>G_gallus_A26_NP_001264772
------------------------------------MEPRESWAALAAGGIAGVFVDLILFPLDTVKTRLQSPQGFRKAGG---------FRGIYAGVPSTAIGSFPNAAAFFITYENVKS--------VLH-----------------------HDSASYLTPVTHMVAASLGEVVACLIRVPSEVVKQRAQ---VSPSAGTFRILSHTLYHEG-------------IQGLYRGYKSTVLREIPFSLVQFPLWEFLKDLWSWK-QGH--------------VVDSWQSAVCGAFAGGFAAAVTTPLDVAKTRIMLAKAGSTTASG----NVLAALGGIWRT------QGLSGLFAGVVPRMTAISLGGFIFLGTYEKTRQLLL
>X_tropicalis_A26_NP_001004820
------------------------------------MERREFCASLLAGGTAGMCVDLILFPLDTIKTRLQSPLGFSKSGG---------FRGIYAGVPSTAVGSFPNAAAFFVTYESAKQ--------LLR-----------------------SDS-SYLSPIIHMAAASLGEVVACLIRVPSEVIKQRAQ---VSPSSTTYQMLSATLRQEG-------------IKGLYRGYKSTVLREIPFSLVQFPLWESLKDLWSWK-QGR--------------AVDSWQSAVCGAFAGGFAAALTTPLDVAKTRIMLAKAGSGVASG----NVLFALHEIWRT------QGIMGLFAGVIPRMTAISLGGFIFLGAYDKVRTLMLREQ
>D_rerio_A26_NP_001025314
------------------------------------MDRREFTASLVAGGCAGMCVDLTLFPLDTIKTRLQSQQGFYKAGG---------FRGIYAGVPSAAIGSFPNAAAFFVTYESTKS--------VFS-----------------------GYTTTNLAPITHMLAASLGEIVACLIRVPTEVVKQRTQ---ANPSISTYRVLLNSLQEEG-------------FRGLYRGYGSTVLREIPFSLVQFPLWEYLKAVWWRR-QGG--------------RLDSWQAAVCGALAGGVAAFVTTPLDVAKTWIMLAKAGTSTASG----NIPMVLCEVWRS------RGIPGLFAGSIPRVMFISMGGFIFLGAYEKVRRTLL
>C_elegans_A26_NP_501552
----------------------------------MPSEEGSVVRWLVCGATAGLAVDIGLYPLDTIKSRMQSKQGFIAAGG---------FKDIYRGMISVLVGSAPGAAIFFLTYKYING--------QMKQ------------------------VIEERNALVDAVSASLAEIAACAVRVPTELCKQRGQ---VNKNERLTLICKEIMETKG-------------IRGFYRGYGSTVAREIPFSIIQFPIWEALKRAVANKKESG--------------RCSPLEGAACGSVAGFIAAGLTTPLDVAKTRIMLTKNGPAPG-------ILSTLKEVYTS------NGVRGLYSGVVPRVMWISGGGFVFFGAYETAMHFTKFLD
>A_californica_A26_XP_005093221_no_introns
-------------------------MEDVIDPDIVENTDFRVALAAGGAAGTSVDIILFPLDTIKTRLQSERGFWKSGG---------FRGIYSGLFSAAVGSAPTAALFFVAYESTKT--------TFNN-----------------------ITQDRRYQSFGHMMAASIGEVSACLIRVPVEVVKQRTQ---ALGTGSSIASFKKTLNSEG-------------LGGFYRGYFSTVLREVPFSIIQFPLWEYLKSRVSKHTEQ---------------PITALQSSLCGATSGAISAAITTPLDVVKTRVMLAEKGSSIAKG----SISFVVQTIIKE------KGFMGLYAGIAPRVMWISIGGSVFLGVYEKVKITLTQMFKV
>C_intestinalis_A26_XP_004225486_no_introns
-------------------------------------MVNEWMTALASGAVAGFCVDVTLFPVDTVKTRLQSSQGFIKAGG---------FKRIYSGIGPAALGSAPGAAVFFTAYDTTRN--------LLKK-----------------------PDRSFGHNLAVDMTAASVGEVVACLIRVPVEVIKQRSQ---VTKNANSFRIFLSCIRTEG-------------VRGMYRGYRSTVVREIPFSLIQFPLWEFLKVTVAKY-RGKE-------------SPDAIESAVCGFFSGGIAAAVTTPLDVAKTRIILAQTNDSAAAG----NILPVLREVWST------GGARALFAGVLPRVVWISVGGFVFLGMYDTTKSLLNGL
>A_planci_A26_XP_022085246_no_introns
---------------------------------MSEQTPPLLVALVSGGLAGTAVDASLFPLDTIKTRLQSSDGFLKAGG---------FRGIYAGLGPAVLGSAPTAGVFFCTYELTKH--------ISSSL----------------------GSAGNYYEPITHMMAASLGEVVACLIRVPVEVVKQRAQ---ASRHASTS-ILRSVLREEG-------------VRGLYRGYLSTVMREIPFSIIQFPLWEILKVRWGQW-QGR--------------PVDAWQSSICGAVAGGISAGVTTPLDVAKTRIILAAKDSKEAKG----NIPGVLKDIMRT------RGVRGLFAGVVPRVLWISIGGAIFLGVYEKAKISLLKLNSPDISS
>D_melanogaster_A26_NP_651415
--------------MAAELGLESAAGSVAIKMQEPVNKLKFFHALVAGGVAGMVVDIALFPIDTVKTRLQSELGFWRAGG---------FRGIYKGLAPAAAGSAPTAALFFCTYECGKQ--------FLSS-----------------------VTQTKDSPYVHMAAASAAEVLACLIRVPVEIAKQRSQT-LQGNKQSGLQILLRAYRTEGL-------------KRGLYRGFGSTIMREIPFSLIQFPLWEYFKLQWTP--LTGF--------------DSTPFSVALCGAVAGGISAGLTTPLDVVKTRIMLAER---ESLN-RRRSARRILHGIYLE------RGFSGLFAGFVPRVLWITLGGAFFFGFYDLTTRILGATSTDH
>B_tabaci_A26_XP_018906253
---------------------MEIEASSTHEAVSQRTTENLTQSIVGGAAAGVVVDTILFPLDTLKTRLQSQYGFWNSGG---------FKSIYKGFAPAILASPFSAAAFFATYNGMKD--------LITP-----------------------HCSQELLPFVHSTSASFAEVSCCCIKVPAEIVKQRRQ--ALLNAKSPLAIMKDTIRQEG-------------MRGIYRGYFSTLFRDIPFSIIQFPVWEYSMKRWKI--FKGR--------------PINSAEVAACGAFSGAVASAITTPLDVAKTQIMLAEAGSKKALN---TNIITVLIDIRKE------HGFKKLFAGFLPRIFSIMLGGGIFFGVYDKTLRIIGFER
>S_frugiperda_A26_XP_035452078
--------------------------MERKGKSKSFKSTHFPPLVAGAIAGVSVDVTLYPLDTLKTRLQSAQGFQKAGG---------FSGVYRGLLTVAATSMPTTALFFVSYEATKT--------LCQP-----------------------LVATQYTPMVHMFAASVGEVLACLIRVPTEIAKQRKQTYVGTNSKSSVSILLHAYKTEGF-------------FRGVYRGFGSTVLRDLPFSFIELPVWEMLKTTVRK--YNNG--------------EISSFQSAICGSIAGGFAAVLTTPLDLAKTRIMLSDG---YRVS-GKMRIRPVLTSIYIE------SGFKGLFAGVMPRMTAIMIGGFVFFGVYEDVKKYVEKYFDRR
>A_echinatior_A26_XP_011064681
----------------------MFHEQKESAPNLSKRDILFTSFISGALAGVVCDVTLFPLDTLKTRLQSQHGFFQSGG---------FRYLYKGIGPVVLGSAPSAAIFFITYEGIKQ--------YSQP-----------------------YVPNQYHSIIHMIAASSSEITACLVRVPVEVIKQRKQ--ALLS---------DTHRLK-------------LRTLYRGYGSTVLRDLPFGVIQMPLWEYFKLYWTQ--QIER--------------ECTPLEGATCGAASVAISAAITTPLDVAKTRIMLSSTSAEKEE----VKISTMLKEVYRH------YGFKGLFAGFLPRVTGFTMGGFIFFGVYEQAREFYISYLVSEALVK
>C_secundus_A26_XP_023711320
-------------------------------MTNETDLPILTSLIAGGTAGITVDVTLFPLDTLKTRLQSPCGFFASGG---------FQHLYKGVGTAAVGSVPSASVFFCTYDTIKK--------IFSK-----------------------QVDPVYLPVIHMGAACAGEIGSCLVKVPIEVVKQRQQ--AALEKLSPLLICKRAIETEG-------------YLGLYRGFVTTTLREIPFVLIQFPIWEWFKAQWYC--AKKE--------------NLSIIEVAACGSVSGGIAAAVTTPLDVAKTRIMLAEKNIHSHVDCWRLRTSTMLINIYRE------QGVQGLFAGFVPRVLWITLGGAVFFGAYQAAVDFCSQSDVLR
>D_ponderosae_A26_XP_019766846
------------------------MGTQASNVEKSQHNLLLSAFWGGGAAGLFVDIVLFPLDTLKTRLQAEQGFKNAGA---------FKGIYKGLGPQVIGSAPQAALFFVTYESIKH--------YSEP-----------------------LVPKAAMPFVYMFGASIAEVMACLVRVPMEIAKQRKQ--ISPTDKSSLRILMSAYKYEGF-------------FKGVYRGFGSTIMREIPFSIIQFPTLEFCKSFYRQKFKNNI--------------PLDSWEVAVCGSIAGGASAAITTPLDVVKTRIMLADR---KVAERSSLTFANTFKKVLRN------EGLKGLFAGIVPRTLWIFLGGYIFFGSYDFAKNNVFDLLEERSDATR

>A_thaliana_A26_NP_568060     1                    2                    3                    4                    5                    6                    7                    8                    9                    10                    11
SATSHDVSKRVMQSSQLKINKGFFASVNTQEDKPFDFFRTLFEGFIAGGTAGVVVETALYPIDTIKTRLQAAR-GGGKIVL---------KGLYSGLAGNIAGVLPASALFVGVYEPTKQ-------KLLKT------------------------FPDHLSAVAHLTAGAIGGLAASLIRVPTEVVKQRMQ---TGQFTSAPSAVRMIASKEG-------------FRGLYAGYRSFLLRDLPFDAIQFCIYEQLCLGYKKAARRELS---------------DPENALIGAFAGALTGAVTTPLDVIKTRLMVQGSAKQYQG------IVDCVQTIVRE------EGAPALLKGIGPRVLWIGIGGSIFFGVLESTKRTLAQRRPNTVKETKEE
>O_sativa_A26_NP_001055312
-------------------------MAVGGEEEKPFNFLQILCEGVIAGGTAGVVVETALYPIDTIKTRLQAAR-GGSQIQW---------KGLYSGLAGNIAGVLPASAVFVGIYEPTKR-------KLLET------------------------FPENLSAVAHFTAGAIGGIAASLIRVPTEVVKQRMQ---TGQFRSAPDAVRLIVGKEG-------------FRGLYAGYGSFLLRDLPFDAIQFCIYEQLRIGYKVVAKRELN---------------DPENALIGAFAGAITGAITTPLDVMKTRLMVQGSANQYSG------IVSCAQTILRE------EGPGAFLKGIEPRVLWIGIGGSIFFGVLEKTKSMLAERRSREPPAGKDE
>P_patens_A26_XP_024397817
--------EKLKRSPADRPLLKFTVSSVQETQKAASLLHDIYVGAVAGATAGVVVEAALYPIDTIKTRLQAAHLGGNPSLF---------KGLYSGLAGNLAGVLPASAVFVGVYEPVKR-------KLLSV------------------------LPDQFSSIAHLTAGVCGGGISSLIRVPTEVVKQRMQ---TGQFVSPQSAVRQIVAKEG-------------IRGLYAGFRSFLLRDLPFDAFQFCIYEQLKISYAKRVRRELN---------------DTETAFIGAIAGAMTGAVTTPLDVIKTRLMIQGPNQQYKG------IQDCVVKIIRD------EGAGTLFQGLGPRILWIGVGGSIFFGALEKTKESMEERSLSKIITAPAGYV
>C_reinhardtii_A26_XP_001695360_2
MSLNANAAPSGPAKVPLFMLASAAETSASAPPPGTPMWRLAAANLAAGATAGCAVELALYPIDTIKTRLQAMIGGGGLKALLQSG---GGKGLYAGVWGNLAGVAPASAIFMAFYEPTKQ-------AVQAE------------------------VSADKQYLGPVVAGMVAGTASSLIRVPTEVVKQRLQ---TGEFKGAITAIRTILGREG-------------LRGMYAGYGAFMLRDLPFDAIEFVAYEQIKKAYGASVGRELN---------------PGETSLIGAFAGGFTGVITTPLDVLKTRLMTQGASGRYKN------LFDATVTIART------EGMGAFMSGWQPRLIWISLGGFVFFPVLEAAKKFYAPK

>S_cerevisiae_A26_SAM5_NP_014395_3_no_introns
-----------------------------------MNTFFLSLLSGAAAGTSTDLVFFPIDTIKTRLQAKGGFFANGG---------YKGIYRGLGSAVVASAPGASLFFISYDYMKVKSRPYISKLYSQGS-----------------------EQLIDT-TTHMLSSSIGEICACLVRVPAEVVKQRTQ--VHSTN-SSWQTLQSILRNDNK---------EGLRKNLYRGWSTTIMREIPFTCIQFPLYEYLKKTWAKANGQS--------------QVEPWKGAICGSIAGGIAAATTTPLDFLKTRLMLNKTTA---------SLGSVIIRIYRE------EGPAVFFSGVGPRTMWISAGGAIFLGMYETVHSLLSKSFPTAGEMRA
>S_pombe_A26_NP_594641
------------HIFHGKNRHQLLRTCHFTPSKRHSAMSFFEALGAGICAGLAVDLSLFPIDTLKTRLQAKGGFVKNGG---------FHGVYRGLGSILVGSAPGASLFFTTYENMKS-------RLSQSG-----------------------LGLSDP-QIHMCSASLGEIAACIVRVPTEVIKQRAQ--ASGGTLSSRNILQTILKS-------------NNVWRDFYAGYGITIAREIPFTLIQFPIWEHLKLKWRIKHSRN-----------KN----LAHEAAISGSIAGGIAAALTTPFDVVKTRIMTSQQRL---------SYVFTIKSIVAH------EGFLALYKGIVPRVLWLSGGGAIFLGCYDVILNFMKAEGL
>N_crassa_A26_XP_956205
----------------------------MASQSSPPPFQTALLAGALAGTTVDLSLFPLDTLKTRLQSAQGFFASGG---------FRGIYRGVGSCVIGSAPGAAFFFVTYETTKG-------FLSTTGNSTTTTTTTTTTHTGDTNKGQGKKGLSSPGVQHGIAASLGEIAACAVRVPTEVIKQRAQ--AGQHGGSSLSSLLHILHQRSTIGL-------TGVWRELYRGWGITVMREVPFTVLQFPLWESLKKWGRERRQRIGWGLFGDSATGKDDKDVSAPESALYGSLAGGFAAALTTPLDVLKTRIMLSERRE---------GVVSLIQTIWRDGKTKTGSGIRPFFSGIGPRVMWISIGGAIFLGSYQSVVNAFEGTTRKKSSGGELSL
>A_fumigatus_A26_XP_753466
-------------------------MAQQSEAEPLVSLWTRSLISGAVAGLTVDCSLYPLDTIKTRLQKARHHAPSTPGSTLSLRQTIRGIYAGLPSVLFGSAPSAASFFIVYDGVKR-------SLLPPAG------------------SDTAATRSRIVITHSLASSMGEIAACAVRVPTEVVKQRAQ--AGLFGGSSLLALKDILALRHPDPTGIAKRGYGQVIREMYRGAGITIAREIPFTVLQFTMWESMKEAYAKRMLVPSTR------ESGAVSQIPASTSAMFGSVAGAIAAGLTTPLDVIKTRVMLARRGEGGDAR---VRIRDVVQEISGE-------GFGAFWRGMGPRVAWIGIGGAVFLGSYQWAWNTLERRREGQ
```

SAMCX subfamily

```
>AT2G35800_A_thaliana                    1                    2                    3                    4                    5
MVLLPYERLQD------DPR---NIWFEAATVVAVAPPVALPAGDVLKSALAGGLASALSTSLMHPIDTIKTRVQAS-TLSFPEVIAKLPEIGVRGVYRGSIPAILGQFSSHGLRTGIFEASKLVLINFAPNLPEIQVQSIASFCSTLLGTAVRIPCEVLKQRLQAGMFNNVGEAIVGTWKQDGPSGFFRGTGATLCREVPLYVVGMGLYAESKKMVAQALGRELEAWETIAVGAVSGGIAAVVTTPFDVMKTRMMTATPGRPISMSMVVVSILRNEGPLGLFKGAVPRFFWVAPLGAMNFAGYELAKKAMQKNEDAVLADQLGQKKLC----
>AT2G26360_A_thaliana
IVSLNFAKVGNFTSRSNEIAPLINEAVAKGKNFDTGTRVGLDVGHLLKSALAGGISCAFSAFLMHPVDTVKTQVQASTTLSFLEILSKIPEIGARGLYKGSIPAVVGQFASHGLRTSIYEASKLALPLVAPTLLDIQVQSIASFIGTVLGTTLRIPCEVLKQRLQANQFDNIVEATVSTWHQEGLKGLFRGTGVTLLREVPFYVAGMGLYNQSKKVVERQLGRELEPWEAIAVGALSGGFTAVLTTPFDVIKTRMMTAPQGVELSMLMAAYSILTHEGPLAFYKGAVPRFFWTAPLGALNLAGYELLQKAMITPLNQSVHSE----------
>AT2G35800_O_sativa_XP_015615134
MLLLPSERLED------DPR---NIWFEAATLVAVPPPVEISAGSVLKSALAGGLASALSTSVMHPIDSMKTRVQAS-SLSFPDLISTLPQIGLRGLYRGSIPAILGQFSSHGLRTGIFEASKLVLKSVAPTLPDIQVQSLSSFCSTILGTAVRIPCEVLKQRLQAGIFNNVGEAIVGTMQKDGPKGFFRGTGATLCREVPFYVAGMCLYAEAKKAAQHVLNRDLEPWETIAVGALSGGLAAVVTTPFDVMKTRMMTAPPGTPVSMQLIVFSILRNEGPLGLFKGAIPRFFWIAPLGAMNFAGYELAKKAMIQTESDSTDSVHEKRTTVGSRG
>AT2G35800_P_patens_XP_024380815
MLLLPPERLGD------DPR---MVWFEAATVVPMAPPVDIPAGSVLKSALAGGMASALSTSMLHPLDTVKTRVQAS-TLSFPEVIAKLPQIGVRGMYRGSIPAILGQFTSHGIRTGVLEASKLLLKNVGPDLSDLQVQSLSSFTSTVIGTAVRIPCEVLKQRLQAGLYNSVGEAIVGTYQRDGLQGFFRGTGVTLCREVPFYVAGMSIYEEAKKVVRKVLHRELQPWETIAIGGLSGGLAAIATTPFDVMKTRTMTAGPGMPSTMGAIMVAIVKDEGLLALFKGAIPRFFWIAPLGAMNFAGYELAKRAMENKDKEVQAVGEQDKSR-----
```

AT5G42130 (MFL1) subfamily

```
>AT5G42130_MFL1_A_thaliana_no_int
RIQTLIKQLSVWERAIIGAGAGGLAGAFTYVTLLPLDAIKTKLQTKGASQVYSNTFDAIVKTFQAKGILGFYSGVSAVIVGSTFSSAVYFGTCEFGKSLLSKFPDFPTVLIPPTAGAMGNIISSAIMVPKELITQRMQAGA-SGRSYQVLLKILEKDGILGLYAGYSATLLRNLPAGVLSYSSFEYLKAAVLEKTKQSHLEPLQSVCCGALAGAISASITTPLDVVKTRLMTQIHVEA---------------VDKLGGAMYTGVAGTVKQILTEEGWVGFTRGMGPRVVHSACFSAIGYFAFETARLTILNEYLKRKEESEANVAADS----
>AT5G42130_O_sativa_XP_015614413_no_int
AAVAATAPPTPLARAAIGACAGAAAGAFTYAALLPIDAVKTRIQAGAAA---GGSWQVFLDILRTDGPLGLYRGLSAVILGSASSSAVYFGTCELAKSLLR--PHLPPFLVPPLAGASGNVSSSAIMVPKELITQRLQSGAAKGRSWQVLLQILQTDGFFGLYAGYAATLLRNLPAGVLSYSSFEYLKAFTLKQRNKESLTPGESVLCGALAGAISAALTTPLDVVKTRLMTRVGTEG---------------SR--------TVVGTMREVVAEEGLMGLSRGIGPRVLHSACFAALGYCAFETARLAILKWYIEDCERKAAAEMKAGVGAA
>AT5G42130_P_patens_XP_024361501_no_int
SSAGRMVKLPLLERAGISALAGGIAGGFTNATLHPIDTVKTKLQTRGASKLYSGPLDVVSKVIAKQGIAGLYSGVQAAFVGSIISSSIYFGTYELGKGVFTSIGNCPKTLVPPLAAALGNITSSAILVPKEVVKQRLQAGM-VGSELDVFLQTIRTEGIGGLYAGYSAALLRNLPSNIISFSTFEYLKLAWLKDSEKTTLEPWQSVISGAAAGALSASLTTPLDVAKTRLMTQARKSVTSAGLSGVRAEAAARAQAIAAYTYTGVASTLHQIWVEEGALGLTQGMGPRLFYSACFSALGFFAFETTRVIILKKYLEDRAATENVDVRILRS--
```

MME subfamily

```
>AT1G74240_A_thaliana   1                    2                    3                    4                    5                    6                    7                    8                    9
MATKSSDSSHKQSPPSFRKS--------VEIKATHDQFFVWREFLWGGIAGAFGEGMMHPVDTLKTRLQSQIIMNATQRQKSILQMLRTVWVGDGLKGFYRGIAPGVTGSLATGATYFGFIESTKKWIEESHPSLAGHWAHFIAGAVGDTLGSFIYVPCEVIKQRMQIQGTSSSWSSYISRNSVPVQPRGDMYGYYTGMFQAGCSIWKEQGPKGLYAGYWSTLARDVPFAGLMVVFYEGLKDLTDQGKKKFP---Q---YGVNSSIEGLVLGGLAGGLSAYLTTPLDVVKTRLQVQGSTIKYKGWLDAVGQIWRKEGPQGFFRGSVPRVMWYLPASALTFMAVEFLRDNFREKSNNNNNVVSNLSIERKTSSTSVHEG
>AT1G74240_O_sativa_XP_015630298
MAA----SPSAPPPPAAAAGVGVWSPAPQSPSPNLANFFVWREFVWGAIAGAFGEGMMHPVDTLKTRLQSQAIITGAKAQKNIFQMIRTVWVSDGLKGFYRGISPGVTGSLATGATYFGVIESTKTWLEHSNPNLSGHWSHFIAGGIGDTLGSFIYVPCEVMKQRMQVQGTKKSWALTATKGNISQTPGAPMYNYYNGMFHAGCSIWRDHGLKGLYAGYWSTLARDVPFAGLMVTFYEAMKELTEYGKRKYL---PESNLHASSSFEGLLLGGLAGGFSAYLTTPLDVIKTRLQVQGSTTSYNGWLDAITKTWANEGMSGLFKGSIPRIIWYIPASAFTFMAVEFLRDHFNEKIDTDARELTGLSMDTRSEVEEAA--
>AT1G74240_P_patens_XP_024361289
-----------------MVEENNLSPRLQASRSSAKGFFSWREFAWGALASGFGETLMHPVDTLKTRIQSGQSGVTLQKQSDIGHALKNIVAMDGVRALYRGVVPGLTGSMITGATYFGFIESTKDWLEDERPNLAGPWAHFCAGAVGDTLGSVVYVPCEVLKQRMQIQGSSKGWHQRHGASSRL---VTPSLQYYPGMWHAGQAILKYEGLSGLYAGYFSTLARDVPFAGFQIMLYEGMRAATVFGRRNSSVPPVEFQKHEFSSLEELMMGGTAGGLSAFLTTPMDVLKTRLQIQGSHMRYKGWFDAWQQIWRLEGIKGFFRGALPRVLWFVPASAVSFMAVEWLRKEFNTQTPVRIDSQ---SIQPDGSLSGSLS-
```

PiC subfamily

>H_sapiens_PiC_SLC25A3
[illegible]
>G_gallus_PiC_NP_001006236
[illegible]
>X_tropicalis_PiC_NP_988928
[illegible]
>D_rerio_PiC_NP_957009
[illegible]
>D_rerio_PiC_NP_998887
[illegible]

>C_elegans_PiC_NP_001076672
[illegible]
>C_elegans_PiC_NP_492561
[illegible]
>C_elegans_PiC_NP_494870
[illegible]
>C_elegans_PiC_NP_506148
[illegible]
>A_californica_PiC_XP_012938672
[illegible]
>C_intestinalis_PiC_XP_002130023
[illegible]
>A_planci_PiC_XP_022104926
[illegible]

>D_melanogaster_PiC_NP_611468
[illegible]
>D_melanogaster_PiC_NP_001246757
[illegible]
>B_tabaci_PiC_XP_018903618
[illegible]
>S_frugiperda_PiC_XP_035453816
[illegible]
>A_echinatior_PiC_XP_011058631
[illegible]
>C_secundus_PiC_XP_023713200
[illegible]
>D_ponderosae_PiC_XP_019765673
[illegible]

>A_thaliana_PiC_NP_190454
[illegible]
>A_thaliana_PiC_NP_196908
[illegible]
>A_thaliana_PiC_NP_179319
[illegible]
>O_sativa_PiC_NP_001063353
[illegible]
>O_sativa_PiC_XP_015633579
[illegible]
>O_sativa_PiC_XP_015611846
[illegible]
>P_patens_PiC_XP_024369444_no_introns
[illegible]
>P_patens_PiC_XP_024392150
[illegible]
>P_patens_PiC_XP_024365442
[illegible]
>C_reinhardtii_PiC_XP_001703471
[illegible]

>S_cerevisiae_PiC_PIC2_NP_010973_3_no_introns
[illegible]
>S_cerevisiae_PiC_MIR1_NP_012611_no_introns
[illegible]
>S_pombe_PiC_NP_596208_no_introns
[illegible]
>N_crassa_PiC_XP_011393175
[illegible]
>N_crassa_PiC_XP_963478
[illegible]
>A_fumigatus_PiC_XP_755540
[illegible]
>A_fumigatus_PiC_XP_754771
[illegible]
>A_fumigatus_PiC_XP_750023
[illegible]
>A_fumigatus_PiC_XP_752883
[illegible]

SLC25A46 subfamily

>H_sapiens_slc25a46
[illegible]
>G_gallus_A46_NP_001007831
[illegible]
>X_tropicalis_A46_NP_001016130
[illegible]
>D_rerio_A46_NP_001002558
[illegible]

>C_elegans_A46_NP_493364
[illegible]
>A_californica_A46_XP_005096971
[illegible]
>C_intestinalis_A46_XP_026692241
[illegible]
>A_planci_A46_XP_022095970
[illegible]

>D_melanogaster_A46_NP_573096
[illegible]
>D_melanogaster_A46_NP_609864
[illegible]
>B_tabaci_A46_XP_018910883
[illegible]
>S_frugiperda_A46_XP_035447278
[illegible]
>A_echinatior_A46_XP_011065288
[illegible]
>C_secundus_A46_XP_023718131
[illegible]
>D_ponderosae_A46_XP_019753589
[illegible]

MTCH subfamily

\>H_sapiens_slc25a49_MTCH1
[illegible]
\>H_sapiens_slc25a50_MTCH2
[illegible]
\>G_gallus_A50_NP_990139
[illegible]
\>X_tropicalis_A50_NP_989114
[illegible]
\>D_rerio_A50_NP_571457
[illegible]

\>C_elegans_A50_NP_495545
[illegible]
\>C_intestinalis_A50_XP_026696400
[illegible]
\>A_californica_A50_XP_005103103
[illegible]
\>A_planci_A50_XP_022112067
[illegible]

\>D_melanogaster_A50_NP_728481
[illegible]
\>B_tabaci_A50_XP_018915792
[illegible]
\>S_frugiperda_A50_XP_035444972_no_introns
[illegible]
\>A_echinatior_A50_XP_011052186
[illegible]
\>C_secundus_A50_XP_023709165
[illegible]
\>D_ponderosae_A50_XP_019755553
[illegible]

MCART subfamily

\>H_sapiens_SLC25A51_NP_219480
[illegible]
\>H_sapiens_A52_NP_001029344
[illegible]
\>H_sapiens_A53_NP_001012773
[illegible]
\>G_gallus_A51_XP_003643156
[illegible]
\>X_tropicalis_A51_NP_001107506
[illegible]
\>X_tropicalis_A53_XP_002940455
[illegible]
\>D_rerio_A51_XP_002664343
[illegible]
\>D_rerio_A51_XP_684328
[illegible]
\>D_rerio_A52_NP_957049
[illegible]

\>C_elegans_A51_NP_501642
[illegible]
\>A_californica_A52_XP_005113383
[illegible]
\>C_intestinalis_A51_XP_009862144
[illegible]
\>A_planci_A51_XP_022110290
[illegible]

\>D_melanogaster_A51_NP_651766
[illegible]
\>B_tabaci_A52_XP_018897075
[illegible]
\>S_frugiperda_A51_XP_035430352
[illegible]
\>Aechinatior_A51_XP_011060285
[illegible]
\>C_secundus_A51_XP_023708196
[illegible]
\>D_ponderosae_A52_XP_019759873
[illegible]

AT4G11440 subfamily

\>A_thaliana_AT4G11440
[illegible]
\>AT4G11440_O_sativa_XP_015627693
[illegible]
\>AT4G11440_P_patens_XP_024401911
[illegible]

AT4G15010 subfamily

\>AT4G15010_A_thaliana
[illegible]
\>AT4G15010_O_sativa_NP_001406624
[illegible]
\>AT4G15010_P_patens_XP_024397039
[illegible]

TABLE S1. The total collection of MC proteins divided into subfamilies. The MC protein sequences from *H. sapiens*, *A. thaliana* and *S. cerevisiae* were used to collect orthologs in well-annotated genomes based on unambiguous reciprocal best hits. This collection contained a total of 768 MC proteins, which are listed according to their main phylogenetic cluster and subfamily. The belonging of all protein sequences to the MC subfamilies was confirmed by using mainly OrthoDB v11 and OMA 2024, and sometimes also considering Treefam v9, EggNOG 6.0 and HOGENOM. The ortholog groups in the hierarchy indicating the specific MC subfamilies are in color.

Table S1. The total collection of MC proteins divided into subfamilies.

Cluster	Subfamily	MC protein	OrthoBD 1	OrthoBD 2	OrthoBD 3	OrthoBD 4	OrthoBD 5	OrthoBD 6	OrthoBD 7	OMA	HOGENOM	Treefam	EggNOG
	(new isoform)												
**MC-NT2**													
	NDT	AtNDT2_AT1G25380	10266426at2759	2972at33090	190085at3193					934339	CLU_015166_6_4	TF314217	KOG0757
		AtNDT1_AT2G47490	10266426at2759	2972at33090	190085at3193					934203	CLU_015166_6_4	TF314217	KOG0764
		NDT1_O_sativa_XP_015640768	10266426at2759	2972at33090						934203	CLU_015166_6_4		KOG0757
		NDT2_O_sativa_XP_015622200	10266426at2759	2972at33090						934289	CLU_015166_6_4		KOG0757
		NDT1_P_patens_XP_024387230	10266426at2759	2972at33090						934215	CLU_015166_6_4		KOG0757
		NDT1_C_reinhardtii_XP_042925003	10266426at2759	2972at33090							CLU_015166_6_1		
		ScNdt1p_YIL006W	10266426at2759	3506746at4751	1872175at4890					934215	CLU_015166_6_1	TF314220	KOG0764
		ScNdt2p_YEL006W	10266426at2759	3506746at4751	1872175at4890					934277	CLU_015166_6_1	TF314217	KOG0764
		NDT1_S_pombe_NP_592957	10266426at2759	3506746at4751							CLU_015166_6_1		
		NDT1_N_crassa_XP_960808	10266426at2759	3506746at4751							CLU_015166_6_1		
		NDT1_A_fumigatus_XP_752148	10266426at2759	3506746at4751							CLU_015166_6_1		
	PNC	HsPNC1_SLC25A33	269120at2759	4289186at33208	929768at7742					934215	CLU_015166_6_0	TF314220	KOG0757
		HsPNC2_SLC25A36	269120at2759	4289186at33208	929768at7742					934196	CLU_015166_6_0	TF314220	KOG0757
		A36_X_tropicalis_A36_XP_002941237	269120at2759	4289186at33208						934196	CLU_015166_6_0		
		A33_D_rerio_A33_NP_998322	269120at2759	4289186at33208							CLU_015166_6_0		
		A36_D_rerio_A36_NP_001002667	269120at2759	4289186at33208							CLU_015166_6_0		
		A36_C_elegans_A36_NP_496236	269120at2759	4289186at33208							CLU_015166_6_0		
		A36_A_californica_A36_XP_012938953	269120at2759	4289186at33208						934247			
		A36_C_intestinalis_A36_XP_002130147	269120at2759	4289186at33208									KOG0757
		A36_A_planci_A36_XP_022090196	269120at2759	4289186at33208						934286			
		A36_D_melanogaster_A36_NP_001245840	269120at2759	4289186at33208							CLU_015166_6_0		
		A36_B_tabaci_A36_XP_018913182	269120at2759	4289186at33208									
		A36_S_frugiperda_A36_XP_035443562	269120at2759	4289186at33208									
		A36_A_echinatior_A36_part_XP_011066235	269120at2759	4289186at33208						934469			
		A36_A_echinatior_A36_part_XP_011051031	269120at2759	4289186at33208						934612			
		A36_C_secundus_A36_XP_023717143	269120at2759	4289186at33208									KOG0757
		A36_D_ponderosae_A36_XP_019771379	269120at2759	4289186at33208									
		ScRim2p_YBR192W	269120at2759	153193at4751	1883104at4890					934196	CLU_015166_6_0	TF314220	KOG0757
		A36_S_pombe_A36_NP_594067	269120at2759	153193at4751							CLU_015166_6_0		
		A36_N_crassa_A36_XP_965547	269120at2759	153193at4751							CLU_015166_6_0		
		A36_A_fumigatus_A36_XP_754911	269120at2759	153193at4751							CLU_015166_6_0		
	MFT/FAD	HsMFT_SLC25A32	428293at2759	68082at33208	1540033at7742					934203	CLU_015166_6_4	TF314217	KOG0764
		A32_G_gallus_A32_NP_001026677	428293at2759										
		A32_X_tropicalis_A32_NP_001107692	428293at2759										
		A32_D_rerio_A32B_NP_001013354	428293at2759										
		A32_D_rerio_A32A_NP_956550_1	428293at2759										
		A32_C_elegans_A32_NP_495746	428293at2759										
		A32_A_californica_A32_XP_005103524	428293at2759										
		A32_C_intestinalis_A32_XP_002121509	428293at2759										
		A32_A_planci_A32_XP_022103614	428293at2759										
		A32_D_melanogaster_A32_NP_724769	428293at2759										
		A32_B_tabaci_A32_XP_018915411	428293at2759										
		A32_S_frugiperda_A32_XP_035446918	428293at2759										
		A32_A_echinatior_A32_XP_011050458	428293at2759										
		A32_C_secundus_A32_XP_033609174	428293at2759										

		A32_D_ponderosae_A32_XP_019762367	428293at2759										
		AtFOLT_AT5G66380	269120at2759	1526389at33090	1335469at3193					934196	CLU_015166_6_4	TF314217	KOG0764
		A32_O_sativa_A32_NP_001051182_1	269120at2759	1526389at33090							CLU_015166_6_4		
		A32_P_patens_XP_024368225	269120at2759	1526389at33090							CLU_015166_6_4		
		A32_C_reinhardtii_A32_XP_042915427	269120at2759	1526389at33090									
		ScFlx1p_YIL134W	428293at2759	28883at4751	660438at4890					934203	CLU_015166_6_4	TF314217	KOG0764
		A32_S_pombe_A32_NP_595541	428293at2759								CLU_015166_6_4		
		A32_N_crassa_A32_XP_961140	428293at2759								CLU_015166_6_4		
		A32_A_fumigatus_A32_XP_747560_2	428293at2759								CLU_015166_6_4		
	peCFNC	HsSLC25A17	10266426at2759	2874181at33208	261800at7742					934197	CLU_015166_6_3	TF324772	KOG0769
		A17_G_gallus_A17_XP_416242	10266426at2759	2874181at33208						934197			
		A17_X_tropicalis_A17_NP_001004781	10266426at2759	2874181at33208						934197	CLU_015166_6_3		
		A17_D_rerio_A17_NP_001092731	10266426at2759	2874181at33208						934197			
		A17_A_californica_A17_XP_005105779	10266426at2759	2874181at33208						934313			
		A17_A_planci_A17_XP_022102396	10266426at2759	2874181at33208						934220			
		A17_D_melanogaster_A17_NP_728982	10266426at2759	2874181at33208						934197	CLU_015166_6_3		
		A17_B_tabaci_A17_XP_018907948	10266426at2759	2874181at33208									
		A17_S_frugiperda_A17_XP_035458321	10266426at2759	2874181at33208									
		A17_A_echinatior_A17_XP_011052484	10266426at2759	2874181at33208									
		A17_C_secundus_A17_XP_023705543	10266426at2759	2874181at33208									
		AtPXN_AT2G39970	2019556at2759	1525446at33090	1316704at3193					934197	CLU_015166_6_3	TF324772	KOG0769
		A17_O_sativa_A17_NP_001063624	2019556at2759							934259	CLU_015166_6_3		
		A17_P_patens_A17_XP_024384212	2019556at2759										
		A17_N_crassa_A17_XP_956119	2019556at2759										
		A17_A_fumigatus_A17_XP_747912_2	2019556at2759										
	peANT	AtPNC1_AT3G05290	446044at2759	1525269at33090	1333004at3193					934220	CLU_015166_6_3	TF324772	KOG0769
		AtPNC2_AT5G27520	446044at2759	1525269at33090	1333004at3193					934280	CLU_015166_6_3	TF324772	KOG0769
		ANT1_O_sativa_PNC1_XP_015640301	446044at2759										
		ANT1_P_patens_PNC1_XP_024371147	446044at2759										
		ScAnt1p_YPR128C	446044at2759	1831536at4751	498210at4890					934220	CLU_015166_6_3	TF332793	KOG0769
		ANT1_N_crassa_XP_957787	446044at2759										
		ANT1_A_fumigatus_XP_750094	446044at2759										
**MC-NT1**													
	AAC	HsSLC25A4	270584at2759	90156at33208	1544060at7742					934278	CLU_015166_12_0	TF300743	KOG0749
		HsSLC25A5	270584at2759	90156at33208	1544060at7742					934239	CLU_015166_12_0	TF300743	KOG0749
		HsSLC25A6	270584at2759	90156at33208	1544060at7742					934245	CLU_015166_12_0	TF300743	KOG0749
		A4_G_gallus_A4_NP_001006443	270584at2759	90156at33208	1544060at7742					934211	CLU_015166_12_0	TF300743	KOG0749
		A5_G_gallus_A5_XP_004940900_2	270584at2759	90156at33208	1544060at7742					934239			
		A6_G_gallus_A6_NP_989562	270584at2759	90156at33208	1544060at7742					934245			
		A4_X_tropicalis_A4_NP_988909	270584at2759	90156at33208	1544060at7742					934211	CLU_015166_12_0	TF300743	KOG0749
		A5_X_tropicalis_A5_NP_988913	270584at2759	90156at33208	1544060at7742					934239	CLU_015166_12_0	TF300743	KOG0749
		A4_D_rerio_A4_NP_999867	270584at2759	90156at33208	1544060at7742					934278		TF300743	KOG0749
		A5_D_rerio_A5_NP_775354	270584at2759	90156at33208	1544060at7742					934290	CLU_015166_12_0	TF300743	KOG0749
		A6_D_rerio_A6_NP_001122204	270584at2759	90156at33208	1544060at7742					934306	CLU_015166_12_0	TF300743	KOG0749
		A6_C_elegans_A6A_NP_001022799	270584at2759	90156at33208	20254at6231					934225	CLU_015166_12_0		KOG0749
		A6_C_elegans_A6B_NP_491927	270584at2759	90156at33208	20254at6231					934320	CLU_015166_12_0		KOG0749
		A6_C_elegans_A6C_NP_501440	270584at2759	90156at33208	20254at6231					934306	CLU_015166_12_0		KOG0749
		A6_C_elegans_A6D_NP_501727	270584at2759	90156at33208	20254at6231					934245	CLU_015166_12_0		KOG0749

		A5_A_planci_A5_XP_022108762	270584at2759	90156at33208						934211			
		A5_C_californica_XP_005107474	270584at2759	90156at33208	188411at1206795					934283			
		A6A_C_intestinalis_XP_002131174	270584at2759	90156at33208						934211	CLU_015166_12_0	TF300743	
		A6B_C_intestinalis_XP_002126946	270584at2759	90156at33208						934405	CLU_015166_12_0	TF300743	
		A4_D_melanogaster_A4_NP_727448	270584at2759	90156at33208	155063at6656					934211	CLU_015166_12_0		KOG0749
		A6_B_tabaci_A6_XP_018907997	270584at2759	90156at33208	155063at6656								
		A6_S_frugiperda_XP_035454457	270584at2759	90156at33208	155063at6656								
		A4_A_echinatior_A4_XP_011066844	270584at2759	90156at33208	155063at6656					934327			
		A6_A_echinatior_A6_XP_011059688	270584at2759	90156at33208	155063at6656					934211			KOG0749
		A4_C_secundus_A4_XP_023727041	270584at2759	90156at33208	155063at6656								KOG0749
		A5_D_ponderosae_A5_XP_019759267	270584at2759	90156at33208	155063at6656					934211	CLU_015166_12_0		
		A4_D_ponderosae_A4_XP_019766514	270584at2759	90156at33208	155063at6656					934306	CLU_015166_12_0		KOG0749
		AtAAC1_AT3G08580	270584at2759	1518916at33090	1325831at3193					934320	CLU_015166_12_1	TF300743	KOG0749
		AtAAC2_AT5G13490	270584at2759	1518916at33090	1325831at3193					934327	CLU_015166_12_1	TF300743	KOG0749
		AtAAC3_AT4G28390	270584at2759	1518916at33090	1325831at3193					934278	CLU_015166_12_0	TF300743	KOG0749
		A4_O_sativa_A4_XP_015640040	270584at2759	1518916at33090	1325831at3193					934211	CLU_015166_12_0		
		A5_O_sativa_A5_XP_015625645	270584at2759	1518916at33090	1325831at3193					934245	CLU_015166_12_0		KOG0749
		A4_P_patens_A4_XP_024394210	270584at2759	1518916at33090	1325831at3193					934290			KOG0749
		A4_C_reinhardtii_A4_XP_001695177	270584at2759	1518916at33090	14661at3041					934290	CLU_015166_12_0		KOG0749
		ScAac1p_YMR056C	270584at2759	38510at4751	1873467at4890					934283	CLU_015166_12_0	TF300743	KOG0749
		ScAac2p_YBL030C	270584at2759	38510at4751	1873467at4890					934211	CLU_015166_12_0	TF300743	KOG0749
		ScAac3p_YBR085W	270584at2759	38510at4751	1873467at4890					934245	CLU_015166_12_0	TF300743	KOG0749
		A4_S_pombe_NP_595323	270584at2759	38510at4751						934211	CLU_015166_12_0		KOG0749
		A4_N_crassa_A4_XP_011393638	270584at2759	38510at4751						934211	CLU_015166_12_0		KOG0749
		A4_A_fumigatus_A4_XP_750288	270584at2759	38510at4751						934211	CLU_015166_12_0		KOG0749
	(SLC25A31)	HsSLC25A31	270584at2759	90156at33208	1548473at7742					934225	CLU_015166_12_0	TF300743	KOG0749
		A31_C_amiliaris_XP_540952_3	270584at2759	90156at33208	1548473at7742					934225			
		A31_B_taurus_NP_001039965	270584at2759	90156at33208	1548473at7742					934225			KOG0749
		A31_M_musculus_NP_848473_2	270584at2759	90156at33208	1548473at7742					934225	CLU_015166_12_0	TF300743	KOG0749
	(ER_ANT)	AtER_ANT_AT5G17400	270584at2759	1518916at33090	1322539at3193					934239	CLU_015166_12_0	TF300743	KOG0749
		ER_ANT1_O_sativa_XP_015615607	270584at2759	1518916at33090	1322539at3193					934239	CLU_015166_12_0		KOG0749
	(PM_ANT)	AtPM_ANT_AT5G56450	270584at2759	1518916at33090	1316380at3193					934225	CLU_015166_12_0	TF300743	KOG0749
		PM_ANT1_O_sativa_NP_001407771	270584at2759	1518916at33090	1316380at3193					934225	CLU_015166_12_0		KOG0749
		PM_ANT1_P_patens_XP_024367991	270584at2759	1518916at33090	1316380at3193					934225			KOG0749
	TAAC	AtPAPST_AT5G01500	270584at2759	908332at33090	1340378at3193					934232	CLU_015166_10_5	TF354298	KOG0752
		At_AT3G51870	270584at2759	908332at33090	1340378at3193					934359	CLU_015166_10_5	TF354298	KOG0752
		TAAC_O_sativa_XP_025880286	270584at2759	908332at33090						934232	CLU_015166_10_5		KOG0752
		TAAC_P_patens_XP_024390888	270584at2759	908332at33090						934328			
		TAAC_P_patens_XP_024393427	270584at2759	908332at33090						934359			
		TAAC_C_reinhardtii_XP_001696228	270584at2759	1405753at33090	897at3041	12403at3166				934465	CLU_015166_10_2		KOG0752
	AACX	At_AT5G64970	270584at2759	1521154at33090	1331766at3193					934445	CLU_015166_10_4	TF354298	KOG0752
		At_AT1G78180	270584at2759	1521154at33090	1331766at3193					934832	CLU_015166_10_4	TF354298	KOG0752/K
		AACX_O_sativa_XP_015633018	270584at2759	1521154at33090						934298	CLU_015166_10_4		KOG0752
	SLC25A43	HsSLC25A43	270584at2759	4226891at33208	79577at7742					934232	CLU_015166_10_3	TF354298	KOG0752
		A43_G_gallus_A43_XP_420343	270584at2759	4226891at33208						934232			
		A43_X_tropicalis_A43_XP_002934585	270584at2759	4226891at33208						934232	CLU_015166_10_3	TF354298	KOG0752
		A43_D_rerio_A43_NP_001004497	270584at2759	4226891at33208						934232			KOG0752
		A43_A_planci_A43_XP_022098882	270584at2759	4226891at33208						934232			

	APCX	At_AT3G55640	270584at2759	1523214at33090	1319328at3193					934344	CLU_015166_10_3	TF314806	KOG0752
		At_AT2G37890	270584at2759	1523214at33090	1319328at3193					934266	CLU_015166_10_3	TF314806	KOG0752
		At_AT3G53940	270584at2759	1523214at33090	1319328at3193					934441	CLU_015166_10_3		KOG0752
		APCX_O_sativa_XP_015632416	270584at2759	1523214at33090						934266	CLU_015166_10_3		KOG0752
		APCX_P_patens_XP_024361098	270584at2759	1523214at33090						934303	CLU_015166_10_3		
		APCX_P_patens_XP_024383346	270584at2759	1523214at33090						934303			
		APCX_C_reinhardtii_XP_042919064	270584at2759	1523214at33090						934303			
	APC	HsSLC25A23	270584at2759	4224206at33208	1053986at7742					934244	CLU_015166_2_0	TF313492	KOG0036
		HsSLC25A24	270584at2759	4224206at33208	541425at7742					934227	CLU_015166_2_0	TF313492	KOG0036
		HsSLC25A25	270584at2759	4224206at33208	1059175at7742	872682at32523				934210	CLU_015166_2_0	TF313492	KOG0036
		A24_G_gallus_A24_XP_422180	270584at2759	4224206at33208	541425at7742					934227			
		A25_G_gallus_A25_XP_004945868_2	270584at2759	4224206at33208	1059175at7742	872682at32523				934210			
		A23_X_tropicalis_A23_NP_001135638	270584at2759	4224206at33208	1059175at7742	872682at32523				934244			
		A24_X_tropicalis_A24_NP_001011047	270584at2759	4224206at33208	541425at7742					934227			KOG0036
		A25_X_tropicalis_A25_NP_001011052	270584at2759	4224206at33208	1059175at7742	872682at32523				934210	CLU_015166_2_0	TF313492	KOG0036
		A23_D_rerio_A23B_NP_001038417	270584at2759	4224206at33208	1059175at7742	389348at7898				934244	CLU_015166_2_0	TF313492	KOG0036
		A24_D_rerio_A24_NP_001004606	270584at2759	4224206at33208	541425at7742					934427	CLU_015166_2_0	TF313492	KOG0036
		A25_D_rerio_A25_NP_001153492	270584at2759	4224206at33208	1059175at7742	389348at7898				934210	CLU_015166_2_0		KOG0036
		A25_C_elegans_A25_NP_001256365	270584at2759	4224206at33208	39060at6231					934263	CLU_015166_2_0		
		A25_C_elegans_A25_NP_510081	270584at2759	4224206at33208	39060at6231					934210	CLU_015166_2_0		KOG0036
		A25_A_californica_A25_XP_005111405	270584at2759	4224206at33208	188652at1206795								
		A24_C_intestinalis_A24_XP_026691621	270584at2759	4224206at33208									KOG0036
		A24_A_planci_A24_XP_022085201	270584at2759	4224206at33208						934244			
		A25_D_melanogaster_A25_NP_996067	270584at2759	4224206at33208	1921245at6656					934263	CLU_015166_2_0		KOG0036
		A23_B_tabaci_A23_XP_018917803	270584at2759	4224206at33208	1921245at6656								
		A25_S_frugiperda_A25_XP_035437774	270584at2759	4224206at33208	1921245at6656								
		A25_A_echinatior_A25_XP_011056141	270584at2759	4224206at33208	1921245at6656								
		A25_C_secundus_A25_XP_023727103	270584at2759	4224206at33208	1921245at6656								
		A25_D_ponderosae_XP_019772927	270584at2759	4224206at33208	1921245at6656								
		AtAPC1_AT5G61810	270584at2759	1405753at33090	1302062at3193					934244	CLU_015166_2_1	TF313492	KOG0036
		AtAPC2_AT5G51050	270584at2759	1405753at33090	1302062at3193					934210	CLU_015166_2_1	TF313492	KOG0036
		AtAPC3_AT5G07320	270584at2759	1405753at33090	1302062at3193					934227	CLU_015166_2_1	TF313492	KOG0036
		A23_O_sativa_A23_NP_001058018	270584at2759	1405753at33090	1302062at3193								
		A25_O_sativa_A25_XP_015623396	270584at2759	1405753at33090	1302062at3193								
		A25_P_patens_A25_XP_024376863	270584at2759	1405753at33090	1302062at3193								
		A24_P_patens_A24_XP_024377247	270584at2759	1405753at33090	1302062at3193								
		A25_C_reinhardtii_A25_XP_042920166	270584at2759	1405753at33090	897at3041	12403at3166				934387			KOG0036
		ScSal1p_YNL083W	270584at2759	3479935at4751	1811004at4890					934284	CLU_015166_2_1	TF313492	KOG0036
		A24_S_pombe_NP_595952	270584at2759	3479935at4751	1811004at4890								
		A24_N_crassa_A24_XP_956201	270584at2759	3479935at4751	1811004at4890								
		A25_A_fumigatus_A25_XP_755913	270584at2759	3479935at4751	1811004at4890								
	(SLC25A41)	HsSLC25A41	270584at2759	4224206at33208	1059175at7742	453605at32523				934255	CLU_015166_10_2	TF313492	KOG0036
		A41_C_familiaris_XP_003639787_2	270584at2759	4224206at33208	1059175at7742	453605at32523							
		A41_B_taurus_NP_001069309	270584at2759	4224206at33208	1059175at7742	453605at32523							
		A41_M_musculus_NP_780542	270584at2759	4224206at33208	1059175at7742	453605at32523							
	CoC	CoC_At_AT4G26180	270584at2759	40693at33090	43221at3193	803565at71240/200460at4447				934194		TF314806	KOG0752
		CoC_At_AT1G14560	270584at2759	40693at33090	43221at3193	803565at71240	20486at3699			934273	CLU_015166_10_1	TF314806	KOG0752
		CoC_O_sativa_A42_XP_015623122	270584at2759	40693at33090	43221at3193	200460at4447							

		CoC_O_sativa_A16_NP_001043375	270584at2759	40693at33090	43221at3193	43221at4447	3221at38820						
		CoC_P_patens_A16_XP_024367005	270584at2759	40693at33090	43221at3193								
		CoC_Sc_Leu5p_YHR002W	270584at2759	13063at4751	1861786at4890					934194	CLU_015166_10_0	TF314806	KOG0752
		CoC_A_fumigatus_A42_XP_756014	270584at2759	13063at4751	1861786at4890								
		CoC_N_crassa_A16_XP_962539	270584at2759	13063at4751	1861786at4890	620691at147550	6271at5139						
		CoC_S_pombe_A16_NP_593578	270584at2759	13063at4751	1861786at4890					934194	CLU_015166_10_0		KOG0752
	(SLC25A42)	HsSLC25A42	270584at2759	177980at33208	414097at7742					934194	CLU_015166_10_1	TF314806	KOG0752
		A42_G_gallus_A42_XP_424684	270584at2759	177980at33208	414097at7742								
		A42_X_tropicalis_A42_NP_001072712	270584at2759	177980at33208	414097at7742								
		A42_D_rerio_A42_NP_001038918	270584at2759	177980at33208	414097at7742								
		A42_C_elegans_A42_NP_492333	270584at2759	177980at33208	145762at6231								
		A42_A_californica_A42_XP_005109276	270584at2759	177980at33208	229724at1206795	24046at6447							
		A42_A_planci_A42_XP_022099816	270584at2759	177980at33208						934194			
		A42_D_melanogaster_A42_NP_650891	270584at2759	177980at33208	43986at6656								
		A42_B_tabaci_A42_XP_018910155	270584at2759	177980at33208	43986at6656					934194			
		A42_S_frugiperda_A42_XP_035457558											
		A42_A_echinatior_A42_XP_011050869	270584at2759	177980at33208	43986at6656								
		A42_C_secundus_A42_XP_023717440	270584at2759	177980at33208	43986at6656								
		A42_D_ponderosae_A42_XP_019768973	270584at2759	177980at33208	43986at6656								
	(SLC25A16)	HsSLC25A16	270584at2759	177980at33208	1540287at7742					934202	CLU_015166_10_1	TF314806	KOG0752
		A16_G_gallus_A16_XP_421570								934202			
		A16_X_tropicalis_A16_NP_001135682	270584at2759	177980at33208	1540287at7742								
		A16_D_rerio_A16_NP_991112	270584at2759	177980at33208	1540287at7742								
		A16_A_californica_A16_XP_005094735	270584at2759	177980at33208	229724at1206795	10894at6447							
		A16_A_planci_A16_XP_022080115	270584at2759	177980at33208									
		A16_C_intestinalis_XP_002130726	270584at2759	177980at33208									
		A16_B_tabaci_A16_XP_018916786	270584at2759	177980at33208	80140at6656								
		A16_S_frugiperda_A16_XP_035443559	270584at2759	177980at33208	80140at6656								
		A16_C_secundus_A16_XP_023713459	270584at2759	177980at33208	80140at6656								
		A16_D_ponderosae_A16_XP_019766837	270584at2759	177980at33208	80140at6656								
	YPR011C	AtADNT1_AT4G01100	270584at2759	1405753at33090	1275098at3193					934529	CLU_015166_10_3	TF314806	KOG0752
		YPR011C_O_sativa_ADNT1_XP_015639781	270584at2759	1405753at33090	1275098at3193								
		YPR011C_P_patens_ADNT1_XP_02436912	270584at2759	1405753at33090	1275098at3193								
		YPR011C_C_reinhardtii_XP_001693350_2	270584at2759	1405753at33090	897at3041	12403at3166				934202			
		Sc_YPR011C	270584at2759	13063at4751	1895167at4890					934202	CLU_015166_10_1	TF314806	KOG0752
		YPR011C_S_pombe_NP_001018227	270584at2759	13063at4751	1895167at4890								
		YPR011C_N_crassa_XP_956574	270584at2759	13063at4751	1895167at4890								
		YPR011C_A_fumigatus_XP_755075	270584at2759	13063at4751	1895167at4890								
	TPC	HsSLC25A19	18574at2759	507438at33208	1497198at7742					934198	CLU_015166_10_3	TF313047	KOG0752
		A19_G_gallus_A19_XP_004946425	18574at2759										
		A19_X_tropicalis_A19_NP_001015953	18574at2759										
		A19_D_rerio_A19_NP_991278	18574at2759										
		A19_C_elegans_A19_NP_001255632	18574at2759										
		A19_A_californica_A19_XP_012945487	18574at2759										
		A19_C_intestinalis_A19_XP_026689747	18574at2759										
		A19_A_planci_A19_XP_022109907	18574at2759										
		A19_D_melanogaster_NP_650034	18574at2759										
		A19_B_tabaci_A19_XP_018900715	18574at2759										
		A19_S_frugiperda_XP_035444948	18574at2759										

		A19_A_echinatior_A19_XP_011064587	18574at2759										
		A19_C_secundus_A19_XP_023724089	18574at2759										
		A19_D_ponderosae_A19_XP_019766947	18574at2759										
		AtTPCh1_AT3G21390	18574at2759	71128at33090	1299418at3193					934350	CLU_015166_10_3	TF313047	KOG0752
		AtTPCh2_AT5G48970	18574at2759	71128at33090	1299418at3193					934198	CLU_015166_10_3	TF313047	KOG0752
		A19_O_sativa_A19_NP_001045309	18574at2759										
		A19_P_patens_A19_XP_024398037	18574at2759										
		ScTpc1p_YGR096W	18574at2759	15774at4751	1990223at4890					934198	CLU_015166_10_3	TF314806	KOG0752
		A19_S_pombe_A19_NP_596636_2	18574at2759										
		A19_N_crassa_A19_XP_958877	18574at2759										
		A19_A_fumigatus_A19_XP_755831_2	18574at2759										
	BT	AtBT1_AT4G32400	270584at2759	1526107at33090	85310at3193					934255	CLU_015166_10_8	TF354298	KOG0752
		AtBT1_O_sativa_XP_015643174	270584at2759	1526107at33090	85310at3193								
		AtBT1_P_patens_XP_024380479	270584at2759	1526107at33090	85310at3193								
	BTL	AtBTL1_AT3G20240	270584at2759	1526107at33090	52054at3193					934403	CLU_015166_10_8	TF354298	KOG0752
		AtBTL1_O_sativa_NP_001409573	270584at2759	1526107at33090	52054at3193								
**MC-CA**													
	OAC	HsSLC25A34	6703404at2759	33089at33208	1553893at7742					934249	CLU_015166_14_3	TF324506	KOG0755
		HsSLC25A35	6703404at2759	33089at33208	1553893at7742					934199	CLU_015166_14_3	TF324506	KOG0755
		A34_G_gallus_A34_XP_015152658	6703404at2759	33089at33208									
		A34_X_tropicalis_A34_NP_001135591	6703404at2759	33089at33208									
		A35_D_rerio_A35_NP_001092917	6703404at2759	33089at33208									
		A35_A_californica_A35_XP_005096117	6703404at2759	33089at33208									
		A35_A_planci_A35_XP_022104222	6703404at2759	33089at33208									
		A34_D_melanogaster_A34_NP_725361	6703404at2759	33089at33208									
		A34_D_melanogaster_A34_NP_001260966	6703404at2759	33089at33208									
		A34_D_melanogaster_A34_NP_610933	6703404at2759	33089at33208									
		A35_B_tabaci_A35_XP_018898797	6703404at2759	33089at33208									
		A35_S_frugiperda_A35_XP_035436167	6703404at2759	33089at33208									
		A35_A_echinatior_A35_XP_011065637	6703404at2759	33089at33208									
		A35_C_secundus_A35_XP_023704125	6703404at2759	33089at33208									
		A35_D_ponderosae_A35_XP_019771649	6703404at2759	33089at33208									
		ScOac1p_YKL120W	6703404at2759	4585at4751	1879648at4890					934199	CLU_015166_14_3	TF324506	KOG0755
		A34_S_pombe_NP_593169	6703404at2759	4585at4751									
		A34_N_crassa_A34_XP_962817	6703404at2759	4585at4751									
		A35_A_fumigatus_A35_XP_753416	6703404at2759	4585at4751									
	AT4G03115	At_AT4G03115	756301at2759	1522479at33090	1286803at3193					934252	CLU_015166_14_1	TF324506	KOG0753
		AT4G03115_O_sativa_XP_015620551	756301at2759	1522479at33090									
		AT4G03115_C_reinhardtii_XP_042916519	756301at2759	45536at33090	45536at3041								
	UCP2-3	HsSLC25A8	448427at2759	2108516at33208	1543353at7742	874044at32523	326790at40674			934218	CLU_015166_14_2	TF323211	KOG0753
		HsSLC25A9	448427at2759	2108516at33208	1543353at7742	874044at32523	3485at40674			934237	CLU_015166_14_2	TF323211	KOG0753
		A9_G_gallus_A9_NP_989438	448427at2759	2108516at33208	1543353at7742	874044at32523	374439at8457						
		A8_X_tropicalis_A8_NP_989179	448427at2759	2108516at33208	1543353at7742	874044at32523							
		A9_X_tropicalis_A9_NP_001107354	448427at2759	2108516at33208	1543353at7742	874044at32523							
		A8_D_rerio_A8_NP_571251	448427at2759	2108516at33208	1543353at7742	113242at7898							

		A9_D_rerio_A9_NP_955817	448427at2759	2108516at33208	1543353at7742	113242at7898							
		A9_C_intestinalis_A9_XP_018669017	448427at2759	2108516at33208									
		A9_A_planci_A9_XP_022111226	448427at2759	2108516at33208						934250			
		A8_B_tabaci_A8_XP_018906955	756301at2759	191178at33208	10034at6656	1605448at6960	1605448at50557	18314at7524					
		A8_A_echinatior_A8_XP_011068012	756301at2759	191178at33208	10034at6656	1605448at6960	1605448at50557	109043at7399					
		A8_C_secundus_A8_XP_023705760	756301at2759	191178at33208	10034at6656	1605448at6960	1605448at50557						
		AtUCP1_AT3G54110	448427at2759	30661at33090	1304698at3193					934218	CLU_015166_14_2	TF323211	KOG0753
		AtUCP2_AT5G58970	448427at2759	30661at33090	1304698at3193					934250	CLU_015166_14_2	TF323211	KOG0753
		A8_O_sativa_A8_NP_001068559	448427at2759	30661at33090	1304698at3193								
		A9_P_patens_A9_XP_024398138	448427at2759	30661at33090	1304698at3193								
		A9_C_reinhardtii_A9_XP_001695417	448427at2759	30661at33090	30661at3041	3329at3166							
	(UCP1)	HsSLC25A7	448427at2759	2108516at33208	1543353at7742	874044at32523	390314at40674			934261	CLU_015166_14_2	TF323211	KOG0753
		A7_BOVINE_NP_001160000	448427at2759	2108516at33208	1543353at7742	874044at32523	390314at40674						
		A7_CANIS_NP_001003046	448427at2759	2108516at33208	1543353at7742	874044at32523	390314at40674						
		A7_MOUSE_NP_033489	448427at2759	2108516at33208	1543353at7742	874044at32523	390314at40674						
	UCP5-6	HsUCP5_SLC25A14	756301at2759	191178at33208	422283at7742					934224	CLU_015166_14_2	TF323211	KOG0753
		HsUCP6_SLC25A30	756301at2759	191178at33208	422283at7742					934229	CLU_015166_14_2	TF323211	KOG0753
		A14_G_gallus_A14_NP_001012901	756301at2759	191178at33208	422283at7742								
		A30_G_gallus_A30_XP_417040	756301at2759	191178at33208	422283at7742								
		A30_X_tropicalis_A30_NP_001165746	756301at2759	191178at33208	422283at7742								
		A14_D_rerio_A14_NP_956458	756301at2759	191178at33208	422283at7742								
		A30_A_californica_A30_XP_005110096	756301at2759	191178at33208	208318at1206795					934229			
		A30_A_planci_A30_XP_022088031	756301at2759	191178at33208						934224			
		A30_D_melanogaster_A30_NP_648501	756301at2759	191178at33208	10034at6656	1605448at6960	1605448at50557	1229998at33392		934224			
		A14_B_tabaci_A14_XP_018904437	756301at2759	191178at33208	10034at6656	1605448at6960	1605448at50557	8340at7524					
		A30_S_frugiperda_A30_XP_035445673	756301at2759	191178at33208	10034at6656	1605448at6960	1605448at50557	1229998at33392	115148at7088				
		A30_A_echinatior_A30_XP_011049652	756301at2759	191178at33208	10034at6656	1605448at6960	1605448at50557	1229998at33392	115148at7088				
		A30_C_secundus_A30_XP_023716238	756301at2759	191178at33208	10034at6656	1605448at6960	1605448at50557						
		A30_D_ponderosae_A30_XP_019757301	756301at2759	191178at33208	10034at6656	1605448at6960	1605448at50557	1229998at33392					
	UCP4	HsUCP4_SLC25A27	756301at2759	191178at33208	1551130at7742					934213	CLU_015166_14_2	TF354328	KOG0753
		A27_X_tropicalis_A27_NP_001011241	756301at2759	191178at33208	1551130at7742								
		A27_D_rerio_A27_NP_956635	756301at2759	191178at33208	422283at7742					934213			
		A27_C_elegans_A27_NP_505414	756301at2759	191178at33208	79470at6231					934213			
		A27_A_californica_A27_XP_005096635	756301at2759	191178at33208	241143at1206795					934213			
		A27_C_intestinalis_A27_XP_026689626	756301at2759	191178at33208						934213			
		A27_A_planci_A27_A27_XP_022093700	756301at2759	191178at33208						934213			
		A27_D_melanogaster_A27_NP_001188664	756301at2759	191178at33208	10034at6656	1203104at6960				934213			
		A27_C_secundus_A27_XP_023714248	756301at2759	191178at33208	10034at6656	1203104at6960							
		A27_D_ponderosae_A27_A27_XP_0197608	756301at2759	191178at33208	10034at6656	1203104at6960							
		AtAtUCP3_AT1G14140	756301at2759	1406775at33090	1320356at3193					934213	CLU_015166_14_2	TF354328	KOG0753
		A27_O_sativa_A27_XP_015636356	756301at2759	1406775at33090									
		A27_P_patens_A27_XP_024383968	756301at2759	1406775at33090									
		A27_C_reinhardtii_A27_XP_042925196	756301at2759	1406775at33090									
	DIC	HsSLC25A10	448427at2759	2108516at33208	377870at7742					934204	CLU_015166_14_1	TF312920	KOG0759
		A10_G_gallus_A10_XP_001232238	448427at2759	2108516at33208	377870at7742								
		A10_X_tropicalis_A10_NP_001017018	448427at2759	2108516at33208	377870at7742								
		A10_D_rerio_A10_NP_957466	448427at2759	2108516at33208	377870at7742								
		A10_C_elegans_A10_NP_509133	448427at2759	2108516at33208	142824at6231					934204			

		A10_A_californica_A10_XP_005092470	448427at2759	2108516at33208	183945at1206795				934204			
		A10_C_intestinalis_A10_XP_002126004	448427at2759	2108516at33208					934204			
		A10_A_planci_A10_XP_022084885	448427at2759	2108516at33208					934204			
		A10_D_melanogaster_A10_NP_731793	448427at2759	2108516at33208	1847644at6656				934204			
		A10_B_tabaci_A10_XP_018898252	448427at2759	2108516at33208	1847644at6656							
		A10_S_frugiperda_A10_XP_035433060	448427at2759	2108516at33208	1847644at6656							
		A10_A_echinatior_A10_XP_011058217	448427at2759	2108516at33208	1847644at6656				934204			
		A10_C_secundus_A10_XP_023715566	448427at2759	2108516at33208	1847644at6656							
		A10_D_ponderosae_A10_XP_019760268	448427at2759	2108516at33208	1847644at6656							
		AtDIC1_AT2G22500	6703404at2759	45536at33090	1305212at3193				934346	CLU_015166_14_1	TF312920	KOG0759
		AtDIC2_AT4G24570	6703404at2759	45536at33090	1305212at3193				934229	CLU_015166_14_1	TF312920	KOG0759
		AtDIC3_AT5G09470	448427at2759	580134at33090	493715at3193				934326	CLU_015166_14_1	TF312920	KOG0759
		A10_O_sativa_XP_015650890	6703404at2759	45536at33090	1305212at3193				934229			
		A10_P_patens_XP_024368012	6703404at2759	45536at33090	1305212at3193				934204			
		A10_P_patens_XP_024381398	6703404at2759	45536at33090	1305212at3193				934282			
		ScDic1p_YLR348C	448427at2759	3438755at4751	494509at4890				934229	CLU_015166_14_1	TF312920	KOG0759
		A10_N_crassa_A10_XP_956963	448427at2759	3438755at4751								
		A10_A_fumigatus_A10_XP_755633	448427at2759	3438755at4751								
	OGC	HsSLC25A11	448427at2759	2108516at33208	1547595at7742				934206	CLU_015166_14_1	TF354262	KOG0759
		A11_X_tropicalis_A11_NP_001025683	448427at2759	2108516at33208	1547595at7742							
		A11_D_rerio_A11_NP_001002099	448427at2759	2108516at33208	1547595at7742							
		A11_C_elegans_A11_NP_493694	448427at2759	2108516at33208	145122at6231				934206			
		A11_A_californica_A11_XP_005091215	448427at2759	2108516at33208	183776at1206795				934206			
		A11_C_intestinalis_A11_XP_009862380	448427at2759	2108516at33208					934206			
		A11_A_planci_A11_XP_022090410	448427at2759	2108516at33208					934206			
		A11_D_melanogaster_A11_NP_651703	448427at2759	2108516at33208	281285at6656				934206			
		A11_B_tabaci_A11_XP_018898281	448427at2759	2108516at33208	281285at6656							
		A11_S_frugiperda_A11_XP_035443806	448427at2759	2108516at33208	281285at6656							
		A11_A_echinatior_A11_XP_011053892	448427at2759	2108516at33208	281285at6656				934206			
		A11_C_secundus_A11_XP_023717148	448427at2759	2108516at33208	281285at6656							
		A11_D_ponderosae_A11_XP_019771380	448427at2759	2108516at33208	281285at6656				934206			
		AtDTC_AT5G19760	756301at2759	1357927at33090	1271508at3193				934206	CLU_015166_14_1	TF354262	KOG0759
		A11_O_sativa_A11_NP_001067793	756301at2759	1357927at33090					934206			
		A11_P_patens_A11_XP_024398078	756301at2759	1357927at33090								
		A11_C_reinhardtii_A11_XP_001695808_2	756301at2759	1357927at33090								
**MC-AAP**	ALC	HsSLC25A29	193856at2759	4200214at33208	616992at7742				934209	CLU_015166_16_1	TF351739	KOG0762
		A29_G_gallus_A29_XP_421366	193856at2759	4200214at33208	616992at7742							
		A29_X_tropicalis_A29_NP_001096191	193856at2759	4200214at33208	616992at7742							
		A29_D_rerio_A29_NP_001025408	193856at2759	4200214at33208	616992at7742							
		A29_C_elegans_A29_NP_872134	193856at2759	4200214at33208	92673at6231				934209			
		A29_C_intestinalis_A29_XP_002127556_3	193856at2759	4200214at33208					934209			
		A29_A_planci_A29_XP_022095671	193856at2759	4200214at33208					934209			
		A29_D_melanogaster_A29_NP_609380	193856at2759	4200214at33208					934209			
		A29_S_frugiperda_A29_XP_035434522	193856at2759	4200214at33208	373304at6656							
		A29_A_echinatior_A29_XP_011060138	193856at2759	4200214at33208	373304at6656				934260			
		A29_C_secundus_A29_XP_033606387	193856at2759	4200214at33208	373304at6656							
		A29_D_ponderosae_A29_XP_019756683	193856at2759	4200214at33208	373304at6656				934485			
		AtBAC2_AT1G79900	193856at2759	42002at33090	1316122at3193				934209	CLU_015166_16_1	TF351739	KOG0762

		A29_O_sativa_A29_NP_001042456	193856at2759	42002at33090						934209			
		A29_P_patens_A29_XP_024403282	193856at2759	42002at33090						934209			
	SLC25A45/A47	A45/A47/A48_C_elegans_A45_NP_506030	193856at2759	4200214at33208	82802at6231	82802at119089				934217	CLU_015166_16_1		
		A45/A47/A48_A_californica_A47_XP_00510(	193856at2759	4200214at33208	232826at1206795	28265at6447				934368			
		A45/A47/A48_C_intestinalis_A48_XP_01867	193856at2759	4200214at33208						934217			
	(SLC25A45)	HsSLC25A45	193856at2759	4200214at33208	349115at7742	901547at32523	6978at40674			934223	CLU_015166_16_1	TF351739	KOG0758
		A45_X_tropicalis_A45_NP_001106560	193856at2759	4200214at33208	349115at7742	901547at32523				934223	CLU_015166_16_1		
		A45_D_rerio_A45_XP_002662242	193856at2759	4200214at33208	1490389at7742	164348at7898				934223			
	(SLC25A47)	HsSLC25A47	193856at2759	4200214at33208	1490389at7742	884819at32523				934222	CLU_015166_16_1	TF351739	KOG0762
		A47_G_gallus_A47_XP_421367	193856at2759	4200214at33208	1490389at7742	884819at32523				934222			
		A47_X_tropicalis_A47_XP_004917228	193856at2759	4200214at33208	1490389at7742	884819at32523				934222			
		A47_D_rerio_A47_NP_001038779	193856at2759	4200214at33208	1490389at7742	155541at7898				934267			
		A47_D_rerio_A47_NP_001083050	193856at2759	4200214at33208	1490389at7742	155541at7898				934800			
	(SLC25A48)	HsSLC25A48	193856at2759	4200214at33208	349115at7742	901547at32523	6409at40674			934217	CLU_015166_11_2	TF351739	KOG0758
		A48_G_gallus_A48_NP_001376493_1	193856at2759	4200214at33208	349115at7742	901547at32523	30786at8457			934217			
		A48_X_tropicalis_A48_XP_031753840_1	193856at2759	4200214at33208	349115at7742	901547at32523				934217			
		A48_D_rerio_A48_NP_001002367	193856at2759	4200214at33208	349115at7742	349115at7898				934217			
	CAC	HsSLC25A20	14252at2759	4215997at33208	355674at7742					934191	CLU_015166_16_0	TF300894	KOG0758
		A20_G_gallus_A20_XP_414400	14252at2759	4215997at33208									
		A20_X_tropicalis_A20_NP_989099	14252at2759	4215997at33208									
		A20_D_rerio_A20_NP_957153	14252at2759	4215997at33208									
		A20_C_elegans_A20_NP_501223	14252at2759	4215997at33208									
		A20_A_californica_A20_XP_005100578	14252at2759	4215997at33208									
		A20_C_intestinalis_A20_XP_002119607	14252at2759	4215997at33208									
		A20_A_planci_A20_XP_022105337	14252at2759	4215997at33208									
		A20_D_melanogaster_A20_NP_477221	14252at2759	4215997at33208									
		A20_B_tabaci_A20_XP_018908070	14252at2759	4215997at33208									
		A20_S_frugiperda_XP_035443836	14252at2759	4215997at33208									
		A20_A_echinatior_A20_XP_011057778	14252at2759	4215997at33208									
		A20_C_secundus_A20_XP_023722836	14252at2759	4215997at33208									
		A20_D_ponderosae_A20_XP_019754947	14252at2759	4215997at33208									
		ScCrc1p_YOR100C	14252at2759	3496544at4751	1883478at4890					934191	CLU_015166_16_0	TF300894	KOG0758
		A20_N_crassa_A20_XP_962117	14252at2759	3496544at4751									
		A20_A_fumigatus_A20_XP_751284	14252at2759	3496544at4751									
	ORC	HsSLC25A15	409586at2759	4281883at33208	1543931at7742					934208	CLU_015166_16_3	TF314880	KOG0763
		HsSLC25A2	409586at2759	4281883at33208	1543931at7742					934262	CLU_015166_16_3	TF314880	KOG0763
		A15_G_gallus_A15_NP_001008442	409586at2759	4281883at33208									
		A15_X_tropicalis_A15_NP_001090866	409586at2759	4281883at33208									
		A15_D_rerio_A15_NP_001074107	409586at2759	4281883at33208									
		A15_D_rerio_A15_NP_005165234	409586at2759	4281883at33208									
		A15_C_elegans_A15_NP_498094	409586at2759	4281883at33208									
		A15_A_californica_A15_XP_005105018	409586at2759	4281883at33208									
		A15_C_intestinalis_A15_XP_002132089	409586at2759	4281883at33208									
		A15_A_planci_A15_XP_022103059	409586at2759	4281883at33208									
		A15_D_melanogaster_A15_NP_001259423	409586at2759	4281883at33208									
		A15_B_tabaci_A15_XP_018917935	409586at2759	4281883at33208									
		A15_S_frugiperda_A15_XP_035431892	409586at2759	4281883at33208									
		A15_A_echinatior_A15_XP_011051191	409586at2759	4281883at33208									

		A15_C_secundus_A15_XP_023718668	409586at2759	4281883at33208								
		A15_D_ponderosae_A15_XP_019754124	409586at2759	4281883at33208								
		AtBAC1_AT2G33820	14252at2759	1121373at33090	1294756at3193				934208	CLU_015166_16_3	TF351739	KOG0758
		A15_C_reinhardtii_A15_XP_042915245	14252at2759	1121373at33090	27041at3166							
		ScOrt1p_YOR100C	2139348at2759	46450at4751	1514081at4890				934191	CLU_015166_16_0	TF300894	KOG0758
		A15_cerevisiae_ORT1_NP_014773_4	2139348at2759	46450at4751	1514081at4890							
		A15_N_crassa_A15_XP_964458	2139348at2759	46450at4751	1514081at4890							
		A15_A_fumigatus_A15_XP_746931	2139348at2759	46450at4751	1514081at4890							
	YMC/BOU	AtBou_AT5G46800	14252at2759	1121373at33090	1330140at3193				934191	CLU_015166_16_3	TF351739	KOG0758
		YMC1_O_sativa_XP_015614184	14252at2759	1121373at33090	1330140at3193				934223			
		YMC1_P_patens_XP_024359340	14252at2759	1121373at33090	1330140at3193				934191			
		ScYmc1p_YPR058W	409586at2759	3444909at4751	1860820at4890				934267	CLU_015166_16_2	TF351739	KOG0758
		ScYmc2p_YBR104W	409586at2759	3444909at4751	1860820at4890				934222	CLU_015166_16_2	TF351739	KOG0758
		YMC1_S_pombe_NP_593701_2	409586at2759	3444909at4751					934222			
		YMC1_N_crassa_XP_959913	409586at2759	3444909at4751					934217			
		YMC1_A_fumigatus_XP_750402	409586at2759	3444909at4751					934217			
**MC-AAN**												
	AGC	HsSLC25A12	2161at2759	4212963at33208	256741at7742				934195	CLU_014931_3_0	TF313209	KOG0751
		HsSLC25A13	2161at2759	4212963at33208	256741at7742				934291	CLU_014931_3_0	TF313209	KOG0751
		A12_G_gallus_A12_XP_428938	2161at2759	4212963at33208	256741at7742							
		A13_G_gallus_A13_NP_001012967	2161at2759	4212963at33208	256741at7742							
		A13_X_tropicalis_A13_NP_001107969	2161at2759	4212963at33208	256741at7742							
		A12_D_rerio_A12_NP_997947	2161at2759	4212963at33208	256741at7742							
		A12_C_elegans_A12_NP_497274	2161at2759	4212963at33208	142792at6231							
		A13_A_californica_A13_XP_005095774_2	2161at2759	4212963at33208	235259at1206795							
		A12_C_intestinalis_A12_XP_009862417	2161at2759	4212963at33208								
		A12_A_planci_A12_XP_022087095	2161at2759	4212963at33208								
		A12_D_melanogaster_A12_NP_001247368	2161at2759	4212963at33208	175569at6656							
		A13_B_tabaci_A13_XP_018913363	2161at2759	4212963at33208	175569at6656							
		A13_S_frugiperda_A13_XP_035438281	2161at2759	4212963at33208	175569at6656							
		A13_A_echinatior_A12_XP_011053164	2161at2759	4212963at33208	175569at6656							
		A12_C_secundus_A12_XP_023712752	2161at2759	4212963at33208	175569at6656							
		A12_D_ponderosae_A12_XP_019768579	2161at2759	4212963at33208	175569at6656							
		A12_P_patens_A12_XP_024383358	1935185at2759	1441075at33090	1354656at3193							
		ScAgc1p_YPR021C	2161at2759	3498214at4751	1851384at4890				273724	CLU_014931_1_0	TF313209	KOG0751
		A12_N_crassa_A12_XP_961607	2161at2759	3498214at4751	1851384at4890							
		A12_A_fumigatus_A12_KAH1350362	2161at2759	3498214at4751	1851384at4890							
	GC	HsSLC25A18	2382881at2759	142312at33208	1548395at7742				934242	CLU_015166_3_4	TF313209	KOG0750
		HsSLC25A22	2382881at2759	142312at33208	1548395at7742				934233	CLU_015166_3_4	TF313209	KOG0750
		A22_G_gallus_A22_NP_001238997	2382881at2759	142312at33208	1548395at7742							
		A18_X_tropicalis_A18_NP_001096428	2382881at2759	142312at33208	1548395at7742							
		A22_X_tropicalis_A22_NP_001005430	2382881at2759	142312at33208	1548395at7742							
		A18_D_rerio_A18_XP_005174420	2382881at2759	142312at33208	1548395at7742							
		A22_D_rerio_A22b_NP_001003448	2382881at2759	142312at33208	1548395at7742							
		A22_D_rerio_A22a_NP_998573	2382881at2759	142312at33208	1548395at7742							
		A18_C_elegans_A18_NP_510493	2382881at2759	142312at33208	47546at6231							
		A22_C_elegans_A22_NP_501198	2382881at2759	142312at33208	47546at6231							
		A22_A_californica_A22_XP_012935169	2382881at2759	142312at33208	230083at1206795							

		A22_C_intestinalis_A22_XP_002129773	2382881at2759	142312at33208									
		A18_A_planci_A18_XP_022079180	2382881at2759	142312at33208									
		A22_D_melanogaster_A22_NP_731657	2382881at2759	142312at33208	164574at6656								
		A18_D_melanogaster_A18_NP_001262490	2382881at2759	142312at33208	164574at6656								
		A22_B_tabaci_A22_XP_018900680	2382881at2759	142312at33208	164574at6656								
		A22_S_frugiperda_A22A_XP_035453423	2382881at2759	142312at33208	164574at6656								
		A22_S_frugiperda_A22B_XP_035453418	2382881at2759	142312at33208	164574at6656								
		A22_A_echinatior_A22_XP_011067792	2382881at2759	142312at33208	164574at6656								
		A18_C_secundus_A18_XP_023706553	2382881at2759	142312at33208	164574at6656								
		A22_D_ponderosae_A22A_XP_019769026	2382881at2759	142312at33208	164574at6656								
		A22_D_ponderosae_A22B_XP_019769047	6701129at2759										
**OUTSIDE main clusters**													
	SFC	AtSFC1_AT5G01340	1924968at2759	1226465at33090	1279735at3193					934192	CLU_015166_5_0	TF314035	KOG0756
		SFC1_O_sativa_XP_015627972	1924968at2759	1226465at33090	1279735at3193								
		SFC1_P_patens_XP_024387979	1924968at2759	1226465at33090	1279735at3193								
		SFC1_C_reinhardtii_XP_042923487	1924968at2759	1226465at33090	9985at3041								
		ScSfc1p_YJR095W	204711at2759	3503307at4751	1973154at4890					934214	CLU_015166_5_0	TF354298	KOG0756
		SFC1_N_crassa_XP_962756_2	204711at2759	3503307at4751	1973154at4890								
		SFC1_A_fumigatus_XP_756030	204711at2759	3503307at4751	1973154at4890								
	CIC	HsSLC25A1	44467at2759	4266615at33208	1564741at7742					934207	CLU_015166_5_1	TF105786	KOG0756
		A1_G_gallus_A1_XP_415059	44467at2759	4266615at33208	1564741at7742								
		A1_X_tropicalis_A1_NP_989391	44467at2759	4266615at33208	1564741at7742								
		A1_D_rerio_A1A_NP_956901	44467at2759	4266615at33208	1564741at7742								
		A1_D_rerio_A1B_NP_001313457_1	44467at2759	4266615at33208	1564741at7742								
		A1_C_elegans_A1_NP_499187	44467at2759	4266615at33208	71386at6231								
		A1_A_californica_A1_XP_012940275	44467at2759	4266615at33208	228575at1206795								
		A1_C_intestinalis_A1_XP_002130220	44467at2759	4266615at33208									
		A1_A_planci_A1_XP_022105121	44467at2759	4266615at33208									
		A1_D_melanogaster_A1_NP_001027175	44467at2759	4266615at33208	173499at6656								
		A1_B_tabaci_A1_XP_018916557	44467at2759	4266615at33208	173499at6656								
		A1_S_frugiperda_A1_XP_035448019	44467at2759	4266615at33208	173499at6656								
		A1_A_echinatior_A1_XP_011067395	44467at2759	4266615at33208	173499at6656								
		A1_C_secundus_A1_XP_023713316	44467at2759	4266615at33208	173499at6656								
		A1_D_ponderosae_A1_XP_019768401	44467at2759	4266615at33208	173499at6656								
		ScCtp1p_YBR291C	44467at2759	926115at4751	1560928at4890					934207	CLU_015166_5_1	TF105786	KOG0756
		Sc_YFR045W	44467at2759	926115at4751	1560928at4890					934342	CLU_015166_5_1	TF332793	KOG0756
		A1_S_pombe_A1_NP_594420	44467at2759	926115at4751	1560928at4890								
		A1_N_crassa_A1_XP_956064	44467at2759	926115at4751	1560928at4890								
		A1_A_fumigatus_A1_XP_755059_2	44467at2759	926115at4751	1560928at4890								
	ODC	HsSLC25A21	434783at2759	4288279at33208	520345at7742					934192	CLU_015166_5_2	TF314035	KOG0754
		A21_G_gallus_A21_XP_015143145_1	434783at2759	4288279at33208	520345at7742								
		A21_X_tropicalis_A21_NP_001017069	434783at2759	4288279at33208	520345at7742								
		A21_D_rerio_A21_NP_001070100	434783at2759	4288279at33208	520345at7742								
		A21_C_elegans_A21_NP_510638	434783at2759	4288279at33208									
		A21_A_californica_A21_XP_005108207	434783at2759	4288279at33208									
		A21_C_intestinalis_A21_XP_002130378	434783at2759	4288279at33208									
		A21_A_planci_A21_XP_022087672	434783at2759	4288279at33208									
		A21_D_melanogaster_A21_NP_569856	434783at2759	4288279at33208									

		A21_B_tabaci_A21_XP_018901660	434783at2759	4288279at33208									
		A21_S_frugiperda_A21_XP_035437831	434783at2759	4288279at33208									
		A21_A_echinatior_A21_XP_011059760	434783at2759	4288279at33208									
		A21_C_secundus_A21_XP_023706270	434783at2759	4288279at33208									
		A21_D_ponderosae_A21_XP_019757086	434783at2759	4288279at33208									
		ScOdc1p_YPL134C	434783at2759	1339401at4751	1611619at4890					934192	CLU_015166_5_2	TF314035	KOG0754
		ScOdc2p_YOR222W	434783at2759	1339401at4751	1611619at4890					934377	CLU_015166_5_2	TF314035	KOG0754
		A21_S_pombe_A21_NP_594211	434783at2759										
		A21_N_crassa_A21_XP_960396_3	434783at2759										
		A21_A_fumigatus_A21_XP_752333	434783at2759										
	SLC25A39-A4(	HsSLC25A39	1747031at2759	4282969at33208	1545656at7742					934238	CLU_015166_0_0	TF314720	KOG0761
		HsSLC25A40	1747031at2759	4282969at33208	1545656at7742					934212	CLU_015166_0_0	TF314720	KOG0761
		A39_G_gallus_A39_XP_015154854	1747031at2759										
		A40_G_gallus_A40_XP_025004170_2	1747031at2759										
		A39_X_tropicalis_A39_NP_001076818	1747031at2759										
		A40_X_tropicalis_A40_NP_989316	1747031at2759										
		A39_D_rerio_A39_NP_956780	1747031at2759										
		A40_D_rerio_A40_NP_001002360	1747031at2759										
		A40_C_elegans_A40_NP_497836	1747031at2759										
		A40_C_intestinalis_A40_XP_002129093	1747031at2759										
		A40_A_californica_A40_XP_005111116	1747031at2759										
		A40_A_planci_A40_XP_022081975	1747031at2759										
		A40_D_melanogaster_A40_NP_001027073	1747031at2759										
		A40_D_melanogaster_A40_NP_649731	1747031at2759										
		A40_B_tabaci_A40_XP_018907002	1747031at2759										
		A40_S_frugiperda_A40_XP_035458122	1747031at2759										
		A40_A_echinatior_A40_XP_011064747	1747031at2759										
		A40_C_secundus_A40_XP_023707512	1747031at2759										
		A40_D_ponderosae_A40_XP_019755065	1747031at2759										
		At_AT2G46320	1747031at2759	1421353at33090	1334934at3193					934238	CLU_015166_0_1	TF314720	KOG0761
		At_AT4G27940	1747031at2759	1421353at33090	1334934at3193					934323	CLU_015166_0_1	TF314720	KOG0761
		A39_O_sativa_A39_NP_001061710	1747031at2759										
		A40_P_patens_A40_XP_024382471	1747031at2759										
		ScMtm1p_YGR257C	1747031at2759	56132at4751	666103at4890					934212	CLU_015166_0_0	TF314720	KOG0761
		A40_S_pombe_NP_595818	1747031at2759										
		A40_N_crassa_XP_963772	1747031at2759										
		A40_A_fumigatus_A40_XP_755259	1747031at2759										
	GlyC	HsSLC25A38	1924968at2759	2084859at33208	1541871at7742					934201	CLU_015166_0_3	TF332793	KOG0766
		A38_G_gallus_A38_XP_418818	1924968at2759	2084859at33208									
		A38_X_tropicalis_A38_NP_001004921	1924968at2759	2084859at33208									
		A38_D_rerio_A38_NP_001189387	1924968at2759	2084859at33208									
		A38_D_rerio_A38_NP_001070070	1924968at2759	2084859at33208									
		A38_A_californica_A38_XP_005089247	1924968at2759	2084859at33208									
		A38_A_planci_A38_XP_022089331	1924968at2759	2084859at33208									
		A38_B_tabaci_A38_XP_018908440	1924968at2759	2084859at33208									
		A38_S_frugiperda_A38_XP_035432283	1924968at2759	2084859at33208									
		A38_A_echinatior_A38_XP_011066154	1924968at2759	2084859at33208									
		A38_C_secundus_A38_XP_023708209	1924968at2759	2084859at33208									
		A38_D_ponderosae_A38_XP_019761947	1924968at2759	2084859at33208									

		A38_P_patens_A38_XP_024382055	1924968at2759	1226465at33090	1328539at3193					934201			
		ScGlyCp_YDL119C	1924968at2759	1878640at4751	1892634at4890					934201	CLU_015166_0_3	TF332793	KOG0766
		A38_S_pombe_A38_NP_593837	1924968at2759	1878640at4751									
		A38_N_crassa_A38_XP_965360	1924968at2759	1878640at4751									
		A38_A_fumigatus_XP_751597	1924968at2759	1878640at4751									
	SLC25A44	HsSLC25A44	250329at2759	148509at33208	1548548at7742					934216	CLU_015166_3_3	TF354268	KOG0765
		A44_X_tropicalis_A44_NP_001008081	250329at2759										
		A44_D_rerio_A44A_AAH85527	250329at2759										
		A44_D_rerio_A44B_NP_001013537	250329at2759										
		A44_C_elegans_A44_NP_001021104	250329at2759										
		A44_C_elegans_A44_NP_501908	250329at2759										
		A44_A_californica_A44_XP_012946464	250329at2759										
		A44_C_intestinalis_A44_XP_002125555	250329at2759										
		A44_A_planci_A44_XP_022084594	250329at2759										
		A44_D_melanogaster_A44_NP_651284	250329at2759										
		A44_B_tabaci_A44_XP_018906614	250329at2759										
		A44_S_frugiperda_A44_XP_035457630	250329at2759										
		A44_A_echinatior_A44_XP_011056070	250329at2759										
		A44_C_secundus_A44_XP_023717432	250329at2759										
		A44_D_ponderosae_A44_XP_019764188	250329at2759										
		At_AT1G72820	250329at2759	1371950at33090	1321697at3193					934281	CLU_015166_3_3	TF354268	KOG0765
		At_AT5G15640	250329at2759	1371950at33090	1285531at3193					934216	CLU_015166_3_3	TF354268	KOG0765
		At_AT5G26200	250329at2759	1371950at33090	1321697at3193					934302	CLU_015166_3_3	TF354268	KOG0765
		A44_O_sativa_A44_NP_001051308	250329at2759										
		A44_P_patens_A44_XP_024379410	250329at2759										
		A44_C_reinhardtii_A44_XP_042927079	250329at2759										
		A44_AT1G72820_O_sativa_XP_015631823	250329at2759										
		A44_AT1G72820_P_patens_XP_024371403	250329at2759										
		A44_AT5G15640_O_sativa_XP_015630493	250329at2759										
		A44_AT5G15640_P_patens_XP_024379410	250329at2759										
	MFRN	HsSLC25A28	43906at2759	4284301at33208	1546119at7742					934200	CLU_015166_3_1	TF314118	KOG0760
		HsSLC25A37	43906at2759	4284301at33208	1546119at7742					934228	CLU_015166_3_1	TF314118	KOG0760
		A28_G_gallus_A28_XP_421702	43906at2759										
		A37_G_gallus_A37_XP_417682	43906at2759										
		A28_X_tropicalis_A28_NP_001072892	43906at2759										
		A37_X_tropicalis_A37_XP_002932597	43906at2759										
		A28_D_rerio_A28_NP_998284	43906at2759										
		A37_D_rerio_A37_NP_001035060	43906at2759										
		A37_C_elegans_A37_NP_496447	43906at2759										
		A28_A_californica_A28_XP_005099447	43906at2759										
		A37_C_intestinalis_A37_XP_002122098	43906at2759										
		A28_A_planci_A28_XP_022080846	43906at2759										
		A37_D_melanogaster_A37_NP_651600	43906at2759										
		A28_B_tabaci_A28_XP_018910492	43906at2759										
		A37_S_frugiperda_A37_XP_035442485	43906at2759										
		A28_A_echinatior_A28_XP_011069065	43906at2759										
		A37_C_secundus_A37_XP_023724204	43906at2759										
		A37_D_ponderosae_A37_XP_019772752	43906at2759										
		At_AT1G07030	276989at2759	4105at33090	1328425at3193					934228	CLU_015166_3_1	TF314118	KOG0760

		At_AT2G30160	276989at2759	4105at33090	1328425at3193					934300	CLU_015166_3_1	TF314118	KOG0760
		A37_O_sativa_A37_NP_001049833	276989at2759	4105at33090									
		A28_P_patens_A28_XP_024397020	276989at2759	4105at33090									
		A28_C_reinhardtii_A28_XP_042915258	276989at2759	4105at33090									
		ScMrs3p_YJL133W	43906at2759	3492908at4751	1942820at4890					934256	CLU_015166_3_1	TF314118	KOG0760
		ScMrs4p_YKR052C	43906at2759	3492908at4751	1942820at4890					934228	CLU_015166_3_1	TF314118	KOG0760
		A37_S_pombe_NP_594283	43906at2759										
		A37_N_crassa_A37_XP_959605	43906at2759										
		A37_A_fumigatus_A37_XP_751133	43906at2759										
	SAMC	HsSLC25A26	276989at2759	4279314at33208	1556791at7742					934193	CLU_015166_3_0	TF313186	KOG0768
		A26_G_gallus_A26_NP_001264772	276989at2759	4279314at33208	1556791at7742								
		A26_X_tropicalis_A26_NP_001004820	276989at2759	4279314at33208	1556791at7742								
		A26_D_rerio_A26_NP_001025314	276989at2759	4279314at33208	1556791at7742								
		A26_C_elegans_A26_NP_501552	276989at2759	4279314at33208	27729at6231								
		A26_A_californica_XP_005093221	276989at2759	4279314at33208	211283at1206795								
		A26_C_intestinalis_XP_004225486	276989at2759	4279314at33208									
		A26_A_planci_XP_022085246	276989at2759	4279314at33208									
		A26_D_melanogaster_A26_NP_651415	276989at2759	4279314at33208	1921459at6656								
		A26_B_tabaci_A26_XP_018906253	276989at2759	4279314at33208	1921459at6656								
		A26_S_frugiperda_A26_XP_035452078	276989at2759	4279314at33208	1921459at6656								
		A26_A_echinatior_A26_XP_011064681	276989at2759	4279314at33208	1921459at6656								
		A26_C_secundus_A26_XP_023711320	276989at2759	4279314at33208	1921459at6656								
		A26_D_ponderosae_A26_XP_019766846	276989at2759	4279314at33208	1921459at6656								
		AtSAMC1_AT4G39460	276989at2759	1520844at33090	1329525at3193					934193	CLU_015166_3_0	TF313186	KOG0768
		AtSAMC2_AT1G34065	276989at2759	1520844at33090	1329525at3193					934317	CLU_015166_3_0	TF313186	KOG0768
		A26_O_sativa_A26_NP_001055312	276989at2759	1520844at33090	1329525at3193								
		A26_P_patens_A26_XP_024397817	276989at2759	1520844at33090	1329525at3193								
		A26_C_reinhardtii_A26_XP_001695360_2	276989at2759	1520844at33090	12432at3041								
		ScSam5p_YNL003C	276989at2759	3499219at4751	1887565at4890					934193	CLU_015166_3_0	TF313186	KOG0768
		A26_S_pombe_A26_NP_594641	276989at2759	3499219at4751	1887565at4890								
		A26_N_crassa_A26_XP_956205	276989at2759	3499219at4751	1887565at4890								
		A26_A_fumigatus_A26_XP_753466	276989at2759	3499219at4751	1887565at4890								
	SAMCX	At_AT2G26360	276989at2759	1523088at33090	1357485at3193					934372	CLU_015166_3_0	TF332793	KOG0768
		At_AT2G35800	276989at2759	1523088at33090	1357485at3193					669076	CLU_015166_9_2	TF332793	KOG0768
		AT2G35800_O_sativa_XP_015615134	276989at2759	1523088at33090	1357485at3193								
		AT2G35800_P_patens_XP_024380815	276989at2759	1523088at33090	1357485at3193								
	AT5G42130	At_AT5G42130	276989at2759	1525443at33090	1341771at3193					934258	CLU_015166_3_7	TF354268	KOG0768
		AT5G42130_MFL1_O_sativa_XP_01561441	276989at2759	1525443at33090	1341771at3193								
		MFL1_P_patens_XP_024361501	276989at2759	1525443at33090	1341771at3193								
	AT4G11440	At_AT4G11440	10253709at2759	72617at33090	29004at3193					671865	CLU_025765_0_0_	TF313186	KOG0768/K
		AT4G11440_O_sativa_XP_015627693	10253709at2759	72617at33090	29004at3193								
		AT4G11440_P_patens_XP_024401911	10253709at2759	72617at33090	29004at3193								
	PiC	HsSLC25A3	427452at2759	1603835at33208	1491182at7742					934205	CLU_039456_3_1	TF314119	KOG0767
		A3_G_gallus_A3_NP_001006236	427452at2759										
		A3_X_tropicalis_A3_NP_988928	427452at2759										
		A3_D_rerio_A3A_NP_957009	427452at2759										

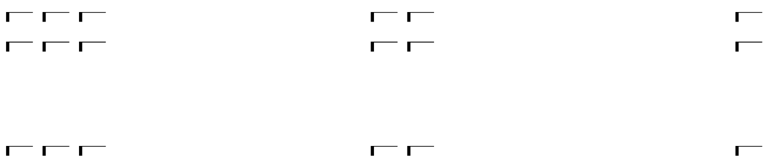

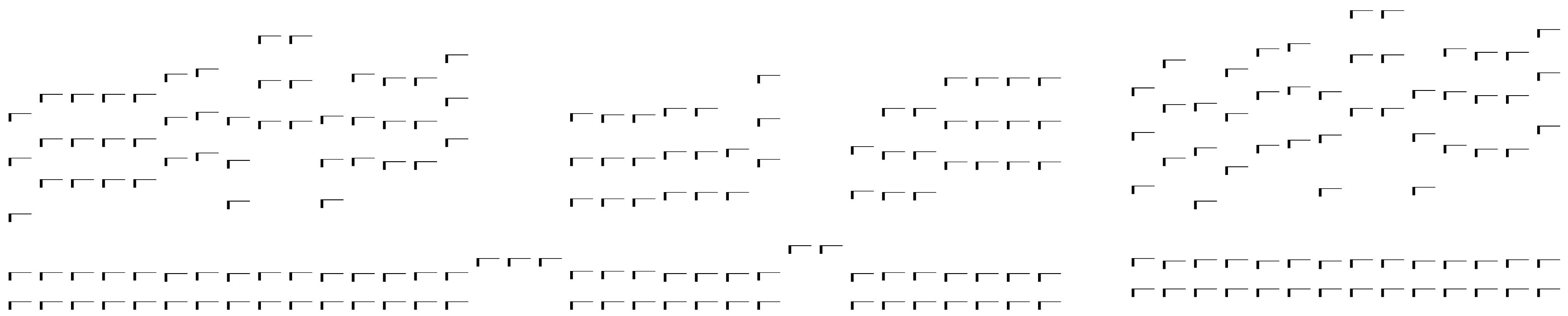

	UGO	ScUgo1p_YDR470C	77989at2759	3463470at4751	1993223at4890					1417767	CLU_029376_0_0		KOG0255
		UGO1_S_pombe_NP_593929	77989at2759										
		UGO1_N_crassa_XP_958084	77989at2759										
		UGO1_A_fumigatus_XP_747953	77989at2759										
	MTCH	HsMTCH1_SLC25A49	10253709at2759	142404at33208	139220at7742					1370621	CLU_058300_1_0	TF313721	KOG2745
		HsMTCH2_SLC25A50	10253709at2759	142404at33208	139220at7742					1370622	CLU_058300_2_0	TF313721	KOG2745
		A50_G_gallus_A50_NP_990139	10253709at2759	142404at33208	139220at7742								
		A50_X_tropicalis_A50_NP_989114	10253709at2759	142404at33208	139220at7742								
		A50_D_rerio_A50_NP_571457	10253709at2759	142404at33208	139220at7742								
		A50_C_elegans_A50_NP_495545	10253709at2759	142404at33208	142404at6231								
		A50_C_intestinalis_A50_XP_026696400	10253709at2759	142404at33208									
		A50_A_californica_A50_XP_005103103	10253709at2759	142404at33208									
		A50_A_planci_A50_XP_022112067	10253709at2759	142404at33208									
		A50_D_melanogaster_A50_NP_728481	10253709at2759	142404at33208									
		A50_B_tabaci_A50_XP_018915792	10253709at2759	142404at33208									
		A50_S_frugiperda_XP_035444972	10253709at2759	142404at33208									
		A50_A_echinatior_A50_XP_011052186	10253709at2759	142404at33208									
		A50_C_secundus_A50_XP_023709165	10253709at2759	142404at33208									
		A50_D_ponderosae_A50_XP_019755553	10253709at2759	142404at33208									
	MCART	HsSLC25A51	2139348at2759	4093182at33208	1435426at7742					1418298	CLU_061821_0_0	TF314192	KOG1519
		HsSLC25A52	2139348at2759	4093182at33208	1435426at7742					1418294		TF314192	KOG1519
		HsSLC25A53	2139348at2759	4093182at33208	1480801at7742					355915	CLU_061821_1_0	TF314192	KOG1519
		A51_G_gallus_A51_XP_003643156	2139348at2759	4093182at33208	1480801at7742								
		A51_X_tropicalis_A51_NP_001107506	2139348at2759	4093182at33208	1480801at7742								
		A53_X_tropicalis_A53_XP_002940455	2139348at2759	4093182at33208	1480801at7742								
		A51_D_rerio_A51_XP_002664343	2139348at2759	4093182at33208									
		A51_D_rerio_A51_XP_684328	2139348at2759	4093182at33208									
		A52_D_rerio_A52_NP_957049	2139348at2759	4093182at33208									
		A51_C_elegans_A51_NP_501642	2139348at2759	4093182at33208									
		A51_A_californica_A52_XP_005113383	2139348at2759	4093182at33208									
		A51_C_intestinalis_A51_XP_009862144	2139348at2759	4093182at33208									
		A51_A_planci_A51_XP_022110290	2139348at2759	4093182at33208									
		A51_D_melanogaster_A51_NP_651766	2139348at2759	4093182at33208									
		A51_B_tabaci_A52_XP_018897075	2139348at2759	4093182at33208									
		A51_S_frugiperda_A51_XP_035430352	2139348at2759	4093182at33208									
		A51_A_echinatior_A51_XP_011060285	2139348at2759	4093182at33208									
		A51_C_secundus_A51_XP_023708196	2139348at2759	4093182at33208									
		A52_D_ponderosae_A52_XP_019759873	2139348at2759	4093182at33208									
	MME	At_AT1G74240	415315at2759	73477at33090	1280301at3193					934230	CLU_015166_3_5	TF354268	KOG0770
		AT1G74240_O_sativa_XP_015630298	415315at2759										
		AT1G74240_P_patens_XP_024361289	415315at2759										
		ScMme1p_YMR166C	415315at2759	3203233at4751	869907at4890					934230	CLU_015166_3_2	TF313186	KOG0770
		MME1_S_pombe_NP_588318	415315at2759										
		MME1_N_crassa_XP_961906_2	415315at2759										
		MME1_A_fumigatus_XP_748970	415315at2759										
	AT4G15010	At_AT4G15010	44467at2759	1386807at33090	1300388at3193					1045005	CLU_046929_0_0		ENOG502Q
		AT4G15010_O_sativa_NP_001406624	44467at2759	1386807at33090									

		AT4G15010_P_patens_XP_024397039	44467at2759	1386807at33090									
	GGC	ScGgc1p_YDL198C	409947at2759	3440182at4751	1872921at4890					934221	CLU_053371_0_0	TF332793	KOG0750
		GGC1_S_pombe_NP_587802	409947at2759										
		GGC1_N_crassa_XP_956124	409947at2759										
		GGC1_A_fumigatus_XP_750491_2	409947at2759										
	YHM2	ScYhm2p_YMR241W	10253709at2759	3444532at4751	1894846at4890					1422930	CLU_052717_0_0	TF332793	KOG0756
		YHM2_S_pombe_NP_595645	10253709at2759	3444532at4751									
		YHM2_N_crassa_XP_011393485	10253709at2759	3444532at4751									
		YHM2_A_fumigatus_XP_747921	10253709at2759	3444532at4751									

TABLE S2. Predictions of the most similar sequence to the MC subfamily exons. The table is divided into sections of MC subfamilies based on the main phylogenetic clusters: (a) MC-NT2; (b) MC-NT1; (c) MC-CA; (d) MC-AAP; (e) MC-AAN; (f) MCs outside the main clusters; and more or less in the order of the phylogenetic tree (Figure S1). The plant subfamily members are indicated with “p” in front of the subfamily name. The exons are numbered as in the alignments for each subfamily (Figure S8). In the subfamily exons column, the OLD exon and full-length sequence set is indicated when the total set is not used in the analysis. The results for each subfamily exon are divided into the columns for the most similar ES, the most similar full-length sequence (FLS) and the final prediction. For details of the calculations of the parameters (subcolumns with a name initiating with “top”) and confidence scores see the Section 4. The number of top ES and FLS hits out of the total for the same exon of all analyzed subfamily members is given in parentheses. The average alignment score (AAS) and average sequence identity (ASI) are given with standard errors of the mean. For the confidence score of the final prediction of the most similar sequence, one point each was given to the hit subfamily with an absolute majority (>50% based on at least four sequence hits) in the four subcolumns with a name initiating with “top”. An additional point was given to subfamilies found exclusively in all four subcolumns, and clear observed trends for final prediction subfamilies not reaching the thresholds of confidence scores are indicated in parentheses. Source**:** * only the most similar ES or FLS prediction was considered when the comparison of their average alignment scores had Chi-square test P-value <0.05; # subfamily hit based on one and the same single sequence hit.

Table S2. Predictions of the most similar sequence to each MC subfamily exon.

a. MC-NT2 cluster subfamilies

NDT subfamily exons	most similar ES				most similar FLS				final prediction
	**top ES hits (number)**	**AAS**	**ASI (%)**	**top 5% ES hits in % of summed AS (number)**	**top FLS hits (number)**	**AAS**	**ASI (%)**	**top 5% FLS hits in % of summed AS (number)**	**subfamily hit (confidence score)**
**1**	ODC (2)<br>MFT/FAD (1)<br>TAAC (1)<br>(4 total)	48±11<br>41<br>37	39±7<br>42<br>37	ODC 55%<br>MFT/FAD 24%<br>TAAC 21%<br>(4 total)	AT5G42130 (1)<br>ORC (1)<br>other 3<br>(5 total)	58<br>52<br>37-42	37<br>32<br>36-47	AT5G42130 25%<br>ORC 22%<br>ER_ANT/TAAC 21%<br>ALC 20%<br>2 other 6%<br>(7 total)	ODC (1)
**1 OLD**	ORC (1)<br>MFT/FAD (1)<br>2 other (1)<br>(4 total)	52<br>41<br>33-34	32<br>42<br>36-42	ORC 33%<br>MFT/FAD 26%<br>2 other 21%<br>(4 total)	ORC (1)<br>ALC (1)<br>2 other (1)<br>(4 total)	52<br>46<br>36-39	32<br>42<br>36-37	ORC 30%<br>ALC 27%<br>2 other ≤ 23%<br>(5 total)	-
**2**	APC (2)<br>PNC (2)<br>PiC (1)<br>(5 total)	83±4<br>74±7<br>78	38±3<br>43±2<br>39	PNC 48%<br>APC 36%<br>PiC 10%<br>MME 7%<br>(10 total)	ODC (1)<br>SLC25A39-A40 (1)<br>PNC (1)<br>other 2<br>(5 total)	84<br>83<br>81<br>71-77	47<br>47<br>42<br>38-44	ODC 21%<br>SLC25A39-A40 21%<br>PNC 21%<br>2 other ≤ 20 %<br>(7 total)	-
**2 OLD**	APC (3)<br>2 other (1)<br>(5 total)	77±8<br>70-78	38±2<br>38-39	APC 49%<br>3 other ≤ 21%<br>(8 total)	SLC25A39-A40 (1)<br>PiC (1)<br>3 other (1)<br>(5 total)	83<br>78<br>71-76	47<br>39<br>34-44	SLC25A39-A40 39%<br>TPC 25%<br>5 other ≤ 10%<br>(11 total)	APC (1)
**3**	APCX (1)<br>ODC (1)<br>APC (1)<br>2 other (1)<br>(5 total)	87<br>86<br>85<br>81-84	40<br>43<br>37<br>39-50	PNC 27%<br>APCX 25%<br>other 6 ≤14%<br>(12 total)	TPC (2)<br>PNC (1)<br>MFT/FAD (1)<br>APC (1)<br>(5 total)	102±7<br>91<br>89<br>85	39±2<br>40<br>43<br>37	TPC 48%<br>PNC 20%<br>7 other ≤ 10%<br>(14 total)	-
**3 OLD**	APC (2)<br>3 other (1)<br>(5 total)	85±1<br>81-85	38±1<br>39-46	TPC 25%<br>5 other ≤ 20%<br>(9 total)	TPC (3)<br>2 other (1)<br>(5 total)	96±11<br>85-89	38±2<br>37-43	TPC 65%<br>MFT/FAD 19%<br>4 other ≤ 5%	TPC (2)

								(11 total)	
**4**	SLC25A39-A40 (1)<br>MFT/FAD (1)<br>3 other<br>(5 total)	62<br>50<br>33-42	71<br>32<br>35-53	SLC25A39-A40 27%<br>MFT/FAD 22%<br>3 other ≤ 18%<br>(6 total)	SLC25A39-A40 (3)<br>MFRN<br>CoC<br>(5 total)	47±12<br>42<br>41	49±15<br>53<br>41	SLC25A39-A40 65%<br>MFRN 18%<br>CoC 18%<br>(5 total)	SLC25A39-A40 (2)
**4 OLD**	MFT/FAD (2)<br>3 other (1)<br>(5 total)	40±10<br>41-62	37±5<br>41-71	SLC25A39-A40 32%<br>MFT/FAD 27%<br>3 other ≤ 19%<br>(7 total)	SLC25A39-A40 (3)<br>MFRN<br>CoC<br>(5 total)	47±12<br>42<br>41	49±15<br>53<br>41	SLC25A39-A40 65%<br>MFRN 18%<br>CoC 18%<br>(5 total)	SLC25A39-A40 (2)
**5**	PNC (3.5)<br>MFT/FAD (1.5)<br>(5 total)	96±5<br>99±8	53±3<br>52±5	PNC 69%<br>MFT/FAD 19%<br>peCFNC 12%<br>(11 total)	MFT/FAD (3)<br>PNC (2)<br>(5 total)	101±9<br>100±4	55±1<br>51±1	MFT/FAD 54%<br>PNC 46%<br>(12 total)	PNC (2)<br>MFT/FAD (2)
**5 OLD**	MFT/FAD (4)<br>peCFNC (1)<br>(5 total)	96±7<br>89	49±5<br>53	MFT/FAD 78%<br>peCFNC 22%<br>(8 total)	MFT/FAD (3)<br>AGC (2)<br>(5 total)	101±9<br>90±4	55±1<br>52±2	MFT/FAD 62%<br>AGC 38%<br>(7 total)	MFT/FAD (4)
**6**	SLC25A44 (3)<br>MFT/FAD (1)<br>SAMC (1)<br>(5 total)	83±4<br>80<br>76	38±3<br>41<br>44	SLC25A44 62%<br>MFT/FAD 20%<br>SAMC 19%<br>(5 total)	YPR011C (2)<br>3 other (1)<br>(5 total)	80±5<br>80	46±2<br>41-50	YPR011C 37%<br>TAAC 20%<br>MFT/FAD 20%<br>2 other ≤ 12%<br>(9 total)	SLC25A44 (2)
**6 OLD**	SLC25A44 (3)<br>MFT/FAD (1)<br>SAMC (1)<br>(5 total)	83±4<br>80<br>76	38±3<br>41<br>44	SLC25A44 62%<br>MFT/FAD 20%<br>SAMC 19%<br>(5 total)	YPR011C (2)<br>MFT/FAD (2)<br>TPC (1)<br>(5 total)	80±5<br>78±2<br>77	46±2<br>40±2<br>41	YPR011C 41%<br>MFT/FAD 40%<br>TPC 19%<br>(10 total)	SLC25A44 (2)
**7**	MFT/FAD (5<br>(5 total)	88±5	55±6	MFT/FAD 100%<br>(6 total)	MFT/FAD (5)<br>(5 total)	84±8	54±5	MFT/FAD 100%<br>(7 total)	MFT/FAD (5)
**7 OLD**	MFT/FAD (5<br>(5 total)	88±5	55±6	MFT/FAD 100%<br>(6 total)	MFT/FAD (5)<br>(5 total)	84±8	54±5	MFT/FAD 100%<br>(7 total)	MFT/FAD (5)
**8**	PNC (4)<br>MFT/FAD (1)<br>(5 total)	53±3<br>48	37±2<br>38	PNC 81%<br>MFT/FAD 19%<br>(5 total)	MFT/FAD (2.5)<br>CoC (2)<br>ALC (0.5)<br>(5 total)	52±1<br>53±2<br>53	36±4<br>41±2<br>41	CoC 41%<br>MFT/FAD 30%<br>PNC 23%<br>ALC 7%<br>(10 total)	PNC (2)
**8 OLD**	peCFNC (2)	48±2	36±4	peCFNC 45%	MFT/FAD (2.5)	52±1	36±4	MFT/FAD 50%	-

	MFT/FAD (2)<br>peANT (1)<br>(5 total)	46±2<br>50	37±1<br>34	MFT/FAD 25%<br>peANT 21%<br>2 other ≤ 4%<br>(9 total)	CoC (2)<br>ALC (0.5)<br>(5 total)	53±2<br>53	41±2<br>41	CoC 40%<br>ALC 10%<br>(7 total)	
**9**	MFT/FAD (2)<br>ALC (1)<br>APC (1)<br>(4 total)	178±6	35±0<br>33<br>32	MFT/FAD 68%<br>ALC 16%<br>3 other ≤ 5%<br>(9 total)	MFT/FAD (3)<br>GC (1)<br>(4 total)	176±8<br>138	36±3<br>30	MFT/FAD 79%<br>GC 21%<br>(10 total)	MFT/FAD (3)
**9 OLD**	MFT/FAD (2)<br>ALC (1)<br>APC (1)<br>(4 total)	178±6	35±0<br>33<br>32	MFT/FAD 68%<br>ALC 16%<br>other 3 ≤5%<br>(9 total)	MFT/FAD (3)<br>(3 total)	176±8	36±3	MFT/FAD 100%<br>(9 total)	MFT/FAD (2)

**PNC subfamily exons**	**most similar ES**				**most similar FLS**				**final prediction**
	**top ES hits (number)**	**AAS**	**ASI (%)**	**top 5% ES hits in % of summed AS (number)**	**top FLS hits (number)**	**AAS**	**ASI (%)**	**top 5% FLS hits in % of summed AS (number)**	**subfamily hit (confidence score)**
**1**	peCFNC (10.5)<br>AGC (2)<br>CoC (2)<br>4 other (≤ 1)<br>(18 total)	42±4<br>34±2<br>33±2<br>26-44	39±4<br>39±2<br>37±0<br>35-50	peCFNC 64%<br>6 other ≤10%<br>(19 total)	ODC (5)#<br>peCFNC (3)<br>TPC (2)<br>APC (2)<br>6 other (1)<br>(18 total)	38±2<br>33±2<br>40±6<br>36±9<br>27-37	36±0<br>38±4<br>39±8<br>41±9<br>32-50	ODC 30%<br>peCFNC 16%<br>TPC 14%<br>10 other ≤9%<br>(25 total)	peCFNC (2)
**1 OLD**	peCFNC (10.5)<br>AGC (2)<br>CoC (2)<br>4 other (≤ 1)<br>(18 total)	42±4<br>34±2<br>33±2<br>26-44	39±4<br>39±2<br>37±0<br>35-50	peCFNC 64%<br>6 other ≤10%<br>(19 total)	peCFNC (8)<br>TPC (2)<br>APC (2)<br>6 other (1)<br>(18 total)	33±1<br>40±6<br>36±9<br>27-37	41±3<br>39±8<br>41±9<br>32-50	peCFNC 44%<br>TPC 15%<br>9 other ≤10%<br>(24 total)	peCFNC (2)
**2**	AGC (2)<br>ALC (1)<br>(3 total)	94±1<br>101	32±0<br>30	-	OAC (3)<br>NDT (2)<br>3 other (1)<br>(8 total)	105±7<br>114±14<br>98-101	32±1<br>31±0<br>30-36	NDT 34%<br>OAC 25%<br>5 other ≤ 12%<br>(11 total)	-
**2 OLD**	AGC (4)<br>ALC (1)<br>(5 total)	82±12<br>*<br>101	33±1<br>30	AGC 76%<br>ALC 24%<br>(5 total)	NDT (3)<br>SAMC (2)<br>ALC (1)	110±14*<br>90±2<br>101	31±1<br>31±0<br>30	NDT 69%<br>SAMC 15%<br>2 other ≤ 8%	NDT (1)

					(6 total)			(10 total)	
**3**	AGC (6.5)<br>APC (2.5)<br>2 other (1)<br>(11 total)	77±4<br>76±2<br>63-84	52±4<br>53±2<br>35-50	AGC 43%<br>APC 36%<br>4 other ≤ 8%<br>(32 total)	AGC (6.5)<br>APC (3.5)<br>SLC25A44 (1)<br>(11 total)	78±5<br>76±2<br>66	54±5<br>54±3<br>46	AGC 39%<br>APC 36%<br>3 other ≤ 14%<br>(26 total)	AGC (2)
**3 OLD**	AGC (6.5)<br>APC (2.5)<br>2 other (1)<br>(11 total)	77±4<br>76±2<br>63-84	52±4<br>53±2<br>35-50	AGC 44%<br>APC 40%<br>3 other ≤ 8%<br>(27 total)	AGC (6.5)<br>APC (3.5)<br>SLC25A44 (1)<br>(11 total)	78±5<br>76±2<br>66	54±5<br>54±3<br>46	AGC 46%<br>APC 41%<br>SLC25A44 8%<br>(21 total)	AGC (2)
**4**	MFRN (4)<br>NDT (2)<br>AGC (2)<br>(8 total)	60±2<br>61±2<br>60±5	34±3<br>37±1<br>44±6	MFRN 50%<br>NDT 25%<br>AGC 25%<br>(9 total)	MFRN (8)<br>SLC25A39-A40<br>(1)<br>(9 total)	73±8<br>65	37±3<br>38	MFRN 90%<br>2 other ≤ 5%<br>(11 total)	MFRN (2)
**4 OLD**	MFRN (4)<br>NDT (2)<br>AGC (2)<br>(8 total)	60±2<br>61±2<br>60±5	34±3<br>37±1<br>44±6	MFRN 50%<br>NDT 25%<br>AGC 25%<br>(9 total)	MFRN (8)<br>SLC25A39-A40<br>(1)<br>(9 total)	73±8<br>65	37±3<br>38	MFRN 90%<br>2 other ≤ 5%<br>(11 total)	MFRN (2)
**5**	MFT/FAD (3)<br>NDT (3)<br>ODC (2)<br>(8 total)	70±9<br>56±0<br>59±2	56±9<br>50±0<br>48±2	MFT/FAD 67%<br>NDT 17%<br>ODC 16%<br>(15 total)	NDT (6)<br>MFT/FAD (2)<br>(8 total)	61±4<br>75±6	45±0<br>66±2	MFT/FAD 50%<br>NDT 47%<br>peCFNC 3%<br>(27 total)	MFT/FAD (2)<br>NDT (1)
**5 OLD**	MFT/FAD (5)<br>NDT (3)<br>(8 total)	65±9<br>56±0	54±8<br>50±0	MFT/FAD 83%<br>NDT 17%<br>(12 total)	NDT (6)<br>MFT/FAD (2)<br>(8 total)	61±4<br>75±6	45±0<br>66±2	MFT/FAD 50%<br>NDT 47%<br>peCFNC 3%<br>(27 total)	MFT/FAD (2)<br>NDT (1)
**6**	NDT (7)<br>ALC (1)<br>(8 total)	13<br>1±36<br>92	33±4<br>41	NDT 88%<br>2 other ≤ 9%<br>(10 total)	NDT (6)<br>2 other (1)<br>(8 total)	145±20<br>81-98	32±2<br>32-38	NDT 86%<br>3 other ≤ 8%<br>(11 total)	NDT (4)
**6 OLD**	NDT (7)<br>ALC (1)<br>(8 total)	13<br>1±36<br>92	33±4<br>41	NDT 88%<br>2 other ≤ 9%<br>(10 total)	NDT (6)<br>2 other (1)<br>(8 total)	145±20<br>76-95	32±2<br>35-38	NDT 93%<br>2 other ≤ 5%<br>(12 total)	NDT (4)
**7**	NDT (4)<br>TPC (3)<br>MFRN (2)	134±1<br>144±3<br>145±2	32±2<br>39±1<br>35±1	TPC 43%<br>NDT 27%<br>2 other ≤ 23%	ODC (5)<br>MFRN (2)<br>NDT (2)	147±9<br>150±2<br>137±3	40±1<br>37±1<br>34±0	ODC 46%<br>NDT 24%<br>MFRN 20%	-

	(9 total)			(17 total)	MFT/FAD (1)<br>(10 total)	161	32	2 other ≤ 5%<br>(16 total)	
**7 OLD**	NDT (4)<br>TPC (3)<br>MFRN (2)<br>(9 total)	134±1<br>144±3<br>145±2	32±2<br>39±1<br>35±1	TPC 43%<br>NDT 27%<br>2 other ≤ 23%<br>(17 total)	TPC (3)<br>NDT (3)<br>MFRN (2)<br>MFT/FAD (2)<br>(10 total)	144±3<br>136±3<br>150±2<br>147±14	39±1<br>34±0<br>37±1<br>33±1	TPC 33%<br>NDT 29%<br>MFRN 21%<br>2 other ≤ 11%<br>(19 total)	-

**MFT/FAD subfamily exons**	**most similar ES**				**most similar FLS**				**final prediction**
	**top ES hits (number)**	**AAS**	**ASI (%)**	**top 5% ES hits in % of summed AS (number)**	**top FLS hits (number)**	**AAS**	**ASI (%)**	**top 5% FLS hits in % of summed AS (number)**	**subfamily hit (confidence score)**
**1**	TPC (8)<br>BT (2)<br>CAC (2)<br>2 other (1)<br>(14 total)	90±7<br>100±7<br>85±1<br>99-103	34±4<br>40±10<br>37±6<br>38-43	TPC 52%<br>CAC 17%<br>BT 15%<br>2 other ≤ 8%<br>(19 total)	TPC (3.5)<br>TAAC (3)<br>7 other (≤ 1)<br>(13 total)	94±11<br>104±9<br>89-114	35±5<br>32±1<br>31-43	TPC 32%<br>TAAC 13%<br>8 other ≤ 12%<br>(19 total)	TPC (2)
**1 OLD**	TPC (13)<br>2 other (1)<br>(15 total)	89±7<br>93-99	34±4<br>38	TPC 83%<br>3 other ≤ 8%<br>(23 total)	TPC (6.5)<br>SLC25A44 (2)<br>ALC (1.5)<br>3 other (1)<br>(13 total)	93±9<br>100±15<br>86±4<br>84-100	34±4<br>34±0<br>32±0<br>37-44	TPC 50%<br>SLC25A44 17%<br>4 other ≤ 11%<br>(16 total)	TPC (2)
**2**	NDT (5)<br>UCP4 (2.5)<br>5 other (≤ 1)<br>(12 total)	111±5<br>113±7<br>107-123	34±1<br>35±2<br>31-39	NDT 35%<br>UCP4 20%<br>8 other ≤ 13%<br>(20 total)	NDT (3)<br>SLC25A48 (2)<br>CoC (2)<br>UCP4 (2)<br>3 other (1)<br>(12 total)	117±6<br>133±8<br>123±2<br>122±2<br>123-127	34±2<br>38±4<br>39±1<br>33±1<br>30-37	NDT 25%<br>CoC 23%<br>UCP4 17%<br>6 other ≤ 13%<br>(26 total)	-
**2 OLD**	NDT (6)<br>UCP4 (3)<br>3 other (1)<br>(12 total)	111±5<br>113±7<br>111-116	34±2<br>35±2<br>31-38	NDT 39%<br>UCP4 25%<br>4 other ≤ 11%<br>(16 total)	NDT (5)<br>CoC (3)<br>UCP4 (3)<br>UCP2-3 (1)<br>(12 total)	120±7<br>123±1<br>122±2<br>126	37±3<br>39±1<br>32±1<br>30	NDT 36%<br>CoC 34%<br>UCP4 22%<br>3 other ≤ 3%<br>(21 total)	(NDT/CoC)
**3**	MFRN (2)<br>4 other (1)	48±7<br>35-47	34±4<br>33-38	SAMC 27%<br>MFRN 26%	MFRN (2.5)<br>5 other (≤ 1)	50±6<br>37-59	33±4<br>30-38	MFRN 36%<br>GlyC 18%	-

	(6 total)			3 other ≤ 18%<br>(7 total)	(7 total)			4 other ≤ 14%<br>(8 total)	
**3 OLD**	MFRN (2)<br>3 other (1)<br>(5 total)	48±7<br>36-47	34±4<br>34-38	SAMC 32%<br>MFRN 31%<br>2 other ≤ 21%<br>(6 total)	MFRN (3)<br>APC (2)<br>2 other (1)<br>(7 total)	50±6<br>39±2<br>47-59	33±4<br>34±3<br>30-38	MFRN 45%<br>APC 23%<br>2 other ≤ 18%<br>(7 total)	-
**4**	peCFNC (6)#<br>2 other (1)<br>(8 total)	128±8<br>121	36±2<br>34-43	peCFNC 64%<br>4 other ≤ 12%<br>(10 total)	NDT (6)<br>2 other (1)<br>(8 total)	129±11<br>128-146	40±2<br>34-35	NDT 80%<br>2 other ≤ 13%<br>(16 total)	peCFNC (2)#<br>NDT (2)
**4 OLD**	peCFNC (6)#<br>2 other (1)<br>(8 total)	128±8<br>112-121	36±2<br>36-43	peCFNC 70%<br>2 other ≤ 18%<br>(10 total)	NDT (7)<br>peCFNC (1)<br>(8 total)	129±10<br>146	39±2<br>34	NDT 93%<br>peCFNC 7%<br>(13 total)	peCFNC (2)#<br>NDT (2)
**5**	NDT (10)<br>peCFNC (1)<br>(11 total)	82±4<br>80	46±6<br>42	NDT 91%<br>peCFNC 9%<br>(19 total)	NDT (9)<br>peCFNC (2)<br>(11 total)	81±5<br>8±1	41±3<br>39±3	NDT 77%<br>peCFNC 23%<br>(18 total)	NDT (4)
**5 OLD**	NDT (10)<br>peCFNC (1)<br>(11 total)	82±4<br>80	46±6<br>42	NDT 91%<br>peCFNC 9%<br>(19 total)	NDT (9)<br>peCFNC (2)<br>(11 total)	81±5<br>8±1	41±3<br>39±3	NDT 77%<br>peCFNC 23%<br>(18 total)	NDT (4)
**6**	PNC (8)<br>APC (2)<br>NDT (2)<br>(12 total)	114±11<br>100±8<br>94±3	33±2<br>36±1<br>32±1	PNC 64%<br>APC 18%<br>NDT 16%<br>CoC 2%<br>(16 total)	NDT (8)<br>PNC (3)<br>peCFNC (1)<br>(12 total)	118±9<br>125±7<br>122	37±3<br>35±1<br>45	NDT 65%<br>PNC 26%<br>peCFNC 9%<br>(15 total)	PNC (2)<br>NDT (2)
**6 OLD**	NDT (6)<br>APC (3)<br>MFRN (2)<br>CoC (1)<br>(12 total)	96±8<br>100±6<br>92±7<br>80	33±3<br>32±4<br>34±1<br>30	NDT 48%<br>APC 27%<br>2 other ≤ 16%<br>(18 total)	NDT (11)<br>peCFNC (1)<br>(12 total)	116±9<br>122	36±4<br>45	NDT 91%<br>peCFNC 9%<br>(17 total)	NDT (2)
**7**	APC (6)<br>NDT (3)<br>3 other (1)<br>(12 total)	114±4<br>106±7<br>110-131	46±4<br>40±5<br>34-41	APC 58%<br>NDT 24%<br>4 other ≤ 11%<br>(30 total)	APC (5)<br>NDT (3)<br>4 other (1)<br>(12 total)	97±4<br>106±7<br>92-121	47±2<br>40±5<br>33-41	APC 49%<br>NDT 27%<br>8 other ≤ 9%<br>(30 total)	APC (1)

**7 OLD**	APC (6)<br>NDT (3)<br>3 other (1)<br>(12 total)	114±4<br>106±7<br>110-131	46±4<br>40±5<br>34-41	APC 59%<br>NDT 24%<br>3 other ≤ 11%<br>(29 total)	APC (5)<br>NDT (3)<br>4 other (1)<br>(12 total)	97±4<br>106±7<br>90-121	47±2<br>40±5<br>35-41	APC 50%<br>NDT 28%<br>6 other ≤ 9%<br>(29 total)	APC (1)

**pMFT/FAD subfamily exons**	**most similar ES**				**most similar FLS**				**final prediction**
	**top ES hits (number)**	**AAS**	**ASI (%)**	**top 5% ES hits in % of summed AS (number)**	**top FLS hits (number)**	**AAS**	**ASI (%)**	**top 5% FLS hits in % of summed AS (number)**	**subfamily hit (confidence score)**
**1**	TPC (1.5)<br>GC (1)<br>CoC (0.5)<br>(3 total)	89±14<br>75-97	38±2<br>36-46	TPC 56%<br>GC 35%<br>2 other ≤ 5%<br>(8 total)	AT5G42130 (1)<br>GC (1)<br>ODC (1)<br>(3 total)	109<br>108<br>88	46<br>42<br>39	AT5G42130 36%<br>GC 35%<br>ODC 29%<br>(4 total)	TPC (1)
**1 OLD**	TPC (1.5)<br>AGC (1)<br>CoC (0.5)<br>(3 total)	89±14<br>75-96	38±2<br>36-41	TPC 56%<br>AGC 35%<br>2 other ≤ 5%<br>(8 total)	NDT (2)<br>SLC25A44 (1)<br>(3 total)	86±10<br>104	43±1<br>36	NDT 87%<br>SLC25A44 13%<br>(6 total)	TPC (1)<br>NDT (1)
**2**	SFC (1)<br>AAC (1)<br>(2 total)	62<br>51	42<br>48	-	SFC (1)<br>A34-S35 (1)<br>(2 total)	65<br>48	42<br>48	-	-
**2 OLD**	SFC (1)<br>AAC (1)<br>(2 total)	62<br>51	42<br>48	-	SFC (1)<br>OGC (1)<br>(2 total)	65<br>46	42<br>32	-	-
**3**	AAC (2)<br>(2 total)	69±2	56±2	-	ORC (2)<br>(2 total)	82±2	48±2	ORC 51%<br>AAC 49%<br>(4 total)	ORC (1)
**3 OLD**	AAC (2)<br>(2 total)	69±2	56±2	-	ORC (2)<br>(2 total)	82±2	48±2	ORC 51%<br>AAC 49%<br>(4 total)	ORC (1)
**4**	MFRN (2)<br>(2 total)	46±5	32±1	-	PNC (1)<br>SAMC (1)<br>(2 total)	58<br>44	38<br>39	-	-
**4 OLD**	MFRN (2)	46±5	32±1	-	MFRN (1)	50	31	-	-

	(2 total)				SAMC (1)<br>(2 total)	44	39		
**5**	PNC (2)<br>(2 total)	83±3	44±6	PNC 100%<br>(4 total)	OAC (2)<br>(2 total)	94±6	44±0	-	PNC (1)
**5 OLD**	NDT (2)<br>(2 total)	70±2	34±0	NDT 68%<br>peCFNC 32%<br>(4 total)	NDT (2)<br>(2 total)	89±5	42±2	-	NDT (1)
**6**	YPR011C (1)<br>APCX (1)<br>(2 total)	88<br>75	63<br>58	-	YPR011C (1)<br>ODC (1)<br>(2 total)	78<br>74	63<br>50	-	-
**6 OLD**	YPR011C (1)<br>SLC25A39-A40 (1)<br>(2 total)	88<br>72	63<br>50	-	YPR011C (1)<br>SLC25A39-A40 (1)<br>(2 total)	78<br>72	63<br>50	-	-
**7**	peCFNC (1)<br>(1 total)	59	34	-	NDT (2)<br>(2 total)	64±11	35±2	-	-
**7 OLD**	peCFNC (1)<br>(1 total)	59	34	-	NDT (2)<br>(2 total)	64±11	35±2	-	-
**8**	peCFNC (1)<br>(1 total)	77	48	-	NDT (1)<br>(1 total)	69	45	NDT 60%<br>peCFNC 20%<br>CoC 20%<br>(5 total)	NDT (1)
**8 OLD**	peCFNC (1)<br>(1 total)	77	48	-	NDT (1)<br>(1 total)	69	45	NDT 60%<br>peCFNC 20%<br>CoC 20%<br>(5 total)	NDT (1)
**9**	MFRN (1)<br>SLC25A45/A47/A48<br>(1)<br>(2 total)	39<br>39	35<br>30	-	MFRN (1)<br>2 other (0.5)<br>(2 total)	39<br>35	35<br>30	MFRN 53%<br>AGC 31%<br>CAC 16%<br>(4 total)	MFRN (1)

**9 OLD**	MFRN (1)<br>AGC (1)<br>(2 total)	39<br>34	35<br>30	-	MFRN (1)<br>AGC (1)<br>(2 total)	39<br>35	35<br>30	-	-
**10**	APC (1)<br>NDT (1)<br>(2 total)	119<br>112	57<br>37	APC 84%<br>NDT 16%<br>(4 total)	APC (1)<br>NDT (1)<br>(2 total)	120<br>112	57<br>37	APC 84%<br>NDT 16%<br>(4 total)	APC (2)
**10 OLD**	APC (1)<br>NDT (1)<br>(2 total)	119<br>112	57<br>37	APC 84%<br>NDT 16%<br>(4 total)	APC (1)<br>NDT (1)<br>(2 total)	120<br>112	57<br>37	APC 84%<br>NDT 16%<br>(4 total)	APC (2)

**peCFNC subfamily exons**	**most similar ES**				**most similar FLS**				**final prediction**
	**top ES hits (number)**	**AAS**	**ASI (%)**	**top 5% ES hits in % of summed AS (number)**	**top FLS hits (number)**	**AAS**	**ASI (%)**	**top 5% FLS hits in % of summed AS (number)**	**subfamily hit (confidence score)**
**1**	PNC (5)<br>MFT/FAD (2)<br>MFRN (2)<br>SFC (1)<br>(10 total)	41±4<br>39±0<br>38±6<br>26	39±4<br>42±2<br>39±6<br>32	PNC 54%<br>MFT/FAD 23%<br>MFRN 20%<br>SFC 3%<br>(20 total)	MFRN (3)<br>CoC (2)<br>4 other (1)<br>(9 total)	38±5<br>41±4<br>35-52	38±5<br>32±0<br>37-44	MFRN 28%<br>CoC 23%<br>5 other ≤ 15%<br>(10 total)	PNC (1)
**1 OLD**	MFRN (5)<br>MFT/FAD (3)<br>SFC (1)<br>(8 total)	36±5<br>37±3<br>26	37±4<br>43±2<br>32	MFRN 57%<br>MFT/FAD 39%<br>SFC 4%<br>(13 total)	MFRN (3)<br>MFT/FAD (3)<br>CoC (2)<br>2 other (1)<br>(10 total)	38±5<br>36±2<br>41±4<br>36-52	38±5<br>40±6<br>32±0<br>37-38	MFRN 29%<br>MFT/FAD 28%<br>CoC 21%<br>2 other ≤ 13%<br>(10 total)	MFRN (2)
**2**	SAMC (5)<br>UCP2-3 (4)<br>YPR011C (1)<br>(10 total)	65±3<br>58±2<br>67	42±6<br>48±0<br>57	SAMC 52%<br>UCP2-3 37%<br>YPR011C 11%<br>(15 total)	peANT (3)<br>UCP2-3 (3)<br>4 other (1)<br>(10 total)	59±5<br>54±0<br>51-61	57±4<br>52±0<br>48-62	peANT 32%<br>UCP2-3 29%<br>5 other ≤ 11%<br>(12 total)	SAMC (1)
**2 OLD**	SAMC (5)<br>UCP2-3 (4)	65±3<br>58±2	42±6<br>48±0	SAMC 52%<br>UCP2-3 37%	peANT (3)<br>UCP2-3 (3)	59±5<br>54±0	57±4<br>52±0	peANT 31%<br>UCP2-3 29%	SAMC (1)

	YPR011C (1)<br>(10 total)	67	57	YPR011C 11%<br>(15 total)	5 other (≤ 1)<br>(10 total)	51-60	48-62	5 other ≤ 11%<br>(11 total)	
**3**	OGC (3.5)<br>PNC (1.5)<br>3 other (1)<br>(8 total)	40±1<br>47±9<br>36-52	37±2<br>41±5<br>36-41	OGC 44%<br>PNC 20%<br>4 other ≤ 15%<br>(12 total)	TPC (2)<br>OGC (2)<br>4 other (1)<br>(8 total)	41±4<br>37±3<br>38-51	39±2<br>43±2<br>41-45	TPC 25%<br>OGC 22%<br>4 other ≤ 17%<br>(10 total)	-
**3 OLD**	OGC (5)<br>3 other (1)<br>(8 total)	39±1<br>36-52	37±2<br>36-41	OGC 60%<br>TPC 16%<br>2 other ≤ 14%<br>(12 total)	OGC (4)<br>TPC (2)<br>CoC (2)<br>(8 total)	37±2<br>41±4<br>41±1	41±3<br>39±2<br>39±2	OGC 48%<br>CoC 27%<br>TPC 26%<br>(11 total)	OGC (2)
**4**	peANT (1)<br>AAC (1)<br>MFT/FAD (1)<br>(3 total)	86<br>76<br>76	33<br>37<br>31	-	AGC (5)<br>4 other (≤ 1)<br>(5 total)	83±1<br>66-97	31±0<br>31-33	AGC 41%<br>peANT 24%<br>3 other ≤ 19%<br>(6 total)	-
**4 OLD**	peANT (1)<br>AAC (1)<br>MFT/FAD (1)<br>(3 total)	86<br>76<br>76	33<br>37<br>31	-	AGC (5)<br>4 other (≤ 1)<br>(5 total)	83±1<br>66-97	31±0<br>31-33	AGC 41%<br>peANT 24%<br>3 other ≤ 19%<br>(6 total)	-
**5**	MFT/FAD (7)<br>PNC (1)<br>(8 total)	83±8<br>74	33±1<br>31	MFT/FAD 89%<br>PNC 11%<br>(8 total)	MFT/FAD (5)<br>NDT (3)<br>(8 total)	84±14<br>86±4	36±4<br>32±1	MFT/FAD 71%<br>NDT 25%<br>SFC 4%<br>(12 total)	MFT/FAD (4)
**5 OLD**	MFT/FAD (7)<br>(7 total)	83±8	33±1	MFT/FAD 100%<br>(7 total)	MFT/FAD (5)<br>NDT (3)<br>(8 total)	84±14<br>86±4	36±4<br>32±1	MFT/FAD 71%<br>NDT 25%<br>SFC 4%<br>(12 total)	MFT/FAD (4)
**6**	BT (3)<br>SLC25A39-A40 (2)<br>MFT/FAD (1)<br>(6 total)	106±5<br>94±4<br>108	39±2<br>32±1<br>41	BT 35%<br>MFT/FAD 26%<br>SLC25A39-A40 26%<br>2 other ≤ 8%	MFT/FAD (3)<br>3 other (1)<br>(6 total)	115±3<br>107-117	41±1<br>38-43	MFT/FAD 53%<br>NDT 21%<br>2 other ≤ 17%<br>(10 total)	MFT/FAD (1)
**6 OLD**	MFT/FAD (3)<br>SLC25A39-A40 (2)<br>TPC (1)<br>(6 total)	103±4<br>94±4<br>99	40±1<br>32±1<br>38	MFT/FAD 60%<br>SLC25A39-A40 32%<br>TPC 8%<br>(9 total)	MFT/FAD (3)<br>3 other (1)<br>(6 total)	115±3<br>107-117	41±1<br>38-43	MFT/FAD 53%<br>NDT 21%<br>2 other ≤ 17%<br>(10 total)	MFT/FAD (2)
**7**	MFT/FAD (2)<br>peANT (2)	76±1<br>71±2	50±2<br>44±3	MFT/FAD 36%<br>peANT 33%	peANT (4)<br>MFT/FAD (2)	74±4<br>77±5	42±3<br>48±0	peANT 60%<br>MFT/FAD 34%	peANT (2)

	2 other (1)<br>(6 total)	66-6<br>8	38-44	4 other ≤ 8%<br>(11 total)	(6 total)			2 other ≤ 3%<br>(13 total)	
**7 OLD**	peANT (3)<br>MFT/FAD (2)<br>TPC (1)<br>(6 total)	70±2<br>76±1<br>66	44±3<br>50±2<br>44	peANT 40%<br>MFT/FAD 36%<br>2 other ≤ 16%<br>(9 total)	peANT (4)<br>MFT/FAD (2)<br>(6 total)	74±4<br>77±5	42±3<br>48±0	peANT 62%<br>MFT/FAD 34%<br>TPC 4%<br>(13 total)	peANT (2)
**8**	AACX (1)<br>A34-APC3 (1)<br>3 other (1)<br>(5 total)	35<br>29<br>23-28	32<br>30<br>30-33	AACX 25%<br>OAC 21%<br>3 other ≤ 20%<br>(5 total)	YPR011C (2)<br>5 other (≤ 1)<br>(6 total)	30±4<br>29-35	32±1<br>30-43	YPR011C 32%<br>AACX 19%<br>4 other ≤ 16%<br>(7 total)	-
**8 OLD**	MFT/FAD (1)<br>APC (1)<br>AAC (1)<br>(3 total)	28<br>23<br>19	33<br>30<br>33	MFT/FAD 40%<br>APC 33%<br>2 other ≤ 13%<br>(4 total)	YPR011C (2)<br>3 other (1)<br>(5 total)	30±4<br>24-30	32±1<br>30-43	YPR011C 41%<br>3 other ≤ 21%<br>(5 total)	-
**9**	peANT (7)<br>2 other (1)<br>(9 total)	99±6<br>82-85	39±4<br>30	peANT 80%<br>2 other ≤ 10%<br>(14 total)	peANT (7)<br>AT4G11440 (2)<br>(9 total)	98±6<br>82±3	38±3<br>33±1	peANT 76%<br>4 other ≤ 9%<br>(19 total)	peANT (4)
**9 OLD**	peANT (8)<br>SLC25A16 (1)<br>(9 total)	96±10<br>85	38±4<br>30	peANT 90%<br>SLC25A16 10%<br>(14 total)	peANT (7)<br>SAMC (2)<br>(9 total)	98±6<br>79±4	38±3<br>31±1	peANT 85%<br>3 other ≤ 10%<br>(22 total)	peANT (4)

ppeCFNC subfamily exons	most similar ES				most similar FLS				final prediction
	**top ES hits (number)**	**AAS**	**ASI (%)**	**top 5% ES hits in % of summed AS (number)**	**top FLS hits (number)**	**AAS**	**ASI (%)**	**top 5% FLS hits in % of summed AS (number)**	**subfamily hit (confidence score)**
**1**	SLC25A16 (2)<br>TPC (1)<br>(3 total)	58±5<br>67	43±2<br>34	TPC 51%<br>SLC25A16 49%<br>(4 total)	APCX (2)<br>32 (1)<br>(3 total)	62±2<br>76	44±0<br>32	APCX 47%<br>MFT/FAD 39%<br>TPC 14%<br>(4 total)	TPC (1)
**1 OLD**	SLC25A16 (2)<br>TPC (1)<br>(3 total)	58±5<br>67	43±2<br>34	TPC 51%<br>SLC25A16 49%<br>(4 total)	TPC (2)<br>MFT/FAD (1)<br>(3 total)	55±3<br>76	46±2<br>32	-	TPC (1)
**2**	GC (1)<br>(1 total)	30	36	-	CoC (1)<br>(1 total)	27	33	-	-
**2 OLD**	MFRN (1)<br>(1 total)	26	31	-	CoC (1)<br>2 other (0.5)<br>(2 total)	27<br>26	33<br>31	-	-
**3**	PNC (2)<br>2 other (0.5)<br>(3 total)	75±6<br>66	58±6<br>48	PNC 70%<br>2 other ≤ 20%<br>(5 total)	PNC (1)<br>MFT/FAD (1)<br>BT (1)<br>(3 total)	72<br>69<br>68	64<br>52<br>44	PNC 35%<br>MFT/FAD 33%<br>BT 32%<br>(4 total)	PNC (1)
**3 OLD**	AGC (2.5)<br>MFT/FAD (0.5)<br>(3 total)	68±5<br>66	49±5<br>48	AGC 89%<br>MFT/FAD 11%<br>(6 total)	MFT/FAD (2)<br>SLC25A39-A40 (1)<br>(3 total)	71±2<br>61	48±4<br>40	MFT/FAD 69%<br>SLC25A39-A40 16%<br>PiC 15%<br>(5 total)	AGC (1)<br>MFT/FAD (1)
**4**	-	-	-	-	SLC25A39-A40 (1)<br>(1 total)	84	30	-	-
**4 OLD**	-	-	-	-	SLC25A39-A40 (1)<br>(1 total)	84	30	-	-
**5**	MFT/FAD (1)<br>PNC (1)<br>NDT (1)<br>(3 total)	62<br>59<br>55	67<br>56<br>55	-	MFT/FAD (2)<br>PNC (1)<br>(3 total)	55±2<br>55	56±0<br>50	NDT 55%<br>MFT/FAD 28%<br>PNC 17%<br>(7 total)	NDT (1)

**5 OLD**	NDT (2)<br>MFT/FAD (1)<br>(3 total)	57±2<br>62	50±0<br>67	-	MFT/FAD (2)<br>NDT (1)<br>(3 total)	55±2<br>54	56±0<br>50	NDT 72%<br>MFT/FAD 28%<br>(6 total)	NDT (1)
**6**	-	-	-	-	-	-	-	-	-
**6 OLD**	-	-	-	-	-	-	-	-	-
**7**	SFC (2)<br>CAC (1)<br>(3 total)	67±8<br>70	47±3<br>52	CAC 56%<br>SFC 44%<br>(8 total)	TPC (3)<br>(3 total)	77±7	47±6	-	CAC (1)
**7 OLD**	SFC (2)<br>ORC (1)<br>(3 total)	67±8<br>62	47±3<br>48	SFC 69%<br>2 other ≤ 15%<br>(4 total)	TPC (3)<br>(3 total)	77±7	47±6	-	SFC (1)
**8**	PNC (1)<br>(1 total)	51	33	-	PNC (1)<br>MFT/FAD (1)<br>(2 total)	51<br>47	33<br>35	-	-
**8 OLD**	-	-	-	-	MFT/FAD (2)<br>(2 total)	44±4	34±1	-	-
**10**	MFT/FAD (2)<br>(2 total)	98±2	33±1	-	MFT/FAD (1)<br>(1 total)	111	30	-	-
**10 OLD**	MFT/FAD (2)<br>(2 total)	98±2	33±1	-	MFT/FAD (2)<br>(2 total)	102±10	30±0	MFT/FAD 67%<br>peANT 23%<br>SLC25A39-A40 11%<br>(5 total)	MFT/FAD (1)

**peANT subfamily exons**	**most similar ES**				**most similar FLS**				**final prediction**
	**top ES hits (number)**	**AAS**	**ASI (%)**	**top 5% ES hits in % of summed AS (number)**	**top FLS hits (number)**	**AAS**	**ASI (%)**	**top 5% FLS hits in % of summed AS (number)**	**subfamily hit (confidence score)**
**1**	peCFNC (3)<br>TPC (1)<br>(4 total)	87±12<br>70	32±2<br>37	peCFNC 79%<br>TPC 21%<br>(4 total)	peCFNC (4)<br>(4 total)	98±5	33±1	peCFNC 100%<br>(5 total)	peCFNC (4)
**1 OLD**	peCFNC (3)<br>TPC (1)	87±12<br>70	32±2<br>37	peCFNC 79%<br>TPC 21%	peCFNC (4)<br>(4 total)	98±5	33±1	peCFNC 100%<br>(5 total)	peCFNC (4)

	(4 total)			(4 total)					
**2**	-	-	-	-	SLC25A39-A40 (1)<br>(1 total)	93	30	-	-
**2 OLD**	-	-	-	-	SLC25A39-A40 (1)<br>(1 total)	93	30	-	-
**3**	peCFNC (1)<br>(1 total)	61	31	-	peCFNC (4)<br>(4 total)	96±3	38±4	peCFNC 94%<br>MFT/FAD 6%<br>(10 total)	peCFNC (2)
**3 OLD**	peCFNC (1)<br>(1 total)	61	31	-	peCFNC (4)<br>(4 total)	96±3	38±4	peCFNC 94%<br>MFT/FAD 6%<br>(10 total)	peCFNC (2)
**4**	peCFNC (4)<br>(4 total)	79±11	38±7	peCFNC 100%<br>(6 total)	peCFNC (4)<br>(4 total)	101±10	34±3	peCFNC 100%<br>(6 total)	peCFNC (5)
**4 OLD**	peCFNC (4)<br>(4 total)	79±11	38±7	peCFNC 100%<br>(6 total)	peCFNC (4)<br>(4 total)	101±10	34±3	peCFNC 100%<br>(6 total)	peCFNC (5)
**5**	peCFNC (3)<br>MFT/FAD (1)<br>(4 total)	99±6<br>112	38±5<br>36	peCFNC 72%<br>MFT/FAD 28%<br>(6 total)	-	-	-	-	peCFNC (2)
**5 OLD**	peCFNC (3)<br>MFT/FAD (1)<br>(4 total)	99±6<br>112	38±5<br>36	peCFNC 72%<br>MFT/FAD 28%<br>(6 total)	-	-	-	-	peCFNC (2)

b. MC-NT1 cluster subfamilies

**AAC subfamily exons**	**most similar ES**				**most similar FLS**				**final prediction**
	**top ES hits (number)**	**AAS**	**ASI (%)**	**top 5% ES hits in % of summed AS (number)**	**top FLS hits (number)**	**AAS**	**ASI (%)**	**top 5% FLS hits in % of summed AS (number)**	**subfamily hit (confidence score)**
**1**	CoC (13)<br>(13 total)	109±7	50±5	CoC 100%<br>(20 total)	YPR011C (7)<br>CoC (6)<br>(13 total)	109±6<br>108±2	52±5<br>51±4	YPR011C 61%<br>CoC 39%<br>(25 total)	CoC (2)<br>YPR011C (2)
**1 OLD**	CoC (13)<br>(13 total)	109±7	50±5	CoC 100%<br>(20 total)	YPR011C (7)<br>CoC (6)	109±6<br>108±2	52±5<br>51±4	YPR011C 61%<br>CoC 39%	CoC (2)<br>YPR011C (2)

					(13 total)			(25 total)	
**2**	(A23-A25)				CoC (2)<br>(2 total)	270±3	31±0	-	-
**2 OLD**	(A23-A25)				CoC (2)<br>APC (1)<br>(3 total)	270±3<br>264	31±0<br>30	-	-
**3**	TAAC (1)<br>CoC (1)<br>(2 total)	62<br>62	34<br>32	-	CoC (2)<br>(2 total)	88±14	34±2	-	-
**3 OLD**	CoC (3)<br>TPC (1)<br>(4 total)	61±2<br>56	30±1<br>30	CoC 76%<br>TPC 24%<br>(4 total)	CoC (4)<br>(4 total)	78±14	34±2	CoC 81%<br>TPC 19%<br>(5 total)	CoC (4)
**4**	OAC (6)<br>ORC (3) #<br>2 other (1)<br>(11 total)	105±3<br>123±2 #<br>100-106	34±3<br>34±1 #<br>33-35	OAC 52%<br>ORC 31% #<br>4 other ≤ 6%<br>(20 total)	ORC (4) #<br>APC (3.5)<br>SLC25A44 (2) #<br>YPR011C (1.5) #<br>(11 total)	126±2 #<br>112±1<br>109±1 #<br>112±2 #	33±1 #<br>37±3<br>33±0 #<br>32±1 #	ORC 40% #<br>APC 37%<br>7 other ≤ 7%<br>(26 total)	OAC (2)
**4 OLD**	AGC (5) #<br>ORC (3)<br>NDT (2) #<br>(10 total)	95±6 #<br>123±2<br>100±1 #	34±1 #<br>34±1<br>38±1 #	AGC 37% #<br>ORC 36%<br>NDT 27% #<br>(12 total)	ORC # (4)<br>APC (3.5)<br>SLC25A44 (2)<br>YPR011C (1.5)<br>(11 total)	126±2 #<br>112±1<br>109±1 #<br>112±2 #	33±1 #<br>37±3<br>33±0 #<br>32±1 #	APC 41%<br>ORC 40% #<br>4 other ≤ 10%<br>(19 total)	-

**New mammalian isoform of AAC: SLC25A31 exons**	**most similar ES**				**most similar FLS**				**final prediction**
	**top ES hits (number)**	**AAS**	**ASI (%)**	**top 5% ES hits in % of summed AS (number)**	**top FLS hits (number)**	**AAS**	**ASI (%)**	**top 5% FLS hits in % of summed AS (number)**	**subfamily hit (confidence score)**
**1**	AAC1 (4)<br>(4 total)	273±2*	60±2	AAC1 100%<br>(4 total)	AAC3 (3)<br>AAC1 (1)<br>(4 total)	290±7*<br>287	60±2<br>63	AAC3 51%<br>AAC1 33%<br>AAC2 16%<br>(31 total)	AAC3 (2)

**2**	AAC1 (4)<br>(4 total)	218±2	85±2	AAC1 65%<br>AAC3 27%<br>AAC2 8%<br>(19 total)	AAC1 (4)<br>(4 total)	218±2	85±2	AAC1 67%<br>AAC3 25%<br>AAC2 8%<br>(19 total)	AAC1 (4)
**3**	AAC1 (4)<br>(4 total)	205±2	86±1	AAC1 70%<br>AAC2 25%<br>AAC3 5%<br>(27 total)	AAC1 (4)<br>(4 total)	209±2	86±1	AAC1 68%<br>AAC2 20%<br>AAC3 12%<br>(34 total)	AAC1 (4)
**4**	AAC2 (2)<br>AAC1 (1)<br>AAC3 (1)<br>(4 total)	240±1<br>238<br>240	74±1<br>76<br>74±1	AAC1 42%<br>AAC2 38%<br>AAC3 21%<br>(21 total)	AAC2 (2)<br>AAC3 (2)<br>(4 total)	230±1<br>231±1	74±1<br>74±1	AAC1 37%<br>AAC2 28%<br>AAC3 35%<br>(29 total)	(AAC)
**5**	AAC1 (4)<br>(4 total)	174±4	72±2	AAC1 84%<br>AAC2 8%<br>AAC3 8%<br>(9 total)	AAC3 (4)<br>(4 total)	185±5	79±2	AAC3 57%<br>AAC1 43%<br>(16 total)	AAC1 (2)<br>AAC3 (2)
**6**	AAC1 (2)<br>AAC2 (1)<br>AAC3 (1)<br>(4 total)	202±2<br>203<br>203	55±2<br>56<br>53	AAC2 52%<br>AAC1 33%<br>AAC3 14%<br>(10 total)	AAC3 (3)<br>AAC1 (1)<br>(4 total)	211±4<br>214	56±1<br>50	AAC3 67%<br>AAC1 17%<br>AAC2 16%<br>(9 total)	AAC3 (2)<br>AAC2 (1)

**pAAC subfamily exons**	**most similar ES**				**most similar FLS**				**final prediction**
	**top ES hits (number)**	**AAS**	**ASI (%)**	**top 5% ES hits in % of summed AS (number)**	**top FLS hits (number)**	**AAS**	**ASI (%)**	**top 5% FLS hits in % of summed AS (number)**	**subfamily hit (confidence score)**
**1**	-	-	-	-	-	-	-	-	(YPR011C)
**1 OLD**	-	-	-	-	-	-	-	-	(YPR011C)
**2**	-	-	-	-	-	-	-	-	-
**2 OLD**	-	-	-	-	-	-	-	-	-
**3**	-	-	-	-	-	-	-	-	-
**3 OLD**	-	-	-	-	-	-	-	-	-

TAAC subfamily exons	most similar ES				most similar FLS				final prediction
	top ES hits (number)	AAS	ASI (%)	top 5% ES hits in % of summed AS (number)	top FLS hits (number)	AAS	ASI (%)	top 5% FLS hits in % of summed AS (number)	subfamily hit (confidence score)
**1**	CoC (2)<br>APCX (1)<br>(3 total)	133±10<br>97	41±4<br>32	CoC 74%<br>APCX 13%<br>APC 13%<br>(4 total)	-	-	-	-	CoC (1)
**1 OLD**	CoC (2)<br>APC (1)<br>(3 total)	133±10<br>96	41±4<br>41	CoC 86%<br>APC 13%<br>(6 total)	-	-	-	-	CoC (1)
**2**	APC (2)<br>SAMC (1)<br>OAC (1)<br>(4 total)	32±3<br>28<br>26	35±3	APC 54%<br>SAMC 24%<br>OAC 22%<br>(4 total)	UCP4 (2)<br>3 other (1)<br>(5 total)	28±3<br>24-26	35±3<br>32	UCP4 42%<br>4 other ≤20%<br>(6 total)	APC (1)
**2 OLD**	APC (2)<br>SAMC (2)<br>(4 total)	32±3<br>26±2	35±3<br>32±0	APC 55%<br>SAMC 45%<br>(4 total)	UCP4 (2)<br>PiC (1)<br>(3 total)	28±3<br>24	35±3<br>32	-	APC (1)
**3**	TPC (4)<br>peCFNC (1)<br>(5 total)	64±2<br>66	54±5<br>43	TPC 79%<br>peCFNC 21%<br>(10 total)	TPC (4)<br>UCP2-3 (1)<br>(5 total)	63±2<br>69	53±6<br>57	TPC 75%<br>UCP2-3 22%<br>OGC 3%<br>(18 total)	TPC (4)
**3 OLD**	TPC (4)<br>peCFNC (1)<br>(5 total)	64±2<br>66	54±5<br>43	TPC 79%<br>peCFNC 21%<br>(10 total)	TPC (4)<br>UCP2-3 (1)<br>(5 total)	63±2<br>69	53±6<br>57	TPC 75%<br>UCP2-3 22%<br>OGC 3%<br>(18 total)	TPC (4)
**4**	YPR011C (2)<br>CoC (1)<br>APCX (1)<br>(5 total)	67±2<br>66±1<br>63	66±3<br>53±0<br>53	YPR011C 51%<br>CoC 40%<br>APCX 9%<br>(12 total)	CoC (5)<br>(5 total)	59±2	58±4	CoC 76%<br>YPR011C 24%<br>(19 total)	YPR011C (1)<br>CoC (2)
**4 OLD**	YPR011C (3)<br>CoC (1)<br>APCX (1)	65±3<br>66±1<br>63	63±4<br>53±0<br>53	YPR011C 50%<br>CoC 50%<br>(10 total)	CoC (5)<br>(5 total)	59±2	58±4	CoC 76%<br>YPR011C 24%<br>(19 total)	YPR011C (1)<br>CoC (2)

	(5 total)								
**5**	YPR011C (4)<br>APC (1)<br>(5 total)	57±5<br>53	45±3<br>43	YPR011C 81%<br>APC 19%<br>(5 total)	YPR011C (4)<br>AACX (1)<br>(5 total)	68±7<br>68	46±3<br>39	YPR011C 82%<br>2 other ≤ 10%<br>(7 total)	YPR011C (4)
**5 OLD**	YPR011C (4)<br>APC (1)<br>(5 total)	57±5<br>53	45±3<br>43	YPR011C 81%<br>APC 19%<br>(5 total)	YPR011C (5)<br>(5 total)	68±6	46±3	YPR011C 100%<br>(5 total)	YPR011C (4)
**6**	CoC (2)<br>3 other<br>(5 total)	65±2<br>53-60	50±2<br>43	CoC 43%<br>YPR011C 20%<br>APC 18%<br>2 other ≤ 10%<br>(6 total)	CoC (3)<br>YPR011C (1)<br>APC (1)<br>(5 total)	62±3<br>60<br>56	48±4<br>43<br>52	CoC 55%<br>APC 25%<br>YPR011C 20%<br>(8 total)	CoC (2)
**6 OLD**	CoC (2)<br>3 other<br>(5 total)	65±2<br>53-60	50±2<br>43	CoC 43%<br>YPR011C 20%<br>APC 18%<br>2 other ≤ 10%<br>(6 total)	CoC (3)<br>YPR011C (1)<br>APC (1)<br>(5 total)	62±3<br>60<br>56	48±4<br>43<br>52	CoC 55%<br>APC 25%<br>YPR011C 20%<br>(8 total)	CoC (2)
**7**	APC (5)<br>(5 total)	110±5	44±3	APC 95%<br>BT (5%)<br>(9 total)	BT (3)<br>APCX (2)<br>(5 total)	105±6<br>109±5	45±0<br>41±2	APCX 50%<br>BT 39%<br>APC 11%<br>(15 total)	APC (2)<br>BT (1)
**7 OLD**	APC (5)<br>(5 total)	110±5	44±3	APC 100%<br>(8 total)	YPR011C (2)<br>APC (2)<br>TPC (1)<br>(5 total)	106±1<br>100±1<br>95	42±0<br>46±1<br>37	APC 40%<br>TPC 26%<br>2 other ≤ 22%<br>(11 total)	APC (2)
**8**	CoC (2)<br>TAAC (1)<br>(3 total)	98±3<br>84	32±2<br>38	-	CoC (3)<br>TPC (1)<br>(4 total)	94±6<br>86	32±3<br>30	CoC 77%<br>TPC 23%<br>(4 total)	CoC (2)
**8 OLD**	CoC (2)<br>(2 total)	98±3	32±2	-	CoC (3)<br>TPC (1)<br>(4 total)	94±6<br>86	32±3<br>30	CoC 77%<br>TPC 23%<br>(4 total)	CoC (2)
**9**	APC (5)<br>(5 total)	82±5	62±2	APC 100%<br>(5 total)	APC (5)<br>(5 total)	82±5	62±2	APC 100%<br>(5 total)	APC (5)
**9 OLD**	APC (5)<br>(5 total)	82±5	62±2	APC 100%<br>(5 total)	APC (5)<br>(5 total)	82±5	62±2	APC 100%<br>(5 total)	APC (5)

**10**	BTL (1)<br>MFRN (1)<br>(2 total)	35<br>28	32<br>31	-	GC (1)<br>BTL (1)<br>(2 total)	37<br>35	31<br>32	-	-
**10 OLD**	MFRN (1)<br>(2 total)	28	31	-	MFRN (1)<br>(1 total)	28	31	-	-

**AACX subfamily exons**	**most similar ES**				**most similar FLS**				**final prediction**
	**top ES hits (number)**	**AAS**	**ASI (%)**	**top 5% ES hits in % of summed AS (number)**	**top FLS hits (number)**	**AAS**	**ASI (%)**	**top 5% FLS hits in % of summed AS (number)**	**subfamily hit (confidence score)**
**1**	-	-	-	-	-	-	-	-	-
**1 OLD**	-	-	-	-	-	-	-	-	-
**2**	YPR011C (2)<br>APC (1)<br>(3 total)	191±6<br>173	32±1<br>31	YPR011C 69%<br>APC 31%<br>(4 total)	APCX (1)<br>TAAC (1)<br>BT (1)<br>(3 total)	240<br>221<br>209	33<br>31<br>35	APCX 44%<br>BT 32%<br>TAAC 24%<br>(6 total)	YPR011C (1)
**2 OLD**	YPR011C (2)<br>APC (1)<br>(3 total)	191±6<br>173	32±1<br>31	YPR011C 69%<br>APC 31%<br>(4 total)	APC (2)<br>YPR011C (1)<br>(3 total)	211±2<br>185	32±0<br>31	APC 61%<br>YPR011C 31%<br>CoC 8%<br>(8 total)	YPR011C (1)<br>APC (1)
**3**	BT (1)<br>(1 total)	186	33	-	-	-	-	-	-
**3 OLD**	-	-	-	-	-	-	-	-	-
**4**	APC (2.7)<br>YPR011C (0.3)<br>(3 total)	66±1<br>65	59±8<br>48	APC 91%<br>other 2 ≤ 5%<br>(11 total)	APC (3)<br>(3 total)	66±1	59±8	APC 100%<br>(9 total)	APC (2)
**4 OLD**	APC (2.7)<br>YPR011C (0.3)<br>(3 total)	66±1<br>65	59±8<br>48	APC 91%<br>other 2 ≤ 5%<br>(11 total)	APC (3)<br>(3 total)	66±1	59±8	APC 100%<br>(9 total)	APC (2)

SLC25A43 subfamily exons	most similar ES				most similar FLS				final prediction
	**top ES hits (number)**	**AAS**	**ASI (%)**	**top 5% ES hits in % of summed AS (number)**	**top FLS hits (number)**	**AAS**	**ASI (%)**	**top 5% FLS hits in % of summed AS (number)**	**subfamily hit (confidence score)**
**1**	YPR011C (3)<br>(3 total)	157±20*	32±1	YPR011C 100%<br>(3 total)	CoC (3)<br>TAAC (1)<br>(4 total)	183±21*<br>189	35±5<br>33	CoC 47%<br>TAAC 45%<br>APCX 8%<br>(7 total)	CoC (1)
**1 OLD**	YPR011C (3)<br>(3 total)	157±20*	32±1	YPR011C 100%<br>(3 total)	CoC (3)<br>YPR011C (1)<br>(4 total)	183±21*<br>150	35±5<br>30	CoC 79%<br>YPR011C 21%<br>(4 total)	CoC (2)
**2**	SFC (3)<br>CoC (1)<br>(4 total)	171±7*<br>165	34±1<br>33	SFC 78%<br>CoC 22%<br>(5 total)	CoC (2)<br>APCX (2)<br>(4 total)	206±7*<br>194±3	37±6<br>37±1	CoC 52%<br>APCX 48%<br>(6 total)	CoC (1)
**2 OLD**	SFC (3)<br>CoC (1)<br>(4 total)	171±7*<br>165	34±1<br>33	SFC 78%<br>CoC 22%<br>(5 total)	CoC (3)<br>YPR011C (1)<br>(4 total)	197±15*<br>180	37±5<br>35	CoC 66%<br>YPR011C 23%<br>(4 total)	CoC (2)
**3**	APCX (1)<br>CoC (1)<br>AGC (1)<br>(3 total)	104<br>98<br>88	30<br>32<br>33	CoC 49%<br>APCX 36%<br>AGC 15%<br>(4 total)	APCX (1)<br>TPC (1)<br>CoC (1)<br>TAAC (1)<br>GC (1)<br>(5 total)	114<br>106<br>102<br>101<br>93	32<br>30<br>33<br>30<br>30	APCX 22%<br>CoC 20%<br>TAAC 20%<br>GC 18%<br>other 3 ≤7%<br>(7 total)	-
**3 OLD**	CoC (1)<br>AGC (1)<br>(2 total)	98<br>88	32<br>33	CoC 53%<br>AGC 24%<br>CoC 23%<br>(3 total)	CoC (2)<br>TPC (1)<br>(3 total)	100±3<br>106	33±1<br>30	CoC 65%<br>other 3 ≤12%<br>(5 total)	CoC (1)
**4**	AAC (3)<br>APC (2)<br>(5 total)	96±1<br>109±1	36±2<br>41±2	AAC 50%<br>APC 50%<br>(11 total)	AAC (2)<br>APC (2)<br>OAC (1)<br>(5 total)	108±2<br>109±1<br>85	37±1<br>39±2<br>30	AAC 53%<br>APC 35%<br>other 2 ≤ 7%<br>(9 total)	AAC (2)
**4 OLD**	AAC (3)<br>APC (2)	96±1<br>109±1	36±2<br>41±2	AAC 50%<br>APC 50%	AAC (3)<br>APC (2)	100±11<br>109±1	36±2<br>39±2	AAC 58%<br>APC 35%	AAC (3)

	(5 total)			(9 total)	(5 total)			YPR011C 7%<br>(9 total)	
**5**	-	-	-	-	-	-	-	-	-
**5 OLD**	-	-	-	-	-	-	-	-	-

**APCX subfamily exons**	**most similar ES**				**most similar FLS**				**final prediction**
	**top ES hits (number)**	**AAS**	**ASI (%)**	**top 5% ES hits in % of summed AS (number)**	**top FLS hits (number)**	**AAS**	**ASI (%)**	**top 5% FLS hits in % of summed AS (number)**	**subfamily hit (confidence score)**
**1**	APC (4)<br>CoC (2)<br>(6 total)	110±8<br>109±6	53±2<br>37±0	APC 78%<br>CoC 22%<br>(11 total)	CoC (1)<br>YPR011C (1)<br>(2 total)	104<br>97	30<br>32	-	APC (2)
**1 OLD**	APC (4)<br>CoC (2)<br>(6 total)	110±8<br>109±6	53±2<br>37±0	APC 78%<br>CoC 22%<br>(11 total)	CoC (1)<br>YPR011C (1)<br>(2 total)	104<br>97	30<br>32	-	APC (2)
**2**	AACX (4)<br>YPR011C (2)<br>(6 total)	124±4<br>121±6	36±1<br>33±1	AACX 75%<br>3 other ≤ 14%<br>(9 total)	TAAC (2)<br>AACX (2)<br>CoC (2)<br>(6 total)	138±4<br>132±2<br>129±8	41±2<br>37±1<br>36±2	TAAC 54%<br>CoC 25%<br>AACX 21%<br>(12 total)	AACX (2)<br>TAAC (1)
**2 OLD**	YPR011C (3)<br>APC (2)<br>TPC (1)<br>(6 total)	121±5<br>111±4<br>101	33±1<br>34±1<br>32	YPR011C 41%<br>APC 34%<br>4 other ≤ 11%<br>(16 total)	CoC (4)<br>APC (1)<br>(5 total)	128±6<br>108	35±2<br>37	CoC 73%<br>APC 27%<br>(9 total)	CoC (2)
**3**	YPR011C (3)<br>CoC (2)<br>TPC (1)<br>(6 total)	112±5<br>117±0<br>109	41±1<br>38±0<br>34	YPR011C 38%<br>CoC 37%<br>TPC 25%<br>(9 total)	CoC (4)<br>2 other (1)<br>(6 total)	118±1<br>121-123	42±2<br>40-42	YPR011C 36%<br>CoC 28%<br>SLC25A43 25%<br>1 other ≤ 11%<br>(12 total)	CoC (1)
**3 OLD**	YPR011C (3)<br>CoC (2)<br>TPC (1)<br>(6 total)	112±5<br>117±0<br>109	41±1<br>38±0<br>34	YPR011C 38%<br>CoC 37%<br>TPC 25%<br>(9 total)	CoC (4)<br>2 other (1)<br>(6 total)	118±1<br>113-123	42±2<br>40-42	YPR011C 37%<br>CoC 37%<br>3 other ≤ 11%<br>(12 total)	CoC (1)

**4**	SLC25A43 (4)<br>2 other (1)<br>(6 total)	90±4<br>78-90	43±3<br>41-47	SLC25A43 57%<br>4 other ≤ 17%<br>(9 total)	UCP2-3 (2)<br>4 other (1)<br>(6 total)	86±4<br>90-104	46±2<br>47-50	SLC25A45/A47/A48 28%<br>UCP2-3 23%<br>6 other ≤ 16%<br>(11 total)	SLC25A43 (2)
**4 OLD**	MFT/FAD (2)<br>SFC (2)<br>2 other (1)<br>(6 total)	88±0<br>82±5<br>75-90	50±0<br>49±2<br>41-44	MFT/FAD 35%<br>SFC 25%<br>3 other ≤ 18%<br>(8 total)	MFT/FAD (2)<br>UCP2-3 (2)<br>2 other (1)<br>(6 total)	94±5<br>86±4<br>84-88	49±2<br>46±2<br>47	MFT/FAD 37%<br>UCP2-3 25%<br>4 other ≤17%<br>(8 total)	-
**5**	TPC (3)<br>MFRN (2)<br>2 other (0.5)<br>(6 total)	68±3<br>62±1<br>63	35±2<br>44±2<br>50	TPC 52%<br>MFRN 32%<br>2 other 8%<br>(7 total)	TPC (3)<br>MFRN (2)<br>2 other (0.5)<br>(6 total)	68±4<br>62±1<br>63	35±2<br>44±2<br>50	TPC 45%<br>4 other ≤19%<br>(12 total)	TPC (1)
**5 OLD**	TPC (3)<br>MFRN (2)<br>2 other (0.5)<br>(6 total)	68±3<br>62±1<br>63	35±2<br>44±2<br>50	TPC 52%<br>MFRN 32%<br>2 other 8%<br>(7 total)	TPC (3)<br>MFRN (2)<br>2 other (0.5)<br>(6 total)	68±4<br>62±1<br>63	35±2<br>44±2<br>50	TPC 45%<br>3 other ≤ 20%<br>(12 total)	TPC (1)
**6**	APC (4)<br>CoC (2)<br>(6 total)	48±2<br>42±2	43±5<br>35±5	APC 72%<br>CoC 17%<br>3 other ≤ 7%<br>(12 total)	UCP5-6 (3)<br>APC (2)<br>TPC (1)<br>(6 total)	50±2<br>50±1<br>51	47±2<br>46±2<br>35	UCP5-6 50%<br>APC 42%<br>TPC 8%<br>(9 total)	APC (2)
**6 OLD**	APC (4)<br>CoC (2)<br>(6 total)	48±2<br>42±2	43±5<br>35±5	APC 72%<br>CoC 25%<br>TPC 4%<br>(11 total)	APC (2)<br>CoC (2)<br>2 other (1)<br>(6 total)	50±1<br>43±3<br>40-51	46±2<br>42±3<br>35-39	APC 45%<br>CoC 31%<br>2 other ≤ 15%<br>(9 total)	APC (2)
**7**	BT (3)<br>APC (2)<br>TPC (1)<br>(6 total)	168±6*<br>183±2<br>168	33±1<br>34±1<br>35	APC 46%<br>TPC 27%<br>BT 19%<br>2 other ≤ 3%<br>(15 total)	APC (5)<br>YPR011C (1)<br>(6 total)	190±8*<br>186	38±1<br>34	APC 71%<br>YPR011C 25%<br>UCP4 4%<br>(12 total)	APC (2)
**7 OLD**	APC (4)<br>TPC (2)<br>(6 total)	173±11*<br>169±1	33±1<br>35±0	APC 56%<br>TPC 32%<br>2 other ≤ 4%<br>(11 total)	APC (5)<br>YPR011C (1)<br>(6 total)	190±8*<br>186	38±1<br>34	APC 71%<br>YPR011C 25%<br>UCP4 4%<br>(12 total)	APC (4)

APC subfamily exons	most similar ES				most similar FLS				final prediction
	**top ES hits (number)**	**AAS**	**ASI (%)**	**top 5% ES hits in % of summed AS (number)**	**top FLS hits (number)**	**AAS**	**ASI (%)**	**top 5% FLS hits in % of summed AS (number)**	**subfamily hit (confidence score)**
**1**	CoC (12)<br>APCX (10)<br>TAAC (1)<br>(23 total)	100±4<br>111±6<br>97	48±1<br>53±3<br>59	APCX 51%<br>CoC 37%<br>3 other ≤ 10%<br>(47 total)	APCX (9)<br>CoC (7)<br>TAAC (4)<br>2 other (≤ 2)<br>(23 total)	108±5<br>101±3<br>101±3<br>95-102	53±2<br>46±4<br>43±2<br>49-54	APCX 39%<br>CoC 26%<br>3 other ≤ 18%<br>(49 total)	CoC (1)<br>APCX (1)
**1 OLD**	CoC (23)<br>(23 total)	99±4	44±3	CoC 99%<br>YPR011C 1%<br>(38 total)	CoC (19)<br>YPR011C (4)<br>(23 total)	99±4<br>98±2	46±3<br>50±4	CoC 79%<br>YPR011C 21%<br>(43 total)	CoC (4)
**2**	APCX (7)<br>YPR011C (7)<br>AAC (1)<br>(15 total)	116±3<br>113±4<br>86	36±3<br>36±3<br>31	YPR011C 49%<br>APCX 47%<br>4 other ≤ 2%<br>(31 total)	YPR011C (7)<br>APCX (4)<br>2 other (≤ 2)<br>(14 total)	112±4<br>123±7<br>108-127	38±2<br>38±1<br>35-43	YPR011C 44%<br>APCX 30%<br>CoC 20%<br>2 other ≤ 3%<br>(31 total)	(YPR011C/<br>APCX)
**2 OLD**	YPR011C (12)<br>3 other (1)<br>(15 total)	111±4<br>86-113	37±3<br>31-35	YPR011C 86%<br>4 other ≤ 5%<br>(26 total)	YPR011C (8)<br>CoC (4)<br>3 other (1)<br>(15 total)	113±5<br>115±7<br>96-113	39±2<br>38±3<br>31-37	YPR011C 66%<br>CoC 27%<br>5 other ≤ 2%<br>(31 total)	YPR011C (4)
**3**	BT (5)<br>AACX (3)<br>6 other (1)<br>(14 total)	72±7<br>74±3<br>53-78	37±1<br>35±3<br>31-39	BT 32%<br>AACX 30%<br>5 other ≤ 17%<br>(21 total)	CoC (8)<br>APCX (2)<br>4 other (1)<br>(14 total)	78±6<br>83±5<br>58-90	40±5<br>39±3<br>31-42	CoC 55%<br>APCX 15%<br>4 other ≤ 10%<br>(18 total)	CoC (2)
**3 OLD**	YPR011C (5)<br>CoC (5)<br>AAC (3)<br>1 other (1)<br>(14 total)	66±5<br>65±4<br>58±8<br>67	33±3<br>37±4<br>31±1<br>31	CoC 54%<br>YPR011C 29%<br>2 other ≤ 13%<br>(25 total)	CoC (11)<br>3 other (1)<br>(14 total)	77±6<br>58-90	40±5<br>31-42	CoC 80%<br>3 other ≤ 8%<br>(16 total)	CoC (3)
**4**	CoC (8)<br>ODC (6)<br>4 other (≤ 2)<br>(19 total)	129±10<br>136±8<br>120-129	37±3<br>36±1<br>30-39	CoC 35%<br>ODC 33%<br>11 other ≤ 7%<br>(38 total)	YPR011C (14)<br>CoC (4)<br>1 other (1)<br>(19 total)	157±9<br>146±7<br>143	42±2<br>38±4<br>39	YPR011C 71%<br>CoC 20%<br>3 other ≤ 4%<br>(27 total)	YPR011C (2)

**4 OLD**	CoC (9)<br>AGC (3)<br>MFRN (3)<br>3 other (≤ 2)<br>(19 total)	128±9<br>129±3<br>130±9<br>120-129	38±3<br>37±2<br>34±1<br>30-38	CoC 53%<br>MFRN 16%<br>5 other ≤ 11%<br>(34 total)	YPR011C (14)<br>CoC (4)<br>1 other (1)<br>(19 total)	157±9<br>147±8<br>143	42±2<br>39±5<br>39	YPR011C 74%<br>CoC 20%<br>2 other ≤ 4%<br>(26 total)	YPR011C (2)<br>CoC (1)
**5**	YPR011C (10)<br>CoC (6)<br>2 other (1)<br>(18 total)	93±8<br>86±7<br>88	34±3<br>35±2<br>33-35	YPR011C 55%<br>CoC 33%<br>3 other ≤ 5%<br>(22 total)	APCX (13)<br>CoC (3)<br>YPR011C (2)<br>(18 total)	104±7<br>101±7<br>98±5	34±2<br>36±1<br>36±3	APCX 66%<br>CoC 25%<br>YPR011C 9%<br>(26 total)	YPR011C (2)<br>APCX (2)
**5 OLD**	YPR011C (11)<br>CoC (6)<br>peANT (1)<br>(18 total)	92±8<br>86±7<br>88	34±3<br>35±2<br>35	YPR011C 61%<br>CoC 33%<br>2 other ≤ 6%<br>(23 total)	CoC (11)<br>YPR011C (6)<br>CoC (1)<br>(18 total)	95±7<br>100±4<br>94	36±2<br>35±3<br>37	CoC 66%<br>YPR011C 34%<br>(23 total)	YPR011C (2)<br>CoC (2)
**6**	APCX (18)<br>YPR011C (1)<br>(19 total)	141±10<br>130	38±4<br>39	APCX 93%<br>YPR011C 7%<br>(31 total)	APCX (8)<br>AACX (3)<br>YPR011C (3)<br>5 other (1)<br>(19 total)	148±5<br>152±4<br>141±6	41±2<br>36±1<br>34±3	APCX 46%<br>YPR011C 15%<br>AACX 14%<br>6 other ≤ 6%<br>(32 total)	APCX (2)
**6 OLD**	ORC (6)#<br>YPR011C (4)<br>MFT/FAD (4)<br>3 other (≤ 2)<br>(19 total)	123±5<br>118±7<br>117±6	35±4<br>33±4<br>40±4	ORC 29%<br>MFT/FAD 22%<br>YPR011C 18%<br>5 other ≤ 13%<br>(27 total)	YPR011C (10)<br>MFT/FAD (3)<br>CoC (3)<br>3 other (1)<br>(19 total)	139±8<br>138±6<br>134±3	36±3<br>36±3<br>37±1	YPR011C 52%<br>MFT/FAD 18%<br>5 other ≤ 12%<br>(40 total)	YPR011C (2)

**New isoform of subfamily APC: APC4 exons**	**most similar ES**				**most similar FLS**				**final prediction**
	**top ES hits (number)**	**AAS**	**ASI (%)**	**top 5% ES hits in % of summed AS (number)**	**top FLS hits (number)**	**AAS**	**ASI (%)**	**top 5% FLS hits in % of summed AS (number)**	**subfamily hit (confidence score)**
**1**	APC1 (4)<br>(4 total)	144±6	70±1	APC1 83%<br>APC2 17%<br>(9 total)	APC1 (4)<br>(4 total)	140±6	70±1	APC1 84%<br>APC2 16%<br>(10 total)	APC1 (4)
**2**	APC2 (4)	226±6	75±1	APC2 100%	APC2 (4)	212±6	75±1	APC2 100%	APC2 (5)

	(4 total)			(4 total)	(4 total)			(4 total)	
**3**	APC2 (4)<br>(4 total)	88±4	50±2	APC2 89%<br>APC1 11%<br>(6 total)	APC2 (3)<br>APC3 (1)<br>(4 total)	81±2<br>80	50±2<br>50	APC2 75%<br>APC3 25%<br>(6 total)	APC2 (4)
**4**	APC2 (4)<br>(4 total)	299±5	88±1	APC2 100%<br>(4 total)	APC2 (4)<br>(4 total)	285±5	88±1	APC2 100%<br>(4 total)	APC2 (5)
**5**	APC2 (3)<br>(3 total)	167±2	53±2	APC2 83%<br>APC1 17%<br>(7 total)	APC1 (2)<br>APC2 (1)<br>(3 total)	151±5<br>148	51±1<br>56	APC1 56%<br>APC2 43%<br>(8 total)	APC2 (1)<br>APC1 (1)
**6**	APC1 (3)<br>(3 total)	200±4	56±2	APC1 100%<br>(4 total)	APC1 (3)<br>(3 total)	190±4	56±2	APC1 100%<br>(4 total)	APC1 (3)

**pAPC subfamily exons**	**most similar ES**				**most similar FLS**				**final prediction**
	**top ES hits (number)**	**AAS**	**ASI (%)**	**top 5% ES hits in % of summed AS (number)**	**top FLS hits (number)**	**AAS**	**ASI (%)**	**top 5% FLS hits in % of summed AS (number)**	**subfamily hit (confidence score)**
**1**	-	-	-	-	YPR011C (4)<br>(4 total)	461±12	36±2	YPR011C 97%<br>APCX 3%<br>(14 total)	YPR011C (2)
**1 OLD**	-	-	-	-	YPR011C (4)<br>(4 total)	461±12	36±2	YPR011C 100%<br>(13 total)	YPR011C (2)
**2**	YPR011C (5)<br>CoC (2)<br>(7 total)	78±4<br>78±3	47±2<br>43±3	YPR011C 72%<br>CoC 28%<br>(8 total)	YPR011C (5)<br>2 other (1)<br>(7 total)	82±5<br>77-80	43±2<br>40-47	YPR011C 73%<br>3 other ≤ 13%<br>(11 total)	YPR011C (4)
**2 OLD**	YPR011C (5)<br>CoC (2)<br>(7 total)	78±4<br>78±3	47±2<br>43±3	YPR011C 72%<br>CoC 28%<br>(8 total)	YPR011C (5)<br>2 other (1)<br>(7 total)	82±5<br>77-80	43±2<br>30-47	YPR011C 77%<br>3 other ≤ 12%<br>(12 total)	YPR011C (4)
**3**	MFT/FAD (2)<br>APCX (2)<br>3 other (1)<br>(7 total)	149±1<br>128±6<br>122-158	41±0<br>37±1<br>37-39	MFT/FAD 36%<br>APCX 26%<br>3 other ≤ 16%<br>(9 total)	MFT/FAD (6)<br>YPR011C (1)<br>(7 total)	155±20<br>140	42±4<br>45	MFT/FAD 65%<br>YPR011C 22%<br>3 other ≤ 6%<br>(19 total)	MFT/FAD (2)
**3 OLD**	MFT/FAD (3)<br>4 other (1)	139±14<br>100-149	46±7<br>37-41	MFT/FAD 44%<br>4 other ≤ 16%	MFT/FAD (6)<br>YPR011C (1)	155±20<br>140	42±4<br>45	MFT/FAD 73%<br>YPR011C 27%	MFT/FAD (2)

	(7 total)			(9 total)	(7 total)			(15 total)	

**CoC SLC25A16 subfamily exons**	**most similar ES**				**most similar FLS**				**final prediction**
	**top ES hits (number)**	**AAS**	**ASI (%)**	**top 5% ES hits in % of summed AS (number)**	**top FLS hits (number)**	**AAS**	**ASI (%)**	**top 5% FLS hits in % of summed AS (number)**	**subfamily hit (confidence score)**
**1**	YPR011C (2)<br>7 other (1)<br>(9 total)	45±8<br>33-74	44±1<br>31-38	YPR011C 24%<br>ALC 19%<br>6 other ≤ 14%<br>(9 total)	YPR011C (2)<br>TPC (2)<br>3 other (1)<br>(7 total)	45±8<br>35±5<br>33-74	44±1<br>34±3<br>31-39	YPR011C 28%<br>ALC 23%<br>3 other ≤ 21%<br>(7 total)	-
**1 OLD**	YPR011C (2)<br>TPC (2)<br>4 other (1)<br>(8 total)	45±8<br>32±2<br>33-74	44±1<br>34±2<br>31-37	YPR011C 25%<br>ALC 20%<br>4 other ≤ 17%<br>(8 total)	YPR011C (2)<br>TPC (2)<br>4 other (≤1)<br>(8 total)	45±8<br>35±5<br>33-74	44±1<br>34±3<br>31-39	YPR011C 24%<br>ALC 20%<br>4 other ≤ 19%<br>(9 total)	-
**2**	APC (6)<br>AAC (2)<br>(8 total)	75±3*<br>70±3	42±4<br>40±2	APC 72%<br>AAC 18%<br>2 other ≤ 6%<br>(14 total)	YPR011C (4)<br>TAAC (3)<br>MCART (1)<br>(8 total)	89±4*<br>89±6<br>85	54±4<br>54±4<br>39	YPR011C 50%<br>TAAC 41%<br>MCART 8%<br>(16 total)	-
**2 OLD**	APC (6)<br>AAC (2)<br>(8 total)	75±3*<br>70±3	42±4<br>40±2	APC 82%<br>AAC 18%<br>(12 total)	YPR011C (5)<br>APC (3)<br>(8 total)	89±4*<br>78±2	54±4<br>44±2	YPR011C 74%<br>APC 26%<br>(18 total)	YPR011C (2)
**3**	YPR011C (4)<br>APCX (2)<br>2 other (1)<br>(8 total)	117±9<br>102±11	43±2<br>39±2	YPR011C 62%<br>APCX 24%<br>3 other ≤ 6%<br>(15 total)	YPR011C (3.5)<br>TAAC (3.5)<br>SFC (1)<br>(8 total)	124±6<br>116±2<br>124	45±2<br>43±0<br>50	YPR011C 66%<br>TAAC 28%<br>SFC 6%<br>(14 total)	YPR011C (2)
**3 OLD**	YPR011C (5)<br>A28/A37 (2)<br>APC (1)<br>(8 total)	114±10<br>96±5<br>103	43±2<br>35±1<br>39	YPR011C 75%<br>A28/A37 9%<br>3 other ≤ 6%<br>(15 total)	YPR011C (7)<br>SFC (1)<br>(8 total)	118±9<br>124	43±3<br>50	YPR011C 93%<br>SFC 7%<br>(11 total)	YPR011C (4)
**4**	APC (4)<br>2 other (1)<br>(6 total)	44±12<br>42-49	50±4<br>33-41	APC 66%<br>SLC25A39-A40 19%<br>2 other ≤ 8%<br>(7 total)	APC (5)<br>TPC (1)<br>(6 total)	45±11<br>43	49±4<br>38	APC 84%<br>TPC 16%<br>(6 total)	APC (4)

**4 OLD**	APC (4)<br>2 other (1)<br>(6 total)	44±12<br>42-49	50±4<br>33-41	APC 66%<br>SLC25A39-A40 19%<br>TPC 16%<br>(6 total)	APC (5)<br>TPC (1)<br>(6 total)	45±11<br>43	49±4<br>38	APC 84%<br>TPC 16%<br>(6 total)	APC (4)
**5**	YPR011C (4)<br>SLC25A43 (1)<br>(5 total)	105±3<br>100	49±1<br>48	YPR011C 71%<br>SLC25A43 29%<br>(6 total)	SLC24A43 (2)<br>YPR011C (2)<br>APCX (1)<br>(5 total)	103±3<br>100±5<br>105	48±0<br>46±4<br>48	SLC25A43 41%<br>APCX 31%<br>YPR011C 29%<br>(9 total)	YPR011C (2)
**5 OLD**	YPR011C (5)<br>(5 total)	103±6	48±3	YPR011C 100%<br>(5 total)	YPR011C (4)<br>UCP2-3 (1)<br>(5 total)	96±5<br>93	44±5<br>30	YPR011C 84%<br>2 other ≤ 10%<br>(5 total)	YPR011C (4)
**6**	APC (5)<br>(5 total)	87±3	61±3	APC 88%<br>YPR011C 12%<br>(7 total)	APC (4)<br>YPR011C (1)<br>(5 total)	89±2<br>87	61±3<br>61	APC 62%<br>YPR011C 38%<br>(11 total)	APC (4)
**6 OLD**	APC (5)<br>(5 total)	87±3	61±3	APC 88%<br>YPR011C 12%<br>(7 total)	APC (4)<br>YPR011C (1)<br>(5 total)	89±2<br>87	61±3<br>61	APC 62%<br>YPR011C 38%<br>(11 total)	APC (4)
**7**	-	-	-	-	MFL (1)<br>peCFNC (1)<br>(2 total)	78<br>67	30<br>30	-	-
**7 OLD**	-	-	-	-	peCFNC (1)<br>(2 total)	67	30	-	-
**8**	APCX (5)<br>YPR011C (1)<br>(6 total)	57±5<br>48	42±2<br>43	APCX 64%<br>YPR011C 21%<br>2 other ≤ 10%<br>(11 total)	APCX (2)<br>4 other (1)<br>(6 total)	64±3<br>48-61	39±0<br>39-48	YPR011C 29%<br>APCX 22%<br>4 other ≤ 18%<br>(13 total)	APCX (2)
**8 OLD**	YPR011C (4)<br>2 other (≤1.5)<br>(6 total)	53±4<br>45-49	44±3<br>30-43	YPR011C 64%<br>AAC 24%<br>2 other ≤ 7%<br>(11 total)	YPR011C (3)<br>3 other (1)<br>(6 total)	56±3<br>46-64	46±2<br>43-57	YPR011C 49%<br>3 other ≤ 19%<br>(7 total)	YPR011C (2)
**9**	APC (6)<br>3 other (1)<br>(9 total)	111±4<br>98-118	32±2<br>32-35	APC 58%<br>MME 17%<br>2 other ≤ 15%<br>(17 total)	APC (5)<br>4 other (1)<br>(9 total)	122±8<br>111-122	33±1<br>31-41	APC 49%<br>7 other ≤ 16%<br>(20 total)	APC (3)
**9 OLD**	APC (6)<br>3 other (1)	111±4<br>98-118	32±2<br>32-35	APC 58%<br>3 other ≤ 17%	APC (6)<br>3 other (1)	119±10<br>111-117	33±1<br>34-41	APC 55%<br>6 other ≤ 17%	APC (4)

	(9 total)			(17 total)	(9 total)			(148total)	

**CoC subfamily exons SLC25A42**	**most similar ES**				**most similar FLS**				**final prediction**
	**top ES hits (number)**	**AAS**	**ASI (%)**	**top 5% ES hits in % of summed AS (number)**	**top FLS hits (number)**	**AAS**	**ASI (%)**	**top 5% FLS hits in % of summed AS (number)**	**subfamily hit (confidence score)**
**1**	PiC (2)<br>5 other (1)<br>(7 total)	21±3<br>24-30	35±1<br>30-57	PiC 17%<br>AACX 17%<br>SFC 17%<br>4 other ≤ 16%<br>(8 total)	TAAC (1)<br>APC (1)<br>7 other (≤ 1)<br>(8 total)	35<br>32<br>18-30	32<br>31<br>30-57	TAAC 16%<br>APC 14%<br>7 other ≤ 13%<br>(9 total)	-
**1 OLD**	NDT (2)<br>PiC (2)<br>3 other (1)<br>(7 total)	26±2<br>21±3<br>18-30	31±1<br>35±1<br>31-43	PiC 25%<br>NDT 22%<br>4 other ≤ 18%<br>(8 total)	APC (1)<br>SFC (1)<br>4 other (1)<br>(6 total)	32<br>30<br>18-29	31<br>32<br>31-43	APC 20%<br>SFC 19%<br>4 other ≤ 18%<br>(6 total)	-
**2**	APC (4)<br>TAAC (2)<br>2 other (1)<br>(8 total)	103±3<br>102±3<br>104-105	49±3<br>49±1<br>43-53	APC 50%<br>TAAC 31%<br>2 other ≤ 12%<br>(12 total)	APC (4.5)<br>TAAC (2)<br>2 other (≤ 1)<br>(8 total)	98±6<br>98±3<br>92-100	51±2<br>50±0<br>47-50	APC 58%<br>TAAC 31%<br>2 other ≤ 7%<br>(13 total)	APC (2)
**2 OLD**	APC (7)<br>TPC (1)<br>(8 total)	100±5<br>105	47±4<br>43	APC 84%<br>TPC 16%<br>(11 total)	APC (5.5)<br>YPR011C (2)<br>AAC (0.5)<br>(8 total)	97±6<br>95±5<br>92	50±3<br>48±1<br>50	APC 72%<br>YPR011C 24%<br>2 other ≤ 10%<br>(11 total)	APC (4)
**3**	SLC25A46 (2)<br>DIC (2)#<br>(4 total)	14±0*<br>12±0	38±0<br>50±0	SLC25A46 54%<br>DIC 46% #<br>(8 total)	AAC (4)#<br>(4 total)	8±1*	63±0	AAC 100% #<br>(4 total)	SLC25A46 (1)
**3 OLD**	DIC (4)#<br>(4 total)	13±1*	50±0	DIC 100% #<br>(4 total)	AAC (4)#<br>(4 total)	8±1*	63±0	AAC 100% #<br>(4 total)	DIC (2)#
**4**	AAC (2)<br>APCX (2)<br>(4 total)	115±12<br>113±2	35±1<br>33±1	AAC 58%<br>APCX 42%<br>(5 total)	APCX (4)#<br>(4 total)	130±4	38±1	APCX 100% #<br>(4 total)	AAC (1)<br>APCX (2)#
**4 OLD**	AAC (4)<br>(4 total)	111±10	33±2	AAC 100%<br>(6 total)	AAC (4)<br>(4 total)	121±5	33±1	AAC 92%<br>APC 8%	AAC (4)

								(10 total)	
**5**	YPR011C (3)<br>APCX (3)<br>SLC25A43 (1)<br>(7 total)	105±2<br>102±2<br>159	45±2<br>46±4<br>39	YPR011C 49%<br>APCX 30%<br>SLC24A43 21%<br>(11 total)	YPR011C (3)<br>APCX (2)<br>2 other (1)<br>(7 total)	105±2<br>119±6<br>98-162	46±2<br>56±2<br>39-49	APCX 44%<br>YPR011C 24%<br>2 other ≤ 20%<br>(13 total)	-
**5 OLD**	YPR011C (7)<br>(7 total)	109±19	44±4	YPR011C 97%<br>APC 3%<br>(13 total)	YPR011C (7)<br>(7 total)	109±20	44±4	YPR011C 87%<br>3 other ≤ 5%<br>(18 total)	YPR011C (4)
**6**	BT (5)<br>APC (1)<br>(6 total)	158±11<br>129	47±4<br>37	BT 82%<br>APC 18%<br>(11 total)	BT (5)<br>APC (1)<br>(6 total)	158±11<br>144	47±4<br>39	BT 81%<br>APC 19%<br>(11 total)	BT (4)
**6 OLD**	APC (6)<br>(6 total)	142±13*	42±4	APC 85%<br>TPC 7%<br>(8 total)	YPR011C (4)<br>APC (2)<br>(6 total)	144±9<br>151±7	43±1<br>45±6	APC 57%<br>YPR011C 43%<br>(17 total)	APC (4)
**7**	APCX (1.5)<br>APC (0.5)<br>(2 total)	156±8<br>148	33±1<br>32	APCX 84%<br>APC 16%<br>(4 total)	YPR011C (4)<br>APCX (1)<br>(5 total)	191±10<br>204	32±1<br>30	YPR011C 73%<br>2 other ≤ 21%<br>(9 total)	APCX (1)<br>YPR011C (2)
**7 OLD**	APC (2)<br>(2 total)	149±1	31±1	-	YPR011C (5)<br>(5 total)	191±9	32±1	YPR011C 87%<br>2 other ≤ 7%<br>(10 total)	YPR011C (2)

**pCoC subfamily exons**	**most similar ES**				**most similar FLS**				**final prediction**
	**top ES hits (number)**	**AAS**	**ASI (%)**	**top 5% ES hits in % of summed AS (number)**	**top FLS hits (number)**	**AAS**	**ASI (%)**	**top 5% FLS hits in % of summed AS (number)**	**subfamily hit (confidence score)**
**1**	AAC (2)<br>2 other (1)<br>(4 total)	116±0<br>122-143	53±1<br>38-44	AAC 46%<br>2 other ≤ 29%<br>(8 total)	YPR011C (3)<br>TAAC (1)<br>(4 total)	127±2<br>139	48±5<br>44	YPR011C 73%<br>TAAC 27%<br>(5 total)	YPR011C (2)
**1 OLD**	AAC (4)<br>(4 total)	115±4	48±5	AAC 98%<br>APC 2%	YPR011C (4)<br>(4 total)	127±2	48±5	YPR011C 100%<br>(5 total)	AAC (2)<br>YPR011C (2)

				(16 total)					
**2**	MFT/FAD (2)<br>3 other (1)<br>(5 total)	65±4<br>58-70	40±2<br>38-45	MFT/FAD 30%<br>APCX 30%<br>2 other ≤ 21%<br>(7 total)	PiC (1)<br>APCX (1)<br>3 other (1)<br>(5 total)	77<br>70<br>58-66	41<br>45<br>34-41	APCX 30%<br>PiC 24%<br>6 other ≤ 22%<br>(11 total)	-
**2 OLD**	MFT/FAD (2)<br>3 other (1)<br>(5 total)	65±4<br>58-66	40±2<br>34-38	MFT/FAD 42%<br>3 other ≤ 21%<br>(7 total)	PiC (1)<br>MFT/FAD (1)<br>3 other (1)<br>(5 total)	77<br>66<br>58-66	41<br>41<br>34-38	MFT/FAD 24%<br>PiC 24%<br>APC 23%<br>5 other ≤ 8%<br>(12 total)	-
**3**	YPR011C (5)<br>(5 total)	168±15	44±4	YPR011C 100%<br>(5 total)	YPR011C (4)<br>APCX (1)<br>(5 total)	175±8<br>162	45±4<br>41	YPR011C 72%<br>APCX 19%<br>TAAC 9%<br>(8 total)	YPR011C (4)
**3 OLD**	YPR011C (5)<br>(5 total)	168±15	44±4	YPR011C 100%<br>(5 total)	YPR011C (4)<br>AAC (1)<br>(5 total)	175±8<br>153	45±4<br>39	YPR011C 94%<br>AAC 6%<br>(8 total)	YPR011C (4)
**4**	CIC (1)<br>ORC (1)<br>3 other (1)<br>(5 total)	100<br>91<br>80-85	42<br>46<br>32-38	ORC 21%<br>MME 20%<br>6 other ≤ 18%<br>(10 total)	CIC (1)<br>CAC (1)<br>3 other (≤ 1)<br>(5 total)	105<br>103<br>80-97	42<br>43<br>31-34	CIC 22%<br>APC 20%<br>6 other ≤ 20%<br>(9 total)	-
**4 OLD**	ALC<br>ORC (1)<br>3 other (1)<br>(5 total)	97<br>91<br>77-85	40<br>46<br>31-38	APC 24%<br>ALC 29%<br>3 other ≤ 21%<br>(8 total)	APC (2)<br>3 other (1)<br>(3 total)	100±3<br>80-98	36±2<br>31-37	APC 37%<br>5 other ≤ 19%<br>(11 total)	-
**5**	YPR011C (4)<br>(4 total)	166±7	41±3	YPR011C 100%<br>(8 total)	YPR011C (4)<br>(4 total)	191±9	46±3	YPR011C 100%<br>(8 total)	YPR011C (5)
**5 OLD**	YPR011C (4)<br>(4 total)	166±7	41±3	YPR011C 100%<br>(8 total)	YPR011C (4)<br>(4 total)	191±9	46±3	YPR011C 100%<br>(8 total)	YPR011C (5)
**6**	TPC (3)<br>YPR011C (1)<br>(4 total)	84±13<br>83	36±1<br>30	TPC 76%<br>YPR011C 24%<br>(5 total)	TPC (3)<br>MFT/FAD (1)<br>(4 total)	79±10<br>77	34±3<br>38	TPC 76%<br>MFT/FAD 24%<br>(5 total)	TPC (4)
**6 OLD**	TPC (3)<br>YPR011C (1)<br>(4 total)	84±13<br>83	36±1<br>30	TPC 76%<br>YPR011C 24%<br>(5 total)	TPC (3)<br>MFT/FAD (1)<br>(4 total)	79±10<br>77	34±3<br>38	TPC 76%<br>MFT/FAD 24%<br>(5 total)	TPC (4)

**7**	YPR011C (5)<br>(5 total)	66±5	45±6	YPR011C 100%<br>(7 total)	YPR011C (3)<br>2 other (1)<br>(5 total)	64±6<br>60-61	44±5<br>32-41	YPR011C 61%<br>2 other ≤ 20%<br>(6 total)	YPR011C (4)
**7 OLD**	YPR011C (5)<br>(5 total)	66±5	45±6	YPR011C 100%<br>(7 total)	YPR011C (3)<br>2 other (1)<br>(5 total)	64±6<br>60-61	44±5<br>32-41	YPR011C 61%<br>2 other ≤ 20%<br>(6 total)	YPR011C (4)

**YPR011C subfamily exons**	**most similar ES**				**most similar FLS**				**final prediction**
	**top ES hits (number)**	**AAS**	**ASI (%)**	**top 5% ES hits in % of summed AS (number)**	**top FLS hits (number)**	**AAS**	**ASI (%)**	**top 5% FLS hits in % of summed AS (number)**	**subfamily hit (confidence score)**
**1**	-	-	-	-	-	-	-	-	-
**1 OLD**	-	-	-	-	-	-	-	-	-
**2**	CoC (3)<br>(3 total)	63±0	81±0	CoC 100%<br>(6 total)	CoC (3)<br>(3 total)	59±0	81±0	CoC 100%<br>(12 total)	CoC (3)
**2 OLD**	CoC (3)<br>(3 total)	63±0	81±0	CoC 100%<br>(6 total)	CoC (3)<br>(3 total)	59±0	81±0	CoC 100%<br>(12 total)	CoC (3)
**3**	CoC (2)<br>YMC/BOU (1)<br>(3 total)	139±0<br>129	36±0<br>38	CoC 69%<br>other 2 ≤16%<br>(6 total)	CoC (3)<br>(3 total)	155±8	43±2	CoC 100%<br>(3 total)	CoC (1)
**3 OLD**	CoC (2)<br>YMC/BOU (1)<br>(3 total)	139±0<br>129	36±0<br>38	CoC 69%<br>other 2 ≤16%<br>(6 total)	CoC (3)<br>(3 total)	155±8	43±2	CoC 100%<br>(3 total)	CoC (1)
**4**	MFT/FAD (1)<br>GC (1)<br>SLC25A45/A47/A48 (1)<br>(3 total)	23<br>23<br>19	46<br>39<br>31	MFT/FAD 36%<br>SLC25A45/A47/A48 15%<br>MTCH 14%<br>other 3 ≤12%<br>(6 total)	MFT/FAD<br>AGC<br>SLC25A39-A40<br>(3 total)	34<br>22<br>18	46<br>39<br>31	MFT/FAD 46%<br>SLC25A39-A40 24%<br>other 2 ≤15%<br>(4 total)	-
**4 OLD**	MFT/FAD (1)<br>AGC (1)<br>peCFNC (1)<br>(3 total)	23<br>22<br>17	46<br>39<br>31	-	MFT/FAD<br>AGC<br>SLC25A39-A40<br>(3 total)	34<br>22<br>18	46<br>39<br>31	MFT/FAD 46%<br>SLC25A39-A40 24%<br>other 2 ≤15%<br>(4 total)	-

**5**	CoC (2)<br>APCX (1)<br>(3 total)	100±2<br>92	49±3<br>46	CoC 89%<br>APCX 11%<br>(5 total)	TAAC (3)<br>(3 total)	104±7	55±3	TAAC 78%<br>CoC 22%<br>(6 total)	CoC (1)<br>TAAC (1)
**5 OLD**	CoC (3)<br>(3 total)	97±4	49±2	CoC 100%<br>(4 total)	CoC (3)<br>(3 total)	98±3	50±1	CoC 100%<br>(7 total)	CoC (2)
**6**	MFT/FAD (2)<br>PiC (1)<br>(3 total)	120±9<br>114	53±3<br>42	MFT/FAD 68%<br>PiC 32%<br>(4 total)	MFT/FAD (2)<br>PiC (1)<br>(3 total)	120±9<br>114	53±3<br>53	MFT/FAD 68%<br>PiC 32%<br>(3 total)	MFT/FAD (1)
**6 OLD**	MFT/FAD (2)<br>PiC (1)<br>(3 total)	120±9<br>114	53±3<br>42	MFT/FAD 68%<br>PiC 32%<br>(4 total)	MFT/FAD (2)<br>PiC (1)<br>(3 total)	120±9<br>114	53±3<br>53	MFT/FAD 68%<br>PiC 32%<br>(3 total)	MFT/FAD (1)
**7**	CoC (3)<br>(3 total)	218±4	33±2	CoC 100%<br>(3 total)	TPC (2)<br>APC (1)<br>(3 total)	231±3<br>224	33±2<br>35	TPC 45%<br>APC 33%<br>2 other ≤11%<br>(7 total)	(CoC/TPC
**7 OLD**	CoC (3)<br>(3 total)	218±4	33±2	CoC 100%<br>(3 total)	TPC (2)<br>APC (1)<br>(3 total)	231±3<br>224	33±2<br>35	TPC 55%<br>APC 33%<br>CoC 11%<br>(6 total)	TPC (1)
**8**	CoC (2)<br>AACX (1)<br>(3 total)	69±1<br>65	48±0<br>48	CoC 57%<br>TAAC 27%<br>AACX 16%<br>(6 total)	TAAC (3)<br>(3 total)	64±2	48±3	CoC 100%<br>(3 total)	CoC (1)
**8 OLD**	CoC (3)<br>(3 total)	67±2	47±2	CoC 100%<br>(4 total)	CoC (2)<br>APC (1)<br>(3 total)	61±2<br>58	43±6<br>33	CoC 68%<br>APC 32%<br>(6 total)	CoC (2)

**TPC subfamily exons**	**most similar ES**				**most similar FLS**				**final prediction**
	**top ES hits (number)**	**AAS**	**ASI (%)**	**top 5% ES hits in % of summed AS (number)**	**top FLS hits (number)**	**AAS**	**ASI (%)**	**top 5% FLS hits in % of summed AS (number)**	**subfamily hit (confidence score)**
**1**	MFT/FAD (6)<br>4 other (1)<br>(10 total)	90±10*<br>74-89	38±3<br>31-34	MFT/FAD 69%<br>6 other ≤ 11%<br>(15 total)	UCP2-3 (6)<br>4 other (1)<br>(10 total)	99±8*<br>93-117	34±3<br>30-39	UCP2-3 60%<br>4 other ≤ 12%<br>(11 total)	UCP2-3 (2)

**1 OLD**	MFT/FAD (7)<br>3 other (1)<br>(10 total)	87±11*<br>77-89	37±3<br>31-34	MFT/FAD 69%<br>5 other ≤ 12%<br>(14 total)	UCP2-3 (6)<br>MFT/FAD (2)<br>3 other (1)<br>(10 total)	99±8*<br>92±2<br>93-117	34±3<br>35±1<br>30-39	UCP2-3 60%<br>MFT/FAD 18%<br>2 other ≤ 12%<br>(11 total)	UCP2-3 (2)
**2**	SFC (2)<br>UCP2-3 (2)<br>5 other (1)<br>(9 total)	97±2<br>92±11<br>84-102	34±1<br>38±5<br>30-35	UCP2-3 22%<br>SFC 18%<br>7 other ≤ 12%<br>(11 total)	TAAC (4)<br>MFT/FAD (2)<br>4 other (1)<br>(10 total)	103±8<br>109±9<br>85-131	35±2<br>37±3<br>33-35	TAAC 39%<br>MFT/FAD 21%<br>5 other ≤ 12%<br>(12 total)	-
**2 OLD**	UCP2-3 (4)<br>SFC (2)<br>2 other (1)<br>(8 total)	87±10<br>97±2<br>86-102	37±3<br>34±1<br>30-33	UCP2-3 42%<br>SFC 23%<br>4 other ≤ 12%<br>(9 total)	MFT/FAD (4)<br>YPR011C (2)<br>4 other (1)<br>(10 total)	101±11<br>108±6<br>81-109	35±4<br>32±1<br>33-35	MFT/FAD 40%<br>YPR011C 21%<br>4 other ≤ 11%<br>(10 total)	-
**3**	APCX (4)<br>4 other (1)<br>(8 total)	107±7<br>91-107	41±2<br>31-39	APCX 57%<br>4 other ≤ 13%<br>(9 total)	APCX (3)<br>4 other (1)<br>(7 total)	110±9<br>103-112	36±1<br>30-33	APCX 57%<br>ALC 14%<br>4 other ≤ 7%<br>(12 total)	APCX (2)
**3 OLD**	ALC (3)<br>4 other (1)<br>(7 total)	102±10<br>79-107	30±1<br>30-39	ALC 39%<br>PiC 16%<br>4 other ≤ 14%<br>(10 total)	CoC (2)<br>5 other (1)<br>(7 total)	98±14<br>90-109	30±0<br>30-32	ALC 27%<br>CoC 24%<br>4 other ≤ 23%<br>(10 total)	-
**4**	BT (3)<br>PiC (2)<br>2 other (1)<br>(7 total)	144±2<br>118±9<br>95-141	38±2<br>34±3<br>31-49	BT 48%<br>PiC 26%<br>2 other ≤ 16%<br>(7 total)	BT (2)<br>CoC (2)<br>2 other (1)<br>(6 total)	150±4<br>143±1<br>108-150	39±2<br>42±4<br>30	BT 33%<br>CoC 23%<br>4 other ≤ 18%<br>(11 total)	-
**4 OLD**	PiC (3)<br>CoC (2)<br>APC (1)<br>(6 total)	123±11<br>134±8<br>134	34±2<br>42±7<br>35	PiC 40%<br>CoC 39%<br>APC 21%<br>(12 total)	CoC (3)<br>3 other (1)<br>(6 total)	143±1<br>103-150	41±3<br>30-39	CoC 32%<br>YPR011C 26%<br>2 other ≤ 24%<br>(11 total)	-
**5**	CoC (4)<br>(4 total)	99±6	37±3	CoC 100%<br>(4 total)	CoC (4)<br>(4 total)	106±4	35±2	CoC 78%<br>3 other ≤ 12%<br>(9 total)	CoC (4)
**5 OLD**	CoC (4)<br>(4 total)	99±6	37±3	CoC 100%<br>(4 total)	CoC (4)<br>(4 total)	106±4	35±2	CoC 90%<br>2 other ≤ 5%<br>(8 total)	CoC (4)

**6**	ALC (2)<br>4 other (1)<br>(6 total)	124±3<br>107-126	35±2<br>30-34	ALC 27%<br>AGC 18%<br>7 other ≤ 16%<br>(10 total)	MFT/FAD (3.5)<br>3 other (≤ 1)<br>(6 total)	126±13<br>116-136	34±2<br>30-33	MFT/FAD 60%<br>PNC 12%<br>5 other ≤ 9%<br>(16 total)	MFT/FAD (2)
**6 OLD**	ALC (2)<br>4 other (1)<br>(6 total)	124±3<br>105-126	35±2<br>30-35	ALC 27%<br>AGC 18%<br>5 other ≤ 16%<br>(8 total)	MFT/FAD (4)<br>ALC (2)<br>(6 total)	126±13<br>125±2	34±2<br>34±3	MFT/FAD 75%<br>ALC 25%<br>(9 total)	MFT/FAD (2)

**pTPC subfamily exons**	**most similar ES**				**most similar FLS**				**final prediction**
	**top ES hits (number)**	**AAS**	**ASI (%)**	**top 5% ES hits in % of summed AS (number)**	**top FLS hits (number)**	**AAS**	**ASI (%)**	**top 5% FLS hits in % of summed AS (number)**	**subfamily hit (confidence score)**
**1**	CoC (1)<br>APC (1)<br>MFT/FAD (1)<br>APCX (1)<br>(4 total)	105<br>94<br>94<br>93	43<br>46<br>33<br>30	CoC 27%<br>3 other 24%<br>(4 total)	NDT (3)<br>YPR011C (1)<br>(4 total)	97±9<br>97	43±4<br>41	NDT 51%<br>YPR011C 38%<br>APC 11%<br>(6 total)	NDT (2)
**1 OLD**	APC (1)<br>2 other (1)<br>(4 total)	89±6<br>94-105	43±3<br>33-43	APC 47%<br>2 other ≤ 28%<br>(5 total)	NDT (3)<br>YPR011C (1)<br>(4 total)	97±9<br>97	43±4<br>41	NDT 51%<br>YPR011C 38%<br>APC 11%<br>(6 total)	NDT (2)
**2**	AGC (2)<br>NDT (1)<br>(3 total)	70±7<br>53	35±1<br>32	AGC 73%<br>2 other ≤ 14%<br>(5 total)	APC (1)<br>A12 (1)<br>YPR011C (1)<br>(3 total)	86<br>76<br>59	36<br>33<br>32	APC 39%<br>A23-A13 34%<br>2 other ≤ 13%<br>(4 total)	AGC (1)
**2 OLD**	AGC (2)<br>NDT (1)<br>(3 total)	70±7<br>53	35±1<br>32	AGC 73%<br>2 other ≤ 14%<br>(5 total)	APC (1)<br>A12 (1)<br>YPR011C (1)<br>(3 total)	86<br>76<br>59	36<br>33<br>32	APC 39%<br>A23-A13 34%<br>2 other ≤ 13%<br>(4 total)	AGC (1)
**3**	PiC (1)<br>AAC (1)<br>SLC25A39-A40 (1)<br>(3 total)	86<br>83<br>78	32<br>32<br>34	PiC 35%<br>AAC 34%<br>2 other ≤ 16%<br>(4 total)	YPR011C (1)<br>GlyC (1)<br>TAAC (1)<br>(3 total)	84<br>81<br>80	42<br>37<br>34	GlyC 33%<br>TAAC 22%<br>3 other ≤ 17%<br>(6 total)	-

**3 OLD**	SLC25A39-A40 (1)<br>PiC (1)<br>(3 total)	77±1<br>86	33±1<br>32	SLC25A39-A40 48%<br>PiC 36%<br>(4 total)	YPR011C (1)<br>GlyC (1)<br>PiC (1)<br>(3 total)	84<br>81<br>77	42<br>37<br>37	GlyC 34%<br>APC 33%<br>2 other ≤ 18%<br>(5 total)	-
**4**	APCX (2.5)<br>CoC (1)<br>SLC25A43 (0.5)<br>(4 total)	84±2<br>84±3<br>84	38±1<br>38±0<br>36	APCX 47%<br>CoC 38%<br>2 other ≤ 10%<br>(12 total)	APC (2)<br>APCX (1)<br>SLC25A39-A40 (1)<br>(4 total)	86±2<br>87<br>86	39±1<br>43<br>33	APC 34%<br>CoC 25%<br>3 other ≤ 16%<br>(8 total)	APCX (1)
**4 OLD**	CoC (4)<br>(4 total)	83±2	39±1	CoC 88%<br>SFC 12%<br>(5 total)	APC (2)<br>CoC (1)<br>SLC25A39-A40 (1)<br>(4 total)	86±2<br>86<br>86	39±1<br>43<br>33	APC 50%<br>CoC 37%<br>SLC25A39-A40<br>13%<br>(5 total)	CoC (2)
**5**	CoC (4)<br>(4 total)	134±6	44±3	CoC 92%<br>APCX 8%<br>(7 total)	YPR011C (2)<br>CoC (2)<br>(4 total)	132±4<br>125±1	41±2<br>38±7	CoC 68%<br>2 other ≤ 16%<br>(13 total)	CoC (3)
**5 OLD**	CoC (4)<br>(4 total)	134±6	44±3	CoC 100%	YPR011C (2)<br>CoC (2)<br>(4 total)	132±4<br>125±1	41±2<br>38±7	CoC 77%<br>YPR011C 23%<br>(11 total)	CoC (3)
**6**	MFT/FAD (2)<br>ALC (2)<br>(4 total)	94±2<br>90±3	33±1<br>35±1	MFT/FAD 51%<br>ALC 49%<br>(5 total)	ALC (2)<br>2 other (1)<br>(4 total)	90±3<br>90-104	32±1<br>32-34	ALC 44%<br>GC 28%<br>3 other ≤ 12%<br>(7 total)	MFT/FAD (1)
**6 OLD**	MFT/FAD (2)<br>ALC (2)<br>(4 total)	94±2<br>90±3	33±1<br>35±1	MFT/FAD 51%<br>ALC 49%<br>(5 total)	ALC (2)<br>2 other (1)<br>(4 total)	90±3<br>84-90	32±1<br>30-32	ALC 50%<br>GlyC 24%<br>2 other ≤ 13%<br>(7 total)	MFT/FAD (1)
**7**	APC (1)<br>YPR011C (1)<br>BT (1)<br>(3 total)	149<br>131<br>125	37<br>32<br>36	-	MFT/FAD (2)<br>AGC (1)<br>(3 total)	155±2<br>159	33±0<br>36	MFT/FAD 44%<br>AGC 34%<br>APC 22%<br>(8 total)	-
**7 OLD**	APC (1)<br>YPR011C (1)<br>MFT/FAD (1)<br>(3 total)	149<br>131<br>118	37<br>32<br>31	APC 45%<br>YPR011C 40%<br>2 other ≤ 7%<br>(6 total)	MFT/FAD (2)<br>AGC (1)<br>(3 total)	155±2<br>159	33±0<br>36	MFT/FAD 44%<br>AGC 34%<br>APC 22%<br>(8 total)	-

BT subfamily exons	most similar ES				most similar FLS				final prediction
	top ES hits (number)	AAS	ASI (%)	top 5% ES hits in % of summed AS (number)	top FLS hits (number)	AAS	ASI (%)	top 5% FLS hits in % of summed AS (number)	subfamily hit (confidence score)
**1**	YPR011C (1)<br>(1 total)	186	30	-	-	-	-	-	-
**1 OLD**	YPR011C (1)<br>(1 total)	186	30	-	-	-	-	-	-
**2**	CoC (1)<br>AGC (1)<br>MFT/FAD (1)<br>(3 total)	117<br>115<br>106	37<br>41<br>50	-	TAAC (2)<br>CoC (1)<br>(3 total)	114±3<br>117	40±3<br>37	TAAC 44%<br>CoC 34%<br>AGC 22%<br>(5 total)	-
**2 OLD**	CoC (1)<br>A12 (1)<br>MFT/FAD (1)<br>(3 total)	117<br>115<br>106	37<br>41<br>50	-	AGC (2)<br>CoC (1)<br>(3 total)	107±10<br>117	38±3<br>37	AGC 64%<br>CoC 36%<br>(2 total)	AGC (1)
**3**	CoC (1)<br>(1 total)	265	30	-	BTL (2)<br>(2 total)	358±4	37±1	BTL 84%<br>CoC 16%<br>(5 total)	BTL (1)
**3 OLD**	CoC (1)<br>(1 total)	265	30	-	CoC (2)<br>(2 total)	343±4	35±3	CoC 64%<br>APC 24%<br>YPR011C 12%<br>(5 total)	CoC (1)

BTL subfamily exons	most similar ES				most similar FLS				final prediction
	top ES hits (number)	AAS	ASI (%)	top 5% ES hits in % of summed AS (number)	top FLS hits (number)	AAS	ASI (%)	top 5% FLS hits in % of summed AS (number)	subfamily hit (confidence score)
**1**	-	-	-	-	-	-	-	-	-
**1 OLD**	-	-	-	-	-	-	-	-	-

**2**	BT (2)<br>(2 total)	77±5	49±2	-	BT (2)<br>(2 total)	77±5	49±2	-	(BT)
**2 OLD**	CoC (1)<br>APC (1)<br>(2 total)	63<br>60	34<br>34	APC 46%<br>CoC 31%<br>A12 23%<br>(5 total)	SAMC (2)<br>(2 total)	74±2	38±0	-	(SAMC)
**3**	APC (1)<br>(1 total)	125	30	-	BT (2)<br>(2 total)	174±6	31±0	BT 100%<br>(4 total)	BT (1)
**3 OLD**	APC (1)<br>(1 total)	125	30	-	-	-	-	-	-
**4**	BT (2)<br>(2 total)	145±11	33±3	-	BT (2)<br>(2 total)	167±9	37±3	BT 100%<br>(4 total)	BT (1)
**4 OLD**	TPC (2)<br>(2 total)	113±5	43±2	-	CoC (2)<br>(2 total)	147±5	33±2	-	(CoC)
**5**	BT (2)<br>(2 total)	193±8	39±0	BT 100%<br>(4 total)	BT (2)<br>(2 total)	195±7	41±1	BT 100%<br>(6 total)	BT (2)
**5 OLD**	APC (1)<br>(1 total)	125	30	-	APC (1)<br>YPR011C (1)<br>(2 total)	161<br>158	35<br>32	-	-

c. MC-CA cluster subfamilies

**OAC subfamily exons**	**most similar ES**				**most similar FLS**				**final prediction**
	**top ES hits (number)**	**AAS**	**ASI (%)**	**top 5% ES hits in % of summed AS (number)**	**top FLS hits (number)**	**AAS**	**ASI (%)**	**top 5% FLS hits in % of summed AS (number)**	**subfamily hit (confidence score)**
**1**	-	-	-	-	OGC (5)<br>(5 total)	234±15	33±1	OGC 100%<br>(8 total)	OGC (2)
**1 OLD**	-	-	-	-	OGC (5)<br>(5 total)	234±15	33±1	OGC 100%<br>(8 total)	OGC (2)
**2**	APC (2)#<br>YPR011C (2)	43±0<br>34±2	44±3<br>41±0	APC 46%<br>YPR011C 36%	APC (2)#<br>SLC25A44 (2)	43±0<br>37±5	44±3<br>48±2	APC 44%<br>SLC25A44 37%	-

	GlyC (2)<br>(5 total)	35	50	GlyC 19%<br>(5 total)	GlyC (1)<br>(5 total)	37	50	GlyC 19%<br>(5 total)	
**2 OLD**	APC (2)#<br>YPR011C (2)<br>GlyC (2)<br>(5 total)	43±0<br>34±2<br>35	44±3<br>41±0<br>50	APC 46%<br>YPR011C 36%<br>GlyC 19%<br>(5 total)	APC (2)#<br>SLC25A44 (2)<br>GlyC (1)<br>(5 total)	43±0<br>37±5<br>37	44±3<br>48±2<br>50	APC 44%<br>SLC25A44 37%<br>GlyC 19%<br>(5 total)	-
**3**	OGC (4)<br>UCP4 (1)<br>(5 total)	101±7<br>98	39±3<br>35	OGC 90%<br>UCP4 10%<br>(9 total)	OGC (4)<br>ER_ANT (1)<br>(5 total)	107±4<br>122	41±3<br>35	OGC 77%<br>ER_ANT 23%<br>(9 total)	OGC (4)
**3 OLD**	OGC (4)<br>UCP4 (1)<br>(5 total)	101±7<br>98	39±3<br>35	OGC 90%<br>UCP4 10%<br>(9 total)	OGC (5)<br>(5 total)	107±4	40±3	OGC 90%<br>DIC 10%<br>(11 total)	OGC (4)
**4**	UCP4 (4)<br>GC (1)<br>(5 total)	81±5<br>84	36±7<br>36	UCP4 79%<br>GC 21%<br>(5 total)	UCP4 (3)<br>SAMCX (1)<br>SAMC (1)<br>(5 total)	87±6<br>101<br>90	35±1<br>36<br>31	UCP4 58%<br>SAMCX 22%<br>SAMC 20%<br>(9 total)	UCP4 (4)
**4 OLD**	UCP4 (5)<br>(5 total)	81±5	35±4	UCP4 100%<br>(5 total)	UCP4 (4)<br>SAMC (1)<br>(5 total)	89±6<br>90	35±2<br>31	UCP4 80%<br>SAMC 20%<br>(9 total)	UCP4 (4)
**5**	UCP4 (3)<br>DIC (1)<br>(4 total)	137±10<br>139	37±2<br>35	UCP4 54%<br>DIC 25%<br>OGC 21%<br>(8 total)	DIC (3)<br>CoC (1)<br>(4 total)	152±10<br>130	35±4<br>34	DIC 51%<br>OGC 22%<br>4 other ≤13%<br>(10 total)	UCP4 (2)<br>DIC (2)
**5 OLD**	UCP4 (3)<br>DIC (1)<br>(4 total)	137±10<br>139	37±2<br>35	UCP4 54%<br>DIC 25%<br>OGC 21%<br>(8 total)	DIC (3)<br>CoC (1)<br>(4 total)	152±10<br>130	35±4<br>34	DIC 65%<br>OGC 24%<br>2 other ≤6%<br>(8 total)	UCP4 (2)<br>DIC (2)

**AT4G03115 subfamily exons**	**most similar ES**				**most similar FLS**				**final prediction**
	**top ES hits (number)**	**AAS**	**ASI (%)**	**top 5% ES hits in % of summed AS (number)**	**top FLS hits (number)**	**AAS**	**ASI (%)**	**top 5% FLS hits in % of summed AS (number)**	**subfamily hit (confidence score)**

**1**	MFRN (1)<br>NDT (1)<br>TPC (1)<br>(3 total)	64<br>60<br>59	40<br>33<br>31	-	MFRN (2)<br>TAAC (1)<br>(3 total)	82±10	36±1	-	-
**1 OLD**	MFRN (1)<br>NDT (1)<br>TPC (1)<br>(3 total)	64<br>60<br>59	40<br>33<br>31	-	MFRN (2)<br>(2 total)	82±10	36±1	-	-
**2**	SFC (2)<br>APC (1)<br>(3 total)	51±2<br>41	51±6<br>30	SFC 72%<br>APC 14%<br>3 other ≤5%<br>(8 total)	SLC25A39-A40 (1)<br>SFC (1)<br>OAC (1)<br>(3 total)	59<br>52<br>40	36<br>45<br>50	-<br>(3 total)	SFC (1)
**2 OLD**	SFC (2)<br>APC (1)<br>(3 total)	51±2<br>41	51±6<br>30	SFC 72%<br>APC 21%<br>ALC 7%<br>(6 total)	SLC25A39-A40 (1)<br>SFC (1)<br>ALC (1)<br>(3 total)	59<br>52<br>40	36<br>45<br>40	-	SFC (1)
**3**	SLC25A39-A40 (1)<br>AACX (1)<br>(2 total)	127<br>121	35<br>32	-	UCP2-3 (2)<br>(2 total)	175±2	37±2	-	(UCP2-3)
**3 OLD**	SLC25A39-A40 (1)<br>(1 total)	127	35	-	UCP2-3 (2)<br>(2 total)	175±2	37±2	-	(UCP2-3)
**4**	OGC (2)<br>(2 total)	129±6	41±4	-	OGC (2)<br>(2 total)	135±1	39±2	-	(OGC)
**4 OLD**	OGC (2)<br>(2 total)	129±6	41±4	-	OGC (2)<br>(2 total)	135±1	39±2	-	(OGC)
**5**	OGC (1)<br>A46 (1)<br>(2 total)	38<br>35	41<br>41	-	OGC (1)<br>OAC (1)<br>(2 total)	38<br>31	35<br>35	-	-
**5 OLD**	OGC (1)<br>CoC (1)<br>(2 total)	38<br>22	41<br>35	-	OGC (1)<br>CoC (1)<br>(2 total)	38<br>22	35<br>35	-	-
**6**	UCP5-6 (1)<br>UCP4 (1)<br>(2 total)	104<br>94	30<br>36	-	NDT (1)<br>GC (1)<br>(2 total)	102<br>99	36<br>39	-	-
**6 OLD**	UCP4 (1)	94	36	-	NDT (1)	102	36	NDT 53%	NDT (1)

	AGC (1)<br>(2 total)	82	30		SLC25A44 (1)<br>(2 total)	92	32	3 other ≤ 16%<br>(4 total)	
**7**	CAC (1)<br>UCP5-6 (1)<br>(2 total)	49<br>37	69<br>62	-	CAC (1)<br>A46 (1)<br>(2 total)	46<br>35	69<br>62	A46 44%<br>OGC 24%<br>4 other ≤ 8%<br>(8 total)	-
**7 OLD**	CoC (1)<br>A13 (1)<br>(2 total)	48<br>35	61<br>54	-	TPC (1)<br>OGC (0.5)<br>DIC (0.5)<br>(2 total)	33<br>45<br>45	69<br>77<br>77	DIC 39%<br>TPC 21%<br>2 other ≤ 20%<br>(5 total)	-
**8**	DIC (2)<br>(2 total)	80±0	38±2	DIC 100%<br>(5 total)	DIC (1)<br>UCP4 (1)<br>(2 total)	77<br>74	39<br>36	-	DIC (1)
**8 OLD**	DIC (2)<br>(2 total)	80±0	38±2	DIC 100%<br>(5 total)	DIC (1)<br>UCP4 (1)<br>(2 total)	77<br>74	39<br>36	-	DIC (1)

**UCP2-3 subfamily exons**	**most similar ES**				**most similar FLS**				**final prediction**
	**top ES hits (number)**	**AAS**	**ASI (%)**	**top 5% ES hits in % of summed AS (number)**	**top FLS hits (number)**	**AAS**	**ASI (%)**	**top 5% FLS hits in % of summed AS (number)**	**subfamily hit (confidence score)**
**1**	TPC (3)<br>UCP4 (3)<br>APC (2)<br>5 other (1)<br>(13 total)	76±4*<br>75±4<br>70±8<br>76-86	34±2<br>36±2<br>33±3<br>31-37	TPC 21%<br>APC 16%<br>UCP4 15%<br>9 other ≤ 9%<br>(20 total)	TPC (7)<br>SAMC (2)<br>4 other (1)<br>(13 total)	98±7*<br>83±6<br>82-102	39±3<br>36±3<br>33-40	TPC 57%<br>6 other ≤ 8%<br>(21 total)	TPC (2)
**1 OLD**	TPC (4)<br>UCP4 (3)<br>DIC (2)<br>APC (2)<br>2 other (1)<br>(13 total)	75±4*<br>75±4<br>79±7<br>70±8<br>70-78	34±3<br>36±2<br>34±3<br>33±3<br>36-37	TPC 33%<br>UCP4 19%<br>APC 17%<br>5 other ≤ 13%<br>(19 total)	TPC (7)<br>UCP4 (3)<br>SAMC (2)<br>SLC25A44 (1)<br>(13 total)	98±7*<br>90±9<br>83±6<br>88	39±3<br>40±0<br>36±3<br>40	TPC 57%<br>3 other ≤ 10%<br>(18 total)	TPC (2)
**2**	GlyC (3)<br>DIC (1)	113±8*<br>133	35±3<br>31	GlyC 76%<br>2 other ≤ 13%	UCP5-6 (6.5)<br>2 other (≤ 1)	128±8*<br>121-122	32±1<br>31-33	UCP5-6 85%<br>other 2 ≤ 12%	UCP5-6 (2)

	UCP4 (1)<br>(5 total)	109	33	(6 total)	(8 total)			(15 total)	
**2 OLD**	GlyC (3)<br>DIC (1)<br>UCP4 (1)<br>(5 total)	113±8<br>133<br>109	35±3<br>31<br>33	GlyC 76%<br>2 other ≤ 13%<br>(6 total)	DIC (4)<br>GlyC (2)<br>OGC (1)<br>(7 total)	114±10<br>127±5<br>116	31±1<br>32±1<br>30	DIC 61%<br>GlyC 23%<br>2 other ≤ 11%<br>(13 total)	GlyC (2)<br>DIC (2)
**3**	DIC (3)<br>SLC25A43 (2)<br>UCP5-6 (2)<br>YMC/BOU (2)<br>3 other (1)<br>(12 total)	150±13*<br>149±7<br>145±5<br>141±4<br>132-151	38±4<br>40±3<br>35±4<br>41±1<br>32-41	DIC 24%<br>YMC/BOU 19%<br>UCP5-6 17%<br>5 other ≤ 13%<br>(19 total)	UCP4 (6)<br>TPC (2)<br>4 other (1)<br>(12 total)	168±14*<br>164±3<br>161-177	41±5<br>43±0<br>37-41	UCP4 48%<br>ALC 13%<br>7 other ≤ 9%<br>(22 total)	-
**3 OLD**	DIC (5)<br>YMC/BOU (2)<br>UCP4 (2)<br>3 other (1)<br>(12 total)	144±13*<br>141±4<br>138±7<br>103-151	37±4<br>41±1<br>39±3<br>31-37	DIC 26%<br>YMC/BOU 22%<br>UCP4 19%<br>5 other ≤ 9%<br>(23 total)	UCP4 (6)<br>TPC (2)<br>ALC (2)<br>2 other (≤ 1)<br>(12 total)	168±14*<br>170±6<br>164±3<br>151-174	41±5<br>42±4<br>43±0<br>37-41	UCP4 60%<br>ALC 13%<br>5 other ≤ 9%<br>(18 total)	UCP4 (1)
**4**	UCP4 (8)<br>(8 total)	74±11	39±4	UCP4 100%<br>(11 total)	UCP4 (8)<br>(8 total)	72±5	41±3	UCP4 100%<br>(15 total)	UCP4 (5)
**4 OLD**	UCP4 (8)<br>(8 total)	74±11	39±4	UCP4 100%<br>(11 total)	UCP4 (8)<br>(8 total)	72±5	41±3	UCP4 100%<br>(15 total)	UCP4 (5)
**5**	DIC (6)<br>CIC (2)<br>(8 total)	158±24*<br>136±6	44±4<br>38±1	DIC 89%<br>CIC 11%<br>(11 total)	DIC (7)<br>OGC (1)<br>(8 total)	176±15*<br>198	42±4<br>52	DIC 81%<br>OGC 19%<br>(16 total)	DIC (4)
**5 OLD**	DIC (8)<br>(8 total)	152±23*	43±4	DIC 100%<br>(10 total)	DIC (7)<br>OGC (1)<br>(8 total)	176±15*<br>198	42±4<br>52	DIC 81%<br>OGC 19%<br>(16 total)	DIC (4)
**6**	UCP5-6 (10)<br>UCP4 (1)<br>(11 total)	89±8<br>81	39±2<br>42	UCP5-6 92%<br>UCP4 8%<br>(15 total)	UCP5-6 (10)<br>UCP4 (1)<br>(11 total)	92±9<br>83	44±3<br>39	UCP5-6 92%<br>UCP4 8%<br>(20 total)	UCP5-6 (4)
**6 OLD**	UCP4 (10)<br>DIC (1)<br>(11 total)	76±5<br>65	39±3<br>35	UCP4 88%<br>DIC 12%<br>(12 total)	UCP4 (9)<br>DIC (2)<br>(11 total)	76±5<br>70±5	38±3<br>33±2	UCP4 79%<br>DIC 21%<br>(15 total)	UCP4 (4)

**new mammalian isoform of UCP2-3: UCP1 exons**	**most similar ES**				**most similar FLS**				**final prediction**
	**top ES hits (number)**	**AAS**	**ASI (%)**	**top 5% ES hits in % of summed AS (number)**	**top FLS hits (number)**	**AAS**	**ASI (%)**	**top 5% FLS hits in % of summed AS (number)**	**subfamily hit (confidence score)**
**1**	UCP3 (4)<br>(4 total)	170±14	67±6	UCP3 67%<br>UCP2 33%<br>(29 total)	UCP3 (4)<br>(4 total)	166±14	70±6	UCP3 66%<br>UCP2 34%<br>(28 total)	UCP3 (4)
**2**	UCP3 (3)<br>UCP2 (1)<br>(4 total)	251±5<br>244	61±357	UCP3 76%<br>UCP2 24%<br>(8 total)	UCP3 (3)<br>UCP2 (1)<br>(4 total)	233±10228	61±4<br>57	UCP3 76%<br>UCP2 24%<br>(8 total)	UCP3 (4)
**3**	UCP3 (4)<br>(4 total)	230±10	59±3	UCP3 100%<br>(4 total)	UCP3 (4)<br>(4 total)	224±10	58±2	UCP3 100%<br>(4 total)	UCP3 (5)
**4**	UCP2 (2.8)<br>UCP3 (1.3)<br>(4 total)	129±5<br>137±10	58±2<br>61±5	UCP2 73%<br>UCP3 27%<br>(14 total)	UCP2 (2.8)<br>UCP3 (1.3)<br>(4 total)	116±5<br>124±9	58±3<br>61±5	UCP2 73%<br>UCP3 27%<br>(14 total)	UCP2 (4)
**5**	UCP3 (4)<br>(4 total)	274±4	70±2	UCP3 52%<br>UCP2 48%<br>(20 total)	UCP3 (4)<br>(4 total)	261±4	70±2	UCP3 58%<br>UCP2 42%<br>(18 total)	UCP3 (4)
**6**	UCP3 (4)<br>(4 total)	147±4	67±2	UCP3 100%<br>(8 total)	UCP3 (4)<br>(4 total)	137±4	64±2	UCP3 100%<br>(9 total)	UCP3 (5)

**pUCP2-3 subfamily exons**	**most similar ES**				**most similar FLS**				**final prediction**
	**top ES hits (number)**	**AAS**	**ASI (%)**	**top 5% ES hits in % of summed AS (number)**	**top FLS hits (number)**	**AAS**	**ASI (%)**	**top 5% FLS hits in % of summed AS (number)**	**subfamily hit (confidence score)**
**1**	UCP4 (2)<br>TPC (1)	46±3<br>46	42±2<br>30	UCP4 43%<br>TPC 21%	PiC (1)<br>UCP4 (1)	50<br>49	30<br>43	OAC 34%<br>UCP4 27%	-

	OAC (1)<br>(4 total)	37	31	OAC 17%<br>(4 total)	MFT/FAD (1)<br>OAC (1)<br>(4 total)	45<br>37	33<br>31	MFT/FAD 25%<br>PiC 14%<br>(6 total)	
**1 OLD**	UCP4 (2)<br>TPC (1)<br>NDT (1)<br>(4 total)	46±3<br>46<br>33	42±2<br>30<br>39	UCP4 54%<br>TPC 27%<br>NDT 19%<br>(4 total)	PiC (1)<br>UCP4 (1)<br>MFT/FAD (1)<br>YMC/BOU (1)<br>(4 total)	50<br>49<br>45<br>35	30<br>43<br>33<br>35	UCP4 32%<br>MFT/FAD 29%<br>2 other ≤ 23%<br>(5 total)	UCP4 (1)
**2**	UCP4 (3)<br>SLC25A45/A47/A48 (1)<br>(4 total)	153±5*<br>146	40±1<br>36	UCP4 60%<br>SLC25A45/A47 24%<br>OAC 16%<br>(8 total)	UCP4 (3)<br>SLC25A45/A47/A48 (1)<br>(4 total)	18<br>6±8*<br>164	42±4<br>36	UCP4 80%<br>AT4G03115 12%<br>2 other ≤ 4%<br>(16 total)	UCP4 (4)
**2 OLD**	UCP4 (4)<br>(4 total)	150±7*	39±3	UCP4 100%<br>(6 total)	UCP4 (3)<br>TPC (1)<br>(4 total)	18<br>6±8*<br>163	42±4<br>36	UCP4 96%<br>TPC 4%<br>(14 total)	UCP4 (4)
**3**	AGC (1)<br>DIC (1)<br>CIC (1)<br>(3 total)	38<br>34<br>34	30<br>34<br>30	AGC 36%<br>DIC 32%<br>2 other ≤16%<br>(4 total)	AGC (1)<br>GC (1)<br>DIC (1)<br>CIC (1)<br>(4 total)	56<br>39<br>39<br>36	33<br>33<br>30<br>33	GC 34%<br>AGC 33%<br>CIC 21%<br>DIC 12%<br>(6 total)	-
**3 OLD**	AGC (1)<br>DIC (1)<br>SAMC (1)<br>(3 total)	38<br>34<br>29	30<br>34<br>30	AGC 38%<br>DIC 34%<br>SAMC 29%<br>(3 total)	DIC (2)<br>AGC (1)<br>(3 total)	38±1<br>56	30±0<br>33	DIC 58%<br>AGC 42%<br>(4 total)	DIC (1)
**4**	UCP4 (4)<br>(4 total)	99±5	38±3	UCP4 100%<br>(7 total)	UCP4 (3)<br>DIC (1)<br>(4 total)	107±6<br>106	45±3<br>43	UCP4 75%<br>DIC 25%<br>(4 total)	UCP4 (4)
**4 OLD**	UCP4 (4)<br>(4 total)	99±5	38±3	UCP4 100%<br>(7 total)	UCP4 (3)<br>DIC (1)<br>(4 total)	107±6<br>106	45±3<br>43	UCP4 75%<br>DIC 25%<br>(4 total)	UCP4 (4)
**5**	AT4G03115 (3)<br>UCP4 (1)<br>(4 total)	107±1<br>104	49±0<br>58	AT4G03115 81%<br>UCP4 19%<br>(8 total)	AT4G03115 (2)<br>OGC (2)<br>(4 total)	112±0<br>109±0	54±2<br>58±0	OGC 50%<br>AT4G03115 38%<br>UCP4 12%<br>(6 total)	AT4G03115<br>(2)
**5 OLD**	UCP4 (4)<br>(4 total)	103±1	57±2	UCP4 89%<br>OGC 12%	OGC (4)<br>(4 total)	108±2	58±2	OGC 63%<br>UCP4 37%	UCP4 (2)<br>OGC (2)

				(5 total)				(7 total)	
**6**	DIC (4)<br>(4 total)	76±3	47±6	DIC 100%<br>(5 total)	DIC (3)<br>UCP4 (1)<br>(4 total)	79±2<br>77	46±6<br>49	DIC 88%<br>UCP4 12%<br>(11 total)	DIC (4)
**6 OLD**	DIC (4)<br>(4 total)	76±3	47±6	DIC 100%<br>(5 total)	DIC (3)<br>UCP4 (1)<br>(4 total)	79±2<br>77	46±6<br>49	DIC 88%<br>UCP4 12%<br>(11 total)	DIC (4)
**7**	CIC (4)<br>(4 total)	69±6	50±5	CIC 89%<br>ALC 11%<br>(5 total)	CIC (2)<br>UCP5-6 (1)<br>AAC (1)<br>(4 total)	67±4<br>86<br>69	48±4<br>44<br>46	CIC 58%<br>UCP5-6 30%<br>AAC 12%<br>(5 total)	CIC (3)
**7 OLD**	UCP4 (2)<br>YMC/BOU (1)<br>ALC (19<br>(4 total)	68±3<br>64<br>60	42±3<br>44<br>40	ALC 36%<br>UCP4 33%<br>3 other ≤ 13%<br>(10 total)	AAC (2)<br>UCP4 (1)<br>ALC (1)<br>(4 total)	67±2<br>70<br>60	42±5<br>44<br>40	AAC 35%<br>ALC 31%<br>UCP4 26%<br>YMC/BOU 8%<br>(7 total)	-
**8**	UCP5-6 (4)<br>(4 total)	106±3	61±1	UCP5-6 100%<br>(10 total)	UCP5-6 (4)<br>(4 total)	117±3	67±3	UCP5-6 100%<br>(10 total)	UCP5-6 (5)
**8 OLD**	DIC (2)<br>UCP4 (2)<br>(4 total)	92±1<br>84±8	57±2<br>45±4	UCP4 48%<br>DIC 39%<br>OGC 13%<br>(5 total)	DIC (4)<br>(4 total)	94±7	57±4	DIC 100%<br>(6 total)	DIC (2)
**9**	-	-	-	-	-	-	-	-	-
**9 OLD**	-	-	-	-	-	-	-	-	-

**UCP5-6 subfamily exons**	**most similar ES**				**most similar FLS**				**final prediction**
	**top ES hits (number)**	**AAS**	**ASI (%)**	**top 5% ES hits in % of summed AS (number)**	**top FLS hits (number)**	**AAS**	**ASI (%)**	**top 5% FLS hits in % of summed AS (number)**	**subfamily hit (confidence score)**
**1**	OGC (7)<br>5 other (1)	45±8	38±4	OGC 64%<br>6 other ≤ 10%	OGC (5)<br>DIC (3)	50±5<br>55±1	39±3<br>44±4	OGC 45%<br>DIC 27%	OGC (2)

	(12 total)			(13 total)	4 other (1)<br>(12 total)			5 other ≤ 8%<br>(14 total)	
**1OLD**	OGC (7.5)<br>5 other ≤ 1)<br>(12 total)	44±8	37±5	OGC 71%<br>5 other ≤ 15%<br>(13 total)	OGC (6)<br>DIC (4)<br>2 other (1)<br>(12 total)	49±6<br>53±3	38±5<br>43±4	OGC 49%<br>DIC 37%<br>3 other ≤ 4%<br>(15 total)	OGC (2)
**2**	UCP4 (11)<br>3 other (1)<br>(14 total)	135±5	46±3	UCP4 86%<br>5 other ≤ 5%<br>(34 total)	UCP4 (12)<br>UCP2-3 (2)<br>(14 total)	142±5<br>141±4	48±2<br>45±2	UCP4 78%<br>UCP2-3 18%<br>2 other ≤ 2%<br>(29 total)	UCP4 (4)
**2OLD**	UCP4 (11)<br>3 other (1)<br>(14 total)	135±5	46±3	UCP4 90%<br>4 other ≤ 5%<br>(33 total)	UCP4 (12)<br>UCP3 (2)<br>(14 total)	142±5<br>141±4	48±2<br>45±2	UCP4 77%<br>UCP2-3 18%<br>2 other ≤ 2%<br>(28 total)	UCP4 (4)
**3**	DIC (6)<br>OGC (1)<br>(7 total)	71±4<br>72	37±3<br>38	DIC 84%<br>OGC 16%<br>(13 total)	OGC (4)<br>3 other (1)<br>(7 total)	68±4	43±3	OGC 69%<br>UCP4 15%<br>3 other ≤ 8%<br>(13 total)	DIC (2)<br>OGC (2)
**3OLD**	DIC (6)<br>OGC (1)<br>(7 total)	71±4<br>72	37±3<br>38	DIC 84%<br>OGC 16%<br>(13 total)	OGC (4)<br>3 other (1)<br>(7 total)	68±4	43±3	OGC 69%<br>UCP4 15%<br>3 other 8%<br>(13 total)	DIC (2)<br>OGC (2)
**4**	GlyC (6)<br>AT4G03115 (1)<br>(7 total)	61±4<br>59	46±2<br>39	GlyC 97%<br>AT4G03115 3%<br>(12 total)	GlyC (3)<br>AT5G42130 (2)<br>other 2 (1)<br>(7 total)	64±3<br>66±2	47±2<br>46	GlyC 35%<br>AT5G42130 29%<br>3 other ≤ 15%<br>(10 total)	GlyC (2)
**4OLD**	GlyC (7)<br>(7 total)	60±4	45±2	GlyC 100%<br>(10 total)	GlyC (6)<br>CoC (1)<br>(7 total)	63±4<br>64	45±3<br>39	GlyC 93%<br>2 other ≤ 4%<br>(14 total)	GlyC (4)
**5**	UCP4 (3)<br>UCP2-3 (3)<br>OGC (1)<br>(7 total)	84±3<br>80±5<br>71	42±1<br>35±4<br>41	UCP4 51%<br>UCP2-3 36%<br>OGC 13%<br>(11 total)	UCP4 (3)<br>4 other (1)<br>(7 total)	84±3	40±4	UCP4 35%<br>OAC 25%<br>3 other ≤ 20%<br>(11 total)	UCP4 (1)
**5OLD**	UCP4 (3)<br>UCP2-3 (3)<br>OGC (1)	84±3<br>80±5<br>71	42±1<br>35±4<br>41	UCP4 51%<br>UCP2-3 36%<br>OGC 13%	UCP4 (4)<br>3 other (1)<br>(7 total)	81±6	38±4	UCP4 50%<br>APC 22%<br>2 other ≤ 14%	UCP4 (2)

	(7 total)			(11 total)				(9 total)	
**6**	UCP4 (6)<br>DIC (4)<br>OGC (2)<br>(12 total)	80±7<br>66±5<br>76±9	43±3<br>36±3<br>37±4	UCP4 62%<br>DIC 22%<br>OGC 16%<br>(21 total)	DIC (4)<br>OGC (3)<br>UCP2-3 (3)<br>UCP4 (2)<br>(12 total)	86±10<br>80±10<br>75±11<br>88±2	38±2<br>38±4<br>38±3<br>46±3	DIC 42%<br>UCP4 20%<br>OGC 20%<br>UCP2-3 18%<br>(21 total)	UCP4 (1)
**6OLD**	UCP4 (6)<br>DIC (4)<br>OGC (2)<br>(12 total)	80±7<br>66±5<br>76±9	43±3<br>36±3<br>37±4	UCP4 62%<br>DIC 22%<br>OGC 16%<br>(21 total)	DIC (4)<br>OGC (3)<br>UCP2-3 (3)<br>UCP4 (2)<br>(12 total)	86±10<br>80±10<br>75±11<br>88±2	38±2<br>38±4<br>38±3<br>46±3	DIC 42%<br>UCP4 20%<br>OGC 20%<br>UCP2-3 18%<br>(21 total)	UCP4 (1)
**7**	DIC (3)<br>UCP4 (2)<br>2 other (1)<br>(7 total)	105±10<br>105±6	41±3<br>40±1	DIC 36%<br>UCP4 25%<br>4 other ≤17%<br>(12 total)	DIC (6)<br>OAC (1)<br>(7 total)	118±1<br>109	44±3<br>32	DIC 58%<br>UCP4 25%<br>3 other ≤ 6%<br>(14 total)	DIC (2)
**7OLD**	DIC (5)<br>UCP4 (2)<br>(7 total)	102±9<br>105±6	40±3<br>40±1	DIC 65%<br>UCP4 30%<br>A12 5%<br>(12 total)	DIC (6)<br>A12 (1)<br>(7 total)	118±1<br>104	44±3<br>34	DIC 59%<br>UCP4 20%<br>2 other ≤ 14%	DIC (4)
**8**	UCP4 (7)<br>(7 total)	110±6	60±2	UCP4 100%<br>(18 total)	UCP2-3 (4)<br>UCP4 (3)<br>(7 total)	109±2<br>117±3	66±2<br>62±2	UCP2-3 55%<br>UCP4 45%<br>(9 total)	UCP4 (2)<br>UCP2-3 (2)
**8OLD**	UCP4 (7)<br>(7 total)	110±6	60±2	UCP4 100%<br>(18 total)	UCP2-3 (4)<br>UCP4 (3)<br>(7 total)	109±2<br>117±3	66±2<br>62±2	UCP2-3 55%<br>UCP4 45%<br>(9 total)	UCP4 (2)<br>UCP2-3 (2)
**9**	NDT (3)<br>4 other (1)<br>(7 total)	39±2	54±0	NDT 44%<br>4 other ≤15%<br>(7 total)	DIC (2)<br>5 other (1)<br>(7 total)	32±2	58±4	DIC 23%<br>7 other ≤17%<br>(12 total)	-
**9OLD**	NDT (3)<br>4 other (1)<br>(7 total)	39±2	54±0	NDT 46%<br>5 other ≤ 15%<br>(9 total)	DIC (2.5)<br>4 other (1)<br>(7 total)	31±2	54±6	DIC 34%<br>6 other ≤ 16%<br>(12 total)	-

**UCP4 subfamily exons**	**most similar ES**	**most similar FLS**	**final prediction**

	top ES hits (number)	AAS	ASI (%)	top 5% ES hits in % of summed AS (number)	top FLS hits (number)	AAS	ASI (%)	top 5% FLS hits in % of summed AS (number)	subfamily hit (confidence score)
**1**	ALC (1)<br>UCP2-3 (1)<br>UCP5-6 (1)<br>(3 total)	50<br>43<br>35	30<br>40<br>32	-	ALC (1)<br>DIC (1)<br>AAC (1)<br>(3 total)	50<br>45<br>45	30<br>35<br>32	-	-
**1 OLD**	ALC (1)<br>UCP2-3 (1)<br>(2 total)	50<br>43	30<br>40	-	ALC (1)<br>DIC (1)<br>AAC (1)<br>(3 total)	50<br>45<br>45	30<br>35<br>32	-	-
**2**	UCP2-3 (2)<br>UCP5-6 (1)<br>(3 total)	153±5<br>140	41±1<br>47	UCP2-3 52%<br>UCP5-6 48%<br>(8 total)	UCP2-3 (2)<br>UCP5-6 (1)<br>(3 total)	155±2<br>153	36±1<br>41	UCP5-6 66%<br>UCP2-3 28%<br>OGC 6%<br>(14 total)	UCP5-6 (1)<br>UCP2-3 (1)
**2 OLD**	UCP2-3 (3)<br>(3 total)	148±8	39±2	UCP2-3 100%<br>(5 total)	UCP2-3 (3)<br>(3 total)	152±4	35±1	UCP2-3 84%<br>OGC 16%<br>(10 total)	UCP2-3 (2)
**3**	CoC (2)<br>YPR011C (1)<br>OAC (1)<br>(4 total)	47±6<br>55<br>38	34±2<br>36<br>36	CoC 50%<br>OAC 35%<br>YPR011C 15%<br>(5 total)	CoC (3)<br>DIC (1)<br>(4 total)	50±4<br>57	33±2<br>39	CoC 72%<br>DIC 28%<br>(4 total)	CoC (2)
**3 OLD**	CoC (2)<br>YPR011C (1)<br>(3 total)	47±6<br>55	34±2<br>36	CoC 63%<br>YPR011C 37%<br>(3 total)	Coc (3)<br>DIC (2)<br>(4 total)	50±4<br>57±1	33±2<br>36±4	CoC 57%<br>DIC 32%<br>YPR011C 10%<br>(6 total)	CoC (2)
**4**	UCP2-3 (5)<br>GC (1)<br>(6 total)	76±8*<br>81	34±0<br>39	UCP2-3 75%<br>GC 18%<br>OAC 7%<br>(11 total)	UCP2-3 (6)<br>(6 total)	94±4*	43±2	UCP2-3 100%<br>(7 total)	UCP2-3 (4)
**4 OLD**	UCP2-3 (5)<br>ALC (1)<br>(6 total)	76±8*<br>79	34±0<br>32	UCP2-3 83%<br>ALC 17%<br>(10 total)	UCP2-3 (6)<br>(6 total)	94±4*	43±2	UCP2-3 100%<br>(7 total)	UCP2-3 (4)
**5**	OGC (4)<br>PiC (1)<br>(5 total)	92±5<br>84	39±2<br>40	OGC 81%<br>PiC 19%<br>(5 total)	UCP2-3 (3)<br>A14/A35 (2)<br>(5 total)	100±5<br>107±1	48±1<br>44±2	UCP5-6 52%<br>UCP2-3 48%<br>(9 total)	OGC (2)<br>UCP2-3 (1)<br>UCP5-6 (1)

**5 OLD**	OGC (4)<br>PiC (1)<br>(5 total)	92±5<br>84	39±2<br>40	OGC 81%<br>PiC 19%<br>(5 total)	UCP2-3 (5)<br>(5 total)	99±4	46±3	UCP2-3 100%<br>(8 total)	OGC (2)<br>UCP2-3 (2)
**6**	DIC (3)<br>UCP2-3 (2)<br>(5 total)	74±5<br>69±2	54±3<br>48±2	DIC 62%<br>UCP2-3 38%<br>(6 total)	DIC (3)<br>UCP2-3 (2)<br>(5 total)	70±1<br>69±2	54±4<br>48±2	DIC 61%<br>UCP2-3 39%<br>(5 total)	DIC (4)
**6 OLD**	DIC (3)<br>UCP2-3 (2)<br>(5 total)	74±5<br>69±2	54±3<br>48±2	DIC 62%<br>UCP2-3 38%<br>(6 total)	DIC (3)<br>UCP2-3 (2)<br>(5 total)	70±1<br>69±2	54±4<br>48±2	DIC 61%<br>UCP2-3 39%<br>(5 total)	DIC (4)
**7**	OAC (4)<br>UCP2-3 (1)<br>(5 total)	78±1<br>76	46±4<br>37	OAC 80%<br>UCP2-3 20%<br>(9 total)	OAC (4)<br>UCP2-3 (1)<br>(5 total)	79±1<br>75	46±1<br>37	OAC 82%<br>2 other ≤10%<br>(6 total)	OAC (4)
**7 OLD**	UCP2-3 (4)<br>DIC (1)<br>(5 total)	71±3<br>65	45±7<br>42	UCP2-3 85%<br>DIC 11%<br>SAMC 4%<br>(10 total)	DIC (2)<br>3 other (1)<br>(5 total)	70±3<br>67-75	52±4<br>37-48	DIC 62%<br>3 other ≤ 10%<br>(8 total)	UCP2-3 (2)<br>DIC (1)
**8**	UCP5-6 (4)<br>(4 total)	118±6	58±5	UCP5-6 100%<br>(4 total)	UCP5-6 (4)<br>(4 total)	132±2	58±2	UCP5-6 100%<br>(10 total)	UCP5-6 (5)
**8 OLD**	29 (2)<br>OGC (1)<br>AGC (1)<br>(4 total)	83±1<br>102<br>92	43±2<br>53<br>47	ALC 44%<br>OGC 36%<br>AGC 20%<br>(7 total)	DIC (2)<br>AGC (1)<br>OGC (1)<br>(4 total)	100±2<br>121<br>104	49±2<br>50<br>53	DIC 47%<br>AGC 28%<br>OGC 24%<br>(4 total)	-
**9**	UCP2-3 (3)<br>SLC25A39-A40 (1)<br>UCP5-6 (1)<br>(5 total)	54±4<br>65<br>47	36±2<br>39<br>30	UCP2-3 59%<br>SLC25A39-A40 24%<br>UCP5-6 17%<br>(5 total)	UCP2-3 (3)<br>SLC25A39-A40 (1)<br>UCP5-6 (1)<br>(5 total)	54±4<br>62<br>47	36±2<br>39<br>30	UCP2-3 49%<br>UCP5-6 28%<br>SLC25A39-A40 23%<br>(5 total)	UCP2-3 (3)
**9 OLD**	UCP2-3 (3)<br>SLC25A39-A40 (1.5)<br>PiC (0.5)<br>(5 total)	54±4<br>54±11<br>43	36±2<br>35±5<br>30	UCP2-3 60%<br>SLC25A39-A40 32%<br>2 other ≤ 4%<br>(8 total)	UCP2-3 (3)<br>SLC25A39-A40 (1)<br>SLC25A44 (1)<br>(5 total)	54±4<br>62<br>44	36±2<br>39<br>35	UCP2-3 56%<br>SLC25A39-A40 36%<br>SLC25A44 8%<br>(5 total)	UCP2-3 (4)

**pUCP4 subfamily exons**	**most similar ES**				**most similar FLS**				**final prediction**
	**top ES hits**	**AAS**	**ASI**	**top 5% ES hits in**	**top FLS hits**	**AAS**	**ASI**	**top 5% FLS hits**	**subfamily hit**

	(number)		(%)	% of summed AS (number)	(number)		(%)	in % of summed AS (number)	(confidence score)
**1**	APCX (1)<br>(1 total)	153	35	-	UCP5-6 (1)<br>(1 total)	219	30	-	-
**1 OLD**	UCP2-3 (1)<br>(1 total)	156	41	-	UCP2-3 (1)<br>(1 total)	218	34	-	-
**2**	DIC (2)<br>OGC (1)<br>(3 total)	394±34*<br>345	37±3<br>32	DIC 85%<br>OGC 15%<br>(4 total)	UCP5-6 (3)<br>(3 total)	485±34*	42±3	UCP5-6 95%<br>UCP2-3 5%<br>(8 total)	UCP5-6 (1)
**2 OLD**	DIC (2)<br>OGC (1)<br>(3 total)	394±34*<br>345	37±3<br>32	DIC 85%<br>OGC 15%<br>(4 total)	UCP2-3 (2)<br>DIC (1)<br>(3 total)	438±1*	36±0	UCP2-3 60%<br>DIC 40%<br>(17 total)	UCP2-3 (1)

DIC subfamily exons	most similar ES				most similar FLS				final prediction
	**top ES hits (number)**	**AAS**	**ASI (%)**	**top 5% ES hits in % of summed AS (number)**	**top FLS hits (number)**	**AAS**	**ASI (%)**	**top 5% FLS hits in % of summed AS (number)**	**subfamily hit (confidence score)**
**1**	OAC (4)<br>OGC (2)<br>4 other (1)<br>(10 total)	74±5<br>74±10<br>60-86	39±1<br>32±2<br>32-41	OAC 41%<br>AAC 19%<br>OGC 15%<br>4 other ≤ 9%<br>(20 total)	OGC (7)<br>AAC (2)<br>3 other (1)<br>(11 total)	78±3<br>84±10<br>73-85	39±4<br>37±4<br>33-42	OGC 60%<br>AAC 15%<br>7 other ≤ 5%<br>(21 total)	OGC (2)
**1 OLD**	OGC (3)<br>CoC (2)<br>5 other (1)<br>(10 total)	70±9<br>65±1<br>60-86	31±1<br>37±0<br>32-41	AAC 25%<br>CoC 20%<br>OGC 18%<br>5 other ≤ 11%<br>(26 total)	OGC (8)<br>AAC (2)<br>SLC25A39-A40 (1)<br>(11 total)	79±4<br>84±10<br>70	39±4<br>37±4<br>33	OGC 74%<br>AAC 16%<br>3 other ≤ 4%<br>(19 total)	OGC (2)
**2**	UCP2-3 (2)<br>OGC (2)<br>3 other (1)<br>(7 total)	79±5<br>76±2<br>73-83	41±3<br>37±2<br>33-35	OGC 28%<br>UCP2-3 23%<br>GC 22%<br>2 other ≤ 14%<br>(9 total)	OGC (2)<br>UCP2-3 (2)<br>5 other (≤ 1)<br>(7 total)	83±6<br>80±2<br>74-80	35±0<br>42±4<br>30-33	OGC 37%<br>UCP2-3 24%<br>8 other ≤ 7%<br>(16 total)	-
**2 OLD**	UCP2-3 (2)	79±5	41±3	UCP2-3 30%	OGC (2)	83±6	35±0	OGC 37%	-

	OGC (2)<br>ALC (2)<br>AGC (1)<br>(7 total)	76±2<br>72±3<br>73	37±2<br>36±3<br>35	OGC 29%<br>ALC 21%<br>AGC 14%<br>(9 total)	UCP2-3 (2)<br>5 other (≤ 1)<br>(7 total)	80±2<br>74-80	42±4<br>30-33	UCP2-3 27%<br>4 other ≤ 13%<br>(11 total)	
**3**	OGC (4)<br>(4 total)	58±11	34±1	OGC 100%<br>(4 total)	OGC (4)<br>UCP4 (1)<br>(5 total)	64±12<br>71	35±2<br>36	OGC 78%<br>UCP4 22%<br>(7 total)	OGC (4)
**3 OLD**	OGC (4)<br>(4 total)	58±11	34±1	OGC 100%<br>(4 total)	OGC (4)<br>UCP4 (1)<br>(5 total)	64±12<br>71	35±2<br>36	OGC 78%<br>UCP4 22%<br>(7 total)	OGC (4)
**4**	SLC25A45/A47/A48 (2)<br>UCP2-3 (1)<br>OGC (1)<br>(4 total)	39±0<br>42<br>40	50±0<br>38<br>50	OGC 70%<br>4 other ≤ 11%<br>(21 total)	UCP4 (3)<br>OGC (1)<br>(4 total)	42±3<br>40	48±6<br>50	UCP4 45%<br>OGC 33%<br>SLC25A45/A47/A48 19%<br>ORC 3%<br>(14 total)	OGC (1)<br>UCP4 (1)
**4 OLD**	OGC (3)<br>UCP2-3 (1)<br>(4 total)	39±1<br>42	46±3<br>38	OGC 81%<br>3 other ≤ 11%<br>(21 total)	UCP4 (3)<br>OGC (1)<br>(4 total)	42±3<br>40	48±6<br>50	UCP4 53%<br>OGC 43%<br>ORC 4%<br>(11 total)	OGC (2)<br>UCP4 (2)
**5**	OGC (5)<br>(5 total)	37±7	54±7	OGC 100%<br>(5 total)	OGC (5)<br>(5 total)	33±7	61±7	OGC 100%<br>(6 total)	OGC (5)
**5 OLD**	OGC (5)<br>(5 total)	37±7	54±7	OGC 100%<br>(5 total)	OGC (5)<br>(5 total)	33±7	61±7	OGC 100%<br>(6 total)	OGC (5)
**6**	OGC (5)<br>(5 total)	45±7	51±8	OGC 100%<br>(5 total)	OGC (3)<br>MFRN (1)<br>PNC (1)<br>(5 total)	42±2<br>34<br>34	58±3<br>53<br>47	OGC 54%<br>MFRN 17%<br>PNC 17%<br>UCP5-6 11%<br>(6 total)	OGC (4)
**6 OLD**	OGC (5)<br>(5 total)	45±7	51±8	OGC 100%<br>(5 total)	OGC (3.3)<br>MFRN (1.7)<br>(5 total)	40±5<br>33±1	55±5<br>50±3	OGC 70%<br>MFRN 26%<br>SLC25A39-A40 4%<br>(9 total)	OGC (4)
**7**	UCP4 (4)<br>other 2 (0.5)<br>(5 total)	44±1<br>39	44±0<br>39	UCP4 86%<br>2 other ≤ 11%<br>(9 total)	YPR011C (2)<br>other 4 (≤ 1)<br>(5 total)	36±3<br>36-39	42±3<br>39	YPR011C 28%<br>UCP5-6 20%<br>UCP4 20%<br>4 other ≤ 12%	UCP4 (2)

								(12 total)	
**7 OLD**	UCP4 (4)<br>other 2 (0.5)<br>(5 total)	44±1<br>39	44±0<br>39	UCP4 86%<br>2 other ≤ 11%<br>(9 total)	YPR011C (2)<br>other 4 (≤ 1)<br>(5 total)	36±3<br>36-39	42±3<br>39-44	UCP4 38%<br>YPR011C 28%<br>4 other ≤ 12%<br>(13 total)	UCP4 (2)
**8**	OGC (5)<br>UCP4 (1)<br>(6 total)	88±10<br>89	54±5<br>52	OGC 91%<br>UCP4 9%<br>(8 total)	OGC (5)<br>UCP2-3 (1)<br>(6 total)	92±7<br>88	57±6<br>52	OGC 84%<br>UCP2-3 16%<br>(11 total)	OGC (4)
**8 OLD**	OGC (5)<br>UCP4 (1)<br>(6 total)	88±10<br>89	54±5<br>52	OGC 91%<br>UCP4 9%<br>(8 total)	OGC (5)<br>UCP2-3 (1)<br>(6 total)	92±7<br>88	57±6<br>52	OGC 84%<br>UCP2-3 16%<br>(11 total)	OGC (4)
**9**	UCP2-3 (7)<br>SAMCX (2)<br>SAMC (2)<br>(11 total)	83±13<br>83±6<br>72±0	56±5<br>60±4<br>61±2	UCP2-3 63%<br>SAMC 19%<br>SAMCX 18%<br>(18 total)	UCP2-3 (10)<br>SAMCX (1)<br>(11 total)	83±11<br>77	55±6<br>56	UCP2-3 82%<br>SAMCX 13%<br>SAMC 5%<br>(21 total)	UCP2-3 (4)
**9 OLD**	UCP2-3 (9)<br>SAMC (2)<br>(11 total)	82±12<br>72±0	55±5<br>61±2	UCP2-3 81%<br>SAMC 19%<br>(21 total)	UCP2-3 (11)<br>(11 total)	82±11	55±6	UCP2-3 95%<br>SAMC 5%<br>(19 total)	UCP2-3 (4)
**10**	UCP2-3 (6)<br>MFRN (2)<br>CIC (1)<br>(9 total)	49±8<br>44±0<br>60	41±6<br>37±5<br>47	UCP2-3 73%<br>MFRN 15%<br>2 other ≤ 7%<br>(11 total)	CIC (3.5)<br>UCP2-3 (2.5)<br>MFRN (2)<br>2 other (0.5)<br>(9 total)	53±5<br>53±2<br>44±0<br>35	45±6<br>44±5<br>40±3<br>37	UCP2-3 35%<br>CIC 32%<br>MFRN 20%<br>3 other ≤ 5%<br>(14 total)	UCP2-3 (2)
**10 OLD**	UCP2-3 (7)<br>MFRN (2)<br>(9 total)	50±8<br>44±0	43±7<br>37±5	UCP2-3 80%<br>MFRN 15%<br>ORC 5%<br>(10 total)	UCP2-3 (5)<br>MFRN (2)<br>TPC (2)<br>(9 total)	54±2<br>53±2<br>39±2	46±5<br>44±5<br>37±5	UCP2-3 62%<br>MFRN 20%<br>TPC 18%<br>(9 total)	UCP2-3 (4)
**11**	OGC (6)<br>A35-A35 (3)<br>(9 total)	97±7<br>99±10	50±4<br>46±2	OGC 73%<br>OAC 27%<br>(22 total)	OGC (6)<br>A35-A35 (3)<br>(9 total)	97±7<br>99±10	50±4<br>46±2	OGC 73%<br>OAC 27%<br>(22 total)	OGC (4)
**11 OLD**	OGC (9)<br>(9 total)	97±7	48±5	OGC 100%<br>(18 total)	OGC (9)<br>(9 total)	97±7	48±5	OGC 100%<br>(18 total)	OGC (5)

**OGC subfamily exons**	**most similar ES**	**most similar FLS**	**final prediction**

	top ES hits (number)	AAS	ASI (%)	top 5% ES hits in % of summed AS (number)	top FLS hits (number)	AAS	ASI (%)	top 5% FLS hits in % of summed AS (number)	subfamily hit (confidence score)
**1**	CIC (5)<br>AAC (2)<br>5 other (1)<br>(12 total)	41±3<br>43±2<br>47-61	39±2<br>33±3<br>32-44	CIC 37%<br>AAC 15%<br>6 other ≤ 11%<br>(12 total)	AAC (4)<br>CIC (3)<br>5 other (1)<br>(12 total)	54±11<br>53±12<br>35-56	32±2<br>39±6<br>30-39	AAC 38%<br>CIC 27%<br>6 other ≤ 9%<br>(15 total)	-
**1 OLD**	AAC (2)<br>UCP4 (1.5)<br>9 other (≤ 1)<br>(12 total)	41±3<br>46±16<br>30-56	39±2<br>35±1<br>30-44	AAC 16%<br>UCP4 14%<br>9 other ≤ 10%<br>(13 total)	AAC (4)<br>CoC (2)<br>6 other (1)<br>(12 total)	54±11<br>48±9<br>35-61	32±2<br>41±6<br>30-39	AAC 41%<br>CoC 17%<br>7 other ≤ 11%<br>(16 total)	-
**2**	OAC (2)<br>PiC (1)<br>AGC (1)<br>(4 total)	128±1<br>124<br>138	40±3<br>43<br>41	OAC 45%<br>AGC 32%<br>2 other ≤ 14%<br>(15 total)	OAC (4)<br>(4 total)	137±3	42±1	OAC 77%<br>UCP4 18%<br>PiC 5%<br>(13 total)	OAC (2)
**2 OLD**	PiC (2)<br>AGC (1)<br>ALC (1)<br>(4 total)	125±1<br>138<br>122	43±0<br>41<br>39	PiC 38%<br>ALC 35%<br>AGC 27%<br>(10 total)	UCP4 (2)<br>PiC (1)<br>AGC (1)<br>(4 total)	132±4<br>126<br>124	40±1<br>43<br>41	UCP4 22%<br>PiC 21%<br>5 other ≤ 17%<br>(15 total)	-
**3**	DIC (3)<br>(3 total)	158±3	40±1	DIC 100%<br>(4 total)	DIC (3)<br>(3 total)	162±5	43±2	DIC 100%<br>(4 total)	DIC (2)
**3 OLD**	DIC (3)<br>(3 total)	158±3	40±1	DIC 100%<br>(4 total)	DIC (3)<br>(3 total)	162±5	43±2	DIC 100%<br>(4 total)	DIC (2)
**4**	DIC (5)<br>(5 total)	99±5	50±2	DIC 100%<br>(5 total)	DIC (4)<br>CoC (1)<br>(5 total)	91±3<br>76	52±2<br>47	DIC 91%<br>CoC 9%<br>(10 total)	DIC (4)
**4 OLD**	DIC (5)<br>(5 total)	99±5	50±2	DIC 100%<br>(5 total)	DIC (4)<br>CoC (1)<br>(5 total)	91±3<br>76	52±2<br>47	DIC 91%<br>CoC 9%<br>(10 total)	DIC (4)
**5**	UCP2-3 (3)<br>UCP4 (2)<br>DIC (1)<br>(6 total)	63±2<br>73±6<br>84	44±1<br>44±5<br>55	UCP2-3 62%<br>DIC 20%<br>2 other ≤ 13%<br>(21 total)	UCP2-3 (5)<br>DIC (1)<br>(6 total)	80±10<br>83	56±5<br>52	UCP2-3 74%<br>DIC 24%<br>(8 total)	UCP2-3 (3)
**5 OLD**	UCP2-3 (3)<br>UCP4 (2)<br>DIC (1)	63±2<br>73±6<br>84	44±1<br>44±5<br>55	UCP2-3 62%<br>DIC 20%<br>2 other ≤ 13%	UCP2-3 (5)<br>DIC (1)<br>(6 total)	80±10<br>83	56±5<br>52	UCP2-3 74%<br>DIC 24%<br>(8 total)	UCP2-3 (3)

	(6 total)			(21 total)					
**6**	DIC (5)<br>UCP2-3 (2)<br>OAC (1)<br>AT4G03115 (1)<br>(9 total)	93±5<br>92±3<br>146<br>88	47±3<br>52±0<br>34<br>49	DIC 53%<br>UCP2-3 21%<br>2 other ≤ 16%<br>(11 total)	DIC (5.5)<br>UCP2-3 (2.5)<br>AT4G03115 (1)<br>(9 total)	107±24<br>96±3<br>88	48±6<br>51±1<br>49	DIC 66%<br>UCP2-3 29%<br>AT4G03115 5%<br>(17 total)	DIC (4)
**6 OLD**	DIC (7)<br>UCP2-3 (2)<br>(9 total)	97±12<br>92±3	44±6<br>52±0	DIC 67%<br>UCP2-3 21%<br>UCP4 12%<br>(12 total)	DIC (6.5)<br>UCP2-3 (2.5)<br>(9 total)	104±24<br>96±3	48±6<br>51±1	DIC 68%<br>UCP2-3 29%<br>UCP4 3%<br>(17 total)	DIC (4)
**7**	GC (6)<br>(6 total)	32±3	42±4	GC 100%<br>(6 total)	DIC (3)<br>GC (3)<br>(6 total)	37±3<br>33±2	39±3<br>51±3	DIC 53%<br>GC 47%<br>(6 total)	GC (2)<br>DIC (1)
**7 OLD**	UCP2-3 (3)<br>AAC (2)<br>(5 total)	22±2*<br>25±4	35±0<br>38±3	UCP2-3 56%<br>AAC 43%<br>(5 total)	DIC (3)<br>UCP4 (3)<br>(6 total)	37±3*<br>24±0	39±3<br>35±0	DIC 61%<br>UCP4 39%<br>(6 total)	DIC (2)
**8**	DIC (6)<br>(6 total)	141±7	40±1	DIC 92%<br>OAC 8%<br>(9 total)	DIC (5)<br>OAC (1)<br>(6 total)	146±9<br>144	42±1<br>40	DIC 69%<br>OAC 25%<br>UCP5-6 5%<br>(14 total)	DIC (4)
**8 OLD**	DIC (6)<br>(6 total)	141±7	40±1	DIC 100%<br>(9 total)	DIC (6)<br>(6 total)	145±9	41±2	DIC 100%<br>(11 total)	DIC (4)

**pOGC subfamily exons**	**most similar ES**				**most similar FLS**				**final prediction**
	**top ES hits (number)**	**AAS**	**ASI (%)**	**top 5% ES hits in % of summed AS (number)**	**top FLS hits (number)**	**AAS**	**ASI (%)**	**top 5% FLS hits in % of summed AS (number)**	**subfamily hit (confidence score)**
**1**	GlyC (2)<br>SLC25A44 (1)<br>(3 total)	86±9<br>86	36±0<br>30	GlyC 69%<br>2 other ≤ 16%<br>(4 total)	GlyC (2)<br>ALC (1)<br>(3 total)	86±9<br>85	35±2<br>32	GlyC 67%<br>ALC 33%<br>(3 total)	GlyC (1)
**1 OLD**	GlyC (2)<br>SLC25A44 (1)	86±9<br>86	36±0<br>30	GlyC 69%<br>2 other ≤ 16%	GlyC (2)<br>ALC (1)	86±9<br>85	35±2<br>32	GlyC 67%<br>ALC 33%	GlyC (1)

	(3 total)			(4 total)	(3 total)			(3 total)	
**2**	PiC (2)<br>(2 total)	68±1	41±2	PiC 100%<br>(3 total)	PiC (2)<br>(2 total)	63±4	45±2	PiC 100%<br>(3 total)	-
**2 OLD**	PiC (2)<br>(2 total)	68±1	41±2	PiC 100%<br>(3 total)	PiC (2)<br>(2 total)	63±4	45±2	PiC 100%<br>(3 total)	-
**3**	DIC (2)<br>(2 total)	67±5	62±0	DIC 100%<br>(6 total)	DIC (2)<br>(2 total)	62±1	62±0	DIC 100%<br>(16 total)	DIC (2)
**3 OLD**	DIC (2)<br>(2 total)	67±5	62±0	DIC 100%<br>(6 total)	DIC (2)<br>(2 total)	62±1	62±0	DIC 100%<br>(16 total)	DIC (2)
**4**	DIC (2)<br>(2 total)	276±9*	40±1	DIC 100%<br>(2 total)	DIC (2)<br>(2 total)	30<br>6±5*	44±2	DIC 100%<br>(9 total)	DIC (1)
**4 OLD**	DIC (2)<br>(2 total)	276±9*	40±1	DIC 100%<br>(2 total)	DIC (2)<br>(2 total)	30<br>6±5*	44±2	DIC 100%<br>(9 total)	DIC (1)
**5**	CAC (2)<br>(2 total)	131±6	32±1	CAC 69%<br>GC 16%<br>DIC 15%<br>(5 total)	OAC (1)<br>SLC25A45/A47/A48 (1)<br>(2 total)	154<br>152	37<br>34	OAC 42%<br>DIC 41%<br>SLC25A45/A47/A48 17%<br>(5 total)	-
**5 OLD**	DIC (1)<br>UCP2-3 (1)<br>(2 total)	120<br>117	34<br>30	DIC 51%<br>UCP2-3 25%<br>MFRN 25%<br>(3 total)	DIC (2)<br>(2 total)	146±1	40±1	DIC 100%<br>(5 total)	DIC (1)
**6**	OAC (1)<br>MCART (1)<br>(2 total)	35<br>25	33<br>33	-	OAC (1)<br>MCART (1)<br>(2 total)	35<br>25	33<br>33	-	-
**6 OLD**	-	-	-	-	peANT (1)<br>(1 total)	21	33	-	-

d. MC-AAP cluster subfamilies

**ALC subfamily exons**	**most similar ES**	**most similar FLS**	**final prediction**

	top ES hits (number)	AAS	ASI (%)	top 5% ES hits in % of summed AS (number)	top FLS hits (number)	AAS	ASI (%)	top 5% FLS hits in % of summed AS (number)	subfamily hit (confidence score)
**1**	ORC (10)<br>2 other<br>(12 total)	50±5<br>30-46	47±8<br>33-80	ORC 87%<br>3 other ≤ 8%<br>(16 total)	ORC (9)<br>2 other<br>(11 total)	47±5<br>29-43	57±7<br>33-80	ORC 86%<br>2 other ≤ 9%<br>(12 total)	ORC (4)
**1 OLD**	ORC (11)<br>PiC (1)<br>(12 total)	48±6<br>30	48±8	ORC 95%<br>PiC 5%<br>(14 total)	ORC (10)<br>PiC (1)<br>(11 total)	46±6	58±8	ORC 98%<br>PiC 2%<br>(13 total)	ORC (4)
**2**	SLC25A45/A47/A48 (7)<br>(7 total)	62±4*	74±5	SLC25A45/A47/48 97%<br>CAC 3%<br>(22 total)	YMC/BOU (4)<br>ORC (2)<br>SLC25A45/A47/A48 (1)<br>(7 total)	52±0*<br>48±1<br>64	73±3<br>82±4<br>86	SLC25A45/A47/A48 50%<br>ORC 33%<br>YMC 17%<br>(21 total)	SLC25A45/A47/A48 (2)
**2 OLD**	ORC (6)<br>YMC/BOU (1)<br>(7 total)	52±3<br>51	80±3<br>79	ORC 88%<br>YMC/BOU 12%<br>(8 total)	YMC/BOU (5)<br>ORC (2)<br>(7 total)	52±1<br>48±1	74±3<br>82±4	YMC/BOU 66%<br>ORC 34%	ORC (2)<br>YMC/BOU (2)
**3**	SLC25A45/A47/A48 (4)<br>CAC (3)<br>(7 total)	82±4<br>83±1	46±6<br>38±2	SLC25A45/A47/A48 64%<br>CAC 36%<br>(16 total)	SLC25A45/A47/A48 (4)<br>3 other (1)<br>(7 total)	80±1<br>64-76	45±2<br>36-43	SLC25A45/A47/A48 66%<br>UCP2-3 19%<br>2 other ≤ 7%<br>(12 total)	SLC25A45/A47/A48 (4)
**3 OLD**	SFC (2)<br>TPC (2)<br>3 other (1)<br>(7 total)	66±4<br>61±3<br>59-77	38±2<br>41±2<br>44-50	TPC 30%<br>SFC 20%<br>4 other ≤ 17%<br>(10 total)	UCP2-3 (3)<br>AAC (2)<br>2 other (1)<br>(7 total)	68±7<br>76±0<br>70-74	43±3<br>36±0<br>39-43	UCP2-3 41%<br>AAC 30%<br>2 other ≤ 15%<br>(7 total)	-
**4**	YMC/BOU (4)<br>(4 total)	399±44*	31±0	YMC/BOU 100%<br>(4 total)	SLC25A45/A47/A48 (4)<br>(4 total)	51<br>6±19*	30±1	SLC25A45/A47/A48 100%<br>(6 total)	SLC25A45/A47/A48 (2)
**4 OLD**	YMC/BOU (4)<br>(4 total)	399±44	31±0	YMC/BOU 100%<br>(4 total)	YMC/BOU (1)<br>(1 total)	474	31	-	YMC/BOU (2)

**pALC subfamily exons**	**most similar ES**				**most similar FLS**				**final prediction**
	**top ES hits**	**AAS**	**ASI**	**top 5% ES hits in**	**top FLS hits**	**AAS**	**ASI**	**top 5% FLS hits**	**subfamily hit**

	(number)		(%)	% of summed AS (number)	(number)		(%)	in % of summed AS (number)	(confidence score)
**1**	ORC (1)<br>YMC/BOU (1)<br>(2 total)	191<br>162	33<br>33	ORC 50%<br>YMC/BOU 50%<br>(4 total)	CAC (2)<br>(2 total)	183±12	34±2	CAC 92%<br>ORC 8%<br>(8 total)	CAC (1)
**1 OLD**	ORC (1)<br>YMC-2 (1)<br>(2 total)	191<br>162	33<br>33	ORC 50%<br>YMC/BOU 50%<br>(4 total)	ORC (1)<br>YMC/BOU (1)<br>(2 total)	191<br>162	33<br>33	ORC 50%<br>YMC/BOU 50%<br>(4 total)	-
**2**	YMC/BOU (0.5)<br>ORC (0.5<br>(1 total)	340<br>340	31<br>31	YMC/BOU 66%<br>ORC 34%<br>(3 total)	SLC25A45/<br>A47/A48 (2)<br>(2 total)	480±33	33±3	SLC25A45/A47/<br>A48 100%<br>(3 total)	-
**2 OLD**	YMC/BOU (0.5)<br>ORC (0.5)<br>(1 total)	340<br>340	31<br>31	YMC/BOU 66%<br>ORC 34%<br>(3 total)	-	-	-	-	-

**SLC25A45/A47 isoform exons**	**most similar ES**				**most similar FLS**				**final prediction**
	**top ES hits (number)**	**AAS**	**ASI (%)**	**top 5% ES hits in % of summed AS (number)**	**top FLS hits (number)**	**AAS**	**ASI (%)**	**top 5% FLS hits in % of summed AS (number)**	**subfamily hit (confidence score)**
**1**	ALC (3)<br>ORC (2)<br>SLC25A39-A40 (2)<br>1 other (1)<br>(8 total)	45±1<br>49±2<br>31±2<br>31	77±3<br>66±4<br>50±4<br>31	ALC 42%<br>ORC 30%<br>3 other ≤ 14%<br>(10 total)	ALC (3)<br>ORC (2)<br>3 other (1)<br>(8 total)	42±1<br>49±2<br>29-30	77±3<br>66±4<br>46-54	ALC 41%<br>ORC 32%<br>4 other ≤ 13%<br>(10 total)	-
**1 OLD**	ALC (3)<br>ORC (2)<br>SLC25A39-A40 (2)<br>1 other (1)<br>(8 total)	45±1<br>49±2<br>31±2<br>31	77±3<br>66±4<br>50±4<br>31	ALC 42%<br>ORC 30%<br>3 other ≤ 14%<br>(10 total)	ALC (3)<br>ORC (2)<br>3 other (1)<br>(8 total)	42±1<br>49±2<br>29-30	77±3<br>66±4<br>46-54	ALC 41%<br>ORC 32%<br>4 other ≤ 13%<br>(10 total)	-
**2**	ALC (7)<br>ORC (1)<br>(8 total)	60±3<br>44	72±3<br>57	ALC 91%<br>ORC 9%<br>(16 total)	ALC (6)<br>CAC (1)<br>ORC (1)<br>(8 total)	50±3<br>47<br>39	73±3<br>79<br>57	ALC 77%<br>CAC 12%<br>ORC 10%<br>(21 total)	ALC (4)

**2 OLD**	ALC (7)<br>ORC (1)<br>(8 total)	60±3<br>44	72±3<br>57	ALC 91%<br>ORC 9%<br>(16 total)	ALC (6)<br>CAC (1)<br>ORC (1)<br>(8 total)	50±3<br>47<br>39	73±3<br>79<br>57	ALC 77%<br>CAC 12%<br>ORC 10%<br>(21 total)	ALC (4)
**3**	ALC (4)<br>CAC (3)<br>AAC (1)<br>(8 total)	81±4<br>77±4<br>57	42±8<br>43±4<br>45	ALC 53%<br>CAC 37%<br>AAC 9%<br>(11 total)	ALC (4)<br>4 other (1)<br>(8 total)	68±8<br>57-71	57±6<br>33-46	ALC 40%<br>CAC 21%<br>4 other ≤ 14%<br>(13 total)	ALC (1)
**3 OLD**	ALC (4)<br>CAC (3)<br>AAC (1)<br>(8 total)	81±4<br>77±4<br>57	42±8<br>43±4<br>45	ALC 53%<br>CAC 37%<br>AAC 9%<br>(11 total)	ALC (4)<br>4 other (1)<br>(8 total)	68±8<br>57-71	57±6<br>33-46	ALC 40%<br>CAC 21%<br>4 other ≤ 14%<br>(13 total)	ALC (1)
**4**	ALC (5)<br>ORC (1)<br>(6 total)	109±17<br>88	32±2<br>31	ALC 86%<br>ORC 14%<br>(6 total)	ALC (5)<br>ORC (1)<br>(6 total)	108±8<br>88	32±2<br>31	ALC 86%<br>ORC 14%<br>(7 total)	ALC (4)
**4 OLD**	ALC (5)<br>ORC (1)<br>(6 total)	109±17<br>88	32±2<br>31	ALC 86%<br>ORC 14%<br>(6 total)	ALC (5)<br>ORC (1)<br>(6 total)	108±8<br>88	32±2<br>31	ALC 86%<br>ORC 14%<br>(7 total)	ALC (4)
**5**	SFC (2)<br>4 other (1)<br>(6 total)	182±24<br>162-<br>177	35±2<br>30-41	SFC 35%<br>ALC 23%<br>CAC 21%<br>3 other ≤ 8%<br>(9 total)	CAC (6)<br>ALC (2)<br>(8 total)	208±21<br>195±1	35±4<br>36±3	CAC 67%<br>ALC 28%<br>SFC 4%<br>(23 total)	CAC (2)
**5 OLD**	SFC (2)<br>4 other (1)<br>(6 total)	182±24<br>162-<br>177	35±2<br>30-41	SFC 35%<br>ALC 23%<br>CAC 21%<br>3 other ≤ 8%<br>(9 total)	CAC (6)<br>ALC (2)<br>(8 total)	208±21<br>195±1	35±4<br>36±3	CAC 67%<br>ALC 28%<br>SFC 4%<br>(23 total)	CAC (2)
**6**	ALC (8)<br>(8 total)	246±12	44±4	ALC 100%<br>(16 total)	ALC (8)<br>(8 total)	247±13	44±4	ALC 100%<br>(16 total)	ALC (5)
**6 OLD**	ALC (8)<br>(8 total)	246±12	44±4	ALC 100%<br>(16 total)	ALC (8)<br>(8 total)	247±13	44±4	ALC 100%<br>(16 total)	ALC (5)

**SLC25A48 isoform exons**	**most similar ES**	**most similar FLS**	**final prediction**

	top ES hits (number)	AAS	ASI (%)	top 5% ES hits in % of summed AS (number)	top FLS hits (number)	AAS	ASI (%)	top 5% FLS hits in % of summed AS (number)	subfamily hit (confidence score)
**1**	ALC (1)<br>SAMC (1)<br>other 2 (<1)<br>(3 total)	42<br>35	44<br>31	ALC 43%<br>SAMC 32%<br>3 other ≤ 11%<br>(5 total)	ALC (1)<br>AAC (1)<br>SAMC (1)<br>(3 total)	40<br>40<br>32	44<br>31<br>31	AAC 33%<br>ALC 26%<br>2 other ≤ 7%<br>(5 total)	-
**1 OLD**	ALC (1)<br>SAMC (1)<br>other 2 (<1)<br>(3 total)	42<br>35	44<br>31	ALC 43%<br>SAMC 32%<br>3 other ≤ 11%<br>(5 total)	ALC (1)<br>AAC (1)<br>SAMC (1)<br>(3 total)	40<br>40<br>32	44<br>31<br>31	AAC 33%<br>ALC 26%<br>2 other ≤ 7%<br>(5 total)	-
**2**	ALC (4)<br>(4 total)	65±2	77±3	ALC 100%<br>(12 total)	ALC (4)<br>(4 total)	50±2	77±3	ALC 96%<br>CAC 4%<br>(21 total)	ALC (4)
**2 OLD**	ALC (4)<br>(4 total)	65±2	77±3	ALC 100%<br>(12 total)	ALC (4)<br>(4 total)	50±2	77±3	ALC 96%<br>CAC 4%<br>(21 total)	ALC (4)
**3**	ALC (2)<br>MFRN (1)<br>ODC (1)<br>(4 total)	60±8<br>53<br>46	33<br>42<br>38	ALC 54%<br>MFRN 24%<br>ODC 21%<br>(4 total)	MFRN (2)<br>AAC (1)<br>ODC (1)<br>(4 total)	54±1<br>64<br>46	42±0<br>50<br>38	AAC 46%<br>MFRN 33%<br>ODC 21%<br>(6 total)	ALC (1)
**3 OLD**	ALC (2)<br>MFRN (1)<br>ODC (1)<br>(4 total)	60±8<br>53<br>46	33<br>42<br>38	ALC 54%<br>MFRN 24%<br>ODC 21%<br>(4 total)	MFRN (2)<br>AAC (1)<br>ODC (1)<br>(4 total)	54±1<br>64<br>46	42±0<br>50<br>38	AAC 46%<br>MFRN 33%<br>ODC 21%<br>(6 total)	ALC (1)
**4**	ALC (3)<br>(3 total)	174±3*	34±1	ALC 84%<br>SLC25A45 16%<br>(4 total)	ALC (3)<br>(3 total)	171±10	34±1	ALC 100%<br>(4 total)	ALC (2)
**4 OLD**	ALC (3)<br>(3 total)	174±3	34±1	ALC 100%<br>(3 total)	ALC (3)<br>(3 total)	171±10	34±1	ALC 100%<br>(4 total)	ALC (2)
**5**	-	-	-	-	CAC (3)<br>(3 total)	179±16	35±2	CAC 100%<br>(5 total)	CAC (1)
**5 OLD**	-	-	-	-	CAC (3)<br>(3 total)	179±16	35±2	CAC 100%<br>(5 total)	CAC (1)
**6**	ALC (4)	143±7	51±3	ALC 100%	ALC (4)	143±7	51±3	ALC 100%	ALC (5)

	(4 total)			(5 total)	(4 total)			(5 total)	
**6 OLD**	ALC (4)<br>(4 total)	143±7	51±3	ALC 100%<br>(5 total)	ALC (4)<br>(4 total)	143±7	51±3	ALC 100%<br>(5 total)	ALC (5)
**7**	ALC (1)<br>MFT/FAD (1)<br>CAC (1)<br>CoC (1)<br>(4 total)	80<br>90<br>78<br>66	38<br>38<br>38<br>41	ALC 26%<br>MFT/FAD 26%<br>CAC 26%<br>CoC 22%<br>(5 total)	CAC (1)<br>ALC (1)<br>MFT/FAD (1)<br>ORC (1)<br>(4 total)	83<br>80<br>80<br>79	33<br>41<br>38<br>35	CAC 58%<br>ALC 19%<br>other 2 ≤14%<br>(9 total)	CAC (1)
**7 OLD**	ALC (1)<br>MFT/FAD (1)<br>CAC (1)<br>CoC (1)<br>(4 total)	80<br>90<br>78<br>66	38<br>38<br>38<br>41	ALC 26%<br>MFT/FAD 26%<br>CAC 26%<br>CoC 22%<br>(5 total)	CAC (1)<br>ALC (1)<br>MFT/FAD (1)<br>ORC (1)<br>(4 total)	83<br>80<br>80<br>79	33<br>41<br>38<br>35	CAC 58%<br>ALC 19%<br>other 2 ≤14%<br>(9 total)	CAC (1)

**CAC subfamily exons**	**most similar ES**				**most similar FLS**				**final prediction**
	**top ES hits (number)**	**AAS**	**ASI (%)**	**top 5% ES hits in % of summed AS (number)**	**top FLS hits (number)**	**AAS**	**ASI (%)**	**top 5% FLS hits in % of summed AS (number)**	**subfamily hit (confidence score)**
**1**	ALC (9)<br>MFT/FAD (1)<br>OGC (1)<br>(11 total)	94±7<br>84<br>88	44±3<br>43<br>39	ALC 83%<br>2 other ≤ 9%<br>(13 total)	ALC (8)<br>3 other (1)<br>(11 total)	96±5<br>84-90	43±5<br>33-37	ALC 78%<br>6 other ≤ 7%<br>(22 total)	ALC (4)
**1 OLD**	ALC (9)<br>MFT/FAD (1)<br>OGC (1)<br>(11 total)	94±7<br>84<br>88	44±3<br>43<br>39	ALC 83%<br>2 other ≤ 9%<br>(13 total)	ALC (8)<br>3 other (1)<br>(11 total)	96±5<br>84-90	43±5<br>33-37	ALC 79%<br>5 other ≤ 7%<br>(21 total)	ALC (4)
**2**	YMC/BOU (5)<br>ALC (1)<br>ORC (1)<br>(7 total)	97±5<br>84<br>81	56±3<br>39<br>38	YMC/BOU (75%)<br>ALC 13%<br>ORC 12%<br>(8 total)	YMC/BOU (5)<br>DIC (2)<br>(7 total)	96±5<br>87±1	56±2<br>47±2	YMC/BOU 74%<br>DIC 26%<br>(8 total)	YMC/BOU (4)
**2 OLD**	YMC/BOU (5)<br>ALC (1)<br>ORC (1)<br>(7 total)	97±5<br>84<br>81	56±3<br>39<br>38	YMC/BOU (75%)<br>ALC 13%<br>ORC 12%<br>(8 total)	YMC/BOU (5)<br>DIC (2)<br>(7 total)	96±5<br>87±1	56±2<br>47±2	YMC/BOU 74%<br>DIC 26%<br>(8 total)	YMC/BOU (4)

**3**	ALC (3)<br>4 other (1)<br>(7 total)	78±3<br>77-86	32±1<br>35-39	ALC 28%<br>PNC 21%<br>4 other ≤ 16%<br>(9 total)	AGC (3)<br>4 other (1)<br>(7 total)	96±6<br>91-103	34±1<br>39-44	AGC 37%<br>SLC25A16 29%<br>4 other ≤ 13%<br>(11 total)	-
**3 OLD**	ALC (5)<br>2 other (1)<br>(7 total)	77±3*<br>79-86	32±1<br>37-39	ALC 47%<br>CoC 21%<br>4 other ≤ 16%<br>(13 total)	AGC (3)<br>CoC (2)<br>SAMC (2)<br>(7 total)	96±6*<br>96±7<br>91±0	34±1<br>43±1<br>37±2	AGC 36%<br>CoC 36%<br>SAMC 27%<br>(8 total)	-
**4**	ORC (3)<br>GlyC (3)<br>(6 total)	68±6<br>66±0	36±2<br>37±0	ORC 42%<br>GlyC 28%<br>YPR011C 27%<br>MFRN 3%<br>(13 total)	ORC (6)<br>(6 total)	67±4	34±2	ORC 68%<br>MCART 28%<br>MFRN 4%<br>(12 total)	ORC (2)
**4 OLD**	ORC (3)<br>GlyC (3)<br>(6 total)	68±6<br>66±0	36±2<br>37±0	ORC 42%<br>GlyC 28%<br>YPR011C 27%<br>MFRN 3%<br>(13 total)	ORC (6)<br>(6 total)	67±4	34±2	ORC 95%<br>MFRN 5%<br>(8 total)	ORC (2)
**5**	ALC (2)<br>YMC/BOU (2)<br>3 other (1)<br>(7 total)	100±2<br>93±1<br>93-98	39±4<br>42±6<br>31-49	ALC 32%<br>7 other ≤ 16%<br>(16 total)	ALC (4.5)<br>YMC/BOU (2)<br>GC (0.5)<br>(7 total)	97±5<br>100±6<br>95	42±5<br>38±2<br>43	ALC 42%<br>YMC/BOU 21%<br>9 other ≤ 8%<br>(26 total)	ALC (1)
**5 OLD**	YMC/BOU (3)<br>ALC (2)<br>2 other (1)<br>(7 total)	93±1<br>100±2<br>95-96	40±6<br>39±4<br>43-49	ALC 34%<br>YMC/BOU 33%<br>2 other ≤ 19%<br>(10 total)	ALC (5)<br>YMC/BOU (2)<br>(7 total)	97±5<br>100±6	42±5<br>38±2	ALC 49%<br>YMC/BOU 27%<br>4 other ≤ 7%<br>(16 total)	ALC (1)
**6**	SLC25A45/A47/A48 (4) #<br>APCX (2)<br>(6 total)	61±2<br>65±0	49±2<br>54±0	SLC25A45/A47/<br>A48 57% #<br>APCX 43%<br>(7 total)	SLC25A45/A47/A48 (4)<br>#<br>APCX (2)<br>(6 total)	61±2<br>65±0	49±2<br>54±0	SLC25A45/A47/A48<br>57% #<br>APCX 43%<br>(7 total)	SLC25A45/<br>A47/A48 (4) #
**6 OLD**	ALC (4)<br>2 other (1)<br>(6 total)	55±4<br>53-62	40±2<br>33-46	ALC 51%<br>MFRN 34%<br>3 other 11%<br>(14 total)	ALC (2)<br>4 other (1)<br>(6 total)	57±4<br>53-62	40±2<br>42-50	MFRN 26%<br>ALC 24%<br>3 other ≤ 17%<br>(7 total)	ALC (2)
**7**	SLC25A45/A47/A48 (4)<br>ALC (3)<br>(7 total)	101±4<br>98±9	51±1<br>46±0	SLC25A45/A47/<br>A48 53%<br>ALC 47%	ALC (5)<br>SLC25A45/A47/48 (2)<br>(7 total)	100±5<br>98±2	46±2<br>49±6	SLC25A45/A47/A48<br>52%<br>ALC 48%	SLC25A45/<br>A47/A48 (3)<br>ALC (1)

				(11 total)				(16 total)	
**7 OLD**	ALC (7)<br>(7 total)	97±7	45±3	ALC 100%<br>(13 total)	ALC (7)<br>(7 total)	97±7	45±3	ALC 97%<br>ORC 3%<br>(14 total)	ALC (4)
**8**	YMC/BOU (5)<br>2 other (1)<br>(7 total)	108±8<br>108-111	44±3<br>39-42	YMC/BOU 71%<br>2 other ≤ 15%<br>(9 total)	YMC/BOU (3)<br>ORC (2)<br>2 other (1)<br>(7 total)	111±10<br>105±1<br>106-108	45±3<br>46±0<br>42	ORC 40%<br>YMC/BOU 36%<br>4 other ≤ 14%<br>(15 total)	YMC/BOU (2)
**8 OLD**	YMC/BOU (5)<br>2 other (1)<br>(7 total)	108±8<br>108-111	44±3<br>39-42	YMC/BOU 71%<br>2 other ≤ 15%<br>(9 total)	YMC/BOU (3)<br>ORC (2)<br>2 other (1)<br>(7 total)	111±10<br>105±1<br>106-108	45±3<br>46±0<br>42	ORC 40%<br>YMC/BOU 36%<br>4 other ≤ 14%<br>(15 total)	YMC/BOU (2)
**9**	ODC (2)<br>ORC (2)<br>3 other (1)<br>(7 total)	41±3<br>28±1<br>38-41	30±0<br>35±0<br>30-40	ODC 32%<br>ORC 22%<br>3 other ≤ 16%<br>(10 total)	SAMCX (2)<br>ODC (2)<br>3 other (1)<br>(7 total)	44±2<br>41±3<br>38-41	35±0<br>33±3<br>30-40	SAMCX 30%<br>ODC 28%<br>3 other ≤ 14%<br>(9 total)	-
**9 OLD**	ORC (5)<br>2 other (1)<br>(7 total)	31±4<br>30-38	38±4<br>30-40	ORC 70%<br>ALC 17%<br>UCP2-3 14%<br>(11 total)	ORC (2)<br>AAC (2)<br>3 other (1)<br>(7 total)	33±5<br>31±2<br>31-38	40±5<br>30±0<br>30-35	ORC 36%<br>AAC 27%<br>3 other ≤ 17%<br>(10 total)	ORC (2)

**ORC subfamily exons**	**most similar ES**				**most similar FLS**				**final prediction**
	**top ES hits (number)**	**AAS**	**ASI (%)**	**top 5% ES hits in % of summed AS (number)**	**top FLS hits (number)**	**AAS**	**ASI (%)**	**top 5% FLS hits in % of summed AS (number)**	**subfamily hit (confidence score)**
**1**	ALC (4)<br>TPC (2)<br>6 other (1)<br>(12 total)	45±10<br>39±1<br>35-43	48±9<br>43±4<br>32-53	ALC 36%<br>TPC 12%<br>OGC 12%<br>5 other ≤ 9%<br>(18 total)	OGC (2)<br>TPC (2)<br>ALC (2)<br>4 other (1)<br>(10 total)	40±1<br>39±1<br>34±3<br>33-42	47±5<br>43±4<br>33±2<br>37-47	OGC 26%<br>PNC 17%<br>TPC 16%<br>4 other ≤ 11%<br>(15 total)	-
**1 OLD**	ALC (6)<br>TPC (2)<br>3 other (1)<br>(12 total)	42±10<br>39±1<br>35-40	45±9<br>43±4<br>38-53	ALC 54%<br>TPC 14%<br>YMC/BOU 14%<br>2 other ≤ 10%<br>(16 total)	TPC (3)<br>OGC (2)<br>ALC (2)<br>3 other (1)<br>(9 total)	36±4<br>40±1<br>34±3	39±6<br>47±5<br>33±2	OGC 30%<br>TPC 27%<br>ALC 20%<br>3 other ≤ 12%<br>(10 total)	ALC (1)

**2**	YMC/BOU (4)<br>ODC (2)<br>(6 total)	145±15*<br>162±7	34±2<br>31±0	YMC/BOU 59%<br>ODC 36%<br>ALC 5%<br>(12 total)	CAC (3)<br>3 other (1)<br>(6 total)	186±4*<br>170-184	34±2<br>31-34	CAC 58%<br>3 other ≤ 17%<br>(14 total)	CAC (1)
**2 OLD**	YMC/BOU (6)<br>(6 total)	144±12*	33±1	YMC/BOU 95%<br>ALC 5%<br>(14 total)	ALC (4)<br>YMC/BOU (2)<br>(6 total)	165±6*<br>156±11	33±1<br>34±2	ALC 70%<br>YMC/BOU 30%<br>(11 total)	ALC (2)
**3**	YMC/BOU (3)<br>CAC (3)<br>GC (1)<br>(7 total)	82±4*<br>77±5<br>74	34±2<br>32±1<br>32	CAC 49%<br>YMC/BOU 37%<br>GC 13%<br>(8 total)	YMC/BOU (7)<br>(7 total)	90±5*	38±2	YMC/BOU 100%<br>(7 total)	YMC/BOU (2)
**3 OLD**	YMC/BOU (5)<br>SLC25A44 (1)<br>(6 total)	75±5*<br>58	32±1<br>30	YMC/BOU 89%<br>SLC25A44 11%<br>(6 total)	YMC/BOU (7)<br>(7 total)	90±5*	38±2	YMC/BOU 100%<br>(7 total)	YMC/BOU (4)
**4**	ALC (4)<br>YMC/BOU (3)<br>2 other (1)<br>(9 total)	110±15<br>103±4<br>121-128	35±2<br>31±0<br>34	ALC 52%<br>YMC/BOU 27%<br>2 other ≤ 13%<br>(15 total)	ALC (6)<br>AT5G42130 (2)<br>YMC/BOU (1)<br>(9 total)	130±25<br>116±4<br>118	37±5<br>35±2<br>35	ALC 69%<br>AT5G42130 21%<br>2 other ≤ 5%<br>(11 total)	ALC (3)
**4 OLD**	ALC (6)<br>YMC/BOU (3)<br>(9 total)	113±13<br>103±4	34±3<br>31±0	ALC 69%<br>YMC/BOU 31%<br>(13 total)	ALC (6)<br>YMC/BOU (2)<br>(8 total)	130±25<br>118±1	37±5<br>35±0	ALC 77%<br>YMC/BOU 23%<br>(10 total)	ALC (4)
**5**	ALC (4)<br>SLC25A45/<br>A47/A48 (2)<br>4 other (1)<br>(10 total)	122±13<br>131±4<br>91-132	35±3<br>37±1<br>35-45	ALC 43%<br>SLC25A45/A47/A48<br>22%<br>5 other ≤ 11%<br>(13 total)	ALC (4)<br>6 other (1)<br>(10 total)	122±13	35±3	ALC 40%<br>8 other ≤ 12%<br>(19 total)	(ALC)
**5 OLD**	ALC (7)<br>3 other<br>(10 total)	122±10<br>91-132	36±3<br>35-45	ALC 75%<br>3 other ≤ 12%<br>(12 total)	ALC (6)<br>4 other (1)<br>(10 total)	122±11<br>109-132	35±3<br>32-45	ALC 72%<br>4 other ≤ 11%<br>(15 total)	ALC (4)
**6**	CAC (6)<br>YMC/BOU (3)<br>3 other (1)<br>(12 total)	100±9<br>85±2<br>81-96	44±2<br>40±3<br>38-43	CAC 61%<br>YMC/BOU 16%<br>6 other ≤ 10%<br>(23 total)	CAC (8.5)<br>4 other (≤1)<br>(12 total)	102±8<br>82-100	45±3<br>37-44	CAC 75%<br>6 other ≤ 9%<br>(23 total)	CAC (3)
**6 OLD**	YMC/BOU (7)<br>ALC (2)<br>3 other (1)	83±5<br>83±4<br>81-89	40±2<br>36±1<br>39-41	YMC/BOU 67%<br>ALC 13%<br>4 other ≤ 8%	YMC/BOU (6)<br>6 other (1)<br>(12 total)	83±5<br>82-100	40±3<br>35-44	YMC/BOU 46%<br>7 other ≤ 10%<br>(26 total)	YMC/BOU (2)

	(12 total)			(22 total)					

YMC/BOU subfamily exons	most similar ES				most similar FLS				final prediction
	**top ES hits (number)**	**AAS**	**ASI (%)**	**top 5% ES hits in % of summed AS (number)**	**top FLS hits (number)**	**AAS**	**ASI (%)**	**top 5% FLS hits in % of summed AS (number)**	**subfamily hit (confidence score)**
**1**	CAC (1)<br>ORC (1)<br>(2 total)	297<br>289	46<br>33	-	CAC (2)<br>(2 total)	344±16	44±1	CAC 100%<br>(4 total)	CAC (1)
**1 OLD**	ORC (2)<br>(2 total)	293±4	33±1	ORC 100%<br>(3 total)	ORC (2)<br>(2 total)	324±4	35±1	ORC 51%<br>ALC 49%<br>(4 total)	(ORC/ALC)
**2**	ALC (1)<br>CAC (1)<br>(2 total)	340<br>337	31<br>32	-	CAC (2)<br>(2 total)	389±18	36±1	CAC 100%<br>(2 total)	-
**2 OLD**	ALC (2)<br>(2 total)	336±4	32±1	ALC 100%<br>(2 total)	ORC (2)<br>(2 total)	356±7	34±1	ORC 75%<br>ALC 25%<br>(5 total)	ORC (1)

e. MC-AAN cluster subfamilies

AGC subfamily exons	most similar ES				most similar FLS				final prediction
	**top ES hits (number)**	**AAS**	**ASI (%)**	**top 5% ES hits in % of summed AS (number)**	**top FLS hits (number)**	**AAS**	**ASI (%)**	**top 5% FLS hits in % of summed AS (number)**	**subfamily hit (confidence score)**
**1**	APC (4)<br>7 other (≤ 2)<br>(15 total)	32±3<br>30-42	38±4<br>32-42	APC 21%<br>GlyC 16%<br>6 other ≤ 15%<br>(18 total)	APC (7)<br>AACX (2)<br>ODC (2)<br>6 other (≤ 1.5)<br>(15 total)	35±4<br>36±6<br>33±1<br>30-35	41±2<br>39±3<br>39±3<br>32-42	APC 48%<br>ODC 16%<br>7 other ≤ 11%<br>(25 total)	(APC)

**1 OLD**	APC (4)<br>7 other (≤ 2)<br>(15 total)	32±3<br>30-42	38±4<br>32-42	APC 21%<br>GlyC 16%<br>6 other ≤ 15%<br>(18 total)	APC (11)<br>5 other (≤ 1.3)<br>(15 total)	33±4	41±2	APC 74%<br>5 other ≤ 8%<br>(24 total)	APC (2)
**2**	GC (6)<br>(6 total)	166±1	53±0	GC 100%<br>(6 total)	GC (6)<br>(6 total)	174±5	49±2	GC 100%<br>(47 total)	GC (5)
**2 OLD**	OGC (6)<br>(6 total)	132±2	42±1	OGC 67%<br>UCP4 33%<br>(10 total)	OGC (4)<br>2 other (≤ 1.5)<br>(6 total)	145±3<br>138-<br>143	41±2<br>40-42	UCP4 51%<br>OGC 36%<br>2 other ≤ 8%<br>(40 total)	OGC (3)<br>UCP4 (1)
**3**	PNC (6)<br>(6 total)	48±1	50±3	PNC 60%<br>GC 40%<br>(11 total)	GC (6)<br>(6 total)	45±1	54±3	GC 96%<br>PNC 4%<br>(12 total)	PNC (2)<br>GC (2)
**3 OLD**	SLC25A39-A40 (6)<br>(6 total)	42±2	54±2	SLC25A39-A40 77%<br>APC 23%<br>(9 total)	APC (5)<br>SLC25A39-A40 (1)<br>(6 total)	32±1<br>36	56±3<br>59	APC 84%<br>SLC25A39-A40<br>16%<br>(6 total)	SLC25A39-A40<br>(2)<br>APC (2)
**4**	GC (10)<br>(10 total)	59±8	42±4	GC 100%<br>(11 total)	GC (10)<br>(10 total)	67±8	45±5	GC 100%<br>(10 total)	GC (5)
**4 OLD**	OGC (5)<br>SLC25A39-A40 (2)<br>3 other (≤ 1)<br>(9 total)	40±2<br>35±2<br>34-46	33±3<br>31±2<br>30-31	OGC 57%<br>SLC25A39-A40 20%<br>3 other ≤ 10%<br>(11 total)	OGC (9)<br>DIC (1)<br>(10 total)	40±4<br>55	33±4<br>31	OGC 79%<br>DIC 13%<br>2 other ≤ 4%<br>(13 total)	OGC (4)
**5**	GC (11)<br>OAC (1)<br>(12 total)	112±5<br>101	38±2<br>34	GC 90%<br>2 other ≤ 8%<br>(19 total)	GC (8)<br>SLC25A45/A47/A48 (3)<br>OAC (1)<br>(12 total)	116±6<br>113±1<br>117	38±3<br>35±1<br>40	GC 77%<br>3 other ≤ 11%<br>(29 total)	GC (4)
**5 OLD**	MFT/FAD (4)<br>peCFNC (2)<br>ALC (2)<br>4 other (1)<br>(12 total)	97±6<br>92±1<br>87±0<br>88-100	37±2<br>31±1<br>33±1<br>30-34	MFT/FAD 35%<br>peCFNC 25%<br>ALC 12%<br>4 other ≤ 9%<br>(15 total)	OGC (6)<br>YPR011C (2)<br>4 other (1)<br>(12 total)	107±2<br>98±7<br>89-106	37±1<br>37±1<br>30-40	OGC 53%<br>YPR011C 18%<br>6 other ≤ 9%<br>(19 total)	OGC (1)
**6**	CAC (3)<br>SLC25A45/A47/A48<br>(3)<br>3 other (1)	106±0<br>95±4<br>97-107	41±2<br>42±1<br>36-45	SLC25A45/A47/A48<br>39%<br>CAC 36%<br>3 other ≤ 15%	GC (4)<br>CAC (4)<br>SLC25A45/A47/A48 (1)<br>(9 total)	107±9<br>106±6<br>94	42±3<br>42±2<br>43	GC 42%<br>CAC 30%<br>SLC25A45/<br>A47/A48 25%	-

	(9 total)			(14 total)				SLC25A43 4%<br>(18 total)	
**6 OLD**	SLC25A39-A40 (7)<br>SAMC (2)<br>(9 total)	91±9<br>86±8	35±2<br>37±1	SLC25A39-A40 79%<br>SAMC 21%<br>(12 total)	SAMC (7)<br>SLC25A39-A40 (2)<br>(9 total)	93±8<br>91±7	34±3<br>37±1	SAMC 73%<br>SLC25A39-A40 14%<br>2 other ≤ 9%<br>(18 total)	SLC25A39-A40 (2)<br>SAMC (2)
**7**	PNC (4)<br>GC (2)<br>MFRN (1)<br>(7 total)	140±6*<br>137±8<br>144	44±2<br>42±2<br>38	PNC 60%<br>GC 19%<br>3 other ≤ 11%<br>(12 total)	GC (5)<br>NDT (2)<br>(7 total)	163±4*<br>154±9	51±1<br>40±2	GC 77%<br>NDT 12%<br>SAMC 11%<br>(13 total)	GC (2)
**7 OLD**	ALC (4)<br>MFRN (2)<br>SFC (1)<br>(7 total)	128±3*<br>131±14<br>120	37±3<br>36±2<br>36	ALC 28%<br>MFRN 28%<br>SLC25A44 21%<br>4 other ≤ 6%<br>(17 total)	NDT (6)<br>SAMC (1)<br>(7 total)	157±6*<br>155	42±3<br>47	NDT 65%<br>SAMC 35%<br>(11 total)	NDT (2)
**8**	TPC (4)<br>ALC (4)<br>CoC (2)<br>2 other (1)<br>(12 total)	58±0<br>52±4<br>54±0<br>22-54	33±0<br>35±3<br>33±0<br>33-55	TPC 38%<br>ALC 37%<br>CoC 17%<br>2 other ≤ 4%<br>(14 total)	TPC (4)<br>CoC (3)<br>ALC (2.5)<br>3 other (≤ 1)<br>(12 total)	59±0<br>51±4<br>54±2<br>20-46	33±0<br>32±2<br>35±3<br>37-45	TPC 37%<br>ALC 28%<br>CoC 19%<br>3 other ≤ 9%<br>(19 total)	-
**8 OLD**	TPC (6)<br>ALC (4)<br>CoC (2)<br>(12 total)	47±13<br>59±0<br>54±0	37±4<br>33±0<br>33±0	ALC 45%<br>TPC 38%<br>CoC 17%<br>(14 total)	ALC (4.5)<br>TPC (4)<br>CoC (3)<br>YMC/BOU (0.5)<br>(12 total)	47±14<br>59±0<br>51±4<br>54	37±5<br>33±0<br>32±2<br>37	TPC 38%<br>ALC 32%<br>CoC 19%<br>2 other ≤ 9%<br>(20 total)	-

**GC subfamily exons**	**most similar ES**				**most similar FLS**				**final prediction**
	**top ES hits (number)**	**AAS**	**ASI in %**	**top 5% ES hits in % of summed AS (number)**	**top FLS hits (number)**	**AAS**	**ASI in %**	**top 5% FLS hits in % of summed AS (number)**	**subfamily hit (confidence score)**
**1**	AAC (4)<br>10 other (≤ 2)<br>(17 total)	19±2<br>≤32	52±19<br>33-71	AAC 23%<br>MFT/FAD 19%<br>10 other ≤ 12%	AAC (3)<br>11 other (≤ 2.5)<br>(18 total)	25±10<br>≤32	61±15<br>32-71	AAC 21%<br>MFT/FAD 18%<br>11 other ≤ 10%	-

				(22 total)				(21 total)	
**1 OLD**	AAC (4)<br>DIC (3)<br>7 other (≤ 2)<br>(17 total)	19±2<br>19±2	52±19<br>55±17	AAC 23%<br>MFT/FAD 20%<br>9 other ≤ 12%<br>(22 total)	AAC (3)<br>CAC (3)<br>8 other (≤ 2.5)<br>(18 total)	25±10	61±15	AAC 21%<br>MFT/FAD 18%<br>9 other ≤ 11%<br>(20 total)	-
**2**	AGC (9)<br>APC (3)<br>3 other ≤ 2)<br>(17 total)	99±6*<br>101±4<br>≤98	42±4<br>37±3<br>≤43	AGC 51%<br>SLC25A45/A47/A48 18%<br>APC 17%<br>2 other ≤ 7%<br>(28 total)	AGC (15)<br>3 other (1)<br>(18 total)	111±6*<br>≤120	46±4<br>≤42	AGC 83%<br>5 other ≤ 6%<br>(31 total)	AGC (4)
**2 OLD**	AGC (11)<br>APC (3)<br>ALC (3)<br>YPR011C (1)<br>(17 total)	98±7*<br>101±4<br>96±1<br>98	42±4<br>37±3<br>36±0<br>38	AGC 67%<br>APC 17%<br>2 other ≤ 10%<br>(28 total)	AGC (15)<br>TPC (2)<br>SLC25A16 (1)<br>(18 total)	111±6*<br>104±2<br>107	46±4<br>40±0<br>40	AGC 88%<br>4 other ≤ 5%<br>(32 total)	AGC (4)
**3**	AGC (4.5)<br>MFT/FAD (3)<br>5 other (≤1)<br>(12 total)	76±4<br>78±1<br>≤81	61±3<br>70±3<br>≤68	AGC 33%<br>MFT/FAD 31%<br>5 other ≤ 13%<br>(43 total)	MFT/FAD (4)<br>6 other ≤ 2<br>(12 total)	78±2<br>≤81	69±4<br>≤68	MFT/FAD 38%<br>AGC 23%<br>7 other ≤ 11%<br>(37 total)	-
**3 OLD**	AGC (4.5)<br>MFT/FAD (3)<br>5 other (≤1)<br>(12 total)	76±4<br>78±1<br>≤81	61±3<br>70±3<br>≤68	AGC 33%<br>MFT/FAD 31%<br>5 other ≤ 13%<br>(43 total)	MFT/FAD (4)<br>6 other ≤ 2<br>(12 total)	78±2<br>≤81	69±4<br>≤68	MFT/FAD 38%<br>AGC 23%<br>7 other ≤ 11%<br>(37 total)	-
**4**	AGC (12)<br>(12 total)	68±6	48±4	AGC 100%<br>(13 total)	AGC (9)<br>APC (3)<br>(12 total)	69±4<br>68±3	47±2<br>36±3	AGC 79%<br>APC 21%<br>(25 total)	AGC (4)
**4 OLD**	AGC (12)<br>(12 total)	68±6	48±4	AGC 100%<br>(13 total)	AGC (9)<br>APC (3)<br>(12 total)	69±4<br>68±3	47±2<br>36±3	AGC 79%<br>APC 21%<br>(25 total)	AGC (4)
**5**	AGC (14)<br>ALC (2)<br>(16 total)	103±9<br>87±3	45±4<br>38±1	AGC 89%<br>ALC 11%<br>(21 total)	AGC (14)<br>other 2 (1)<br>(16 total)	108±8<br>≤90	47±5<br>≤38	AGC 92%<br>3 other ≤ 6%<br>(27 total)	AGC (4)
**5 OLD**	AGC (14)<br>ALC (2)<br>(16 total)	103±9<br>87±3	45±4<br>38±1	AGC 89%<br>ALC 11%<br>(21 total)	AGC (14)<br>other 2 (1)<br>(16 total)	108±8<br>≤90	47±5<br>≤38	AGC 92%<br>3 other ≤ 6%<br>(27 total)	AGC (4)
**6**	CAC (2)	85±3	35±1	CAC 24%	APCX (4)	95±7	33±1	APCX 40%	-

	APCX (2)<br>UCP4 (2)<br>3 other (1)<br>(9 total)	79±6<br>72±15<br>≤97	44±6<br>32±1<br>≤35	UCP4 20%<br>5 other ≤ 17%<br>(11 total)	5 other (≤1)<br>(9 total)	≤100	≤36	7 other ≤ 14%<br>(11 total)	
**6 OLD**	CAC (2)<br>UCP4 (2)<br>4 other (1)<br>(8 total)	85±3<br>72±15<br>≤97	35±1<br>32±1<br>≤35	CAC 26%<br>UCP4 22%<br>4 other ≤ 15%<br>(9 total)	A40 (2)<br>5 other (1)<br>(7 total)	96±5<br>≤100	33±2<br>≤36	A40 31%<br>5 other ≤ 16%<br>(8 total)	-
**7**	AGC (5)<br>SLC25A39-A40<br>(4)<br>(9 total)	114±10<br>124±4	37±5<br>38±3	AGC 49%<br>SLC25A39-A40 51%<br>(12 total)	SLC25A39-A40 (5)<br>AGC (3)<br>SAMC (1)<br>(9 total)	123±4<br>121±4<br>123	36±3<br>42±4<br>38	SLC25A39-A40<br>50%<br>AGC 39%<br>SAMC 11%<br>(12 total)	SLC25A39-<br>A40 (1)<br>AGC (1)
**7 OLD**	AGC (5)<br>SLC25A39-A40<br>(4)<br>(9 total)	114±10<br>124±4	37±5<br>38±3	AGC 49%<br>SLC25A39-A40 51%<br>(12 total)	SLC25A39-A40 (5)<br>AGC (3)<br>SAMC (1)<br>(9 total)	123±4<br>121±4<br>123	36±3<br>42±4<br>38	SLC25A39-A40<br>50%<br>AGC 39%<br>SAMC 11%<br>(12 total)	SLC25A39-<br>A40 (1)<br>AGC (1)
**8**	AGC (7)<br>4 other (1)<br>(11 total)	63±7<br>≤67	38±5<br>≤40	AGC 81%<br>5 other ≤ 5%<br>(24 total)	AGC (5.5)<br>CAC (2.5)<br>4 other (≤1)<br>(11 total)	69±3<br>64±6<br>≤65	44±4<br>43±9<br>≤45	AGC 58%<br>CAC 16%<br>6 other ≤ 8%<br>(25 total)	AGC (3)
**8 OLD**	AGC (7)<br>4 other (1)<br>(11 total)	63±7<br>≤67	38±5<br>≤40	AGC 81%<br>4 other ≤ 8%<br>(23 total)	AGC (5.5)<br>CAC (2.5)<br>3 other (≤1)<br>(11 total)	69±3<br>64±6<br>≤65	44±4<br>43±9<br>≤45	AGC 63%<br>CAC 16%<br>5 other ≤ 8%<br>(24 total)	AGC (3)
**9**	AGC (12)<br>APC (3)<br>3 other (1)<br>(18 total)	79±8<br>81±6<br>≤78	33±2<br>36±4<br>≤35	AGC 65%<br>APC 21%<br>5 other ≤ 6%<br>(24 total)	AGC (9)<br>APC (6)<br>A36 (3)<br>(17 total)	93±6<br>89±3<br>85±3	35±3<br>34±4<br>31±1	AGC 52%<br>APC 39%<br>2 other ≤ 7%<br>(32 total)	AGC (4)
**9 OLD**	AGC (12)<br>APC (3)<br>3 other (1)<br>(18 total)	79±8<br>81±6<br>≤78	33±2<br>36±4<br>≤35	AGC 65%<br>APC 21%<br>4 other ≤ 6%<br>(23 total)	AGC (9)<br>APC (6)<br>A36 (3)<br>(17 total)	93±6<br>89±3<br>85±3	35±3<br>34±4<br>31±1	AGC 52%<br>APC 40%<br>PNC 8%<br>(31 total)	AGC (4)

f. MC subfamilies outside the main clusters

SFC subfamily exons	most similar ES				most similar FLS				final prediction
	**top ES hits (number)**	**AAS**	**ASI (%)**	**top 5% ES hits in % of summed AS (number)**	**top FLS hits (number)**	**AAS**	**ASI (%)**	**top 5% FLS hits in % of summed AS (number)**	**subfamily hit (confidence score)**
**1**	-	-	-	-	CIC (3)<br>(3 total)	240±23	31±1	CIC 100%<br>(3 total)	(CIC)
**1 OLD**	-	-	-	-	-	-	-	-	-
**2**	-	-	-	-	AGC (1)<br>(1 total)	288	31	-	-
**2 OLD**	-	-	-	-	AGC (1)<br>(1 total)	288	31	-	-

CIC subfamily exons	most similar ES				most similar FLS				final prediction
	**top ES hits (number)**	**AAS**	**ASI (%)**	**top 5% ES hits in % of summed AS (number)**	**top FLS hits (number)**	**AAS**	**ASI (%)**	**top 5% FLS hits in % of summed AS (number)**	**subfamily hit (confidence score)**
**1**	CoC (1)<br>AT4G15010 (1)<br>(2 total)	53<br>49	31<br>32	-	GC (1)<br>PiC (1)<br>(2 total)	54<br>52	31<br>32	-	-
**1 OLD**	CoC (1)<br>(1 total)	53	31	-	PiC (1)<br>A28<br>(2 total)	54<br>45	31<br>38	-	-
**2**	ALC (3)<br>GC (2)<br>ODC (1)<br>(6 total)	80±3<br>73±2<br>76	36±0<br>36±3<br>41	ALC 52%<br>ODC 29%<br>2 other ≤ 13%<br>(9 total)	GC (4)<br>ODC (1)<br>AGC (1)<br>(6 total)	90±2<br>87<br>74	39±2<br>33<br>35	GC 61%<br>ODC 26%<br>2 other ≤ 9%<br>(13 total)	ALC (1)<br>GC (2)
**2 OLD**	ALC (3)<br>YMC/BOU (1)<br>(4 total)	80±3<br>69	36±039	ALC 73%<br>YMC/BOU 18%<br>MFRN 9%<br>(5 total)	MFT/FAD (2)<br>4 other (1)<br>(5 total)	74±0<br>74-89	36±0<br>33-36	MFT/FAD 31%<br>ALC 23%<br>3 other ≤ 19%<br>(8 total	ALC (2)
**3**	ORC (3)	88±6	44±3	ORC 67%	CoC (2)	90±3	49±3	CoC 39%	ORC (1)

	PNC (2)<br>MFT/FAD (1)<br>(6 total)	88±3<br>85	43±4<br>37	PNC 25%<br>MFT/FAD 8%<br>(9 total)	CAC (2)<br>ORC (1)<br>PNC (1)<br>(6 total)	87±2<br>97<br>90	48±2<br>46<br>46	A18/CAC 27%<br>ORC 18%<br>3 other ≤ 8%<br>(13 total)	
**3 OLD**	ORC (4)<br>MFT/FAD (1)<br>AGC (1)<br>(6 total)	87±6<br>85<br>84	43±4<br>37<br>42	ORC 65%<br>AGC 26%<br>MFT/FAD 8%<br>(12 total)	CoC (3)<br>ORC (2)<br>AGC (1)<br>(6 total)	90±2<br>90±8<br>84	48±3<br>43±4<br>46	CoC 46%<br>AGC 24%<br>ORC 23%<br>2 other ≤ 4%<br>(12 total)	ORC (2)
**4**	SFC (6)<br>(6 total)	83±6	37±1	SFC 100%<br>(6 total)	SFC (6)<br>(6 total)	97±4	38±3	SFC 100%<br>(6 total)	SFC (5)
**4 OLD**	SFC (6)<br>(6 total)	83±6	37±1	SFC 100%<br>(6 total)	SFC (6)<br>(6 total)	97±4	38±3	SFC 100%<br>(6 total)	SFC (5)
**5**	ALC (5)<br>AAC (1)<br>(6 total)	66±2<br>58	34±2<br>48	ALC 85%<br>AAC 15%<br>(8 total)	ALC (2)<br>MFT/FAD (2)<br>UCP5-6 (2)<br>(6 total)	63±4<br>59±2<br>58±4	37±2<br>35±0<br>33±2	MFT/FAD 41%<br>ALC 35%<br>3 other ≤ 10%<br>(13 total)	ALC (2)
**5 OLD**	ALC (5)<br>AAC (1)<br>(6 total)	66±2<br>58	34±2<br>48	ALC 85%<br>AAC 15%<br>(8 total)	MFT/FAD (3)<br>ALC (2)<br>AAC (1)<br>(6 total)	58±1<br>63±4<br>58±4	35±0<br>37±2<br>33±2	MFT/FAD 49%<br>ALC 36%<br>AAC 15%<br>(9 total)	ALC (2)
**6**	SFC (4)<br>DIC (1)<br>TPC (1)<br>(6 total)	75±1<br>75<br>68	35±1<br>31<br>38	SFC 79%<br>TPC 15%<br>DIC 6%<br>(8 total)	APCX (3)<br>SFC (2)<br>TPC (1)<br>(6 total)	78±1<br>80±5<br>68	45±1<br>36±2<br>38	APCX 58%<br>SFC 34%<br>TPC 7%<br>(7 total)	SFC (2)<br>APCX (1)
**6 OLD**	SFC (4)<br>DIC (1)<br>TPC (1)<br>(6 total)	75±1<br>75<br>68	35±1<br>31<br>38	SFC 79%<br>TPC 15%<br>DIC 6%<br>(8 total)	SFC (4)<br>DIC (1)<br>TPC (1)<br>(6 total)	77±4<br>75<br>68	35±1<br>31<br>38	SFC 79%<br>TPC 15%<br>DIC 6%<br>(8 total)	SFC (4)
**7**	CoC (5)<br>MME (1)<br>(6 total)	85±4<br>86	44±3<br>47	CoC 52%<br>MME 25%<br>3 other ≤ 11%<br>(13 total)	CoC (3)<br>GC (2)<br>MME (1)<br>(6 total)	93±4<br>89±4<br>86	45±3<br>50±0<br>47	CoC 41%<br>GC 38%<br>3 other ≤ 8%<br>(9 total)	CoC (2)
**7 OLD**	CoC (6)<br>(6 total)	85±4	44±3	CoC 77%<br>3 other ≤ 11%<br>(12 total)	CoC (3)<br>3 other (1)<br>(6 total)	93±4<br>82-84	45±3<br>32-40	CoC 49%<br>6 other ≤ 12%<br>(14 total)	CoC (2)

**8**	UCP2-3 (3)<br>AAC (2)<br>3 other (1)<br>(8 total)	60±1<br>72±7<br>59-75	41±5<br>46±2<br>44-56	UCP2-3 29%<br>AAC 27%<br>GC 20%<br>2 other ≤ 12%<br>(10 total)	GC (4)<br>AAC (3)<br>A28 (1)<br>(8 total)	71±9<br>68±5<br>69	52±3<br>48±3<br>40	GC 46%<br>AAC 34%<br>4 other ≤ 6%<br>(13 total)	-
**8 OLD**	AAC (3)<br>UCP2-3 (3)<br>YMC/BOU (2)<br>(8 total)	68±8<br>60±1<br>61±5	49±5<br>41±5<br>48±8	AAC 40%<br>A8/UCP3 35%<br>YMC/BOU 24%<br>(8 total)	AAC (4)<br>4 other (1)<br>(8 total)	66±6<br>62-72	49±3<br>40-48	AAC 41%<br>MFT/FAD 20%<br>DIC 18%<br>4 other ≤ 7%<br>(12 total)	(AAC)
**9**	MFT/FAD (8.5)<br>SFC (1.5)<br>(10 total)	70±3<br>66±1	40±3<br>36±1	MFT/FAD 87%<br>2 other ≤ 8%<br>(18 total)	MFT/FAD (8)<br>YPR011C (1)<br>APC (1)<br>(10 total)	72±4<br>77<br>72	41±2<br>35<br>33	MFT/FAD 86%<br>3 other ≤ 7%<br>(19 total)	MFT/FAD (4)
**9 OLD**	MFT/FAD (8.5)<br>SFC (1.5)<br>(10 total)	70±3<br>66±1	40±3<br>36±1	MFT/FAD 87%<br>2 other ≤ 8%<br>(18 total)	MFT/FAD (8)<br>YPR011C (1)<br>APC (1)<br>(10 total)	72±4<br>77<br>72	41±2<br>35<br>33	MFT/FAD 86%<br>3 other ≤ 7%<br>(19 total)	MFT/FAD (4)

**ODC subfamily exons**	**most similar ES**				**most similar FLS**				**final prediction**
	**top ES hits (number)**	**AAS**	**ASI (%)**	**top 5% ES hits in % of summed AS (number)**	**top FLS hits (number)**	**AAS**	**ASI (%)**	**top 5% FLS hits in % of summed AS (number)**	**subfamily hit (confidence score)**
**1**	NDT (5)<br>4 other (1)<br>(9 total)	48±7<br>39-74	41±4<br>36-46	NDT 52%<br>4 other ≤ 16%<br>(9 total)	NDT (3)<br>AAC (2)<br>other 6 (1)<br>(11 total)	46±7<br>42±6<br>39-71	42±3<br>36±6<br>37-46	NDT 22%<br>11 other ≤ 12%<br>(16 total)	NDT (2)
**1 OLD**	NDT (5)<br>4 other (1)<br>(9 total)	48±7<br>39-74	41±4<br>33-39	NDT 53%<br>4 other ≤ 17%<br>(9 total)	NDT (3)<br>AAC (2)<br>other 6 (1)<br>(11 total)	46±7<br>42±6<br>39-71	42±3<br>36±6<br>37-39	NDT 28%<br>ORC 17%<br>7 other ≤ 13%<br>(14 total)	NDT (2)
**2**	SFC (5)<br>NDT (1)<br>(6 total)	51±0<br>44	63±0<br>56	SFC 43%<br>GC 42%<br>NDT 15%<br>(12 total)	MFRN (5) #<br>SAMCX (1)<br>(6 total)	53±0<br>42	69±0<br>63	MFRN 87% #<br>3 other ≤ 5%<br>(8 total)	MFRN (2) #<br>SFC (1)

**2 OLD**	SFC (5)<br>NDT (1)<br>(6 total)	51±0<br>44	63±0<br>56	SFC 85%<br>NDT 15%<br>(12 total)	MFRN (5) #<br>MFT/FAD (1)<br>(6 total)	53±0<br>42	69±0<br>44	MFRN 87% #<br>4 other ≤ 7%<br>(11 total)	SFC (2)<br>MFRN (2) #
**3**	AGC (1)<br>SLC25A43 (1)<br>4 other (1)<br>(6 total)	75<br>71<br>55-69	43<br>41<br>36-42	AGC 26%<br>6 other ≤ 18%<br>(7 total)	GC (4)<br>other 4 (1)<br>(8 total)	71±12<br>60-71	48±2<br>32-39	GC 53%<br>6 other ≤ 13%<br>(12 total)	GC (1)
**3 OLD**	AGC (2)<br>ALC (2)<br>2 other (1)<br>(6 total)	73±3<br>67±2<br>50-66	44±1<br>37±1<br>39	AGC 37%<br>ALC 34%<br>3 other ≤ 13%<br>(8 total)	AGC (2)<br>NDT (1.5)<br>5 other (≤ 1)<br>(8 total)	76±1<br>53±7	46±1<br>34±2	AGC 30%<br>NDT 15%<br>8 other ≤ 13%<br>(16 total)	-
**4**	AGC (4)<br>ALC (1)<br>(5 total)	56±3<br>60	46±3<br>36	AGC 79%<br>ALC 21%<br>(8 total)	AGC (4)<br>CAC (1)<br>(5 total)	61±3<br>64	46±3<br>59	CAC 51% #<br>AGC 49%<br>(8 total)	AGC (3)<br>CAC (1) #
**4 OLD**	AGC (4)<br>ALC (1)<br>(5 total)	56±3<br>60	46±3<br>36	AGC 79%<br>ALC 21(1)<br>(8 total)	AGC (5)<br>(5 total)	61±3	45±3	AGC 100%<br>(6 total)	AGC (4)
**5**	AT4G11440 (3)<br>APC (3)<br>3 other (1)<br>(9 total)	44±4<br>39±2<br>37-48	43±4<br>40±7<br>35-69	AT4G11440 35%<br>APC 21%<br>5 other ≤ 13%<br>(9 total)	CoC (5)<br>A23-A25(3)<br>MFRN (1)<br>(9 total)	48±4<br>43±4<br>39	41±7<br>40±0<br>35	CoC 63%<br>APC 25%<br>2 other ≤ 9%	CoC (2)
**5 OLD**	CoC (2)<br>APC (2)<br>3 other (1)<br>(7 total)	38±1<br>36±1<br>35-48	45±0<br>32±2<br>35-43	CoC 28%<br>APC 20%<br>4 other ≤ 18%<br>(8 total)	CoC (6)<br>APC (2)<br>MFRN (1)<br>(9 total)	47±5<br>44±5<br>39	42±6<br>40±0<br>35	CoC 65%<br>APC 26%<br>MFRN 9%<br>(14 total)	CoC (2)
**6**	GC (4)<br>SFC (3)<br>MCART (1)<br>(8 total)	87±4<br>86±5<br>68	46±2<br>42±0<br>30	GC 72%<br>SFC 23%<br>MCART 5%<br>(14 total)	SFC (5)<br>GC (2)<br>MCART (1)<br>(8 total)	90±4<br>96±2<br>68	46±2<br>47±3<br>30	SFC 51%<br>GC 39%<br>3 other ≤ 5%<br>(17 total)	SFC (2)<br>GC (1)
**6 OLD**	SFC (7)<br>MFT/FAD (1)<br>(8 total)	82±8<br>60	41±3<br>30	SFC 91%<br>2 other ≤ 5%<br>(10 total)	SFC (7)<br>MFT/FAD (1)<br>(8 total)	89±5<br>66	44±3<br>33	SFC 95%<br>MFT/FAD 5%<br>(12 total)	SFC (4)
**7**	GlyC (5)<br>other 2 (1)<br>(7 total)	100±7<br>91-97	33±1<br>31-33	GlyC 68%<br>3 other ≤ 14%<br>(11 total)	GlyC (3)<br>GC (2)<br>other 3 (1)<br>(8 total)	98±4<br>92±3<br>86-97	34±2<br>33±2<br>31-33	GlyC 45%<br>GC 16%<br>SLC25A39-A40 16%<br>3 other ≤ 13%	GlyC (2)

								(13 total)	
**7 OLD**	GlyC (5)<br>other 2 (1)<br>(7 total)	100±7<br>91-97	33±1<br>31-33	GlyC 68%<br>3 other ≤ 14%<br>(11 total)	GlyC (4)<br>4 other (1)<br>(8 total)	95±7<br>83-97	34±2<br>31-33	GlyC 51%<br>4 other ≤ 16%<br>(12 total)	GlyC (3)
**8**	OGC (4)<br>SFC (2)<br>UCP2-3 (2)<br>SLC25A39-A40 (1)<br>(10 total)	98±6<br>115±6<br>98±7<br>120	31±1<br>32±1<br>34±3<br>34	OGC 27%<br>SFC 24%<br>UCP2-3 21%<br>3 other ≤ 13%<br>(13 total)	PNC (5)<br>OGC (2)<br>UCP2-3 (2)<br>3 other (1)<br>(12 total)	118±4<br>118±7<br>112±8<br>121-145	34±3<br>33±0<br>30±0<br>30-36	PNC 25%<br>8 other ≤ 15%<br>(19 total)	-
**8 OLD**	OGC (4)<br>SFC (3)<br>UCP2-3 (2)<br>SLC25A39-A40 (1)<br>(10 total)	98±6<br>115±6<br>98±7<br>120	31±1<br>32±1<br>34±3<br>34	OGC 42%<br>SFC 24%<br>UCP2-3 20%<br>SLC25A39-A40 13%<br>(12 total)	SLC25A39-A40 (2)<br>OGC (2)<br>UCP2-3 (2)<br>CoC (2)<br>4 other (1)<br>(12 total)	121±1<br>118±7<br>112±8<br>111±2<br>107-145	32±3<br>33±0<br>33±3<br>34±1<br>31-36	OGC 21%<br>SLC25A39-A40 17%<br>CoC 16%<br>6 other ≤ 12%<br>(15 total)	-
**9**	SFC (4)<br>other 2 (2)<br>(6 total)	62±3<br>57-67	64±3<br>56-67	SFC 59%<br>UCP5-6 32%<br>DIC 9%<br>(16 total)	SFC (4)<br>UCP5-6 (2)<br>(6 total)	62±3<br>61±4	64±3<br>70±3	SFC 67%<br>UCP5-6 33%<br>(13 total)	SFC (4)
**9 OLD**	SFC (5)<br>DIC (1)<br>(6 total)	60±5<br>67	64±5<br>56	SFC 72%<br>DIC 18%<br>2 other ≤ 5%<br>(9 total)	SFC (5)<br>UCP2-3 (1)<br>(6 total)	61±4<br>61	64±4<br>61	SFC 83%<br>UCP2-3 17%<br>(10 total)	SFC (4)
**10**	TPC (2)<br>AAC (2)<br>3 other (≤1)<br>(6 total)	43±2<br>36±7<br>32-39	40±3<br>38±6<br>35-37	AAC 31%<br>TPC 29%<br>4 other ≤ 17%<br>(8 total)	TPC (2)<br>4 other (1)<br>(6 total)	43±2<br>32-43	40±3<br>32-40	TPC 25%<br>MFT/FAD 20%<br>5 other ≤ 16%<br>(10 total)	-
**10 OLD**	TPC (2)<br>AAC (2)<br>3 other (≤1)<br>(6 total)	43±2<br>36±7<br>32-36	40±3<br>38±6<br>35-37	AAC 32%<br>TPC 29%<br>4 other ≤ 17%<br>(9 total)	TPC (2)<br>A4-A6<br>2 other (1)<br>(6 total)	43±2<br>36±7<br>36	40±3<br>38±6<br>37-40	TPC 26%<br>AAC 22%<br>MFT/FAD 20%<br>3 other ≤ 16%<br>(10 total)	-

**SLC25A39-A40 subfamily exons**	**most similar ES**	**most similar FLS**	**final prediction**

	top ES hits (number)	AAS	ASI (%)	top 5% ES hits in % of summed AS (number)	top FLS hits (number)	AAS	ASI (%)	top 5% FLS hits in % of summed AS (number)	subfamily hit (confidence score)
**1**	APCX (3)<br>SLC25A43 (2)<br>6 other (1)<br>(11 total)	45±3<br>44±0<br>28-58	32±1<br>32±1<br>30-48	APCX 28%<br>MFT/FAD 18%<br>6 other ≤ 12%<br>(14 total)	CAC (2)<br>SLC25A43 (2)<br>SAMC (2)<br>NDT (2)<br>5 other (1)<br>(13 total)	48±8<br>47±3<br>39±7<br>31±3<br>40-50	33±2<br>32±1<br>33±2<br>31±0<br>31-34	SLC25A43 17%<br>CAC 14%<br>10 other ≤ 11%<br>(16 total)	-
**1 OLD**	UCP2-3 (2)<br>peANT (2)<br>DIC (2)<br>SFC (2)<br>3 other (1)	39±6<br>36±8<br>32±2<br>30±5<br>28-37	31±0<br>31±0<br>43±6<br>31±0<br>30-38	UCP2-3 21%<br>peANT 20%<br>5 other ≤ 17%<br>(13 total)	SAMC (3)<br>APC (2)<br>NDT (2)<br>4 other (1)<br>(11 total)	39±6<br>44±5<br>31±3<br>26-44	34±15<br>31±0<br>31±0<br>31-34	SAMC 21%<br>APC 20%<br>NDT 14%<br>5 other ≤ 10%<br>(12 total)	-
**2**	SLC25A48 (2.5)<br>7 other (≤ 1)<br>(9 total)	56±3<br>52-64	51±2<br>45-55	SLC25A48 28%<br>MME 16%<br>ALC 15%<br>SFC 14%<br>4 other ≤ 9%<br>(19 total)	MME (2)<br>7 other (1)<br>(9 total)	64±5<br>54-56	53±3<br>55-60	TPC 35%<br>MME 19%<br>5 other ≤ 16%<br>(19 total)	-
**2 OLD**	UCP4 (2)<br>ALC (2)<br>TPC (2)<br>SFC (2)<br>YMC/BOU (1)<br>(9 total)	60±0<br>56±3<br>55±2<br>55±3<br>57	55±0<br>58±3<br>58±3<br>55±0<br>45	ALC 27%<br>SFC 22%<br>TPC 22%<br>3 other ≤ 12%<br>(13 total)	TPC (3.5)<br>SFC (3)<br>ALC (1.5)<br>NDT (1)<br>(9 total)	56±2<br>55±2<br>55±2<br>63	53±2<br>53±2<br>55±0<br>55	TPC 37%<br>SFC 24%<br>3 other ≤ 18%<br>(22 total)	-
**3<br>SLC25A39 only**	AT4G15010 (1)<br>A12 (1)<br>YPR011C (1)<br>(3 total)	38<br>33<br>27	35<br>47<br>33	-	A12 (1)<br>AT4G15010 (1)<br>2 other (≤ 0.5)	40<br>38	47<br>35	-	-
**3 OLD<br>SLC25A39 only**	A12 (1)<br>YPR011C (1)<br>(2 total)	33<br>27	47<br>33	-	A12 (1)<br>YPR011C (1)<br>APC (1)<br>(2 total)	40<br>27<br>23	47<br>33<br>35	-	-
**4**	-	-	-	-	-	-	-	-	-
**4 OLD**	-	-	-	-	-	-	-	-	-

**5**	OGC (3)<br>UCP4 (3)<br>ODC (2)<br>peCFNC (2)<br>TPC (1)<br>(11 total)	71±3<br>68±4<br>73±0<br>70±4<br>70	53±2<br>57±4<br>50±2<br>54±7<br>61	OGC 33%<br>ODC 19%<br>UCP4 18%<br>4 other ≤ 12%<br>(19 total)	peCFNC (5)<br>UCP5-6 (2)<br>4 other (1)<br>(11 total)	73±5<br>73±2<br>67-73	59±6<br>61±0<br>52-61	UCP5-6 31%<br>peCFNC 31%<br>OGC 14%<br>5 other ≤ 10%<br>(25 total)	-
**5 OLD**	UCP4 (5)<br>OGC (3)<br>peCFNC (2)<br>TPC (1)<br>(11 total)	68±3<br>71±3<br>70±4<br>70	57±4<br>53±2<br>54±7<br>61	UCP4 42%<br>OGC 36%<br>2 other ≤ 12%<br>(19 total)	peCFNC (6)<br>5 other (1)<br>(11 total)	73±4<br>64-73	59±5<br>52-67	peCFNC 50%<br>OGC 16%<br>5 other ≤ 12%<br>(23 total)	-
**6**	AGC (3)<br>MFRN (3)<br>5 other (1)<br>(11 total)	66±3<br>64±6<br>62-72	33±2<br>32±1<br>31-36	MFRN 34%<br>AGC 20%<br>6 other ≤ 11%<br>(15 total)	CAC (4)<br>NDT (3)<br>2 other (1)<br>(9 total)	75±4<br>85±2<br>69-76	35±3<br>33±3<br>31-33	CAC 43%<br>NDT 37%<br>2 other ≤ 11%<br>(10 total)	-
**6 OLD**	AGC (4)<br>MFRN (3)<br>4 other (1)<br>(11 total)	63±5<br>64±6<br>60-69	33±2<br>32±1<br>31-36	MFRN 35%<br>AGC 30%<br>4 other ≤ 11%<br>(14 total)	NDT (4)<br>AGC (2)<br>MFRN (2)<br>3 other (1)<br>(11 total)	81±8<br>60±5<br>59±2<br>69-76	33±3<br>38±5<br>33±2<br>31-33	NDT 42%<br>AGC 16%<br>MFRN 14%<br>5 other ≤ 9%<br>(16 total)	-
**7**	YMC/BOU (4)<br>3 other (1)<br>(7 total)	127±9<br>65-125	33±1<br>31-42	YMC/BOU 56%<br>MFT/FAD 16%<br>3 other ≤ 12%<br>(9 total)	AGC (7)<br>YMC/BOU (2)<br>AACX (1)<br>(10 total)	132±9<br>133±8<br>65	33±2<br>33±2<br>42	AGC 54%<br>YMC/BOU 19%<br>7 other ≤ 9%<br>(26 total)	YMC/BOU (2)<br>AGC (2)
**7 OLD**	YMC/BOU (4)<br>3 other (≤ 1)<br>(7 total)	127±9<br>54-125	33±1<br>31-33	YMC/BOU 58%<br>MFT/FAD 16%<br>5 other ≤ 12%<br>(10 total)	AGC (7)<br>YMC/BOU (2)<br>peANT (1)<br>(10 total)	132±9<br>133±8<br>54	33±2<br>33±2<br>33	AGC 63%<br>YMC/BOU 19%<br>6 other ≤ 5%<br>(23 total)	YMC/BOU (2)<br>AGC (2)
**8**	MFT/FAD (2)<br>ALC (2)<br>BTL (2)<br>4 other (1)<br>(10 total)	54±6<br>53±5<br>50±6<br>47-104	31±0<br>31±0<br>31±0<br>33-58	ALC 22%<br>MFT/FAD 18%<br>6 other ≤ 16%<br>(14 total)	GC (2)<br>AT4G11440 (2)<br>4 other (1)<br>(8 total)	80±29<br>57±0<br>54-125	46±15<br>32±1<br>31-36	AGC 22%<br>AT4G11440 20%<br>GC 18%<br>4 other ≤ 12%<br>(10 total)	-
**8 OLD**	MFT/FAD (2)<br>ALC (2)<br>4 other (1)<br>(8 total)	54±6<br>53±5<br>47-104	31±0<br>31±0<br>33-58	ALC 25%<br>MFT/FAD 20%<br>YPR011C 19%<br>GlyC 18%	ALC (3)<br>5 other (1)<br>(8 total)	52±4<br>49-125	32±1<br>31-58	ALC 28%<br>AGC 22%<br>GlyC 22%<br>3 other ≤ 13%	-

				2 other ≤ 10%<br>(9 total)				(10 total)	
**9**	SAMCX (3.5)<br>GlyC (3)<br>7 other (≤ 1.5)<br>(14 total)	60±2<br><br><br>54±5<br>50-68	44±1<br><br><br>37±2<br>35-48	SAMCX 29%<br>GlyC 18%<br>10 other ≤ 8%<br>(26 total)	SAMCX (2)<br>SAMC (2)<br>SLC25A44 (1.3)<br>10 other (≤ 1)<br>(14 total)	60±2<br><br>59±5<br>58±4<br>55-71	45±2<br><br>45±2<br>45±2<br>36-52	SAMC 16%<br>AT4G11440 14%<br>14 other ≤ 9%<br>(31 total)	-
**9 OLD**	MFRN (3)<br>NDT (3)<br>GlyC (3)<br>TPC (2)<br>3 other (1)<br>(14 total)	58±7<br>55±4<br>54±5<br>61±2<br>52-61	36±5<br>37±5<br>37±2<br>39±1<br>32-48	GlyC 20%<br>MFRN 19%<br>NDT 18%<br>6 other ≤ 12%<br>(22 total)	SAMC (3)<br>SLC25A44 (1.5)<br>MFRN (1.5)<br>8 other (1)<br>(14 total)	62±6<br>58±4<br>58±4<br>52-63	46±2<br>45±2<br>43±0<br>32-52	SAMC 26%<br>MFRN 15%<br>10 other ≤ 11%<br>(23 total)	-
**10**	NDT (5)<br>5 other (1)<br>(10 total)	57±6<br>49-59	35±4<br>37-44	NDT 39%<br>peANT 15%<br>5 other ≤ 11%<br>(12 total)	NDT (2)<br>SAMC (2)<br>7 other (≤ 1.5)<br>(11 total)	64±1<br>54±4<br>49-63	44±0<br>36±4<br>33-52	SAMC 28%<br>NDT 26%<br>8 other ≤ 10%<br>(19 total)	-
**10 OLD**	NDT (6)<br>4 other (1)<br>(10 total)	54±7<br>44-59	35±4<br>30-41	NDT 44%<br>peANT 16%<br>5 other ≤ 11%<br>(14 total)	NDT (2)<br>TPC (2)<br>SAMC (2)<br>6 other (≤ 1)<br>(11 total)	64±1<br>63±3<br>54±4<br>49-63	44±0<br>36±2<br>36±4<br>33-46	SAMC 26%<br>NDT 24%<br>8 other ≤ 13%<br>(19 total)	NDT (1)
**11**	MFRN (11)<br>MFT/FAD (1)<br>(12 total)	81±8<br>68	44±3<br>33	MFRN 93%<br>MFT/FAD 7%<br>(15 total)	MFRN (11)<br>ODC (1)<br>(12 total)	81±8<br>70	44±3<br>37	MFRN 93%<br>ODC 7%<br>(16 total)	MFRN (4)
**11OLD**	MFRN (11)<br>MFT/FAD (1)<br>(12 total)	81±8<br>68	44±3<br>33	MFRN 93%<br>MFT/FAD 7%<br>(15 total)	MFRN (11)<br>MFT/FAD (1)<br>(12 total)	81±8<br>68	44±3<br>33	MFRN 93%<br>MFT/FAD 7%<br>(16 total)	MFRN (4)

pSLC25A39-A40 subfamily exons	most similar ES				most similar FLS				final prediction
	top ES hits (number)	AAS	ASI (%)	top 5% ES hits in % of summed AS (number)	top FLS hits (number)	AAS	ASI (%)	top 5% FLS hits in % of summed AS (number)	subfamily hit (confidence score)
**1**	MFRN (1)<br>APCX (1)<br>UCP5-6 (1)<br>(3 total)	60<br>58<br>57	36<br>31<br>31	MFRN 34%<br>APCX 33%<br>2 other ≤ 16%<br>(5 total)	APC (1)<br>SLC25A44 (1)<br>DIC (1)<br>(3 total)	71<br>66<br>62	36<br>31<br>36	DIC 31%<br>APC 18%<br>TPC 17%<br>3 other ≤ 11%<br>(7 total)	-
**1 OLD**	MFRN (1)<br>APC (1)<br>MFT/FAD (1)<br>(3 total)	60<br>56<br>53	36<br>33<br>36	MFRN 52%<br>3 other ≤ 17%<br>(5 total)	APC (1)<br>SLC25A44 (1)<br>DIC (1)<br>(3 total)	71<br>66<br>62	36<br>31<br>36	DIC 31%<br>APC 18%<br>3 other ≤ 17%<br>(6 total)	MFRN (1)
**2**	UCP2-3 (1)<br>ALC (1)<br>(2 total)	46<br>41	32<br>38	-	MTCH (1)<br>SLC25A43 (1)<br>ALC (1)<br>(3 total)	44<br>43<br>41	30<br>36<br>38	-	-
**2 OLD**	UCP2-3 (1)<br>ALC (1)<br>(2 total)	46<br>41	32<br>38	-	ALC (1)<br>UCP2-3 (1)<br>SAMC (1)<br>(3 total)	41<br>40<br>40	38<br>33<br>32	-	-
**3**	-	-	-	-	BT (1)<br>(1 total)	71	33	-	-
**3 OLD**	UCP2-3 (1)<br>(1 total)	45	31	-	NDT (1)<br>(1 total)	62	33	-	-
**4**	AAC (2)<br>CoC (1)<br>(3 total)	62±6<br>53	52±5<br>55	AAC 69%<br>CoC 31%<br>(6 total)	CoC (2)<br>AAC (1)<br>(3 total)	53±1<br>56	55±0<br>55	CoC 65%<br>AAC 35%<br>(4 total)	AAC (1)<br>CoC (1)
**4 OLD**	AAC (2)<br>CoC (1)<br>(3 total)	62±6<br>53	52±5<br>55	AAC 69%<br>CoC 31%<br>(6 total)	CoC (2)<br>AAC (1)<br>(3 total)	53±1<br>56	55±0<br>55	CoC 65%<br>AAC 35%<br>(4 total)	AAC (1)<br>CoC (1)
**5**	ALC (1)<br>BT (1)<br>(2 total)	104<br>104	35<br>30	-	ALC (1)<br>(1 total)	109	35	-	-

**5 OLD**	ALC (1)<br>CoC (1)<br>(2 total)	104<br>90	35<br>30	-	ALC (1)<br>(1 total)	109	35	-	-
**6**	YHM2 (1)<br>ORC (0.5)<br>YMC/BOU (0.5)<br>(2 total)	47<br>35<br>35	31<br>32<br>32	-	AT4G03115 (2)<br>ORC (0.3)<br>YMC/BOU (0.3)<br>MTCH (0.3)<br>(3 total)	53±6<br>35<br>35<br>35	36±1<br>32<br>32<br>32	AT4G03115 72%<br>3 other ≤ 8%<br>(5 total)	AT4G03115 (1)
**6 OLD**	ORC (0.5)<br>YMC/BOU (0.5)<br>(1 total)	35<br>35	32<br>32	-	OGC (1)<br>ORC (0.5)<br>YMC/BOU (0.5)<br>(2 total)	31<br>35<br>35	31<br>32<br>32	-	-
**7**	MFRN (3)<br>(3 total)	69±3*	39±2	A28/A39 85%<br>SAMC 15%<br>(6 total)	GlyC (2)<br>MFRN (1)<br>(3 total)	85±3*<br>79	38±2<br>30	GlyC 68%<br>MFRN 32%<br>(4 total)	GlyC (1)
**7 OLD**	MFRN (3)<br>(3 total)	69±3*	39±2	A28/A39 85%<br>SAMC 15%<br>(6 total)	GlyC (2)<br>MFRN (1)<br>(3 total)	85±3*<br>79	38±2<br>30	GlyC 68%<br>MFRN 32%<br>(4 total)	GlyC (1)
**8**	MFRN (3)<br>SAMC (1)<br>(4 total)	105±7<br>104	37±1<br>36	MFRN 75%<br>SAMC 25%<br>(4 total)	MFRN (2)<br>SAMC (1)<br>MME (1)<br>(4 total)	116±13<br>104<br>103	39±1<br>36<br>32	MFRN 53%<br>SAMC 24%<br>MME 24%<br>(5 total)	MFRN (3)
**8 OLD**	MFRN (3)<br>SAMC (1)<br>(4 total)	105±7<br>104	36	MFRN 75%<br>SAMC 25%<br>(4 total)	MFRN (2)<br>SAMC (1)<br>DIC (1)<br>(4 total)	116±13<br>104<br>96	39±1<br>36<br>34	MFRN 53%<br>SAMC 24%<br>2 other ≤ 11%<br>(6 total)	MFRN (3)
**9**	ODC (1)<br>APC (1)<br>(2 total)	29<br>22	35<br>30	-	AT4G15010 (1)<br>MFRN (1)<br>TPC (1)<br>(3 total)	36<br>33<br>25	30<br>35<br>35	-	-
**9 OLD**	APC (2)<br>(2 total)	24±2	30±0	-	MFRN (1)<br>TPC (1)<br>ORC (1)<br>(3 total)	33<br>25<br>30	35<br>35<br>35	MFRN 32%<br>TPC 24%<br>3 other ≤ 20%<br>(7 total)	-
**10**	APCX (1)	88	40	MFRN 41%	GlyC (2)	88±1	42±6	GlyC 39%	-

	APC (1)<br>MFRN (1)<br>ORC (1)<br>(4 total)	85<br>77<br>77	36<br>43<br>36	4 other ≤ 14%<br>(9 total)	MFRN (1.5)<br>ORC (0.5)<br>(4 total)	82±4<br>85	38±0<br>38	MFRN 36%<br>2 other ≤ 13%<br>(6 total)	
**10 OLD**	MFT/FAD (1)<br>APC (1)<br>MFRN (1)<br>ORC (1)<br>(4 total)	87<br>85<br>77<br>77	38<br>36<br>43<br>36	MFRN 41%<br>4 other ≤ 14%<br>(9 total)	GlyC (2)<br>MFRN (1.5)<br>ORC (0.5)<br>(4 total)	88±1<br>82±4<br>85	42±6<br>38±0<br>38	GlyC 39%<br>MFRN 36%<br>2 other ≤ 13%<br>(6 total)	-

**GlyC subfamily exons**	**most similar ES**				**most similar FLS**				**final prediction**
	**top ES hits (number)**	**AAS**	**ASI (%)**	**top 5% ES hits in % of summed AS (number)**	**top FLS hits (number)**	**AAS**	**ASI (%)**	**top 5% FLS hits in % of summed AS (number)**	**subfamily hit (confidence score)**
**1**	AACX (1)<br>DIC (1)<br>MFRN (1)<br>OAC (1)<br>(4 total)	39<br>21<br>15<br>12	30<br>31<br>60<br>80	AACX 45%<br>DIC 24%<br>2 other ≤ 17%<br>(4 total)	OAC (2)<br>3 other (1)<br>(5 total)	12±0<br>19-39	70±10<br>30-38	AACX 39%<br>OAC 24%<br>2 other ≤ 19%<br>(4 total)	-
**1 OLD**	DIC (1)<br>CoC (1)<br>MFRN (1)<br>SAMC (1)<br>(4 total)	21<br>19<br>15<br>12	31<br>35<br>60<br>40	DIC 31%<br>CoC 28%<br>2 other ≤ 22%<br>(4 total)	SLC25A39-A40 (1)<br>YMC/BOU (1)<br>3 other (1)<br>(5 total)	21<br>19<br>7-16	30<br>38<br>33-60	SLC25A39-A40 29%<br>YMC/BOU 26%<br>NDT 22%<br>2 other ≤ 13%<br>(9 total)	-
**2**	TPC (3)<br>ALC (2)<br>AGC (2)<br>CAC (1)<br>(8 total)	97±8<br>94±6<br>81±2<br>88	42±4<br>40±0<br>38±0<br>39	TPC 40%<br>ALC 26%<br>3 other ≤ 17%<br>(11 total)	TPC (2)<br>GC (2)<br>4 other (1)<br>(8 total)	99±2<br>88±2<br>88-102	42±3<br>38±0<br>37-41	TPC 35%<br>GC 14%<br>9 other ≤ 10%<br>(20 total)	(TPC)
**2 OLD**	TPC (3)<br>ALC (2)<br>AGC (2)<br>UCP3 (1)<br>(8 total)	97±8<br>94±6<br>81±2<br>77	42±4<br>40±0<br>38±0<br>39	TPC 45%<br>ALC 30%<br>3 other ≤ 17%<br>(15 total)	TPC (3)<br>AGC (2)<br>ALC (2)<br>CoC (1)<br>(8 total)	95±6<br>96±7<br>94±6<br>93	40±4<br>37±2<br>40±0<br>37	TPC 40%<br>AGC 19%<br>ALC 17%<br>4 other ≤ 12%<br>(16 total)	(TPC)

**3**	APC (3)<br>UCP4 (2)<br>OGC (2)<br>3 other (1)<br>(10 total)	73±3<br>69±3<br>68±0<br>63-71	42±5<br>40±1<br>45±2<br>38-43	APC 26%<br>UCP4 21%<br>OGC 18%<br>3 other ≤ 14%<br>(14 total)	APC (3)<br>OGC (3)<br>UCP4 (2)<br>2 other (1)<br>(10 total)	77±2<br>71±3<br>71±1<br>60-71	44±2<br>42±4<br>42±1<br>46-52	APC 28%<br>OGC 25%<br>UCP5-6 19%<br>3 other ≤ 13%<br>(22 total)	-
**3 OLD**	APC (3)<br>UCP4 (3)<br>OGC (2)<br>2 other (1)<br>(10 total)	73±3<br>68±2<br>68±0<br>63-67	42±5<br>40±1<br>45±2<br>38-40	APC 30%<br>UCP4 25%<br>OGC 21%<br>2 other ≤ 15%<br>(16 total)	UCP4 (4)<br>APC (3)<br>OGC (3)<br>(10 total)	67±4<br>77±2<br>71±3	42±2<br>44±2<br>42±4	APC 37%<br>OGC 34%<br>UCP4 34%<br>SLC25A39-A40<br>5%<br>(21 total)	-
**4**	SLC25A39-A40 (3)<br>2 other (1)<br>(5 total)	96±2<br>88-99	34±2<br>30-31	SLC25A39-A40<br>61%<br>ALC 21%<br>AGC 18%<br>(5 total)	SLC25A39-A40 (2)<br>4 other (1)<br>(6 total)	105±7<br>92-103	33±3<br>30-37	SLC25A39-A40<br>43%<br>4 other ≤ 17%<br>(7 total)	SLC25A39-A40<br>(2)
**4 OLD**	SLC25A39-A40 (3)<br>2 other (1)<br>(5 total)	96±2<br>88-99	34±2<br>30-31	SLC25A39-A40<br>61%<br>ALC 21%<br>AGC 18%<br>(5 total)	SLC25A39-A40 (3)<br>3 other (1)<br>(6 total)	102±6<br>88-99	34±3<br>31-33	SLC25A39-A40<br>53%<br>4 other ≤ 17%<br>(10 total)	SLC25A39-A40<br>(3)
**5**	MME (4.5)<br>ALC (3)<br>UCP2-3 (0.5)<br>(8 total)	131±5<br>123±11<br>124	40±2<br>41±4<br>39	MME 66%<br>ALC 20%<br>2 other ≤ 8%<br>(15 total)	SLC25A39-A40 (3)<br>MME (3)<br>2 other (1)<br>(8 total)	141±8<br>136±2<br>124-130	46±2<br>41±2<br>32-41	SLC25A39-A40<br>40%<br>MME 32%<br>4 other ≤ 12%<br>(16 total)	MME (2)
**5 OLD**	ALC (7)<br>UCP2-3 (1)<br>(8 total)	119±8*<br>124	40±3<br>39	ALC 70%<br>UCP2-3 13%<br>2 other ≤ 12%<br>(11 total)	SLC25A39-A40 (4)<br>4 other (1)<br>(8 total)	138±8*<br>116-132	45±2<br>32-42	SLC25A39-A40<br>58%<br>4 other ≤ 13%<br>(12 total)	SLC25A39-A40<br>(1)
**6**	UCP5-6 (2)<br>8 other (1)<br>(10 total)	88±9<br>81-92	35±0<br>30-33	UCP5-6 20%<br>10 other ≤ 11%<br>(13 total)	TPC (2)<br>DIC (2)<br>5 other (1)<br>(9 total)	108±9<br>98±2<br>96-108	32±1<br>31±0<br>31-36	TPC 24%<br>6 other 16%<br>(10 total)	-
**6 OLD**	CoC (2)<br>TPC (2)<br>4 other (1)	91±6<br>80±1<br>81-92	31±0<br>32±1<br>31-33	CoC 26%<br>TPC 23%<br>6 other ≤ 13%	DIC (3)<br>TPC (2)<br>SLC25A39-A40 (2)	97±2<br>108±9<br>94±4	31±0<br>32±1<br>31±0	DIC 31%<br>TPC 28%<br>SLC25A39-A40	-

	(8 total)			(10 total)	YPR011C (1)<br>(8 total)	86	31	25%<br>2 other 11%<br>(10 total)	
**7**	APC (5)<br>peCFNC (1)<br>(6 total)	86±4<br>87	37±2<br>31	APC 83%<br>peCFNC 17%<br>(6 total)	APC (4)<br>UCP4 (2)<br>(6 total)	88±2<br>84±2	37±2<br>34±2	APC 76%<br>UCP4 24%<br>(7 total)	APC (4)
**7 OLD**	APC (5)<br>peCFNC (1)<br>(6 total)	86±4<br>87	37±2<br>31	APC 83%<br>peCFNC 17%<br>(6 total)	APC (4)<br>UCP4 (2)<br>(6 total)	88±2<br>84±2	37±2<br>34±2	APC 76%<br>UCP4 24%<br>(7 total)	APC (4)

**SLC25A44 subfamily exons**	**most similar ES**				**most similar FLS**				**final prediction**
	**top ES hits (number)**	**AAS**	**ASI (%)**	**top 5% ES hits in % of summed AS (number)**	**top FLS hits (number)**	**AAS**	**ASI (%)**	**top 5% FLS hits in % of summed AS (number)**	**subfamily hit (confidence score)**
**1**	-	-	-	-	-	-	-	-	-
**1 OLD**	-	-	-	-	-	-	-	-	-
**2**	MFRN (4)<br>MFT/FAD (3)<br>2 other (1)<br>(9 total)	93±2<br>95±3<br>80-88	38±2<br>38±2<br>33-36	MFRN 50%<br>MFT/FAD 34%<br>2 other ≤ 11%<br>(10 total)	AT4G11440 (2)<br>MFRN (2)<br>5 other (1)<br>(9 total)	93±3<br>90±3<br>86-91	32±1<br>38±3<br>33-40	AT4G11440 23%<br>MFRN 17%<br>7 other ≤ 16%<br>(14 total)	(MFRN)
**2 OLD**	MFRN (5)<br>MFT/FAD (4)<br>(9 total)	90±7<br>90±9	37±2<br>36±4	MFRN 56%<br>MFT/FAD 44%<br>(10 total)	A28 /A37 (3)<br>CoC (3)<br>AGC (2)<br>MFT/FAD (1)<br>(9 total)	88±4<br>86±4<br>80±2<br>86	38±2<br>39±1<br>35±1<br>36	MFRN 29%<br>CoC 28%<br>3other ≤ 16%	MFRN (2)
**3**	APC (1)<br>AACX (1)<br>3 other (1)<br>(5 total)	97<br>90<br>78-88	32<br>32<br>30	TPC 34%<br>APC 22%<br>5 other ≤ 13%<br>(11 total)	APCX (2.5)<br>2 other (≤1)<br>(4 total)	98±3<br>96-97	31±1<br>30-32	APCX 50%<br>SLC25A39-A40<br>28%<br>NDT 22%<br>(7 total)	APCX (1)
**3 OLD**	MFRN (2)<br>4 other (1)<br>(6 total)	87±1<br>74-97	33±3<br>30-32	TPC 29%<br>APC 19%<br>5 other ≤ 15%	SLC25A39-A40 (2)<br>(2 total)	98±2	32±0	-	-

pSLC25A44 subfamily exons	most similar ES				most similar FLS				final prediction
	**top ES hits (number)**	**AAS**	**ASI (%)**	**top 5% ES hits in % of summed AS (number)**	**top FLS hits (number)**	**AAS**	**ASI (%)**	**top 5% FLS hits in % of summed AS (number)**	**subfamily hit (confidence score)**
**1**	BT (1)<br>MCART (1)<br>(2 total)	41<br>25	30<br>35	-	BT (1)<br>MCART (1)<br>(2 total)	41<br>29	41<br>29	-	-
**1 OLD**	ORC or YMC/BOU (1)<br>(1 total)	37	33	-	peCFNC (1)<br>SAMC (1)<br>(2 total)	32<br>22	30<br>33	-	-
**2**	A28 (2)<br>(2 total)	199±3	30±0	-	A28 (2)<br>(2 total)	190±12	30±0	-	(MFRN)
**2 OLD**	A28 (2)<br>(2 total)	199±3	30±0	-	A28 (2)<br>(2 total)	190±12	30±0	-	(MFRN)
**3**	NDT (3)<br>(3 total)	129±3	32±2	NDT 57%<br>YMC/BOU 43%<br>(6 total)	ORC (1)<br>NDT (1)<br>CAC (1)<br>(3 total)	151<br>144<br>123	35<br>35<br>30	NDT 44%<br>ORC 36%<br>2 other ≤ 10%<br>(4 total)	NDT (1)
**3 OLD**	NDT (3)<br>(3 total)	129±3	32±2	NDT 57%<br>YMC/BOU 43%<br>(6 total)	NDT (2)<br>ORC (1)<br>(3 total)	133±11<br>151	33±2<br>35	NDT 51%<br>ORC 37%<br>YMC/BOU 12%<br>(4 total)	NDT (2)
**4**	GGC1 (1)<br>MFRN (1)<br>CIC (1)<br>(3 total)	96<br>92<br>87	33<br>40<br>38	MFRN 34%<br>SAMC 22%<br>4 other ≤ 12%<br>(7 total)	MFRN (1)<br>AT4G11440 (1)<br>CIC (1)<br>(3 total)	135<br>103<br>102	39<br>33<br>38	-	-
**4 OLD**	SAMC (2)<br>MFRN (1)<br>(3 total)	91±5<br>92	41±4<br>40	MFRN 51%<br>SAMC 49%<br>(4 total)	MFRN (1)<br>AGC (1)<br>SAMC (1)<br>(3 total)	135<br>97<br>95	39<br>33<br>36	MFRN 56%<br>SAMC 29%<br>AGC 15%<br>(5 total)	MFRN (2)
**5**	APC (2)<br>NDT (1)	88±0*<br>106	34±0<br>30	-	UCP2-3 (2)<br>NDT (1)	110±6*<br>106	33±1<br>30	-	(UCP2-3)

	(3 total)				(3 total)				
**5 OLD**	APC (2)<br>PiC (1)<br>(3 total)	88±0*<br>84	34±0<br>46	-	UCP2-3 (3)<br>(3 total)	104±9*	34±1	-	(UCP2-3)
**6**	SAMC (1)<br>ODC (1)<br>CIC (1)<br>(3 total)	15<br>14<br>11	43<br>43<br>56	-	GlyC (1)<br>MTCH (1)<br>SLC25A45/A47/A48 (1)<br>(3 total)	13<br>12<br>10	43<br>44<br>43	-	-
**6 OLD**	SAMC (1)<br>ORC (1)<br>(2 total)	15<br>10	43<br>43	-	GlyC (1)<br>TPC (1)<br>AAC (1)<br>(3 total)	13<br>11<br>9	43<br>44<br>57	-	-

**MFRN subfamily exons**	**most similar ES**				**most similar FLS**				**final prediction**
	**top ES hits (number)**	**AAS**	**ASI (%)**	**top 5% ES hits in % of summed AS (number)**	**top FLS hits (number)**	**AAS**	**ASI (%)**	**top 5% FLS hits in % of summed AS (number)**	**subfamily hit (confidence score)**
**1**	YPR011C (2)<br>CoC (2)<br>5 other (1)<br>(9 total)	79±6<br>69±2<br>63-81	36±3<br>33±3<br>30-36	YPR011C 24%<br>ODC 16%<br>6 other ≤ 13%<br>(12 total)	ODC (5)<br>4 other (1)<br>(9 total)	87±3<br>77-82	40±4<br>30-33	ODC 63%<br>4 other ≤ 10%<br>(10 total)	ODC (2)
**1 OLD**	CoC (3)<br>YPR011C (2)<br>4 other (1)<br>(9 total)	68±2<br>79±6<br>63-81	32±2<br>36±3<br>30-36	CoC 24%<br>YPR011C 22%<br>5 other ≤ 11%<br>(10 total)	TPC (2)<br>5 other (1)<br>(7 total)	72±1<br>70-84	38±0<br>31-36	YPR011C 22%<br>DIC 20%<br>5 other ≤ 15%<br>(9 total)	-
**2**	MFT/FAD (3)<br>4 other (≤ 1)<br>(6 total)	111±10<br>106-115	34±4<br>30	MFT/FAD 41%<br>NDT 17%<br>ALC 17%<br>2 other ≤ 16%<br>(8 total)	SLC25A39-A40 (2)<br>PNC (2)<br>5 other (1)<br>(9 total)	139±1<br>128±5<br>118-147	30±0<br>30±0<br>30-33	SLC25A39-A40 24%<br>PNC 22%<br>5 other ≤ 12%<br>(10 total)	-
**2 OLD**	MFT/FAD (3)<br>4 other (1)<br>(7 total)	111±10<br>98-115	34±4<br>30	MFT/FAD 38%<br>ALC 22%<br>3 other ≤ 15%<br>(8 total)	SLC25A39-A40 (4)<br>MFT/FAD (2)<br>ALC (2)<br>2 other (1)	126±14<br>116±3<br>114±6<br>113-147	30±1<br>32±1<br>30±0<br>30	SLC25A39-A40 37%<br>MFT/FAD 22%<br>4 other ≤ 12%	-

					(9 total)			(16 total)	
**3**	APC (5)[1]<br>GC (5)[2]<br>PNC (4)<br>PiC (1)<br>(15 total)	39±2<br>35±0<br>42±1<br>30	39±3<br>46±2<br>34±3<br>37	APC 34%<br>GC 28%<br>PNC 25%<br>3 other ≤ 5%<br>(20 total)	GlyC (4)<br>SLC25A45/A47/A48 (3)<br>APC (3)<br>5 other (1)<br>(15 total)	33±1<br>35±2<br>35±2<br>33-45	42±0<br>51±2<br>46±2<br>32-53	APC 22%<br>SLC25A45/A47/<br>A48 20%<br>GlyC 16%<br>6 other ≤ 9%<br>(21 total)	-
**3 OLD**	APC (5)[1]<br>AGC (5)[2]<br>GlyC (3)<br>2 other (1)<br>(15 total)	39±2<br>32±1<br>33±7<br>30-39	39±3<br>46±2<br>45±11<br>32-37	APC 37%<br>AGC 31%<br>GlyC 18%<br>2 other ≤ 8%<br>(19 total)	GlyC (6.5)<br>APC (3.5)<br>SLC25A39-A40 (2.5)<br>3 other (≤ 1)<br>(15 total)	34±3<br>35±2<br>34±3<br>33-36	44±4<br>45±3<br>46±2<br>32-47	GlyC 34%<br>APC 26%<br>SLC25A39-A40<br>15%<br>4 other ≤ 11%<br>(24 total)	-
**4**	-	-	-	-	SLC25A39-A40 (1)<br>(1 total)	260	30	-	-
**4 OLD**	-	-	-	-	SLC25A39-A40 (1)<br>(1 total)	260	30	-	-

[1] more common with SLC25A37 sequences. [2] more common with SLC25A28 sequences.

**pMFRN subfamily exons**	**most similar ES**				**most similar FLS**				**final prediction**
	**top ES hits (number)**	**AAS**	**ASI (%)**	**top 5% ES hits in % of summed AS (number)**	**top FLS hits (number)**	**AAS**	**ASI (%)**	**top 5% FLS hits in % of summed AS (number)**	**subfamily hit (confidence score)**
**1**	-	-	-	-	-	-	-	-	
**1 OLD**	-	-	-	-	-	-	-	-	
**2**	SLC25A39-A40 (4)<br>(4 total)	125±5	37±1	SLC25A39-A40<br>100%<br>(5 total)	SLC25A39-A40 (4)<br>(4 total)	140±13	38±3	SLC25A39-A40<br>100%<br>(7 total)	SLC25A39-A40<br>(5)
**2 OLD**	SLC25A39-A40 (4)<br>(4 total)	125±5	37±1	SLC25A39-A40<br>100%<br>(5 total)	SLC25A39-A40 (4)<br>(4 total)	140±13	38±3	SLC25A39-A40<br>100%<br>(7 total)	SLC25A39-A40<br>(5)

**AT4G11440**	**most similar ES**	**most similar FLS**	**final**

subfamily exons									prediction
	top ES hits (number)	AAS	ASI (%)	top 5% ES hits in % of summed AS (number)	top FLS hits (number)	AAS	ASI (%)	top 5% FLS hits in % of summed AS (number)	subfamily hit (confidence score)
**1**	MFT/FAD (1)<br>(1 total)	74	40	-<br>(1 total)	-	-	-	-	-
**1 OLD**	MFT/FAD (1)<br>(1 total)	74	40	-<br>(1 total)	-	-	-	-	-
**2**	SLC25A39-A40 (1)<br>SAMC (1)<br>AT5G42130 (1)<br>(3 total)	81<br>79<br>63	39<br>41<br>36	SLC25A39-A40 46%<br>SAMC 45%<br>AT5G42130 9%<br>(5 total)	SAMC (3)<br>(3 total)	89±6	43±3	SAMC 100%<br>(6 total)	SAMC (1)
**2 OLD**	SLC25A39-A40 (1.5)<br>SAMC (1.5)<br>(3 total)	72±10<br>71±9	40±1<br>41±0	SLC25A39-A40 50%<br>SAMC 50%<br>(4 total)	SAMC (3)<br>(3 total)	89±6	43±3	SAMC 100%<br>(6 total)	SAMC (1)
**3**	MFRN (2)<br>MFT/FAD (1)<br>(3 total)	80±6<br>76	34±3<br>31	MFRN 68%<br>MFT/FAD 32%<br>(4 total)	MFRN (2)<br>SAMC (1)<br>(3 total)	99±2<br>82	35±2<br>41	-	MFRN (1)
**3 OLD**	MFRN (2)<br>MFT/FAD (1)<br>(3 total)	80±6<br>76	34±3<br>31	MFRN 68%<br>MFT/FAD 32%<br>(4 total)	MFRN (2)<br>SAMC (1)<br>(3 total)	99±2<br>82	35±2<br>41	-	MFRN (1)
**4**	AT5G42130 (1)<br>CAC (1)<br>OAC (1)<br>(3 total)	73<br>73<br>66	46<br>36<br>36	AT5G42130 45%<br>CAC 35%<br>2 other ≤ 10%<br>(5 total)	GC (1)<br>UCP4 (1)<br>AT5G42130 (1)<br>(3 total)	78<br>78<br>74	46<br>36<br>42	GC 34%<br>AT5G42130 33%<br>2 other ≤ 17%<br>(4 total)	-
**4 OLD**	SLC25A39-A40 (2)<br>DIC (1)<br>(3 total)	62±4<br>73	38±5<br>36	SLC25A39-A40 51%<br>DIC 49%<br>(4 total)	UCP4 (2)<br>PiC (1)<br>(3 total)	74±5<br>70	36±0<br>36	-	-
**5**	CoC (1)<br>CIC (1)<br>ODC (1)<br>(3 total)	51<br>50<br>47	31<br>35<br>43	CoC 35%<br>CIC 34%<br>ODC 32%<br>(4 total)	APC (1)<br>CIC (1)<br>YPR011C (1)<br>(3 total)	61<br>50<br>45	35<br>35<br>35	APC 39%<br>CIC 32%<br>2 other ≤ 18%<br>(5 total)	-

**5 OLD**	CoC (1)<br>YPR011C (1)<br>peCFNC (1)<br>(3 total)	51<br>45<br>39	31<br>35<br>35	-	APC (1)<br>peCFNC (1)<br>YPR011C (1)<br>(3 total)	61<br>49<br>45	35<br>31<br>35	APC 31%<br>3 other ≤ 23%<br>(4 total)	-
**6**	YPR011C (1)<br>MFT/FAD (1)<br>ALC (1)<br>(3 total)	93<br>92<br>81	61<br>57<br>57	YPR011C 35%<br>4 other ≤ 17%<br>(5 total)	AGC (1)<br>MFT/FAD (1)<br>SLC25A39-A40 (1)<br>(3 total)	95<br>88<br>86	68<br>61<br>61	AGC 42%<br>SLC25A39-A40 39%<br>3 other ≤ 7%<br>(7 total)	-
**6 OLD**	YPR011C (1)<br>MFT/FAD (1)<br>ALC (1)<br>(3 total)	93<br>92<br>81	61<br>57<br>57	YPR011C 35%<br>ALC 31%<br>2 other ≤ 17%<br>(4 total)	AGC (1)<br>MFT/FAD (1)<br>SLC25A39-A40 (1)<br>(3 total)	95<br>88<br>86	68<br>61<br>61	AGC 44%<br>SLC25A39-A40 41%<br>2 other ≤ 8%<br>(6 total)	-
**7**	MFT/FAD (1)<br>UCP2-3 (1)<br>CoC (1)<br>(3 total)	91<br>78<br>69	45<br>48<br>41	-	UCP2-3 (1)<br>MFT/FAD (1)<br>APC (1)<br>(3 total)	78<br>74<br>64	41<br>48<br>64	MFT/FAD 44%<br>UCP2-3 36%<br>2 other ≤ 10%<br>(5 total)	-
**7 OLD**	MFT/FAD (1)<br>UCP2-3 (1)<br>CoC (1)<br>(3 total)	91<br>78<br>69	45<br>48<br>41	-	UCP2-3 (1)<br>MFT/FAD (1)<br>APC (1)<br>(3 total)	78<br>74<br>64	41<br>48<br>64	MFT/FAD 49%<br>UCP2-3 36%<br>APC1 15%<br>(4 total)	-
**8**	MFRN (1)<br>other 2 (0.5)<br>(2 total)	63<br>61	31<br>34	-	SLC25A39-A40 (1)<br>PiC (1)<br>(2 total)	68<br>68	40<br>31	-	-
**8 OLD**	MFRN (1)<br>other 2 (0.5)<br>(2 total)	63<br>61	31<br>34	-	SLC25A39-A40 (1)<br>PiC (1)<br>(2 total)	68<br>68	40<br>31	-	-

**SAMC subfamily exons**	**most similar ES**				**most similar FLS**				**final prediction**
	**top ES hits (number)**	**AAS**	**ASI (%)**	**top 5% ES hits in % of summed AS (number)**	**top FLS hits (number)**	**AAS**	**ASI (%)**	**top 5% FLS hits in % of summed AS (number)**	**subfamily hit (confidence score)**
**1**	MTCH (1)<br>CIC (1)	55<br>25	42<br>45	MTCH 29%<br>OGC 19 %	SLC25A44 (3)<br>7 other (1)	23±0<br>21-45	40±4<br>31-45	SLC25A44 27%<br>MTCH 17%	-

	OGC (1)<br>4 other (1)<br>(7 total)	25<br>19-24	38<br>30-45	5 other ≤ 15%<br>(9 total)	(10 total)			6 other ≤ 13%<br>(10 total)	
**1 OLD**	OGC (2)<br>SLC25A39-A40 (2)<br>3 other (1)<br>(7 total)	23±2<br>20±1<br>16-32	42±4<br>44±1<br>36-47	OGC 29%<br>SLC25A39-A40 25%<br>3 other ≤ 20%<br>(7 total)	SLC25A44 (4)<br>AAC (2)<br>2 other (1)<br>(8 total)	24±2<br>20±4<br>19-34	38±5<br>41±5<br>31-36	SLC25A44 51%<br>AAC 21%<br>2 other ≤ 18%<br>(8 total)	SLC25A44 (1)
**2**	MME 4)<br>NDT (2)<br>CAC (2)<br>YPR011C (1)<br>(9 total)	133±7<br>134±4<br>132±3<br>127	43±2<br>42±2<br>41±3<br>36	MME 45%<br>NDT 28%<br>CAC 22%<br>YPR011C 5%<br>(12)	NDT (3)<br>MME (2.5)<br>CAC (2)<br>2 other (≤ 1)<br>(9 total)	133±7<br>131±7<br>139±4<br>122-131	40±2<br>42±2<br>40±4<br>38-40	MME 33%<br>NDT 30%<br>CAC 23%<br>4 other ≤ 4%<br>(17 total)	-
**2 OLD**	NDT (4)<br>ORC (2)<br>3 other (1)<br>(9 total)	120±15<br>111±2<br>116-127	38±4<br>34±2<br>30-36	NDT 54%<br>5 other ≤ 13%<br>(15 total)	NDT (3)<br>SFC (3)<br>CoC (2)<br>ORC (1)<br>(9 total)	133±4<br>124±2<br>130±1<br>123	40±2<br>38±2<br>40±2<br>32	NDT 35%<br>SFC 28%<br>CoC 26%<br>5 other ≤ 5%<br>(18 total)	NDT (1)
**3**	PNC (2)<br>4 other (1)<br>(6 total)	63±15<br>53-56	31±1<br>31-37	PNC 36%<br>4 other ≤ 16%<br>(7 total)	PNC (2)<br>5 other (1)<br>(7 total)	64±3<br>55-75	35±5<br>31-44	PNC 29%<br>5 other ≤ 17%<br>(7 total)	-
**3 OLD**	AGC (1)<br>MFT/FAD (1)<br>MFRN (1)<br>4 other (≤ 1)<br>(6 total)	56<br>54<br>54<br>42-53	31<br>37<br>31<br>31-34	AGC 18%<br>MFRN 18%<br>MFT/FAD 18%<br>4 other ≤ 17%<br>(7 total)	NDT (2)<br>MFRN (2)<br>2 other (1)<br>(6 total)	60±1<br>58±1<br>55-61	32±1<br>32±1<br>31-36	NDT 34%<br>MFRN 25%<br>3 other ≤ 17%<br>(7 total)	-
**4**	UCP2-3 (1)<br>SAMCX (1)<br>MME (1)<br>(3 total)	60<br>53<br>49	34<br>31<br>31	UCP2-3 37%<br>SAMCX 33%<br>2 other ≤ 15%<br>(4 total)	AT5G42130 (3)<br>AAC (1)<br>(4 total)	62±1<br>52	38±1<br>30	AT5G42130 78%<br>AAC 22%<br>(4 total)	AT5G42130 (2)
**4 OLD**	UCP2-3 (3)<br>(3 total)	52±6	32±1	-	AGC (1.5)<br>3 other (≤ 1)<br>(4 total)	56±3<br>52-53	31±0<br>30-34	AGC 39%<br>3 other ≤ 24%<br>(5 total)	-
**5**	GC (3)<br>ALC (1)<br>(4 total)	53±0<br>55	63±0<br>69	GC 58%<br>ALC 33%<br>ORC 8%<br>(6 total)	ALC (4)<br>(4 total)	53±2	64±3	ALC 65%<br>3 other ≤ 22%<br>(11 total)	GC (2)<br>ALC (2)

**5 OLD**	ALC (4)<br>(4 total)	53±2	64±3	ALC 75%<br>2 other ≤ 16%<br>(9 total)	ALC (4)<br>(4 total)	53±2	64±3	ALC 65%<br>3 other ≤ 22%<br>(11 total)	ALC (4)
**6**	GC (3.5)<br>SLC25A39-A40 (0.5)<br>(4 total)	40±4<br>38±0	54±2<br>53±0	GC 91%<br>SLC25A39-A40<br>9%<br>(13 total)	GC (3.5)<br>SLC25A39-A40<br>(0.5)<br>(4 total)	41±4<br>38±0	54±2<br>53±0	GC 91%<br>SLC25A39-A40<br>9%<br>(14 total)	GC (4)
**6 OLD**	SLC25A39-A40 (2)<br>2 other (1)<br>(4 total)	38±0<br>40-42	53±0<br>53	SLC25A39-A40<br>48%<br>2 other ≤ 26%<br>(4 total)	SLC25A39-A40<br>(2)<br>2 other (1)<br>(4 total)	38±0<br>37-39	53±0<br>33-53	SLC25A39-A40<br>50%<br>3 other ≤ 26%<br>(6 total)	-
**7**	SFC (1.5)<br>3 other (≤ 1)<br>(4 total)	52±4<br>48-54	33±0<br>33-38	SFC 39%<br>SLC25A43 26%<br>2 other ≤ 24%<br>(7 total)	SLC25A43 (1)<br>UCP4 (1)<br>2 other (1)<br>(4 total)	64<br>59<br>48-50	38<br>33<br>33-38	SLC25A43 29%<br>UCP4 27%<br>3 other ≤ 23%<br>(6 total)	-
**7 OLD**	SFC (2.5)<br>ORC (1.5)#<br>(4 total)	50±3<br>48±0	33±0<br>33±0	SFC 64%<br>ORC 36%<br>(6 total)	UCP4 (1)<br>2 other (1)<br>(3 total)	59<br>48-50	33<br>33-38	UCP4 38%<br>CoC 32%<br>2 other ≤ 15%<br>(4 total)	SFC (2)
**8**	MFRN (2)<br>2 other (1)<br>(4 total)	64±1<br>61-64	62±0<br>52-67	SAMCX 50%<br>MFRN 38%<br>SLC25A39-A40<br>12%<br>(10 total)	SAMCX (3)<br>SLC25A44 (1)<br>(4 total)	61±2<br><br>64	65±2<br><br>62	SAMCX 58%<br>SLC25A44 32%<br>MFRN 10%<br>(8 total)	SAMCX (2)
**8 OLD**	MFRN (3)<br>SLC25A39-A40 (1)<br>(4 total)	63±1<br>61	59±4<br>52	MFRN 69%<br>SLC25A39-A40<br>31%<br>(10 total)	SLC25A44 (2)#<br>MFRN (2)<br>(4 total)	62±2<br>57±1	63±0<br>55±7	MFRN 60%<br>SLC25A44 32%<br>SLC25A39-A40<br>8%<br>(10 total)	MFRN (3)
**9**	ORC (1)<br>NDT (1)<br>peANT (1)<br>(3 total)	50<br>45<br>38	32<br>32<br>32	NDT 36%<br>peANT 28%<br>2 other ≤ 19%<br>(5 total)	OAC (2)<br>ORC (1)<br>peANT (1)<br>(4 total)	51±7<br>61<br>45	44±4<br>40<br>32	OAC 49%<br>ORC 30%<br>3 other ≤ 7%<br>(6 total)	-
**9 OLD**	ORC (1)<br>NDT (1)<br>peANT (1)<br>(3 total)	50<br>45<br>38	32<br>32<br>32	NDT 52%<br>peANT 29%<br>ORC 19%<br>(4 total)	ORC (3)<br>peANT (1)<br>(4 total)	52±9<br>45	36±3<br>32	ORC 68%<br>NDT 17%<br>2 other ≤ 7%<br>(7 other)	NDT (1)<br>ORC (2)

**10**	AACX (2.3)<br>ODC (2)<br>SAMCX (1.7)<br>MME (1)<br>(7 total)	67±3<br>65±6<br>71±1<br>58	33±2<br>41±4<br>34±3<br>45	SAMCX 36%<br>ODC 24%<br>4 other ≤ 13%<br>(11 total)	ORC (5)<br>4 other (1)<br>(9 total)	70±5<br>64-87	37±3<br>34-38	ORC 48%<br>SAMCX 16%<br>4 other ≤ 13%<br>(13 total)	ORC (1)
**10 OLD**	PiC (2)<br>ORC (2)<br>4 other (≤ 1)<br>(8 total)	58±1<br>57±4<br>54-62	31±0<br>36±1<br>30-42	ORC 31%<br>PiC 25%<br>5 other ≤ 14%<br>(12 total)	ORC (5)<br>AAC (2)<br>MFT/FAD (1)<br>(8 total)	70±5<br>63±1<br>82	37±3<br>34±1<br>34	ORC 63%<br>AAC 22%<br>MFT/FAD 15%<br>(9 total)	ORC (2)

**pSAMC subfamily exons**	**most similar ES**				**most similar FLS**				**final prediction**
	**top ES hits (number)**	**AAS**	**ASI (%)**	**top 5% ES hits in % of summed AS (number)**	**top FLS hits (number)**	**AAS**	**ASI (%)**	**top 5% FLS hits in % of summed AS (number)**	**subfamily hit (confidence score)**
**1**	UCP4 (1)<br>(1 total)	26	32	-	CoC (1)<br>TPC (1)<br>(2 total)	32<br>23	32<br>32	-	-
**1 OLD**	UCP4 (1)<br>(1 total)	26	32	-	CoC (1)<br>TPC (1)<br>(2 total)	32<br>23	32<br>32	-	-
**2**	MFRN (3) #<br>APC (1)<br>(4 total)	89±2<br>86	59±0<br>56	APC 58%<br>MFRN 42%<br>(9 total)	APC (4)<br>(4 total)	87±1	59±4	APC 88%<br>AT5G42130 12%<br>(6 total)	APC (3)<br>MFRN (1) #
**2 OLD**	A28/A37# (3)<br>APC (1)<br>(4 total)	89±2<br>86	59±0<br>56	APC 58%<br>MFRN 42%<br>(9 total)	APC (4)<br>(4 total)	87±1	59±4	APC 100%<br>(5 total)	APC (3)<br>MFRN (1) #
**3**	NDT (2)<br>MFT/FAD (1)<br>APC (1)<br>(4 total)	64±8<br>54<br>53	42±0<br>30<br>38	NDT 55%<br>MFT/FAD 23%<br>APC 22%<br>(5 total)	BT (2)<br>NDT (1)<br>MFT/FAD (1)<br>(4 total)	62±2<br>71<br>58	40±3<br>42<br>38	BT 49%<br>NDT 28%<br>MFT/FAD 23%<br>(4 total)	NDT (1)
**3 OLD**	NDT (2)<br>MFT/FAD (1)<br>APC (1)<br>(4 total)	64±8<br>54<br>53	42±0<br>30<br>38	NDT 55%<br>MFT/FAD 23%<br>APC 22%<br>(5 total)	NDT (1)<br>MFT/FAD (1)<br>AAC (1)<br>2 other (0.5)	71<br>58<br>58<br>58	42<br>38<br>38<br>33	NDT 37%<br>MFT/FAD 24%<br>3 other ≤ 16%<br>(8 total)	NDT (1)

					(4 total)				
**4**	MME (2)<br>YMC/BOU (2)<br>(4 total)	44±2<br>43±5	33±0<br>35±2	MME 51%<br>YMC/BOU 49%	AT4G11440 (4)<br>(4 total)	56±4	43±3	AT4G11440 100%<br>(4 total)	AT4G11440 (2)<br>MME (1)
**4 OLD**	YMC/BOU (3)<br>DIC (1)<br>(4 total)	42±4<br>35	33±3<br>30	YMC/BOU 62%<br>DIC 22%<br>MFRN 16%<br>(7 total)	YMC/BOU (3)<br>2 other (0.5)<br>(4 total)	42±4<br>37	33±3<br>30	YMC/BOU 65%<br>MFRN 23%<br>APC 11%<br>(6 total)	YMC/BOU (4)
**5**	OAC (2)<br>SLC25A43 (1)<br>ORC (1)<br>(4 total)	42±2<br>40<br>36	53±3<br>50<br>39	OAC 40%<br>ORC 23%<br>3 other ≤ 17%<br>(7 total)	OAC (1)<br>ORC (1)<br>SLC25A43 (1)<br>MME (1)<br>(4 total)	43<br>41<br>40<br>39	56<br>50<br>50<br>33	MME 24%<br>A34-APC3 18%<br>AT5G42130 18%<br>3 other ≤ 16%<br>(11 total)	-
**5 OLD**	peCFNC (1)<br>UCP2-3 (1)<br>MFRN (1)<br>ORC (1)<br>(4 total)	40<br>38<br>36<br>36	50<br>33<br>50<br>39	ORC 46%<br>MFRN 24%<br>2 other ≤ 22%<br>(7 total)	ORC (3.7)<br>MFRN (0.3)<br>(4 total)	38±3<br>36	49±2<br>50	ORC 85%<br>MFRN 8%<br>TPC 7%<br>(11 total)	ORC (2)
**6**	NDT (2)<br>ALC (1)<br>AT5G42130 (1)<br>(4 total)	74±2<br>84<br>66	42±2<br>47<br>50	NDT 50%<br>ALC 28%<br>AT5G42130 22%<br>(4 total)	NDT (2)<br>ALC (1)<br>TPC (1)<br>(4 total)	75±2<br>74<br>68	48±2<br>47<br>41	NDT 52%<br>ALC 25%<br>2 other ≤ 12%<br>(5 total)	NDT (1)
**6 OLD**	NDT (2)<br>ALC (1)<br>APC (1)<br>(4 total)	74±2<br>84<br>66	42±2<br>47<br>34	NDT 50%<br>ALC 28%<br>APC 22%<br>(4 total)	NDT (2)<br>ALC (1)<br>TPC (1)<br>(4 total)	75±2<br>74<br>68	48±2<br>47<br>41	NDT 58%<br>ALC 29%<br>TPC 13%<br>(4 total)	NDT (1)
**7**	MME (4)<br>(4 total)	77±4	47±2	MME 92%<br>AGC 8%<br>(6 total)	MME (4)<br>(4 total)	78±3	46±1	MME 100%<br>(8 total)	MME (4)
**7 OLD**	AGC (3)<br>CoC (1)<br>(4 total)	67±5<br>63	40±3<br>37	AGC 75%<br>CoC 25%<br>(10 total)	SLC25A39-A40 (3)<br>AGC (1)<br>(4 total)	64±3<br>63	41±3<br>47	SLC25A39-A40 63%<br>AGC 31%<br>CoC 6%<br>(11 total)	AGC (2)<br>SLC25A39-A40 (2)
**8**	BTL (2)<br>MFRN (1)	35±1<br>33	44±0<br>39	BTL 68%<br>MFRN 32%	CoC (1)<br>peCFNC (1)	34<br>32	39<br>50	CoC 35%<br>peCFNC 33%	BTL (1)

	(3 total)			(5 total)	BTL (1)<br>(3 total)	31	44	BTL 32%<br>(4 total)	
**8 OLD**	CoC (1.5)<br>MFRN (1)<br>GlyC (0.5)<br>(3 total)	32±2<br>33<br>30	36±3<br>39<br>33	CoC 51%<br>MFRN 34%<br>GlyC 15%<br>(4 total)	CoC (2)<br>peCFNC (1)<br>(3 total)	32±2<br>32	36±3<br>50	-	CoC (1)
**9**	MFRN (3)<br>(3 total)	68±3	73±6	MFRN 85%<br>AT5G42130 15%<br>(4 total)	AT5G42130 (2)<br>MFRN (1)<br>(3 total)	67±2<br>68	70±5<br>80	AT5G42130 54%<br>MFRN 46%<br>(5 total)	MFRN (1)<br>AT5G42130 (1)
**9 OLD**	MFRN (3)<br>(3 total)	68±3	73±6	-	MFRN (3)<br>(3 total)	64±3	73±6	-	(MFRN)
**10**	APC (1)<br>AGC (1)<br>3 other (≤ 1)<br>(4 total)	80<br>80<br>70	59<br>56<br>44-52	APC 27%<br>AGC 27%<br>YPR011C 23%<br>4 other ≤ 6%<br>(8 total)	GC (1)<br>AGC (1)<br>YPR011C (1)<br>TPC (1)<br>(4 total)	81<br>80<br>76<br>75	56<br>56<br>44<br>48	AGC 26%<br>YPR011C 24%<br>TPC 24%<br>2 other ≤ 13%<br>(8 total)	-
**10 OLD**	APC (1)<br>AGC (1)<br>YPR011C (1)<br>MFT/FAD (1)<br>(4 total)	80<br>80<br>70	59<br>56<br>44-52	APC 27%<br>AGC 27%<br>YPR011C 23%<br>3 other ≤ 8%<br>(7 total)	APC (1)<br>AGC (1)<br>YPR011C (1)<br>TPC (1)<br>(4 total)	80<br>80<br>76<br>75	59<br>56<br>44<br>48	APC 26%<br>AGC 26%<br>YPR011C 24%<br>TPC 24%<br>(5 total)	-
**11**	SFC (1)<br>SLC25A39-A40 (1)<br>(2 total)	55<br>53	32<br>30	-	MME (2)<br>YMC/BOU (1)<br>(3 total)	69±3<br>59	32±0<br>32	-	-
**11 OLD**	SFC (1)<br>SLC25A39-A40 (1)<br>(2 total)	55<br>53	32<br>30	-	YMC/BOU (1)<br>GlyC (1)<br>(2 total)	59<br>50	32<br>32	-	-

**SAMCX subfamily exons**	**most similar ES**				**most similar FLS**				**final prediction**
	**top ES hits (number)**	**AAS**	**ASI (%)**	**top 5% ES hits in % of summed AS (number)**	**top FLS hits (number)**	**AAS**	**ASI (%)**	**top 5% FLS hits in % of summed AS (number)**	**subfamily hit (confidence score)**
**1**	ALC (2)	76±3	32±1	ALC 61%	AT5G42130 (3)	81±2	36±7	AT5G42130 65%	AT5G42130 (2)

	AT5G42130 (1)<br>MFRN (1)<br>(4 total)	84<br>68	46<br>31	AT5G42130 28%<br>MFRN 11%<br>(5 total)	ALC (1)<br>(4 total)	67	33	ALC 22%<br>UCP2-3 13%<br>(5 total)	ALC (1)
**1 OLD**	ALC (2)<br>MFRN (2)<br>(4 total)	76±3<br>62±6	32±1<br>31±0	ALC 80%<br>MFRN 13%<br>AGC 7%<br>(7 total)	ALC (2)<br>UCP2-3 (1)<br>TPC (1)<br>(4 total)	73±6<br>79<br>66	32±1<br>33<br>34	ALC 50%<br>UCP2-3 27%<br>TPC 23%<br>(4 total)	ALC (1)
**2**	MFT/FAD (2)<br>TPC (1)<br>(3 total)	79±4<br>60	34±3<br>30	MFT/FAD 73%<br>2 other ≤14%<br>(4 total)	SLC25A45/A47/A48 (1)<br>3 other (1)<br>(4 total)	84<br>69-75	31<br>31-38	NDT 37%<br>MFT/FAD 27%<br>2 other ≤ 23%<br>(6 total)	MFT/FAD (1)
**2 OLD**	MFT/FAD (2)<br>TPC (1)<br>(3 total)	79±4<br>60	34±3<br>30	MFT/FAD 73%<br>2 other ≤14%<br>(4 total)	MFT/FAD (2)<br>2 other (1)<br>(4 total)	79±4<br>69-73	34±3<br>31-38	MFT/FAD 40%<br>NDT 37%<br>YPR011C 23%<br>(5 total)	MFT/FAD (1)
**3**	A46 (1)<br>ORC (1)<br>(2 total)	45<br>33	31<br>31	-	A46 (1)<br>3 other (1)<br>(4 total)	45<br>28-36	31<br>31-38	A46 32%<br>NDT 23%<br>3 other ≤ 20%<br>(5 total)	-
**3 OLD**	ORC (1)<br>NDT (1)<br>SFC (1)<br>(2 total)	33<br>26<br>22	31<br>31<br>31	-	UCP2 (1)<br>MFRN (1)<br>NDT (1)<br>UCP4 (1)<br>(4 total)	39<br>36<br>33<br>28	31<br>39<br>31<br>35	UCP2 29%<br>NDT 24%<br>3 other ≤ 21%<br>(5 total)	-
**4**	SAMC (3)<br>MFT/FAD (1)<br>(4 total)	138±5<br>130	31±1<br>30	SAMC 76%<br>MFT/FAD 24%<br>(4 total)	SAMC (3)<br>ORC (1)<br>(4 total)	149±4<br>140	32±2<br>33	SAMC 63%<br>ORC 25%<br>2 other 6%<br>(12 total)	SAMC (4)
**4 OLD**	SAMC (3)<br>MFT/FAD (1)<br>(4 total)	138±5<br>130	31±1<br>30	SAMC 76%<br>MFT/FAD 24%<br>(4 total)	SAMC (3)<br>ORC (1)<br>(4 total)	149±4<br>140	32±2<br>33	SAMC 63%<br>ORC 25%<br>2 other 6%<br>(12 total)	SAMC (4)
**5**	A40 (2)<br>AT5G42130 (1)<br>(3 total)	195±17<br>166	33±2<br>30	-	SAMC (3)<br>SLC25A39-A40 (1)<br>(4 total)	210±8<br>197	33±2<br>32	SAMC 79%<br>SLC25A39-A40<br>21%<br>(7 total)	SAMC (2)

**5 OLD**	SLC25A39-A40 (2)<br>AGC (1)<br>(3 total)	195±17<br>149	33±2<br>30	SLC25A39-A40 73%<br>AGC 18%<br>APC 9%<br>(5 total)	SAMC (3)<br>SLC25A39-A40 (1)<br>(4 total)	210±8<br>197	33±2<br>32	SAMC 79%<br>SLC25A39-A40 21%<br>(7 total)	SAMC (2)<br>SLC25A39-A40 (1)

**MME subfamily exons**	**most similar ES**				**most similar FLS**				**final prediction**
	**top ES hits (number)**	**AAS**	**ASI (%)**	**top 5% ES hits in % of summed AS (number)**	**top FLS hits (number)**	**AAS**	**ASI (%)**	**top 5% FLS hits in % of summed AS (number)**	**subfamily hit (confidence score)**
**1**	PiC (1)<br>CAC (1)<br>(2 total)	49<br>30	33<br>39	-	-	-	-	-	-
**1 OLD**	PiC (1)<br>(1 total)	49	33	-	A3<br>(1 total)	59	33	-	-
**2**	ODC (1)<br>APC (1)<br>SAMCX (1)<br>(3 total)	90<br>89<br>82	34<br>37<br>42	ODC 35%<br>SAMCX 32%<br>2 other ≤ 17%<br>(4 total)	UCP5-6 (2)<br>SAMCX (1)<br>(3 total)	90±7<br>100	39±0<br>34	UCP5-6 51%<br>SAMCX 17%<br>2 other ≤ 12%<br>(13 total)	UCP5-6 (1)
**2 OLD**	AGC (2)<br>APC (1)<br>(3 total)	79±3<br>89	37±0<br>37	AGC 65%<br>2 other ≤ 18%<br>(4 total)	APC (1)<br>AGC (1)<br>SAMC (1)<br>(3 total)	100<br>96<br>81	45<br>37<br>37	APC 41%<br>SAMC 30%<br>2 other ≤ 19%<br>(9 total)	AGC (1)
**3**	SLC25A45/A47/A48 (1)<br>AACX (1)<br>MFRN (1)<br>(3 total)	41<br>40<br>34	35<br>40<br>45	AACX 35%<br>MFRN 30%<br>2 other ≤ 18%<br>(4 total)	PNC (1)<br>UCP4 (1)<br>MFT/FAD (1)<br>(3 total)	45<br>44<br>41	40<br>50<br>35	PNC 35%<br>UCP4 34%<br>3 other ≤ 16%<br>(6 total)	-
**3 OLD**	MFT/FAD (1)<br>SLC25A44 (1)<br>MFRN (1)<br>(3 total)	39<br>38<br>34	45<br>30<br>45	MFT/FAD 35%<br>MFRN 31%<br>2 other ≤ 17%<br>(5 total)	MFT/FAD (2)<br>UCP4 (1)<br>(3 total)	40±1<br>44	40±5<br>50	MFT/FAD 54%<br>UCP4 36%<br>SAMC 11%<br>(6 total)	MFT/FAD (1)
**4**	MFRN (1)<br>PiC (1)<br>(2 total)	82<br>74	30<br>30	-	MFT/FAD (2)<br>SAMC (1)<br>(3 total)	101±1<br>99	33±1<br>38	-	(MFT/FAD)

PiC subfamily exons	most similar ES				most similar FLS				final prediction
	top ES hits (number)	AAS	ASI (%)	top 5% ES hits in % of summed AS	top FLS hits (number)	AAS	ASI (%)	top 5% FLS hits in % of summed AS (number)	subfamily hit (confidence score)
**4 OLD**	MFRN (1)<br>PiC (1)<br>(2 total)	82<br>74	30<br>30	-	MFT/FAD (2)<br>SAMC (1)<br>(3 total)	101±1<br>99	33±1<br>38	-	(MFT/FAD)
**5**	UCP5-6 (1)<br>(1 total)	99	33	-	-	-	-	-	-
**5 OLD**	-	-	-	-	-	-	-	-	-
**6**	GlyC (2)<br>(2 total)	44±0	60±0	-	SLC25A39-A40 (2)<br>(2 total)	47±0	60±0	-	-
**6 OLD**	GlyC (2)<br>SAMC (1)<br>(3 total)	44±0<br>45	60±0<br>67	-	SLC25A39-A40 (2)<br>SAMC (1)<br>(3 total)	47±0<br>45	60±0<br>67		
**7**	-	-	-	-	-	-	-	-	-
**7 OLD**	-	-	-	-	-	-	-	-	-
**8**	AGC (1)<br>SLC25A39-A40 (1)<br>NDT (1)<br>(3 total)	74<br>64<br>63	54<br>54<br>46	AGC 49%<br>SLC25A39-A40 34%<br>4 other ≤ 8%<br>(10 total)	SAMC (2)<br>OAC (1)<br>(3 total)	70±1<br>66	67±4<br>42	SAMC 73%<br>3 other ≤ 11%<br>(8 total)	SAMC (1)
**8 OLD**	AGC (1)<br>SLC25A39-A40 (1)<br>NDT (1)<br>(3 total)	74<br>64<br>63	54<br>54<br>46	AGC 48%<br>SLC25A39-A40 33%<br>3 other ≤ 9%<br>(10 total)	SAMC (2.5)<br>UCP4 (0.5)<br>(3 total)	68±2<br>65	63±7<br>54	SAMC 78%<br>3 other ≤ 11%<br>(8 total)	SAMC (1)
**9**	BT1 (1)<br>TPC (1)<br>(2 total)	118<br>112	33<br>31	-	ODC (1)<br>AGC (1)<br>(2 total)	135<br>112	32<br>30	-	-
**9 OLD**	TPC (1)<br>(1 total)	112	31	-	AGC (2)<br>(2 total)	113±1	30±0	AGC 61%<br>2 other ≤ 23%<br>(5 total)	AGC (1)

				**(number)**					
**1**	CAC (1)<br>ALC (1)<br>(2 total)	51<br>30	30<br>33	-	MFRN (1)<br>YPR011C (1)<br>(2 total)	42<br>38	30<br>31	-	-
**1 OLD**	ALC (1)<br>(1 total)	30	33	-	MFRN (1)<br>YPR011C (1)<br>(2 total)	42<br>38	30<br>31	-	-
**2**	TPC (4)<br>3 other (1)<br>(7 total)	70±14*<br>60-74	32±1<br>30-35	TPC 57%<br>3 other ≤ 15%<br>(8 total)	MFT/FAD (4)<br>4 other (4)<br>(8 total)	119±4*<br>76-101	46±2<br>31-36	MFT/FAD 59%<br>5 other ≤ 13%<br>(9 total)	MFT/FAD (1)
**2 OLD**	TPC (4)<br>APC (2)<br>ORC (1)<br>(7 total)	70±14*<br>71±4<br>60	32±1<br>34±2<br>30	TPC 58%<br>APC 29%<br>ORC 13%<br>(8 total)	MFT/FAD (4)<br>TPC (3)<br>GlyC (1)<br>(8 total)	119±4*<br>81±4<br>76	46±2<br>35±2<br>32	MFT/FAD 60%<br>TPC 25%<br>2 other ≤ 10%<br>(9 total)	MFT/FAD (1)
**3**	MFRN (3)<br>YPR011C (2)<br>CoC (1)<br>(6 total)	97±3<br>94±4<br>47	33±1<br>39±2<br>31	MFRN 51%<br>YPR011C 39%<br>CoC 10%<br>(7 total)	YPR011C (3)<br>CoC (2)<br>(5 total)	111±7<br>109±7	31±1<br>36±1	CoC 44%<br>YPR011C 30%<br>2 other ≤ 16%<br>(11 total)	MFRN (1)<br>YPR011C (1)
**3 OLD**	MFRN (3)<br>YPR011C (2)<br>CoC (1)<br>(6 total)	97±3<br>94±4<br>47	33±1<br>39±2<br>31	MFRN 51%<br>YPR011C 39%<br>CoC 10%<br>(7 total)	YPR011C (3)<br>CoC (2)<br>(5 total)	111±7<br>109±7	31±1<br>36±1	CoC 46%<br>YPR011C 45%<br>MFRN 9%<br>(9 total)	MFRN (1)<br>YPR011C (1)
**4**	GlyC (2)<br>2 other (1)<br>(4 total)	79±5<br>98	35±2<br>31	GlyC 45%<br>TPC 28%<br>ODC 28%<br>(4 total)	UCP2-3 (3)<br>3 other (1)<br>(6 total)	95±7<br>90-114	34±1<br>30-31	UCP2-3 41%<br>APC 19%<br>5 other ≤ 9%<br>(9 total)	-
**4 OLD**	GlyC (2)<br>TPC (1)<br>(3 total)	79±5<br>98	35±2<br>31	-	UCP2-3 (3)<br>2 other (1)<br>(5 total)	95±7<br>104-114	34±1<br>30-31	UCP2-3 49%<br>APC 23%<br>3 other ≤ 10%<br>(7 total)	UCP2-3 (1)
**5**	-	-	-	-	SLC25A39-A40 (1)<br>ODC (1)<br>(2 total)	98<br>88	30<br>31	-	-
**5 OLD**	-	-	-	-	A40 (1)<br>(1 total)	98	30	-	-

**6**	A36 (1)<br>ODC (1)<br>SAMC (1)<br>CIC (1)<br>(4 total)	53<br>50<br>49<br>48	32<br>30<br>30<br>30	ODC 25%<br>SAMC 25%<br>CIC 24%<br>2 other ≤ 13%<br>(5 total)	CAC (2)<br>4 other<br>(6 total)	60±7<br>58-65	30±0<br>30-32	CAC 23%<br>SLC25A44 21%<br>AGC 21%<br>2 other ≤ 17%<br>(8 total)	-
**6 OLD**	SAMC (1)<br>DIC (1)<br>(2 total)	49<br>44	30<br>30	-	SLC25A44 (2)<br>ALC (1)<br>AGC (1)<br>(4 total)	59±3<br>65<br>58	34±2<br>30<br>30	SLC25A44 49%<br>ALC 27%<br>AGC 24%<br>(4 total)	-
**7**	ODC (1)<br>(1 total)	61	32	-	TAAC (1)<br>(1 total)	94	30	-	-
**7 OLD**	-	-	-	-	-	-	-	-	-

**pPiC subfamily exons**	**most similar ES**				**most similar FLS**				**final prediction**
	**top ES hits (number)**	**AAS**	**ASI (%)**	**top 5% ES hits in % of summed AS (number)**	**top FLS hits (number)**	**AAS**	**ASI (%)**	**top 5% FLS hits in % of summed AS (number)**	**subfamily hit (confidence score)**
**1**	MFRN (1)<br>CoC (1)<br>AAC (1)<br>(3 total)	129<br>112<br>71	30<br>35<br>30	-	MFRN (1)<br>CIC (1)<br>GlyC (1)<br>(3 total)	129<br>94<br>71	30<br>31<br>33	-	-
**1 OLD**	MFRN (1)<br>CoC (1)<br>AAC (1)<br>TPC (1)<br>(4 total)	129<br>112<br>71<br>60	30<br>35<br>30<br>30	MFRN 34%<br>CoC 30%<br>2 other ≤ 19%<br>(7 total)	MFRN (1)<br>GlyC (1)<br>(2 total)	129<br>71	30<br>33	-	-
**2**	-	-	-	-	MFT/FAD (2)<br>(2 total)	198±4	31±0	-	-
**2 OLD**	-	-	-	-	MFT/FAD (2)<br>MFRN (1)<br>(3 total)	198±4<br>162	31±0<br>31	-	-
**3**	UCP4 (4)<br>3 other (1)	48±2<br>37-49	53±5<br>33-47	UCP4 42%<br>GC 31%	UCP4 (4)<br>3 other (1)	51±3<br>43-58	58±3<br>40-67	UCP4 65%<br>3 other ≤ 16%	UCP4 (3)

	(7 total)			4 other ≤ 12%<br>(14 total)	(7 total)			(9 total)	
**3 OLD**	UCP4 (4)<br>3 other (1)<br>(7 total)	48±2<br>36-49	53±5<br>33-47	UCP4 52%<br>TPC 16%<br>5 other ≤ 12%<br>(10 total)	UCP4 (4)<br>AGC (2)<br>TPC (1)<br>(7 total)	51±3<br>48±3<br>43	58±3<br>44±4<br>47	UCP4 67%<br>AGC 20%<br>TPC 12%<br>(8 total)	UCP4 (4)
**4**	-	-	-	-	-	-	-	-	-
**4 OLD**	-	-	-	-	-	-	-	-	-
**5**	SLC25A39-A40 (1)<br>MFRN (1)<br>(2 total)	71<br>62	33<br>31	-	SLC25A39-A40 (1)<br>MFRN (1)<br>(2 total)	71<br>62	33<br>31	-	-
**5 OLD**	SLC25A39-A40 (1)<br>MFRN (1)<br>(2 total)	71<br>62	33<br>31	-	SLC25A39-A40 (1)<br>MFRN (1)<br>(2 total)	71<br>62	33<br>31	-	-
**6**	UCP5-6 (1)<br>CAC (1)<br>4 other (1)<br>(6 total)	29<br>28<br>21-27	47<br>37<br>31-44	CAC 18%<br>SLC25A45/A47/48 17%<br>CoC 17%<br>4 other ≤ 16%<br>(7 total)	PNC (1.5)<br>6 other (≤ 1)<br>(7 total)	30±2<br>19-28	42±1<br>33-42	PNC 31%<br>SLC25A44 15%<br>5 other ≤ 14%<br>(9 total)	-
**6 OLD**	NDT (1)<br>CoC (1)<br>3 other (1)<br>(6 total)	28<br>27<br>21-26	47<br>33<br>31-44	NDT 22%<br>CoC 21%<br>SLC25A44 20%<br>3 other ≤ 17%<br>(7 total)	MFRN (2)<br>5 other (1)<br>(7 total)	28±0<br>19-26	39±4<br>33-44	MFRN 35%<br>SLC25A44 16%<br>CoC 16%<br>3 other ≤ 13%<br>(9 total)	-

**SLC25A46 subfamily exons**	**most similar ES**				**most similar FLS**				**final prediction**
	**top ES hits (number)**	**AAS**	**ASI (%)**	**top 5% ES hits in % of summed AS (number)**	**top FLS hits (number)**	**AAS**	**ASI (%)**	**top 5% FLS hits in % of summed AS (number)**	**subfamily hit (confidence score)**
**1**	-	-	-	-	-	-	-	-	-
**1 OLD**	-	-	-	-	-	-	-	-	-
**2**	APCX (4)	26±2	38±3	APCX 82%	CIC (3)	27±0	43±0	CIC 78%	APCX (2)

	A8/UCP3 (1)<br>(5 total)	23	43	A8/UCP3 18%<br>(6 total)	GC (1)<br>(4 total)	23	43	2 other ≤ 11%<br>(5 total)	CIC (2)
**2 OLD**	A8/UCP3 (2.5)<br>SLC25A39-A40 (1.5)<br>CoC (1)<br>(5 total)	22±0<br>31±7<br>25	38±3<br>36±0<br>36	A8/UCP3 42%<br>SLC25A39-A40 25%<br>2 other ≤ 17%<br>(9 total)	CIC (3.25)<br>SLC25A44 (1)<br>3 other (0.25)<br>(5 total)	26±2<br>19<br>22	41±3<br>50<br>36	CIC 70%<br>SLC25A44 16%<br>3 other ≤ 7%<br>(10 total)	CIC (2)
**3**	CoC (3)<br>MFRN (2)<br>(5 total)	29±2<br>29±2	40±3<br>37±0	CoC 60%<br>MFRN 40%<br>(12 total)	CoC (4.5)<br>MFRN (0.5)<br>(5 total)	26±2<br>24	37±0<br>37	CoC 79%<br>MFRN 21%<br>(7 total)	CoC (4)
**3 OLD**	CoC (3)<br>MFRN (2)<br>(5 total)	29±2<br>29±2	40±3<br>37±0	CoC 60%<br>MFRN 40%<br>(12 total)	CoC (4.5)<br>MFRN (0.5)<br>(5 total)	26±2<br>24	37±0<br>37	CoC 79%<br>MFRN 21%<br>(7 total)	CoC (4)
**4**	DIC (1)<br>(1 total)	37	31	-	GC (1)<br>SAMC (1)<br>(2 total)	32<br>23	35<br>35	-	-
**4 OLD**	DIC (1)<br>SLC25A44 (1)<br>(2 total)	37<br>21	31<br>31	-	TPC (1)<br>SAMC (1)<br>(2 total)	31<br>23	35<br>35	-	-
**5**	ORC (0.5)<br>YMC/BOU (0.5<br>(1 total)	61<br>61	32<br>32	-	OAC (2)<br>UGO1 (1)<br>(3 total)	67±1<br>67	32±0<br>35	-	-
**5 OLD**	ORC (0.5)<br>YMC/BOU (0.5<br>(1 total)	61<br>61	32<br>32	-	SLC25A39-A40 (3)<br>(3 total)	62±1	36±1	-	-
**6**	AT4G03115 (2)<br>AT4G11440 (2)<br>MFT/FAD (2)<br>2 other (1)<br>(8 total)	33±3<br>31±1<br>23±4<br>21-22	38±3<br>35±3<br>35±3<br>32-37	AT4G03115 30%<br>AT4G11440 29%<br>MFT/FAD 21%<br>2 other ≤10%<br>(8 total)	MFT/FAD (4)<br>5 other (1)<br>(9 total)	26±2<br>20-33	33±2<br>32-42	MFT/FAD 39%<br>6 other ≤ 14%<br>(13 total)	-
**6 OLD**	MFT/FAD (3)<br>2 other (1)<br>(5 total)	22±3<br>21-29	34±2<br>32-42	MFT/FAD 57%<br>CoC 25%<br>AAC 18%<br>(5 total)	MFT/FAD (4)<br>5 other (1)<br>(9 total)	26±2	33±2	MFT/FAD 41%<br>5 other ≤ 14%<br>(10 total)	MFT/FAD (2)
**7**	YMC/BOU (1)<br>(1 total)	23	37	-	SLC25A43 (3)<br>other 3 (1)<br>(6 total)	31±1	42±0	SLC25A43 58%<br>OAC 17%<br>4 other ≤ 8%	SLC25A43 (2)

								(9 total)	
**7 OLD**	YMC/BOU (1)<br>(1 total)	23	37	-	YMC/BOU (1)<br>TPC (1)<br>CoC (1)<br>A38(1)	23<br>21<br>21<br>20	37<br>32<br>32<br>32	TPC 25%<br>CoC 25%<br>4 other ≤ 14%<br>(6 total)	-
**8**	-	-	-	-	-	-	-	-	-
**8 OLD**	-	-	-	-	-	-	-	-	-

**MTCH subfamily exons**	**most similar ES**				**most similar FLS**				**final prediction**
	**top ES hits (number)**	**AAS**	**ASI (%)**	**top 5% ES hits in % of summed AS (number)**	**top FLS hits (number)**	**AAS**	**ASI (%)**	**top 5% FLS hits in % of summed AS (number)**	**subfamily hit (confidence score)**
**1**	NDT (1)<br>CAC (1)<br>5 other (1)<br>(7 total)	107<br>67<br>42-55	30<br>43<br>31-42	NDT 26%<br>CAC 16%<br>8 other ≤ 13%<br>(11 total)	CAC (2)<br>OGC (2)<br>3 other ≤ 1<br>(6 total)	56±12<br>48±1<br>47-55	44±1<br>31<br>31-45	CAC 26%<br>ODC 18%<br>7 other ≤13%<br>(13 total)	-
**1 OLD**	NDT (1)<br>CAC (1)<br>5 other (1)<br>(7 total)	107<br>67<br>42-55	30<br>43<br>31-42	NDT 27%<br>CAC 17%<br>6 other ≤ 14%<br>(11 total)	CAC (2)<br>OGC (2)<br>3 other ≤ 1<br>(6 total)	56±12<br>48±1<br>47-55	44±1<br>31<br>31-45	CAC 35%<br>ODC 17%<br>5 other ≤ 15%<br>(9 total)	-
**2**	-	-	-	-	TPC (1)<br>SFC (1)<br>5 other (1)<br>(7 total)	50<br>49<br>33-46	31<br>34<br>31-34	TPC 17%<br>SFC 17%<br>5 other ≤ 16%<br>(7 total)	-
**2 OLD**	-	-	-	-	TPC (1)<br>SFC (1)<br>4 other (1)<br>(6 total)	50<br>49<br>33-46	31<br>34<br>31-34	TPC 20%<br>SFC 19%<br>4 other ≤ 18%<br>(6 total)	-
**3**	TPC (2)<br>SLC25A39-A40 (2)<br>4 other (1)<br>(8 total)	71±4<br>69±5<br>60-75	37±3<br>39±2<br>31-40	TPC 31%<br>SLC25A39-A40 30%<br>5 other ≤ 11%<br>(12 total)	PNC (2)<br>7 other (1)<br>(9 total)	70±4<br>43-86	39±2<br>31-40	PNC 26%<br>AAC 18%<br>7 other ≤ 12%<br>(14 total)	-

**3 OLD**	TPC (2)<br>SLC25A39-A40 (2)<br>4 other (1)<br>(8 total)	71±4<br>69±5<br>60-75	37±3<br>39±2<br>31-40	TPC 31%<br>SLC25A39-A40 30%<br>4 other ≤ 16%<br>(11 total)	PNC (2)<br>7 other (1)<br>(9 total)	70±4<br>43-86	39±2<br>31-40	PNC 26%<br>AAC 18%<br>7 other ≤ 12%<br>(14 total)	-
**4**	SLC25A45/A47/A48<br>(1)<br>ORC (1)<br>PNC (1)<br>BTL (1)<br>(4 total)	9-17	33-56	-	APC (1)<br>GlyC (1)<br>PNC (1)<br>BTL (1)<br>(4 total)	9-13	44-67	-	-
**4 OLD**	ORC (1)<br>PNC (1)<br>(42total)	9<br>13	56<br>44	-	APC (1)<br>GlyC (1)<br>PNC (1)<br>(3 total)	12-113	44-67	-	-
**5**	peCFNC (1.33)<br>A46 (1)<br>PiC (0.67)<br>(3 total)	20-28	32-39	-	ODC (1)<br>A46 (1)<br>PiC (1)<br>GC (1)<br>(4 total)	19-26	32-39	-	-
**5 OLD**	peCFNC (1.33)<br>PiC (0.67)<br>(2 total)	20-28	32-35	-	ODC (1)<br>PiC (1)<br>(2 total)	20-23	33-35	-	-
**6**	DIC (2)<br>CAC (2)<br>3 other (1)<br>(7 total)	37±3<br>38±3<br>34-37	38±8<br>36±1<br>35	DIC 22%<br>CAC 29%<br>4 other ≤ 18%<br>(9 total)	SLC25A39-A40 (3)<br>4 other (1)<br>(7 total)	37±1<br>34-40	40±0<br>35-40	SLC25A39-<br>A40 43%<br>6 other ≤ 15%<br>(10 total)	-
**6 OLD**	DIC (2)<br>CAC (2)<br>3 other (1)<br>(7 total)	37±3<br>38±3<br>28-37	38±8<br>36±1<br>35-50	CAC 30%<br>DIC 25%<br>3 other ≤ 19%<br>(8 total)	SLC25A39-A40 (3)<br>4 other (1)<br>(7 total)	37±1<br>34-40	40±0<br>35-40	SLC25A39-<br>A40 43%<br>6 other ≤ 15%<br>(10 total)	-
**7**	PiC (4)<br>2 other (1)<br>(6 total)	23±2<br>23-28	33±0<br>41-47	PiC 56%<br>ALC 20%<br>2 other ≤ 16%<br>(7 total)	UCP4 (3)<br>2 other (1)<br>(5 total)	19±3<br>20-28	39±6<br>35-41	UCP4 55%<br>ALC 27%<br>2 other ≤1 0%<br>(6 total)	PiC (2)<br>UCP4 (2)
**7 OLD**	PiC (4)<br>2 other (1)<br>(6 total)	23±2<br>23-28	33±0<br>41-47	PiC 56%<br>ALC 20%<br>2 other ≤ 16%	UCP4 (3)<br>2 other (1)<br>(5 total)	19±3<br>20-28	39±6<br>35-41	UCP4 55%<br>ALC 27%<br>2 other ≤1 0%	PiC (2)<br>UCP4 (2)

				(7 total)				(6 total)	
**8**	OAC (3)<br>4 other (1)<br>(7 total)	61±5<br>39-66	60±4<br>35-60	OAC 40%<br>5 other ≤ 19%<br>(13 total)	OAC (3)<br>4 other (1)<br>(6 total)	68±5<br>40-56	63±5<br>40-60	OAC 51%<br>5 other ≤14%<br>(10 total)	OAC (1)
**8 OLD**	OAC (3)<br>4 other (1)<br>(7 total)	61±5<br>39-66	60±4<br>35-60	OAC 40%<br>5 other ≤ 19%<br>(13 total)	OAC (3)<br>4 other (1)<br>(6 total)	68±5<br>40-56	63±5<br>40-60	OAC 51%<br>5 other ≤14%<br>(10 total)	OAC (1)
**9**	APC (2)<br>MCART (1)<br>(3 total)	44±0<br>42	32±0<br>35	-	APC (3)<br>SLC25A39-A40 (1)<br>(4 total)	43±4<br>57	35±0<br>35	APC 70%<br>2 other ≤ 20%	APC (2)
**9 OLD**	APC (2)<br>(2 total)	44±0	32±0	-	APC (3)<br>SLC25A39-A40 (1)<br>(4 total)	43±4<br>57	35±0<br>35	APC 69%<br>A40 31%<br>(4 total)	APC (2)
**10**	ORC (2)<br>3 other (1)<br>(5 total)	20±5<br>20-23	35±4<br>31-53	ORC 38%<br>3 other ≤ 22%<br>(5 total)	OGC (2)<br>3 other (1)<br>(5 total)	19±2<br>20-25	46±8<br>31-38	OGC 36%<br>DIC 24%<br>3 other ≤ 19%<br>(7 total)	-
**10 OLD**	ORC (2)<br>3 other (1)<br>(5 total)	20±5<br>20-23	35±4<br>31-53	ORC 38%<br>3 other ≤ 22%<br>(5 total)	OGC (2)<br>3 other (1)<br>(5 total)	19±2<br>20-25	46±8<br>31-38	OGC 36%<br>DIC 24%<br>2 other ≤ 20%<br>(6 total)	-
**11**	CoC (5)<br>peCFNC (3)<br>2 other (1)<br>(10 total)	41±5<br>40±1<br>32-41	43±4<br>32±2<br>30-35	CoC 63%<br>peCFNC 17%<br>3 other ≤ 10%<br>(19 total)	PiC (3)<br>CoC (2)<br>TPC (2)<br>3 other (1)<br>(10 total)	41±1<br>42±6<br>44±1<br>35-38	42±2<br>41±5<br>33±3<br>35-39	PiC 29%<br>CoC 20%<br>TPC 16%<br>5 other ≤ 9%<br>(13 total)	CoC (1)
**11 OLD**	CoC (5)<br>peCFNC (3)<br>2 other (1)<br>(10 total)	41±5<br>40±1<br>32-41	43±4<br>32±2<br>30-35	CoC 63%<br>peCFNC 17%<br>3 other ≤ 10%<br>(19 total)	PiC (3)<br>CoC (3)<br>TPC (2)<br>3 other (1)<br>(10 total)	41±1<br>37±7<br>44±1<br>29-38	42±2<br>39±6<br>33±3<br>35-39	PiC 30%<br>CoC 27%<br>TPC 16%<br>4 other ≤ 9%<br>(13 total)	CoC (1)
**12**	AAC (3)<br>2 other (1)<br>(5 total)	45±3<br>35-41	33±2<br>32	AAC 52%<br>3 other ≤ 20%<br>(6 total)	AGC (3)<br>AAC (2)<br>CoC (1)<br>(6 total)	50±5<br>44±4<br>52	35±4<br>34±2<br>32	AGC 44%<br>AAC 31%<br>2 other ≤ 18%<br>(7 total)	AAC (2)
**12 OLD**	AAC (3)	45±3	33±2	AAC 52%	AGC (3)	50±5	35±4	AGC 44%	AAC (2)

	2 other (1)<br>(5 total)	35-41	32	3 other ≤ 20%<br>(6 total)	AAC (2)<br>CoC (1)<br>(6 total)	44±4<br>52	34±2<br>32	AAC 31%<br>2 other ≤ 18%<br>(7 total)	
**13**	APC (1)<br>CoC (1)<br>(2 total)	43<br>29	33<br>30	-	AT5G42130 (1)<br>CoC (1)<br>ORC (1)<br>(3 total)	58<br>29<br>27	37<br>30<br>32	-	-
**13 OLD**	APC (1)<br>CoC (1)<br>(2 total)	43<br>29	33<br>30	-	CoC (1)<br>ORC (1)<br>(2 total)	29<br>27	30<br>32	-	-

**MCART subfamily exons (insect)**	**most similar ES**				**most similar FLS**				**final prediction**
	**top ES hits (number)**	**AAS**	**ASI (%)**	**top 5% ES hits in % of summed AS (number)**	**top FLS hits (number)**	**AAS**	**ASI (%)**	**top 5% FLS hits in % of summed AS (number)**	**subfamily hit (confidence score)**
**1**	-	-	-	-	TPC (1)<br>(1 total)	63	30	-	(TPC)
**1 OLD**	-	-	-	-	TPC (1)<br>(1 total)	63	30	-	(TPC)
**2**	-	-	-	-	-	-	-	-	-
**2 OLD**	-	-	-	-	-	-	-	-	-

**AT4G15010 subfamily exons**	**most similar ES**				**most similar FLS**				**final prediction**
	**top ES hits (number)**	**AAS**	**ASI (%)**	**top 5% ES hits in % of summed AS (number)**	**top FLS hits (number)**	**AAS**	**ASI (%)**	**top 5% FLS hits in % of summed AS (number)**	**subfamily hit (confidence score)**
**1**	MFT/FAD (1)<br>MFRN (1)<br>AT4G11440 (1)<br>(3 total)	76<br>67<br>65	31<br>30<br>31	AT4G11440 31%<br>MFRN 28%<br>MFT/FAD 25%<br>AGC 16%<br>(6 total)	peCFNC (1)<br>SAMC (1)<br>GC (1)<br>(3 total)	79<br>76<br>62	39<br>39<br>32	peCFNC 42%<br>SAMC 21%<br>MFRN 20%<br>GC 17%<br>(4 total)	-

**1 OLD**	MFRN (2)<br>MFT/FAD (1)<br>(3 total)	64±4<br>76	32±2<br>31	MFRN 49%<br>MFT/FAD 25%<br>2 other ≤ 16%<br>(8 total)	peCFNC (1)<br>SAMC (1)<br>(2 total)	79<br>76	39<br>39	-	-
**2**	AGC (1)<br>peCFNC (1)<br>(2 total)	51<br>35	39<br>30	-	TPC (1)<br>TAAC (1)<br>(2 total)	49<br>35	35<br>37	-<br>(2 total)	-
**2 OLD**	AGC (1)<br>peCFNC (1)<br>(2 total)	51<br>35	39<br>30	-	TPC (1)<br>2 other (0.5)<br>(2 total)	49<br>35	35<br>30	-	-
**3**	CIC (1)<br>CoC (1)<br>ORC (1)<br>(3 total)	67<br>67<br>52	47<br>41<br>38	CIC 36%<br>CoC 36%<br>2 other ≤14%<br>(4 total)	APCX (1)<br>SAMC (1)<br>CIC (1)<br>(3 total)	74<br>74<br>66	44<br>44<br>47	CIC 38%<br>APCX 21%<br>SAMC 21%<br>CoC 20 %<br>(4 total)	-
**3 OLD**	CoC (1)<br>ALC (1)<br>ORC (1)<br>(3 total)	67<br>58<br>52	41<br>31<br>38	CoC 38%<br>ALC 33%<br>ORC 29%<br>(4 total)	SAMC (1)<br>CoC (1)<br>SFC<br>(3 total)	74<br>71<br>66	44<br>47<br>38	-	-
**4**	-	-	-	-	-	-	-	-	-
**4 OLD**	-	-	-	-	-	-	-	-	-
**5**	CAC (1)<br>UGO1 (1)<br>(2 total)	72<br>66	37<br>33	-<br>(3 total)	CAC (2)<br>SAMC (1)<br>(3 total)	73±5<br>77	32±1<br>31	CAC 42%<br>SAMC 27%<br>2 other ≤17%<br>(6 total)	-
**5 OLD**	UCP2-3 (1)<br>TPC (1)<br>SLC25A39-A40 (1)<br>(3 total)	60<br>56<br>54	37<br>36<br>31	UCP2-3 35%<br>TPC 33%<br>2 other ≤16%<br>(4 total)	SLC25A39-A40 (2)<br>SAMC (1)<br>(3 total)	61±4<br>77	31±0<br>31	SLC25A39-A40 62%<br>SAMC 38%<br>(4 total)	SLC25A39-A40<br>(1)
**6**	-	-	-	-	-	-	-	-	-
**6 OLD**	-	-	-	-	-	-	-	-	-

TABLE S3. Predictions of the most similar MC subfamily transmembrane α-helices. The table is divided into sections of MC subfamilies based on the main phylogenetic clusters: (a) MC-NT2; (b) MC-NT1; (c) MC-CA; (d) MC-AAP; (e) MC-AAN; (f) MCs outside the main clusters; and more or less in the order of the phylogenetic tree (Figure S1). In the MC subfamily column of queries the OLD H1-H6 sequence set is indicated when the total set is not used in the analysis. The results for the most similar transmembrane α-helices are indicated with the percentage of the number of subfamily H1-H6 hits with the highest SI to the same transmembrane α-helix of all orthologs of the same subfamily. The "most similar" MC subfamily transmembrane α-helix prediction was considered; strong (in bold), if the hit subfamily was found in more than 50% hits and was mutually found in more than 50% of the hit subfamily; good, if the hit subfamily was found in more than 50% hits; and weak (light green), if the precentage was at least 40% and double than the second best. For the strong and good predictions, the average SI of the hits is given in parentheses with the standard errors of the mean. The remaining results are shown in gray. Source: *mainly depending on one sequence hit.

Table S3. Predictions of the most similar MC subfamily transmembrane α-helices.

MC subfamily	most similar MC transmembrane α-helices					
	**H1**	**H2**	**H3**	**H4**	**H5**	**H6**
**a. MC-NT2**						
**NDT**	SAMC_H5 22<br>SLC25A39-A40_H5 15	SLC25A39-A40_H2 38<br>PNC_H2 23	MFT/FAD_H3 71 (69±3)	**MFT/FAD_H4 89 (62±5)**	**PNC_H5 80 (63±3)**	MFT/FAD_H6 62 (58±6)
**NDT OLD**	SAMC_H5 29<br>SLC25A39-A40_H5 26	SLC25A39-A40_H2 49<br>SAMC_H2 20	**MFT/FAD_H3 90 (69±3)**	**MFT/FAD_H4 89 (62±5)**	**MFT/FAD_H5 74 (58±3)**	MFT/FAD_H6 66 (58±6)
**PNC**	SLC25A39-A40_H5 54 (66±3)	APCX_H4 42<br>AGC_H2 15	NDT_H3 50<br>MFT/FAD_H3 34	NDT_H4 59 (44±4)	**NDT_H5 83 (59±4)**	MFT/FAD_H6 36<br>NDT_H6 13
**PNC OLD**	SLC25A39-A40_H5 70 (66±3)	AGC_H2 29<br>SAMC_H2 18	NDT_H3 50<br>MFT/FAD_H3 35	NDT_H4 61 (44±4)	NDT_H5 88 (59±4)	MFT/FAD_H6 37<br>NDT_H6 14
**MFT/FAD**	CAC_H1 19<br>TPC_H5 10	MFRN_H2 19<br>PNC_H2 15	NDT_H3 49<br>peCFNC_H3 18	**NDT_H4 58 (60±5)**	NDT_H5 46<br>PNC_H5 29	PNC_H6 32<br>NDT_H6 31
**MFT/FAD OLD**	ALC_H3 19<br>GlyC_H4 13	MFRN_H2 21<br>AAC_H4 13	**NDT_H3 65 (65±5)**	**NDT_H4 59 (59±7)**	**NDT_H5 52 (57±4)**	NDT_H6 39<br>APC_H6 26
**peCFNC**	CoC_H3 27<br>peANT_H1 23	PNC_H2 17<br>NDT_H2 17	SAMC_H5 26<br>MFT/FAD_H3 25	**peANT_H4 53 (60±3)**	MFT/FAD_H5 21<br>TAAC_H1 13	peANT_H6* 36<br>MCART_H2 21
**peCFNC OLD**	CoC_H3 35<br>peANT_H1 28	NDT_H2 32<br>MFT/FAD_H2 14	SAMC_H5 26<br>MFT/FAD_H3 25	**peANT_H4 53 (60±3)**	MFT/FAD_H5 27<br>peANT_H5 20	**peANT_H6* 58 (54±6)**
**peANT**	SLC25A45/A47/A48_H1* 46<br>AGC_H1 15	peCFNC_H2 37<br>ODC_H4 14	AAC_H3 25<br>SLC25A39-A40_H5 21	**peCFNC_H4 83 (60±5)**	peCFNC_H5 60 (60±5)	peCFNC_H6 62 (56±8)
**peANT OLD**	peCFNC_H5 43<br>AGC_H1 18	peCFNC_H2 64 (46±2)	AAC_H3 36<br>SLC25A39-A40_H5 21	**peCFNC_H4 86 (60±5)**	peCFNC_H5 61 (60±5)	**peCFNC_H6 64 (56±8)**
**b. MC-NT1**						
**AAC**	CoC_H1 73 (66±4)	AACX_H2* 50<br>BT_H2 33	YPR011C_H3 73 (62±4)	SLC25A44_H2 47<br>A1_H2 10	TAAC_H5 41<br>CoC_H4 30	OAC_H4 27<br>MTCH_H2 23

**AAC OLD**	CoC_H1 93 (66±4)	CoC_H2 40<br>YPR011C_H2 11	YPR011C_H3 74 (62±4)	SLC25A44_H2 59 (49±3)	CoC_H5 63 (48±4)	ORC_H4 25<br>NDT_H6 18
**TAAC**	**CoC_H1 83 (75±3)**	YPR011C_H2 50<br>SLC25A43_H2* 42	TPC_H3 53 (64±2)	PNC_H2 51 (64±6)	AAC_H5* 89 (60±6)	APC_H6 29<br>APC_H4 25
**TAAC OLD**	CoC_H1 83 (75±3)	YPR011C_H2 73 (66±7)	TPC_H3 53 (64±2)	APC_H4 30<br>CoC_H4 21	AAC_H5* 89 (60±6)	APC_H6 29<br>APC_H4 25
**AACX**	YPR011C_H1 60 (70±5)	BT/BTL_H2 67 (59±0)	CoC_H3* 100 (72±2)	TPC_H6 47<br>peCFNC_H2 33	CoC_H5 67 (62±2)	APC_H6 100 (68±3)
**AACX OLD**	YPR011C_H1 60 (70±5)	AAC_H2 96 (59±6)	CoC_H3* 100 (72±2)	TPC_H6 67 (62±3)	CoC_H5 67 (62±2)	APC_H6 100 (68±3)
**SLC25A43**	APC_H1 43<br>CoC_H3 20	TAAC_H2 48<br>YPR011C_H2 32	OGC_H1* 35<br>APCX_H3 20	SLC25A39-A40_H2 21<br>CoC_H4 20	TPC_H3 31<br>OAC_H1 28	TPC_H4 30<br>MFT/FAD_H6 20
**SLC25A43 OLD**	APC_H1 43<br>CoC_H3 20	YPR011C_H2 66 (59±4)	OGC_H1* 47<br>APC_H3 20	SLC25A39-A40_H2 21<br>CoC_H4 20	TPC_H3 45<br>APC_H3 15	TPC_H4 30<br>MFT/FAD_H6 20
**APCX**	APC_H1 100 (75±3)	CoC_H2 27<br>AACX_H2* 25	CoC_H3* 71 (79±2)	PNC_H2 67 (69±6)	APCX_H3* 33<br>BT/BTL_H5 22	APC_H6 35<br>YPR011C_H6 34
**APCX OLD**	APC_H1 100 (75±3)	CoC_H2 40<br>APC_H2 16	CoC_H3* 71 (79±2)	SFC_H2 30<br>MFRN_H2 21	CoC_H3 45<br>CoC_H5 20	APC_H6 35<br>YPR011C_H6 34
**APC**	TAAC_H1 35<br>APCX_H1 29	YPR011C_H2 37<br>GC_H2 24	CoC_H5 16<br>AGC_H1 11	CoC_H4 77 (64±5)	CoC_H5 45<br>AGC_H1 19	AACX_H6 28<br>MFT/FAD_H6 18
**APC OLD**	YPR011C_H1 58 (70±3)	YPR011C_H2 51 (58±3)	CoC_H5 21<br>AGC_H1 13	CoC_H4 83 (63±5)	CoC_H5 48<br>AGC_H1 21	MFT_/FAD_H6 36<br>CoC_H6 25
**CoC**	**TAAC_H1 60 (70±5)**	YPR011C_H2 57 (62±6)	APCX_H3 44<br>YPR011C_H3 24	TPC_H4 39<br>APC_H4 32	**YPR011C_H5 63 (68±4)**	APC_H6 27<br>YPR011C_H6 25
**CoC OLD**	APC_H1 39<br>YPR011C_H1 23	**YPR011C_H2 63 (61±7)**	YPR011C_H3 55 (65±3)	TPC_H4 45<br>APC_H4 33	**YPR011C_H5 73 (67±5)**	APC_H6 38<br>YPR011C_H6 33
**YPR011C**	CoC_H1 61 (81±8)	APC_H2 44<br>TAAC_H2 34	APCX_H3* 41<br>CoC_H5 36	CoC_H4 38<br>BT/BTL_H4* 25	**CoC_H5 79 (71±6)**	CoC_H6 50<br>APC_H6 42
**YPR011C OLD**	CoC_H1 63 (81±8)	**CoC_H2 52 (66±4)**	CoC_H5 50<br>AAC_H3 25	AAC_H4* 50<br>CoC_H4 38	**CoC_H5 79 (71±6)**	CoC_H6 50<br>APC_H6 42
**TPC**	SLC25A43_H1 12	MFT/FAD_H4 10	SLC25A39-A40_H5	CoC_H4 42	CoC_H3 15	YMC_H6 13

	UCP5-6_H3 10	MFT/FAD_H6 10	14<br>AACX_H3 12	TAAC_H4 10	CoC_H1 10	SLC25A39-A40_H2 11
**TPC OLD**	SAMC_H5 15<br>APC_H1 14	MFT/FAD_H4 19<br>MFT/FAD_H6 10	CoC_H3 20<br>SLC25A39-A40_H5 18	CoC_H4 52 (62±6)	CoC_H3 19<br>CoC_H1 12	YMC_H6 13<br>SLC25A39-A40_H2 12
**BT/BTL**	SAMC_H5* 40<br>YPR011C 35	APC_H2 20<br>AAC_H2 20	MFT/FAD_H1 37<br>ODC_H1 18	PNC_H2 45<br>TAAC_H4 20	CoC_H5 52 (62±2)	APC_H6 52 (56±7)
**BT/BTL OLD**	SAMC_H5* 40<br>YPR011C 39	APC_H2 40<br>AAC_H2 20	MFT/FAD_H1 47<br>AGC_H3 20	APC_H4 30<br>SLC25A39-A40_H2 27	CoC_H5 61 (61±2)	APC_H6 52 (56±7)
**c. MC-CA**						
**OAC**	MFRN_H5 17<br>PNC_H1 13	AT4G03115_H2 28<br>OGC_H2 15	SLC25A39-A40_H5 16<br>AGC_H1 14	TAAC_H2 23<br>AT4G03115_H4 19	GC_H5 12<br>SLC25A39-A40_H5 11	OGC_H6 41<br>DIC_H6 25
**OAC OLD**	MFRN_H5 25<br>SLC25A39-A40_H5 12	UCP4_H2 28<br>OGC_H2 21	SLC25A39-A40_H5 25<br>AGC_H1 24	OGC_H4 28<br>AAC_H6 14	SLC25A39-A40_H5 29<br>UCP4_H5 8	OGC_H6 44<br>DIC_H6 36
**AT4G03115**	-	UCP4_H2 56 (59±0)	-	-	-	OGC_H6 63 (61±3)
**AT4G03115 OLD**	-	UCP4_H2 56 (59±0)	-	-	SLC25A44_H5 43<br>NDT_H3 20	OGC_H6 67 (61±3)
**UCP2-3**	UCP4_H1 32<br>DIC_H1 26	DIC_H2 36<br>AT4G03115_H2 28	**UCP4_H3 52 (61±5)**	UCP4_H4 62 (52±5)	DIC_H5 41<br>SLC25A39-A40_H1 22	**UCP5-6_H6 82 (61±6)**
**UCP2-3 OLD**	UCP4_H1 37<br>SAMC_H1 31	DIC_H2 37<br>OGC_H2 29	**UCP4_H3 68 (60±6)**	**UCP4_H4 70 (52±5)**	DIC_H5 48<br>SLC25A39-A40_H1 22	DIC_H6 80 (54±4)
**UCP5-6**	UCP4_H1 46<br>DIC_H1 17	OGC_H2 83 (59±5)	GlyC_H5 18<br>TPC_H1 14	UCP4_H4 80 (59±5)	DIC_H5 73 (65±6)	**UCP2-3_H6 58 (66±5)**
**UCP5-6 OLD**	UCP4_H1 46<br>DIC_H1 23	OGC_H2 83 (59±5)	GlyC_H5 26<br>UCP2-3_H3 22	UCP4_H4 80 (59±5)	DIC_H5 79 (64±6)	UCP2-3_H6 59 (66±5)
**UCP4**	UCP5-6_H1 46<br>DIC_H5 20	AT4G03115_H2 42<br>DIC_H2 19	**UCP2-3_H3 71 (65±4)**	UCP2-3_H4 45<br>UCP5-6_H4 37	SLC25A39-A40_H5* 30<br>DIC_H5 24	UCP5-6_H6 92 (58±6)

**UCP4 OLD**	DIC_H5 28<br>UCP2-3_H1 21	DIC_H2 39<br>UCP2-3_H2 12	**UCP2-3_H3 71 (64±4)**	**UCP2-3_H4 76 (54±3)**	SLC25A39-A40_H5* 30<br>DIC_H5 24	SLC25A44_H4 24<br>DIC_H6 23
**DIC**	APC_H1 23<br>AT4G03115_H1 14	**OGC_H2 99** (65±7)	OGC_H3 34<br>OAC_H3 18	OGC_H4 30<br>AT4G03115_H4 27	SAMC_H5 17<br>UCP5-6_H5 13	OGC_H6 42<br>OAC_H6 40
**DIC OLD**	APC_H1 46<br>OGC_H1 20	**OGC_H2 100** (65±7)	OGC_H3 44<br>ALC_H1 27	UCP4_H4 41<br>OGC_H4 37	SLC25A39-A40_H5 22<br>SAMC_H5 17	**OGC_H6 76** (61±7)
**OGC**	OAC_H1 27<br>APC_H1 19	**DIC_H2 94** (75±3)	DIC_H3 74 (61±5)	DIC_H4 83 (63±5)	DIC_H5 31<br>AT4G03115_H5* 25	OAC_H6 53 (71±11)
**OGC OLD**	CoC_H1 33<br>APC_H1 19	**DIC_H2 100** (74±3)	DIC_H3 98 (60±5)	DIC_H4 83 (63±5)	DIC_H5 52 (59±3)	**DIC_H6 82** (71±12)
**d. MC-AAP**						
**ALC**	SLC25A45/A47/A48_H1 38<br>ORC_H1 37	CAC_H2 38<br>YMC_H2 23	GC_H3 23<br>APC_H3 20	CAC_H4 23<br>GlyC_H4 18	**YMC/BOU_H5 54 (68±6)**	SLC25A45/A47/A48_H6 37<br>ORC_H6 26
**ALC OLD**	**ORC_H1 78 (73±9)**	YMC/BOU_H2 51 (56±8)	APC_H3 33<br>ORC_H3 19	GlyC_H4 33<br>ORC_H4 20	**YMC/BOU_H5 68 (66±6)**	ORC_H6 62 (63±5)
**SLC25A45/A47/A48**	ALC_H1 78 (72±5)	ALC_H2 49<br>YMC/BOU_H2 22	YMC/BOU_H1 14<br>ALC_H3 10	CAC_H4 40<br>AGC_H4 34	ALC_H5 43<br>CAC_H5 13	ALC_H6 56 (63±9)
**SLC25A45/A47/A48 OLD**	ALC_H1 78 (72±5)	ALC_H2 49<br>YMC/BOU_H2 22	YMC/BOU_H1 14<br>ALC_H3 10	CAC_H4 40<br>AGC_H4 34	ALC_H5 45<br>CAC_H5 13	ALC_H6 56 (63±9)
**CAC**	ALC_H1 42<br>SLC25A45/A47/A48 32	ALC_H2 52 (57±4)	ORC_H3 48<br>GlyC_H5 10	GC_H4 40<br>SLC25A39-A40_H4 30	SLC25A45/A47/A48 47<br>ALC_H5 18	ORC_H6 46<br>YMC/BOU_H6 34
**CAC OLD**	ALC_H1 59 (67±4)	ALC_H2 71 (56±4)	ORC_H3 53 (52±2)	SLC25A39-A40_H4 44<br>GlyC 21	TPC_H1 28<br>ALC_H5 26	ORC_H6 46<br>YMC/BOU_H6 34
**ORC**	ALC_H1 47<br>YMC/BOU_H1 37	MCART_H4 25<br>YMC/BOU_H2 13	**YMC/BOU_H3 65 (59±5)**	ALC_H4 38<br>YMC/BOU_H4 37	YMC/BOU_H5 28<br>ALC_H5 21	CAC_H6 38<br>SLC25A39/A40 15
**ORC OLD**	**ALC_H1 56 (73±7)**	YMC/BOU_H2 28<br>ALC_H2 9	**YMC/BOU_H3 71 (59±6)**	YMC/BOU_H4 41<br>ALC_H4 38	YMC/BOU_H5 34<br>ALC_H5 27	YMC/BOU_H6 34<br>ALC_H6 26
**YMC/BOU**	ALC_H1 55 (74±2)	ALC_H2 42	**ORC_H3 56 (63±7)**	ORC_H4 51 (64±9)	**ALC_H5 63 (69±4)**	CAC_H6 50

		PiC_H6 19				ORC_H6 18
**YMC/BOU OLD**	ALC_H1 55 (74±2)	ALC_H2 50<br>PiC_H6 25	**ORC_H3 56 (63±7)**	ORC_H4 52 (64±9)	**ALC_H5 75 (68±4)**	ORC_H6 63 (61±5)
**e. MC-AAN**						
**AGC**	**GC_H1 68 (73±5)**	**GC_H2 56 (63±5)**	BT_H3 36<br>OAC_H1 34	GC_H4 98 (74±3)	GC_H5 60 (73±3)	ALC_H4 38<br>SLC25A39-A40_H6 16
**AGC OLD**	APC_H3* 87 (71±4)	SLC25A39-A40_H6 57 (57±4)	NDT_H3 27<br>YPR011C_H3 21	SLC25A39-A40_H4 66 (65±6)	MFRN_H5 60 (69±8)	ALC_H4 46<br>SLC25A39-A40_H6 21
**GC**	**AGC_H1 62 (68±4)**	**AGC_H2 54 (62±5)**	AGC_H3 66 (60±3)	SLC25A39-A40_H4 37<br>AGC_H4 30	SLC25A39-A40_H5 28<br>AGC_H5 21	SLC25A39-A40_H6 39<br>AGC_H6 22
**GC OLD**	AGC_H1 82 (67±5)	AGC_H2 59 (62±5)	AGC_H3 66 (60±3)	SLC25A39-A40_H4 47<br>AGC_H4 36	SLC25A39-A40_H5 35<br>AGC_H5 22	SLC25A39-A40_H6 40<br>AGC_H6 29
**f. MCs outside the main clusters**						
**SFC**	CAC_H1 36<br>SAMC_H1 15	AT4G03115_H2 50<br>CIC_H2 26	**CIC_H3 97 (66±6)**	**CIC_H4 68 (57±5)**	AT4G11440_H5 20<br>SLC25A44_H5 14	**ODC_H6 64 (59±5)**
**SFC OLD**	SAMC_H1 24<br>TPC_H5 21	ALC_H2 31<br>SLC25A39_A40_H3 20	SLC25A39_A40_H1 61 (52±4)	DIC_H6 23<br>NDT_H2 14	SLC25A39_A40_H5 45<br>SLC25A44_H5 14	SLC25A39_A40_H6 42<br>YMC_H4 14
**CIC**	ODC_H1 67 (56±3)	SFC_H2 58 (58±4)	**SFC_H3 100 (64±4)**	**SFC_H4 62 (56±5)**	AT5G42130_H5 33<br>GC_H5 21	MFT/FAD_H6 53 (51±4)
**CIC OLD**	MFRN_H1 37<br>SAMC_H1 15	SFC_H2 76 (58±4)	SFC_H3 100 (64±4)	SFC_H4 64 (56±5)	SAMC_H5 59 (66±4)	MFT/FAD_H6 56 (51±4)
**ODC**	**MFRN_H1 51 (70±6)**	CAC_H2 53 (53±3)	GC_H5 64 (62±5)	GC_H4 66 (56±5)	SLC25A39-A40_H5 55 (61±4)	**SFC_H6 52 (57±4)**
**ODC OLD**	**MFRN_H1 86 (68±6)**	NDT_H2* 46<br>MFT/FAD 13	SLC25A39-A40_H5 32	GlyC_H4 21<br>ORC_H4 20	SLC25A39-A40_H5 58 (60±5)	SFC_H6 53 (57±4)

			SFC_H3 24			
**SLC25A39-A40**	GC_H5 25<br>NDT_H1 8	ORC_H6 20<br>GC_H4 13	BT_H1 26<br>APCX_H3 11	GC_H4 42<br>GlyC_H4 39	SAMCX_H5 38<br>MFRN_H5 16	APC_H6 17<br>MFRN_H6 17
**SLC25A39-A40 OLD**	TPC_H1 19<br>NDT_H1 12	ORC_H6 36<br>NDT_H2 17	NDT_H1 16<br>APCH3 15	**GlyC_H4 60 (73±7)**	MFRN_H5 37<br>SAMC_H5 34	APC_H6 20<br>MFRN_H6 18
**GlyC**	OGC_H1* 39<br>NDT_H1 7	ALC_H4 20<br>SLC25A45/A47/A48 17	SLC25A39-A40_H5 26<br>NDT_H1 21	SLC25A39-A40_H4 51 (74±6)	MFRN_H5 35<br>SLC25A39-A40_H5 29	MFRN_H6 40<br>SAMC_H6 10
**GlyC OLD**	OGC_H1* 39<br>NDT_H1 8	ALC_H4 21<br>SLC25A39-A40 11	SLC25A39-A40_H5 28<br>NDT_H1 21	**SLC25A39-A40_H4 76 (72±7)**	SLC25A39-A40_H5 38<br>MFRN_H5 36	MFRN_H6 46<br>SAMC_H6 14
**SLC25A44**	APC_H5 22<br>SAMC_H1 21	AAC_H4 21<br>MFRN_H2 10	MFRN_H3 21<br>MTCH_H5 13	AAC_H2 19<br>SLC25A39-A40 14	CoC_H3 37<br>MFRN_H5 13	SLC25A39-A40 35<br>AT5G42130_H6 9
**SLC25A44 OLD**	APC_H5 22<br>SAMC_H1 22	AAC_H4 28<br>MFRN_H2 17	MFRN_H3 27<br>CoC_H5 19	AAC_H2 19<br>AGC_H4 15	CoC_H3 40<br>MFRN_H5 20	SLC25A39-A40 38<br>GlyC_H4 9
**MFRN**	**ODC_H1 77 (70±7)**	ALC_H4 20<br>SLC25A39-A40 11	ODC_H3 21<br>SLC25A44_H3 21	SAMC_H4 20<br>SLC25A39-A40_H2 15	SAMC_H5 44<br>SLC25A39-A40_H5 21	SLC25A39-A40_H6 47<br>PiC_H6 10
**MFRN OLD**	SAMC_H1 28<br>AGC_H1 16	ALC_H4 28<br>SLC25A39-A40 21	SAMC_H3 18<br>GlyC_H5 16	SAMC_H4 32<br>SLC25A39-A40_H2 19	**SAMC_H5 65 (77±6)**	SLC25A39-A40_H6 52 (53±4)
**SAMC**	AT4G11440_H1 42<br>CAC_H5 19	MME_H2 30<br>A33/A36_H2 19	AT5G42130_H3* 50<br>MME_H3 10	GC_H4 36<br>MME_H4 31	MFRN_H5 68 (77±4)	ODC_H6 32<br>MME1_H4 14
**SAMC OLD**	MFRN_H1 31<br>NDT_H1 12	MFRN_H2 17<br>MFT/FAD_H2 12	SFC_H3 25<br>ORC_H3 19	SLC25A39-A40_H4 31<br>AGC_H4 27	**MFRN_H5 94 (77±5)**	SFC_H6 27<br>MFT/FAD_H4 9
**SAMCX**	GlyC_H1 33<br>MFRN_H1 17	MFT/FAD_H2 50<br>OAC_H2 50	AT1G74240_H3 40<br>MFRN_H3 26	GC_H4 94 (57±3)	SLCA39-A40_H5 65 (79±4)	TAAC_H4 33<br>SAMC_H6 21
**SAMCX OLD**	GlyC_H1 50<br>MFRN_H1 17	MFT/FAD_H2 75 (47±0)	SAMC_H3 39<br>MFRN_H3 32	SAMC_H4 43<br>ORC_H4 43	SLCA39-A40_H5 69 (79±4)	SAMC_H6 54 (47±0)
**AT5G42130**	MFRN_H1 38<br>SAMC_H1 36	MFRN_H1 33<br>SAMC_H1 33	SAMC_H3 56 (55±5)	APC_H6* 67 (59±0)	SAMC_H5 56 (85±0)	SAMC_H2 38<br>GlyC_H2 17
**AT5G42130 OLD**	SAMC_H1 56 (63±3)	-	SAMC_H3 83 (52±6)	APC_H6* 67 (59±0)	SAMC_H5 56 (85±0)	SAMC_H2 38<br>GlyC_H2 17

**AT4G11440**	SAMC_H1 78 (68±2)	SAMC_H2 46<br>PNC_H2 38	SLC25A44_H3 44<br>ORC_H3 17	MCART_H4* 78 (65±0)	SLC25A39-A40 78 (78±2)	CIC_H6 33<br>MCART_H2 15
**AT4G11440 OLD**	SAMC_H1 100 (68±2)	SAMC_H2 63 (51±3)	SLC25A44_H3 46<br>ORC_H3 25	AGC_H4 45<br>SFC_H2 19	-	PiC_H2 43<br>SLC25A39-A40 19
**PiC**	TPC_H5 25<br>MFRN_H5 16	AT4G11440_H6 27<br>SAMC_H6 12	UCP2-3_H1 28<br>ORC_H3 21	DIC_H6* 66 (54±4)	CAC_H5 26<br>MFRN_H1 17	MFRN_H6 43<br>TPC_H4 17
**PiC OLD**	TPC_H5 25<br>AGC_H1 11	CoC_H4 21<br>SAMC_H6 15	UCP2-3_H1 31<br>ORC_H3 29	DIC_H6* 72 (54±4)	MFRN_H1 19<br>SAMC_H1 19	MFRN_H6 46<br>TPC_H4 20
**SLC25A46**	ODC_H3 31<br>TPC_H1 13	APCX_H4 21<br>ALC_H4 12	AAC_H6 17<br>SAMC_H5 10	ODC_H3 13<br>AAC_H4 9	peANT 16<br>MFRN_H3 13	AAC_H6 27<br>peANT 16
**SLC25A46 OLD**	ODC_H3 34<br>TPC_H1 13	ALC_H4 19<br>OAC_H2 13	AAC_H6 17<br>SAMC_H5 10	AAC_H4 19<br>ODC_H3 17	PiC_H3 23<br>peANT 18	AAC_H6 27<br>peANT 16
**UGO**	ORC_H1 25<br>GC_H5 25	SLC25A44_H6 25<br>GlyC_H1 21	SLC25A39-A40_H1 25<br>UCP2-3_H5 25	SLC25A45/A47/A48_H2 31<br>SLC25A39-A40_H3 13	SLC25A46 42<br>ALC_H3 25	MFT/FAD_H2 25<br>UCP2-3_H3 25
**UGO OLD**	ORC_H1 25<br>YMC_H1 13	GlyC_H1 38<br>PiC_H2 25	SLC25A39-A40_H1 25<br>UCP2-3_H5 25	SAMC_H3 21<br>MFRN_H1 17	ALC_H3 25<br>GlyC_H2 25	MFT/FAD_H2 25<br>UCP2-3_H3 25
**MTCH**	OGC_H1 10<br>SLC25A44_H1 8	SLC25A39-A40_H2 18<br>SLC25A44_H6 8	OAC_H5 7<br>ALC_H1 7	MFT/FAD_H2 15<br>APC_H4 10	TPC_H5 18<br>peCFNC_H5 15	SLC25A43_H2* 27<br>SAMC_H6 8
**MTCH OLD**	OGC_H1 10<br>SLC25A44_H1 8	SLC25A39-A40_H2 21<br>MFT/FAD_H2 12	OAC_H5 8<br>ALC_H1 7	MFT/FAD_H2 15<br>APC_H4 13	TPC_H5 18<br>peCFNC_H5 15	SAMC_H6 9<br>AAC_H5 8
**MCART**	GC_H3 20<br>AGC_H3 12	peCFNC_H6 20<br>ODC_H2 20	PNC_H1 17<br>MFRN_H6 11	UCP5-6_H6 15<br>CAC_H6 13	NDT_H1 11<br>YMC_H5 11	MFT/FAD_H2 26<br>CIC_H6 25
**MCART OLD**	CIC_H1 16<br>AGC_H3 12	ODC_H2 26<br>MFT/FAD_H2 22	ODC_H3 20<br>PNC_H1 17	CAC_H6 18<br>UCP2-3_H6 14	APC_H3 15<br>NDT_H1 11	MFT/FAD_H2 27<br>CIC_H6 25
**MME**	SAMCX_H1* 67 (63±3)	SAMC_H2 45<br>peANT_H6 36	SAMC_H3 53 (54±2)	SAMC_H4 41<br>SLC25A39-A40_H4 19	MFRN_H5 60 (78±3)	SAMC_H6 44<br>SFC_H6 15
**MME OLD**	SAMC_H1 31<br>MFRN_H1 27	SAMC_H2 45<br>peANT_H6 36	SAMC_H3 62 (54±2)	SAMC_H4 50<br>SLC25A39-A40_H4	MFRN_H5 60 (78±3)	SAMC_H6 51 (49±3)

				19		
**AT4G15010**	SLC25A44_H5 33<br>AGC_H1 33	SAMC_H2 46<br>CIC_H2 33	YMC_H1 33<br>MFT/FAD_H1 33	SLC25A38_H4 33<br>GC_H4 33	UCP2-3_H1 44<br>CAC_H1 22	MFRN_H6 33<br>YMC_H6 18
**AT4G15010 OLD**	SLC25A44_H5 33<br>AGC_H1 33	SAMC_H2 51 (47±5)	-	UCP2-3_H2 53<br>(47±0)	UCP2-3_H1 67<br>(53±3)	MFRN_H6 33<br>YMC_H6 18
**GGC**	MFRN_H1 42<br>CAC_H5 30	PiC_H2 60 (35±0)	SLC25A39-A40_H1<br>32<br>TPC_H1 32	ORC_H4 48<br>CAC_H4 32	NDT_H1 63 (55±0)	MCART_H4 95<br>(63±3)
**GGC OLD**	MFRN_H1 50<br>SFC_H1 27	PiC_H2 64 (34±3)	SLC25A39-A40_H1<br>32<br>TPC_H1 32	ORC_H4 94 (53±0)	NDT_H1 63 (55±0)	SLC25A39-A40_H2<br>26<br>CoC_H4 16
**YHM2**	GlyC_H1 60 (52±5)	SLC25A39-A40_H1<br>46<br>peANT_H1 46	PiC_H4* 75 (40±0)	SFC_H4* 78 (51±3)	PNC_H5 50<br>UCP5-6_H3 14	SLC25A39-A40_H6<br>50<br>UCP4_H2 25
**YHM2 OLD**	GlyC_H1 63 (52±5)	SLC25A39-A40_H1<br>46<br>peANT_H1 46	PiC_H4* 75 (40±0)	SFC_H4* 78 (51±3)	YMC_H3 31<br>ORC_H1 31	SLC25A39-A40_H6<br>50<br>UCP4_H2 25
